# Cryptic enzymatic assembly of peptides armed with β-lactone warheads

**DOI:** 10.1038/s41589-024-01657-7

**Published:** 2024-07-01

**Authors:** Guangcai Xu, Daniele Torri, Sebastian Cuesta-Hoyos, Deepanjan Panda, Luke R. L. Yates, Rémi Zallot, Kehan Bian, Dongxu Jia, Andreea I. Iorgu, Colin Levy, Sarah A. Shepherd, Jason Micklefield

**Affiliations:** Department of Chemistry and Manchester Institute of Biotechnology, The University of Manchester, Manchester, UK

**Keywords:** Biocatalysis, Biosynthesis, Natural products, Enzymes, Peptides

## Abstract

Nature has evolved biosynthetic pathways to molecules possessing reactive warheads that inspired the development of many therapeutic agents, including penicillin antibiotics. Peptides armed with electrophilic warheads have proven to be particularly effective covalent inhibitors, providing essential antimicrobial, antiviral and anticancer agents. Here we provide a full characterization of the pathways that nature deploys to assemble peptides with β-lactone warheads, which are potent proteasome inhibitors with promising anticancer activity. Warhead assembly involves a three-step cryptic methylation sequence, which is likely required to reduce unfavorable electrostatic interactions during the sterically demanding β-lactonization. Amide-bond synthetase and adenosine triphosphate (ATP)-grasp enzymes couple amino acids to the β-lactone warhead, generating the bioactive peptide products. After reconstituting the entire pathway to β-lactone peptides in vitro, we go on to deliver a diverse range of analogs through enzymatic cascade reactions. Our approach is more efficient and cleaner than the synthetic methods currently used to produce clinically important warhead-containing peptides.

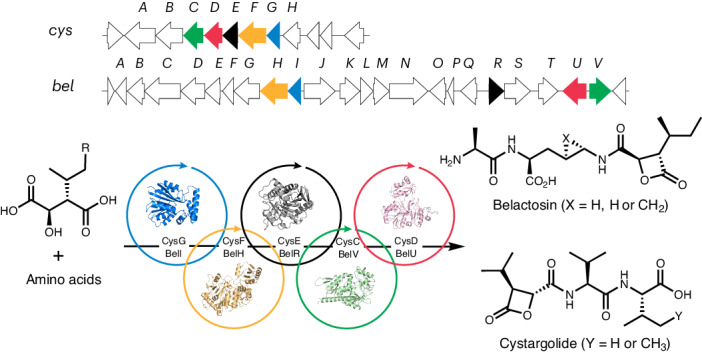

## Abstract (web summary)

Total in vitro biosynthesis can reveal unusual pathways evolved by nature to produce natural products. Here the authors report on enzymatic cascades, comprising a cryptic methylation sequence, efficiently delivering β-lactone-containing peptide proteasome inhibitors with promising anticancer activity.

## Main

**Fig. 1:**
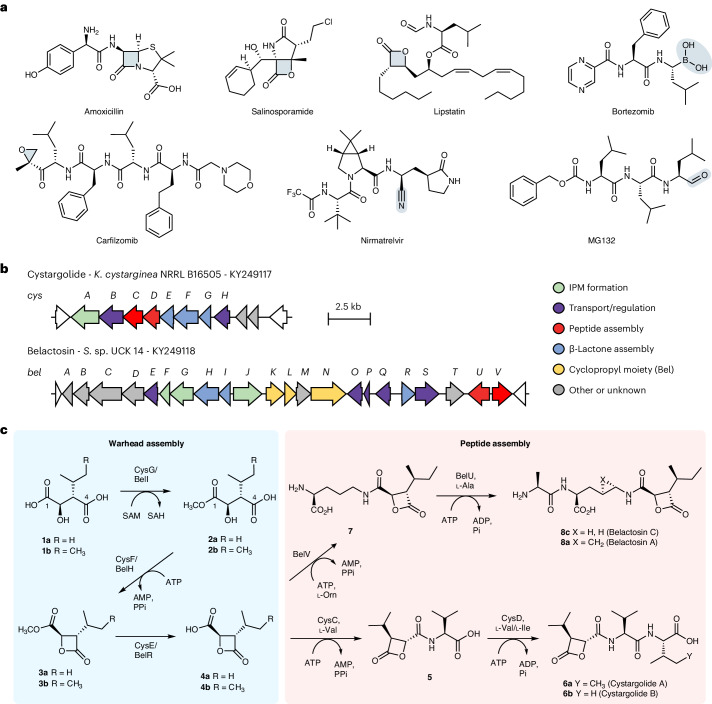
Warhead-containing therapeutic agents and biosynthesis of β-lactone-containing peptides. **a**, Examples of natural products and synthetic derivatives bearing electrophilic warheads. **b**, The cys and bel BGCs, including revisions based on this work. IPM, isopropyl malic acid. **c**, The biosynthetic pathways to cys and bel based on the in vitro characterization of the complete set of ten pathway enzymes CysGFECD and BelIHRVU, as described in this study. AMP, adenosine monophosphate; ADP, adenosine diphosphate; SAH, *S*-adenosylhomocysteine.

Covalent inhibitors with reactive functionality (warheads) that bond irreversibly with a biological target have been widely used as therapeutic agents and are becoming increasingly important in modern drug discovery^1–6^. The design of this class of inhibitors is often inspired by natural products^7–10^. For example, the discovery of penicillin, over 90 years ago, led to many semisynthetic antibiotics with reactive β-lactam rings, which remain the most widely prescribed class of antibiotics today (Fig. 1a)^11,12^. Peptide natural products with epoxyketone warheads inspired the discovery of carfilzomib, a proteasome inhibitor that was approved for the treatment of cancer^10^ and provided payloads for antibody–drug conjugates^13^. Peptides armed with aldehydes, vinyl sulfones, nitriles and other electrophilic warheads have also provided many other antimicrobial, anticancer and antiviral agents^1–10,14^, including nirmatrelvir, which was recently approved to treat COVID-19 (ref. ^6^). Most of these important therapeutic agents are currently produced by chemical synthesis. The chemical synthesis of peptides, particularly those containing reactive functionality, can be challenging. Although peptide synthesis works well on a small scale, multistep processes using an excess of deleterious reagents, atom-inefficient protecting groups and large volumes of solvents are problematic to implement at the scale required for peptide therapeutics^15–17^. By virtue of their benign operating conditions, selectivity and evolvability^18^, enzymes could provide more efficient and sustainable routes, making these medically valuable agents more widely available. Exploring the biosynthesis of warhead-containing peptide natural products could, therefore, provide important new enzymes that can be used to produce peptide therapeutics.

Several natural products have been discovered with β-lactone warheads^19^, including antiobesity drug lipstatin^20^ and the proteasome inhibitor salinosporamide, which is in clinical trials for the treatment of cancer^21^. β-Lactone-containing peptides cystargolide (cys) and belactosin (bel) have also been isolated from *Kitasatospora cystarginea* and *Streptomyces* sp. UCK 14, respectively^22,23^. The promising antitumor activity of cys and bel stimulated extensive medicinal chemistry efforts leading to the synthesis of improved derivatives^24–31^, including cys analogs that were more potent proteasome inhibitors than carfilzomib when tested in vitro^29,30^. The cys and bel biosynthetic gene clusters (BGCs) have been sequenced and the putative functions of pathway enzymes were proposed on the basis of bioinformatics analysis (Fig. 1b)^32^. The biosynthetic origin of the malic acid and cyclopropyl ornithine precursors of cys and bel have also been explored^32–36^. However, the enzymes required for assembly of the β-lactone-containing peptides have not been characterized.

In this paper, we provide complete in vitro characterization of the entire set of ten enzymes required for cys and bel biosynthesis, revealing cryptic pathways to reactive β-lactone intermediates and unanticipated timing of peptide-bond-forming steps (Fig. 1c). Our experiments show that the functions of all cys and bel biosynthetic enzymes differ from those predicted in silico, illustrating the unpredictable and unusual nature of the biosynthetic pathways. We also demonstrate how the cys and bel enzymes can be combined in cascade reactions to produce many peptides with different warheads.

## Results

### CysF catalyzes β-lactone formation

**Fig. 2:**
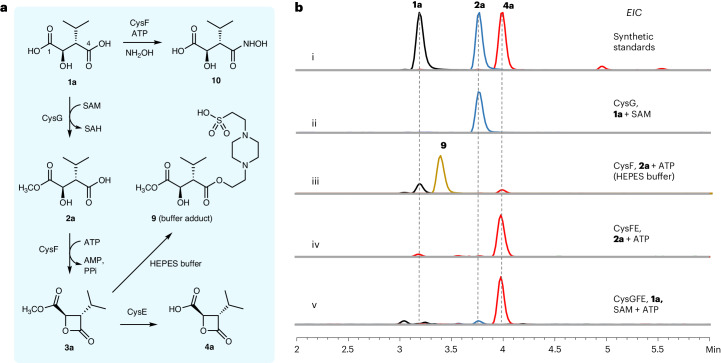
In vitro characterization of the β-lactone assembly enzymes CysGFE. **a**, Confirmed pathway for β-lactone assembly based on in vitro characterization of the CysGFE enzymes. **b**, LC–MS analysis for the in vitro reactions of CysGFE. EIC, extracted ion chromatogram.

**Extended Data Fig. 1:**
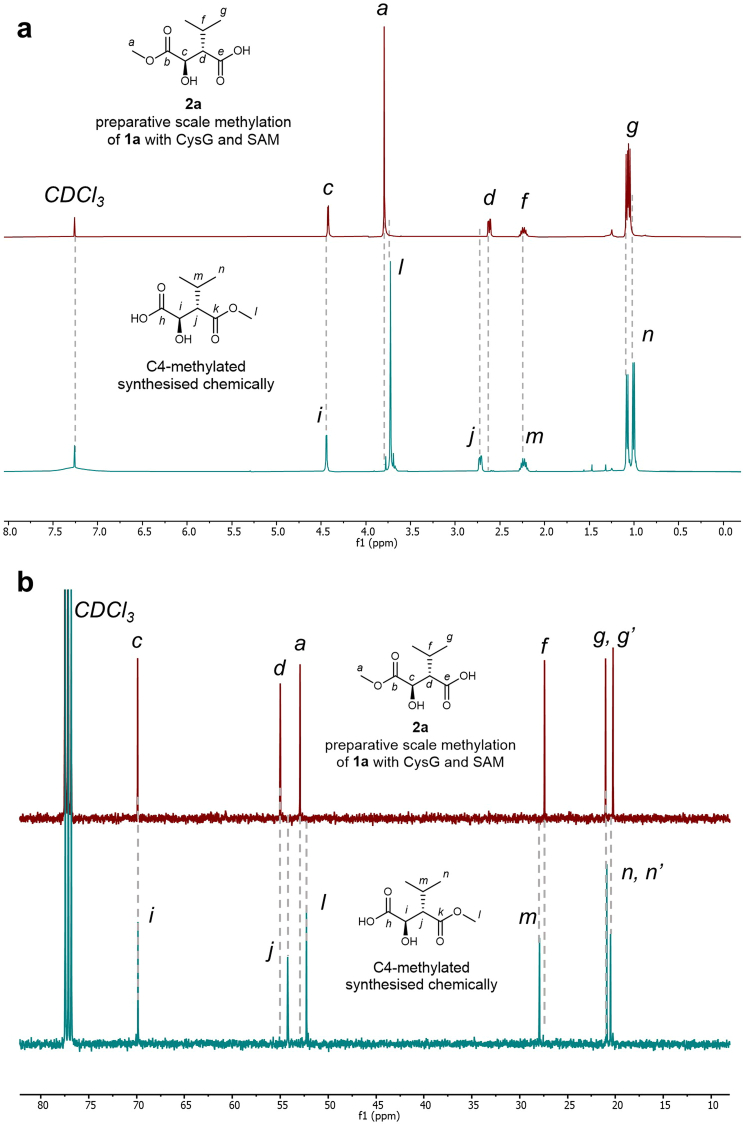
Comparison of the NMR spectra between enzymatic (CysG) product 2a and the synthetic C4-methyl ester. **a**,^1^H NMR spectra. **b**,^13^C NMR spectra.

**Extended Data Fig. 2:**
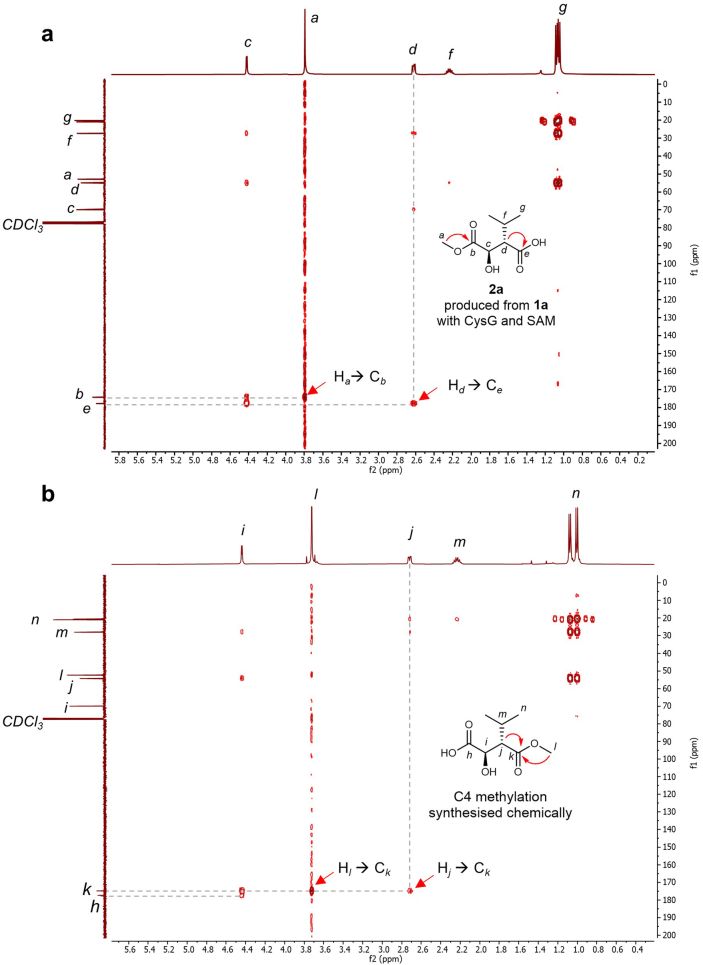
Comparison of the HMBC spectra of 2a (enzymatic) and C4 methyl ester. **a**, HMBC spectra of **2a** produced from **1a** with CysG and SAM. **b**, HMBC spectra of synthetic C4-methyl ester standard.

The presence of orthologs of the well-known primary metabolic enzyme isopropyl malic acid synthetase (IPMS) within the cys and bel BGCs led to the proposal that malic acid derivatives (**1a** and **1b**) are likely β-lactone precursors^32^. It was suggested that the putative methyltransferases CysG and BelI methylate the C4-carboxylates of **1a** and **1b**, respectively, to generate C4-methyl ester intermediates that are cyclized to β-lactones **4a** and **4b** by putative hydrolases CysE and BelR^32^. Alternatively, it was suggested that CysC and BelV, which show similarity to adenosine triphosphate (ATP)-dependent acyl-CoA synthetases, may activate **1a** and **1b**, forming C4-CoA thioesters facilitating cyclization to **4a** and **4b**^32^. To explore the cys and bel pathways, the proposed (2*R*,3*S*)-malic acid precursors (**1a** and **1b**; Fig. 1c) were synthesized as described previously (Supplementary Fig. 1)^37^. Ten enzymes potentially involved in the assembly of bel and cys from the malic acid precursors (CysCDEFG and BelHIRUV) were overproduced in *Escherichia coli* and purified (Supplementary Fig. 2 and Supplementary Tables 1 and 2). First, we incubated **1a** with the putative acyl-CoA synthetase CysC and ATP, with and without CoASH but this failed to produce β-lactone **4a** as proposed^32^ (Supplementary Fig. 3). Next, we incubated **1a** with the putative methyltransferase CysG, *S*-adenosyl methionine (SAM) and hydrolase CysE, which also failed to produce the β-lactone **4a**. The CysG reaction of **1a** and SAM did result in the formation of a methyl ester with the same mass as the proposed C4-methyl ester intermediate. However, detailed nuclear magnetic resonance (NMR) analysis and comparisons with an authentic synthetic standard showed that CysG exclusively produced the C1-methyl ester **2a** from **1a** and SAM (Fig. 2a and Extended Data Figs. 1 and 2).

**Extended Data Fig. 3:**
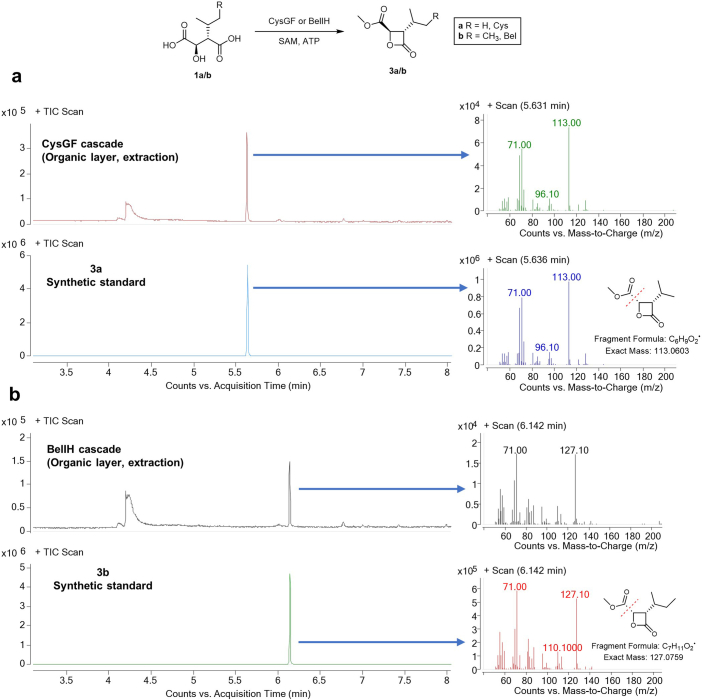
GC-MS analysis of the CysGF and BelIH cascade confirmed the formation of 3a/b. **a**, Comparison of the GC-MS spectra between CysGF cascade and **3a** synthetic standard. **b**, Comparison of the GC-MS spectra between BelIH cascade and **3b** synthetic standard.

**Extended Data Fig. 4:**
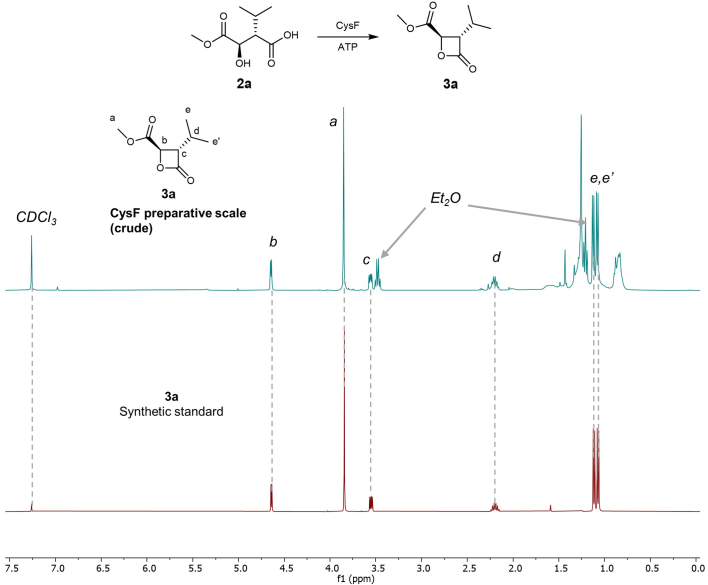
Comparison of the ^1^H NMR of 3a prepared enzymatically and chemically. **3a** was synthesised enzymatically with CysF, **2a**, and ATP, and chemically by methylation of **4a** using TMS-diazomethane (column purified). See supplementary methods for detail experiment procedures.

A SAM-dependent methyltransferase has been shown to activate a carboxylic acid precursor for subsequent amide-bond formation^38^. We are unaware, however, of any other related examples of SAM-dependent carboxyl activation. The cyclization of malic acid C4-methyl ester to a strained β-lactone (**4a**), as suggested^32^, is also thermodynamically unfavorable. We, therefore, reasoned that CysG, rather than activating the C4-carboxyl^32^, may function to mask the C1-carboxylic acid as a methyl ester (**2a**) with one of the other putative ATP-dependent enzymes in the cys BGC (CysC, CysD or CysF) catalyzing β-lactonization of **2a** to **3a** instead (Fig. 2a). Incubation of **2a** with CysC or CysD and ATP failed to give any products. The reaction of CysF, a putative adenylate (AMP)-forming enzyme, with **2a** and ATP did result in a new product but liquid chromatography–mass spectrometry (LC–MS) and NMR analysis showed that this was a C4-adduct formed from a reaction with the HEPES buffer component (**9**) (Fig. 2a,b and Supplementary Fig. 4). This suggested that lactone ester **3a** may have been produced but was attacked by the HEPES nucleophile because of the reactive nature of the β-lactone ring. To address this, we changed the buffer to MOPS or potassium phosphate and **2a** was again consumed in the CysF reaction with ATP. No β-lactone ester **3a** or adducts were evident by LC–MS, which may have been because of instability or poor ionization of **3a** under the analysis conditions. Gas chromatography (GC)–MS analysis did, however, reveal a product with the same retention time, mass and fragmentation pattern as a synthetic standard of **3a** (Extended Data Fig. 3 and Supplementary Fig. 1). A preparative-scale reaction of **2a** and ATP catalyzed by CysF was carried out, extracted with diethyl ether and rapidly subjected to ^1^H NMR analysis, confirming the structure of the hydrolytically labile β-lactone **3a** (Extended Data Fig. 4). These results showed that CysF is responsible for β-lactonization, rather than functioning as an amide or peptide synthetase as initially proposed^32^.

### β-Lactone assembly involves a cryptic three-step sequence

**Fig. 3:**
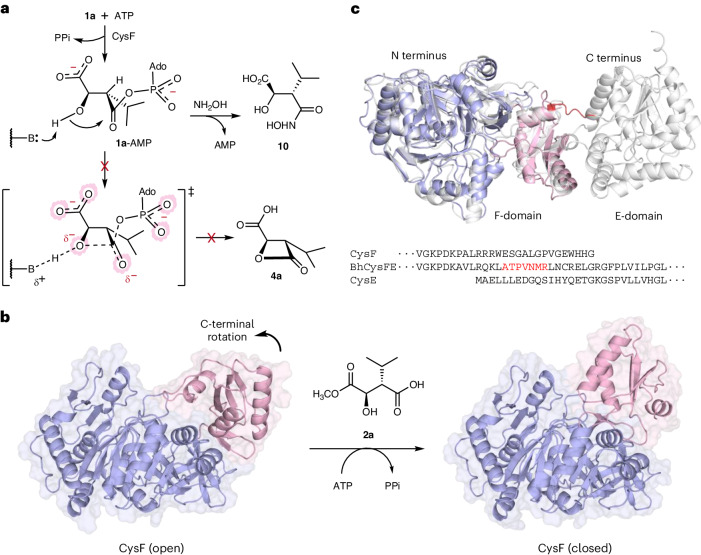
Structural and mechanistic considerations for CysF β-lactone formation. **a**, Adenylation of **1a** by CysF results in **1a**-AMP, which can be intercepted by hydroxylamine to give **10** but does not result in β-lactone formation (**4a**), possibly because of electrostatic repulsion in the strained four-membered ring transition state. **b**, X-ray crystal structure of CysF (1.9 Å) in the open adenylation state (PDB 8RA0). CysF has a large N-terminal region (residues 1–378), shown in blue, and a small C-terminal region (residues 379–491), shown in pink. A model of CysF in the closed state is also shown, which is based on similar conformational changes in structurally related adenylate-forming enzymes. **c**, AlphaFold model of the bifunctional fusion enzyme BhCysFE in gray (Extended Data Fig. 8), with the CysF X-ray crystal structure overlaid in blue and pink. The protein sequence alignment indicates a short linker (highlighted in red) in the fused enzyme. Protein structures were visualized with PyMOL (version 2.5.7).

**Extended Data Fig. 5:**
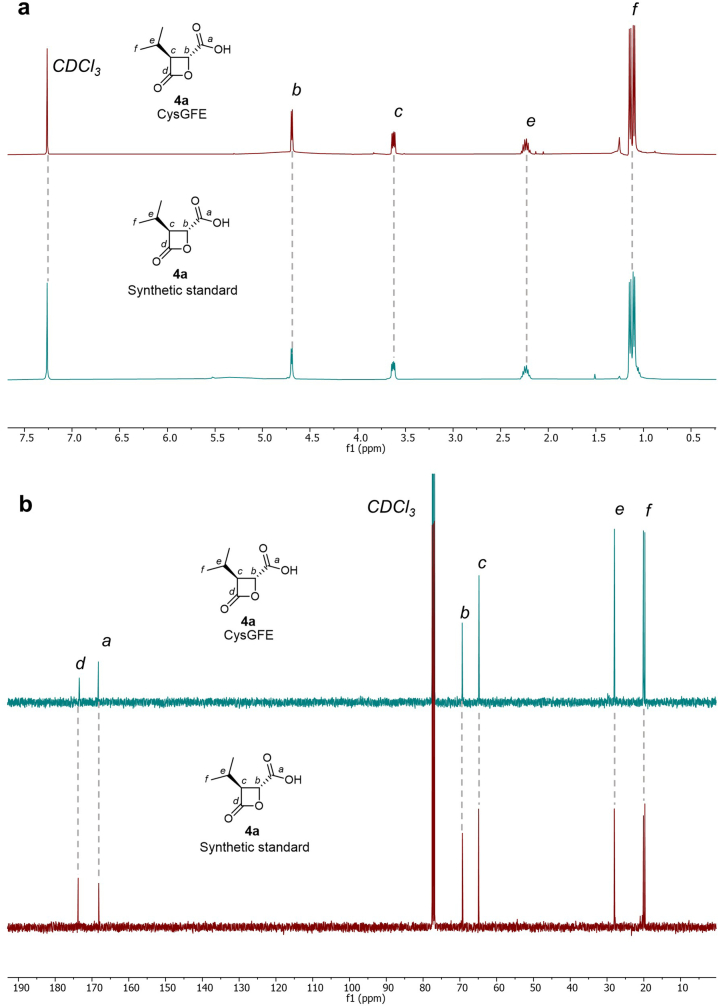
Comparison of the NMR spectra of 4a prepared enzymatically and chemically. **4a** was synthesised enzymatically with CysGFE, **1a**, SAM, and ATP, and chemically using previously reported procedure^37^. **a**, ^1^H-NMR comparison. **b**, ^13^C-NMR comparison.

**Extended Data Fig. 6:**
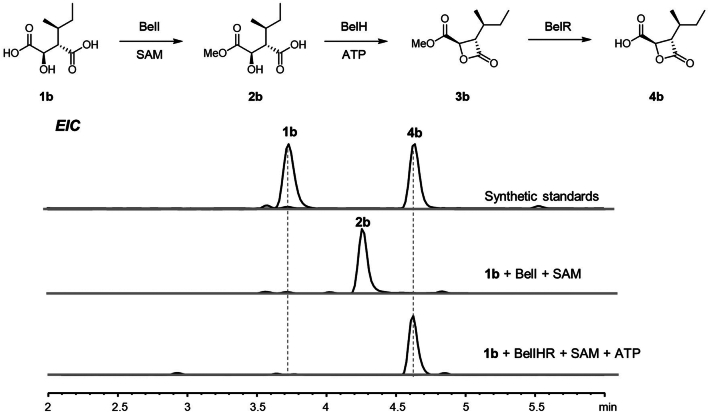
Bel pathway β-lactone formation LC-MS assay. β-lactone warhead assembly in the bel pathway follows the same methylation-lactonisation-hydrolysis sequence as the cys pathway. See Method section (enzyme assay) for experimental details.

Given that the methyl ester of **3a** must be hydrolyzed or transformed directly to an amide or peptide product whilst retaining the labile β-lactone ring structure, we reasoned that **3a** may be the substrate of putative hydrolase CysE. Although overproduction of CysE proved problematic, a small amount of the soluble enzyme was obtained. Incubating **2a** in a tandem reaction with CysF and CysE, including ATP, resulted in the β-lactone carboxylic acid **4a** (Fig. 2a,b). With each step for β-lactone formation confirmed, the cascade reaction of malic acid **1a** with the three enzymes, CysGFE, was carried out on a larger scale, with SAM and ATP, to give **4a** in 94% isolated yield. The NMR spectra of **4a** were identical to a sample of **4a** that was produced by asymmetric synthesis using Evan’s oxazolidinone (Extended Data Fig. 5 and Supplementary Fig. 1)^37^. Assays with the set of enzyme orthologs from the bel pathway (BelIHR) also resulted in the complete conversion of 3-*sec*-butyl malic acid **1b** to give **4b** (Fig. 1c), which was identical to the synthetic **4b** (Supplementary Fig. 1 and Extended Data Fig. 6). The cascade reaction of **1b** with BelIHR, SAM and ATP was also performed on a preparative scale to give **4b** in 87% isolated yield. The fact that three enzymatic steps consuming two high-energy cofactors (SAM and ATP) are required to transform malic acids (**1a** and **1b**) to the β-lactone (**4a** and **4b**) was unexpected. In principle, the ATP-dependent activation of **1a** or **1b** by CysF or BelH may enable direct cyclization to **4a** or **4b** in a single step, rather than three (Figs. 1c and 2a). To explore why nature evolved this cryptic methylation pathway (**1**→**2**→**3**→**4**), we incubated **1a** with CysF and ATP alone. Although the CysF reaction with **1a** did not afford **4a**, the inclusion of hydroxylamine in this assay did lead to the hydroxamic acid derivative **10** in ~50% yield (Figs. 2a and 3a and Supplementary Fig. 5). This indicates that CysF can efficiently activate the C4-carboxyl of **1a** but the resulting acyl-adenylate (**1a**-AMP) is not subsequently cyclized to form the lactone **4a**. Cyclization of **1a**-AMP and the C1-methyl ester **2a**-AMP would involve similar steric constraints (Fig. 3a). However, with **1a**-AMP, the negatively charged C1-carboxylate results in additional unfavorable electrostatic interactions in the sterically hindered and strained transition state, which presumably impedes the direct cyclization to β-lactone **4a** (Fig. 3a). We, therefore, suggest that nature may have evolved this unusual cryptic methylation sequence, at the expense of SAM and ATP, to reduce electrostatic repulsion and stabilize the strained transition state for the sterically demanding β-lactonization reaction to proceed.

**Extended Data Fig. 7:**
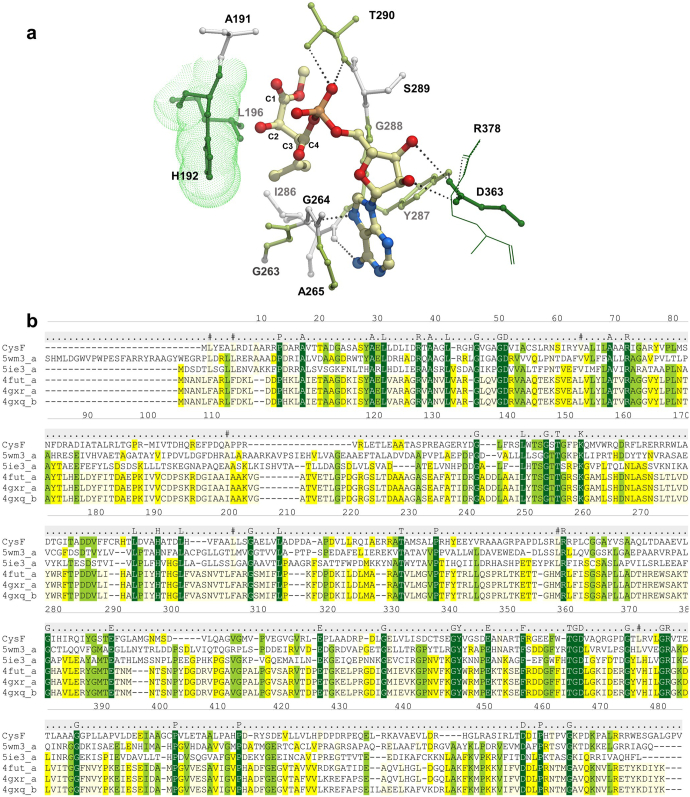
Docking and sequence alignment of CysF. **a**, *In silico* docking of **2a**-AMP in the crystal structural of CysF. **b**, sequence alignment of CysF with other adenylate-forming enzymes. Pairwise levels of sequence identities between CysF and the related adenylate forming enzymes (5IE3, 4GXR, 5WM3, 4GXQ & 4FUT)^39–43^ are between 18 – 30% and the key residues involved in the binding of AMP are highly conserved (T290, D363, R378). The docked conformation of **2a**-AMP suggests H192 may perform the role of a general base during β-lactone formation. The close proximity of the C1-methyl ester to the phosphoryl group, of the docked **2a**-AMP, further underscores the potential for electrostatic repulsion if the C1-carboxylate group was not methylated. Polyethylene glycol (PEG4) is observed to occupy a region of the putative active site in the crystal structure of CysF, which is likely to be an artifact of the crystallisation process. Additional electron density is present in the crystal structure which is consistent with carboxylation resulting in an N-terminal carbamate stabilised by interactions to R131. Carboxylation is suggested to be a common, spontaneous and reversible post translation modification^65^. Given the CysF N-terminus is not located near the active site, it is unlikely that this modification has any effect on the catalytic mechanism of the enzyme.

**Extended Data Fig. 8:**
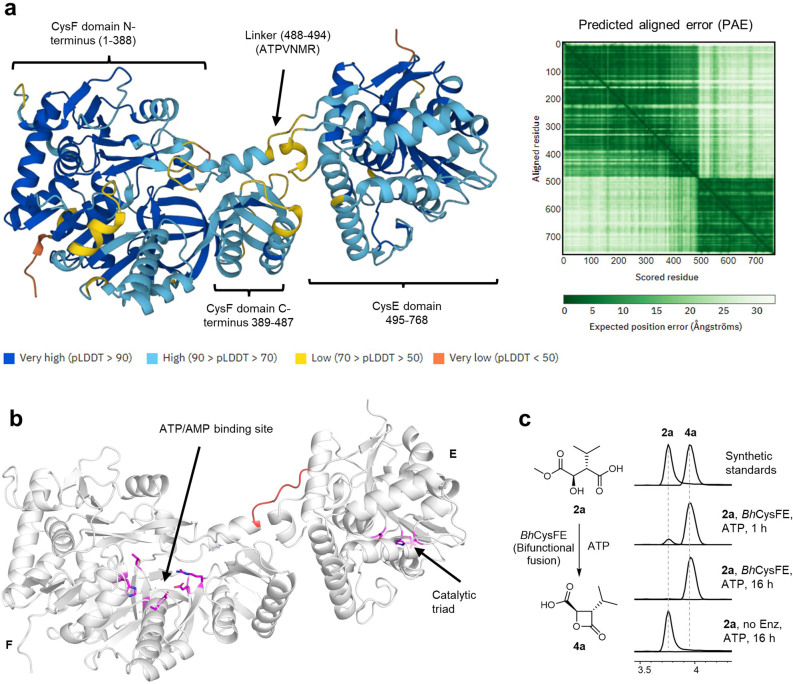
AlphaFold structural model and activity assay of bifunctional enzyme *Bh*CysFE. **a**, Structural prediction by AlphaFold, AF-A0A562R406-F1-model_v4 (https://www.alphafold.ebi.ac.uk/entry/A0A562R406), revealed two domains similar to CysF (aa. 1-487) and CysE (aa. 495-768), connected by a short linker (sequence ATPVNMR) colour coded showing regions with lower (orange) to higher accuracy (blue). **b**, The position of the predicted active site residues of the F domain are highlighted in magenta based on sequence alignment and docking as described in Extended Data Fig. 7. The predicted catalytic triad present in the E domain Glu607, His739 and Ser582, which is highly conserved in hydrolase enzymes is also highlighted in magenta. **c**, Assay of the bifunctional fusion *Bh*CysFE with **2a** results in direct formation of **4a**.

We determined a 1.9-Å-resolution X-ray crystal structure of CysF, which revealed a large N-terminal domain with a smaller C-terminal domain (Fig. 3b), similar to structures of the related acyl-CoA and amide-bond synthetases in the open adenylation state^39–43^. As with the related adenylate-forming enzymes^39–43^, it is likely that the C-terminal domain undergoes a rotation to a closed state, which may facilitate β-lactonization of the intermediate **2a**-AMP (Fig. 3a,b). In silico docking (ICM Molsoft) was performed with CysF to investigate the potential binding mode of the intermediate **2a**-AMP (Extended Data Fig. 7). The docked conformation of **2a**-AMP was consistent with the acyl-AMP-binding or ATP-binding modes observed in related enzymes^39–43^ and showed that the C2-OH of **2a**-AMP was in close proximity to H192, which may act as a general base facilitating β-lactone formation. Bioinformatics analysis of available bacterial genome sequences also revealed several BGCs similar to the cys BGC, except where CysF and CysE are produced as a single fusion protein with a linker sequence between the two (Fig. 3c, Extended Data Fig. 8 and Supplementary Fig. 6). One of the fusions (BhCysFE) was overproduced in *E*. *coli* (Supplementary Fig. 2 and Supplementary Table 3), purified and then incubated with **2a** and ATP, leading to a quantitative production of **4a** (Extended Data Fig. 8). The β-lactone ring of methyl ester **3a** is more labile than corresponding acid **4a**, which may be because of the additional electrostatic repulsion provided by the charged carboxylate group of **4a** hindering attack by water or other nucleophiles (Supplementary fig. 7). CysFE may, therefore, have evolved as a bifunctional fusion protein to facilitate channeling of the more unstable β-lactone **3a** between the two catalytic active sites (Fig. 3c and Extended Data Fig. 8). Various channeling mechanisms are proposed to occur when different enzyme active sites are in close proximity and these mechanisms serve to sequester unstable intermediates, limiting diffusion and subsequent degradation or toxic effects^44–46^. Further studies would be required to explore whether channeling operates between the two active sites of CysFE.

### Characterization of the cys and bel peptide assembly enzymes

**Fig. 4:**
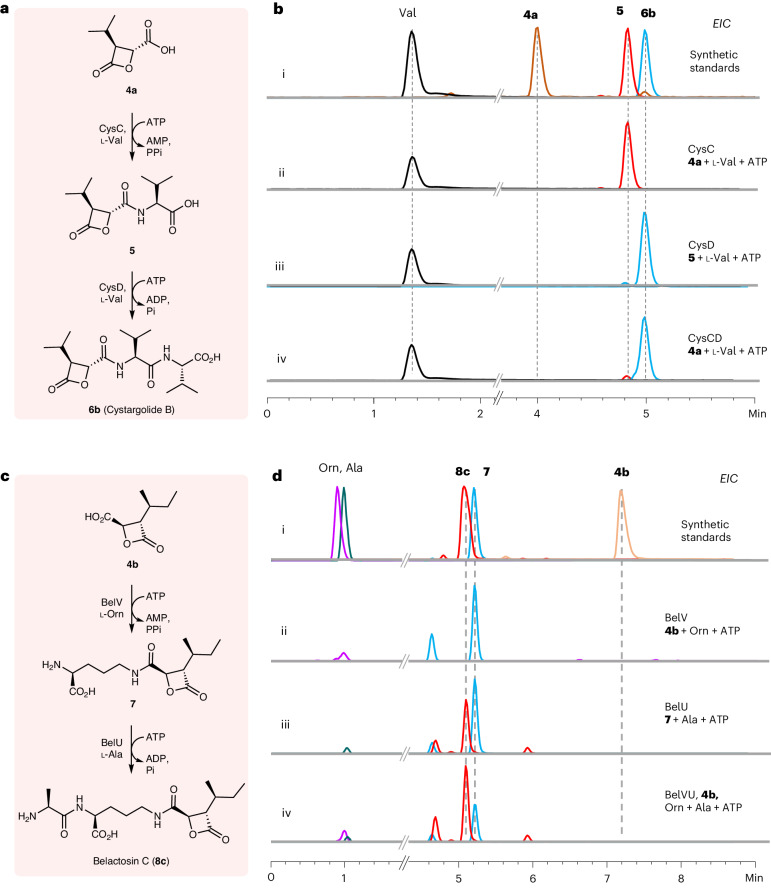
In vitro characterization of the peptide assembly enzymes CysCD and BelVU. **a**, The confirmed pathway for cys peptide assembly. **b**, LC–MS analysis for the reaction catalyzed by the CysCD enzymes. **c**, The confirmed pathway for bel peptide assembly. **d**, LC–MS analysis for the reaction catalyzed by the BelVU enzymes.

Having established the pathway to the β-lactone warheads, we next sought to elucidate the remaining steps in the biosynthesis of cys and bel. Previously, it was proposed that a putative ATP-grasp enzyme CysD ligates l-valine to generate the dipeptide l-valine-l-valine, which is then coupled with the lactone acid **4a**, by CysF^32^. We incubated CysD with l-valine and ATP, but this failed to produce l-valine-l-valine. Similarly, CysF did not catalyze ligation of **4a** with l-valine or the dipeptide (l-valine-l-valine), which was unsurprising after we showed that CysF catalyzes β-lactonization. Next, we tested the other putative ATP-dependent enzymes in the cys BGC (CysC and CysD) to establish whether they catalyzed the ligation of β-lactone **4a** and l-valine. Whilst CysD failed to generate any products, the reaction of CysC with **4a,** l-valine and ATP gave **5** in excellent yields (Fig. 4a,b). CysC exhibits sequence similarity to other adenylate-forming enzymes, including amide-bond synthetases such as CfaL, which also *N*-acylates hydrophobic amino acids^43^. By a process of elimination, we confirmed that the remaining ligase, CysD, catalyzes coupling of **5** with l-valine to complete the biosynthesis of cys (**6**) (Fig. 4a,b). Although other ATP-grasp ligases, such as CysD, are known to couple two amino acids to generate dipeptides^47^, a pathway to cys through l-valine-l-valine^32^ is unlikely to have evolved in nature, given that this dipeptide would be particularly susceptible to proteolysis. The route that nature has selected (Fig. 4a) proceeds through a more stable intermediate (**5**) and, thus, conserves ATP. In the predicted bel pathway, it was suggested that the ATP-grasp enzyme BelU couples l-alanine with l-ornithine to generate a dipeptide (l-alanine-l-ornithine) that is then acylated with β-lactone **4b** by BelH to give **8c** (ref. ^32^). We tested both enzymes but neither possessed the proposed activity. Instead, we found that BelV, which is most similar to CysC, ligates **4b** with the side chain amino group l-ornithine to produce **7**. BelU then activates l-alanine for ligation with **7** to complete the biosynthesis of bel (Fig. 4c,d). We also found that potassium ions are required for the activity of BelU, which has been observed for other ATP-grasp enzymes^48^.

### Enzymatic cascades to cys and bel analogs

**Fig. 5:**
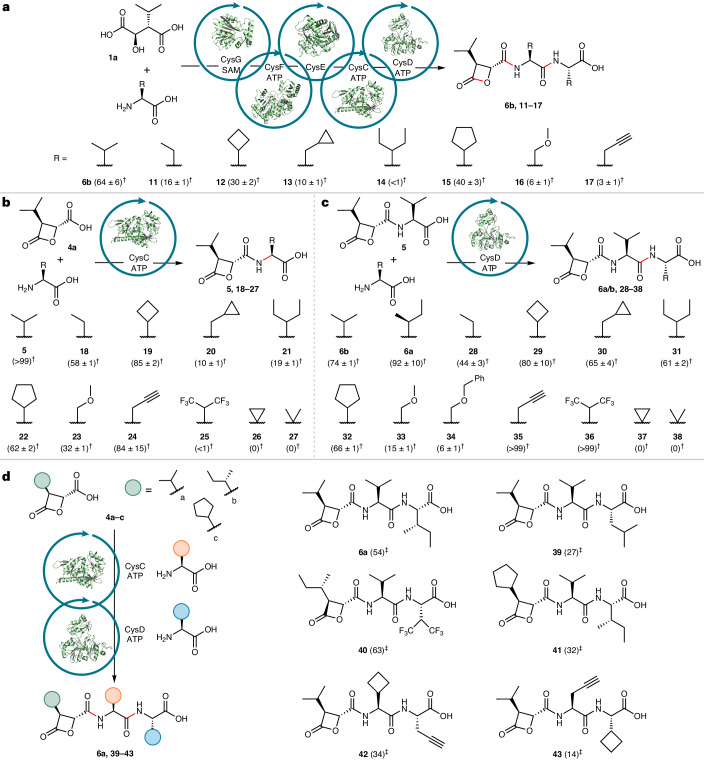
Synthetic scope of CysGFECD and enzymatic cascades to cys derivatives. **a**, Total in vitro biosynthesis of cys B (**6b**) and analogs using the entire set of enzymes in the cys pathway. Assays were carried out with 20 µM of each enzyme (CysG, BhCysFE, CysC and CysD), SAM (3 mM), ATP (9 mM), **1a** (2 mM) and amino acids (6 mM), with incubation for 6 h at 25 °C. **b**,**c**, Assays to determine the amino acid scope of CysC and CysD were carried out with 20 µM CysC or CysD, ATP (3 mM), **1a** or **5** (2 mM) and amino acids (3 mM), with incubation for 5 h at 25 °C. **d**, Preparative-scale CysCD enzymatic cascade reactions were carried out at 25 °C with **4a**–**4c** (0.08 mmol, 4.0 mM), the first amino acid (4.0 mM), ATP (4.4 mM) and CysC (20 µM) with incubation for 12 h, before the second amino acid (5 mM), ATP (4.4 mM) and CysD (20 µM) were added for a further 12 h before quenching. ^†^Percentage conversions were determined in triplicate by LC–MS using calibration curves with synthetic standards. ^‡^Isolated yields were obtained after product isolation and purification (see Methods and the Supplementary Information for full experimental details and Extended Data Fig. 10 for similar bel cascade reactions).

**Extended Data Fig. 9:**
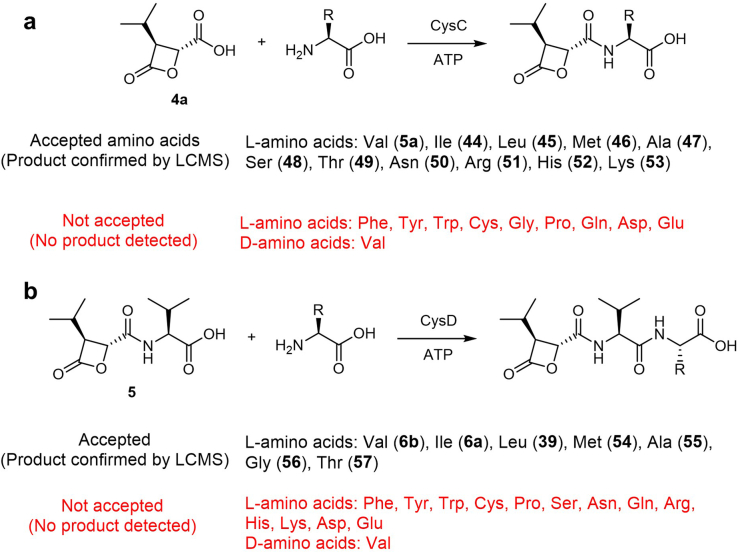
Proteinogenic amino acids scope of ligase CysC and CysD. **a**, Ligation of natural donor carboxylic acid **4a** with acceptor proteinogenic amino acids catalysed by CysC. **b**, Ligation of natural donor carboxylic acid **5** with acceptor proteinogenic amino acids catalysed by CysD. Reactions were carried out on analytical scale (see method section enzyme assays). Tandem mass (MS/MS) data of products (in bracket) are shown in supplementary methods (LC-MS/MS data).

After elucidating the pathways to cys and bel, we set out to explore the substrate scope of the pathway enzymes and develop cascade reactions to cys and bel derivatives, including non-natural precursors similar to those used in the synthesis of promising analogs^26,29,30^. We first reconstituted both pathways in vitro. Using the entire set of enzymes, CysGFECD or BelIHRVU, total in vitro biosynthesis of cys and bel was achieved in one-pot cascade reactions from commercially available malic acid derivatives **1a** or **1b** and amino acid substrates (l-valine or l-ornithine and l-alanine) with SAM and ATP (Fig. 5a and Supplementary Fig. 8). The enzymatic synthesis of cys (**6b**) in 64% yield, in a single unoptimized reaction from commercially available starting material, compares favorably with the chemical syntheses of cys and bel, which typically require 9–11 steps^26–30,37^, with an average overall yield of 10% for cys^37^. Next, we tested the amino acid scope of the peptide assembly enzymes CysC and CysD. Whilst both enzymes had preference for hydrophobic amino acids, as expected, they exhibited considerable flexibility, accepting a wide range of non-natural amino acids commonly found in peptide therapeutics (Fig. 5b,c). CysC also accepted 11 of the proteinogenic amino acids as substrates (Extended Data Fig. 9). *O*-Benzyl-l-serine is also a CysD substrate, producing the analog **34** in low yield. An ester derivative of **34** was previously shown to be a promising lead compound and is a notably more potent proteasome inhibitor in vitro than the parent cys or the widely used anticancer agent carfilzomib^29^. Given that promising cys analogs, such as **34**, require laborious syntheses^29^, we sought to exploit the promiscuity of CysC and CysD to develop cleaner, more efficient enzymatic cascade reactions to other analogs. First, we demonstrated that the five-enzyme, one-pot CysGFECD cascade could transform simple malic acid **1a** to a range of cys analogs (**11**–**17**) in one step, with l-valine replaced by alternative nonproteinogenic amino acids (Fig. 5a). We then developed a chemoenzymatic approach to assemble cys analogs with control over the amino acid sequence, including both native and non-natural warheads (Fig. 5d). In these examples, a synthetic β-lactone warhead (**4a**–**4c**) is incubated with CysC along with the first amino acid (1.0 eq.) and ATP. After 12 h, the ligase CysD and a second, different amino acid (1.25 eq) are added to the reaction. By simply changing the order of addition of amino acids, without isolating intermediates, we can control the final peptide sequence, delivering cys A (**6a**) and various analogs (**39**–**43**) on a preparative scale in sufficient yields without any process optimization. Although the cys analogs prepared in this work possess C-terminal carboxylic acids, they could be easily transformed to C-terminal amides or esters, which have been shown to be more potent proteasome inhibitors.

**Extended Data Fig. 10:**
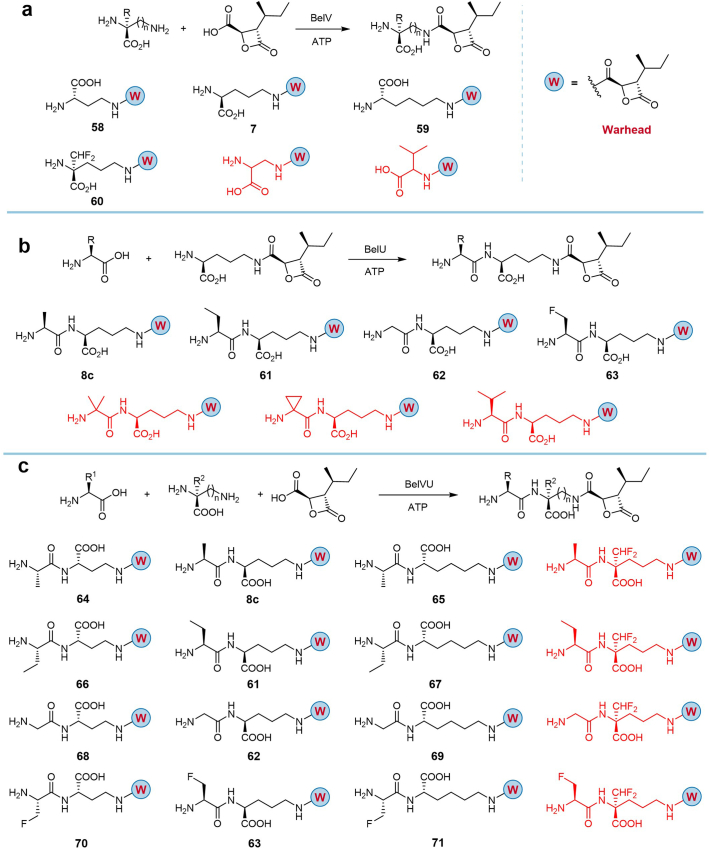
Amino acid scope tested for the bel pathway ligases. **a**, Amino acid substrates tested for BelV reaction. **b**, Amino acid substrates tested for BelU reaction. **c**, Substrate scope tested for BelVU cascade reaction. The reaction was analysed by LC-MS. Black colour compounds have anticipated product mass detected, while the red colour indicated no product mass was detected. For assay conditions see Methods section (enzyme assays). Tandem mass (MS/MS) data of products are shown in supplementary methods: LC-MS/MS data.

Similar cascade reactions were also used to produce bel analogs with l-lysine or l-2,4-diaminobutyrate replacing l-ornithine and various alternative amino acids in place of l-alanine (Extended Data Fig. 10). Unlike CysCD, the BelVU ligases had substantially different (orthogonal) amino acid selectivity. Consequently, BelVU cascade reactions could be performed with both enzymes and different combinations of amino acids added concomitantly. The substantial number of β-lactone peptides that we generated enzymatically (over 40 peptides; Fig. 5 and Extended Data Figs. 9 and 10) is not exhaustive or optimized and we envisage that many more analogs could be accessible using just the native cys and bel enzymes. It should also be possible, by exploiting established engineering approaches^18^, to evolve the cys and bel enzymes to increase their substrate scope, thereby creating more analogs, or to enhance enzyme activity and selectivity for the synthesis of specific target (lead) compounds for future drug discovery.

## Discussion

We deciphered the cryptic enzymatic logic that underpins the biosynthesis of therapeutically relevant β-lactone-containing peptide natural products, revealing several surprising features, including carboxyl methylation for transient protection facilitating β-lactone assembly. Other pathways to β-lactone-containing natural products have been investigated^19,21,49,50^ but none of these rely on cryptic carboxyl methylation. Aside from a few volatile methyl esters known to be produced by SAM-dependent carboxyl methyltransferases in plants^51^, methyl esters are scarce in nature because of their hydrolytic instability, particularly in cells where hydrolase enzymes are abundant. There are rare examples where methyl esters have been shown to exist transiently in the biosynthetic pathways to capuramycin and biotin^38,52^. However, we are unaware of any examples where nature has used cryptic methylation to facilitate energetically demanding enzymatic reactions, such as β-lactonization. Our observation that CysFE exists as a bifunctional fusion is also noteworthy, suggesting that these enzymes collaborate closely to transfer reactive β-lactone intermediates between active sites. In addition to well-characterized examples of physical tunneling to facilitate the transfer of intermediates between active sites, other proximity channeling mechanisms have been proposed^44–46^. Although channeling is thought to be prevalent in primary metabolism^44–46^, there are only a few examples where it is suggested to occur in secondary metabolism^53^. It will be interesting to further explore the structure and properties of CysFE to establish whether the channeling phenomenon occurs in this case.

The pathways to cys and bel are also unusual as the reactive warhead is synthesized first, followed by peptide assembly catalyzed by standalone ligases. Other peptide natural products armed with warheads (for example, epoxyketones and aldehydes) use nonribosomal peptide synthetase (NRPS) enzymes to first assemble the peptide on carrier proteins before addition of the unstable warhead^54–57^. The unusual ligase assembly pathways described here offer notable advantages over NRPS systems for the production of analogs and future engineering. Using native pathway enzymes, we showed how a large and diverse range of cys and bel analogs can be produced by in vitro biosynthesis through scalable enzymatic cascade reactions. NRPS enzymes, on the other hand, are large and complex multimodular enzyme systems that are more difficult to use for synthesis in vitro. Misfolding can cause problems with the expression of active soluble NRPS and only analytical-scale quantities of NRPS products are typically produced in vitro^58,59^. Although major advances in NRPS engineering have been made in recent years, the diversity of new products and yields obtained remain low^58–60^. The use of standalone ligases, including ligases from different pathways and improved engineered variants^18,43^, could offer greater flexibility and scope for producing a wider range of bioactive peptides for future drug development. Our approach for delivering analogs in one-pot scalable cascade reactions from available malic acid or β-lactone precursors is also much cleaner and more efficient than the multistep chemical syntheses currently used to manufacture peptide drugs of this type.

## Methods

### General information

Chemicals were purchased from Sigma-Aldrich, Fluorochem, Acros Organics, Fisher Scientific UK, Bachem, Alfa Aesar or Apollo Scientific and used without further purification unless otherwise stated. Molecular biology enzymes were purchased from New England Biolabs unless otherwise stated. Codon-optimized genes were synthesized by Twist Bioscience. Proteins were analyzed by SDS–PAGE on precast gels (Invitrogen Novex WedgeWell 8–16% Tris-glycine gel). Low-resolution LC–MS was performed on an Agilent 1260 LC system fitted with an Agilent 6130 Quadrupole MS. High-resolution LC–MS and tandem LC–MS/MS were recorded on an Agilent 6560 quadrupole time-of-flight (Q-TOF) + Agilent 1290 Infinity LC system. GC–MS was performed on an Agilent GC 7890B coupled with 5975 Series mass selective detector. NMR spectra were recorded on Bruker Avance III spectrometers (400 MHz, 500 MHz or 800 MHz) with TopSpin and IconNMR. Deuterated solvents used included CDCl_3_, CD_3_OD, DMSO-d_6_, D_2_O and D_2_O with 0.1% DCl. NMR data were processed using MestReNova version 11 software.

### Cloning

Synthetic genes for CysF, BelH, CysD and BelU were initially cloned into the pET28-a(+) plasmid (using NdeI and XhoI sites) with an N-terminal histidine tag sequence. However, N-terminal histidine-tagged CysF produced insoluble protein. N-terminal histidine-tagged CysD and BelU produced soluble expression but with no activity. CysF, CysD and BelU were, therefore, cloned into the pET21-a(+) plasmid (using NdeI and XhoI sites) to insert a C-terminal histidine tag (Supplementary Tables 1 and 2). Synthetic genes for CysG, CysE, CysC, BelI, BelR, BelV and BhCysFE were inserted into the pET28-a(+) plasmid (using NcoI and XhoI sites) carrying C-terminal histidine tag sequences (Supplementary Tables 1 and 2).

### Protein expression

Luria–Bertani medium (10 ml) with 50 μg ml^−1^ kanamycin (pET28-a(+)) or 100 μg ml^−1^ ampicillin (pET21-a(+)) was inoculated with *E*. *coli* BL21(DE3) containing a plasmid and incubated for 18 h at 37 °C with shaking (180 r.p.m.). The resulting seed culture (8 ml) was then used to inoculate 800 ml of autoinduction 2YT broth (with trace elements from Formedium) containing 50 μg ml^−1^ kanamycin (pET28a-(+)) or 100 μg ml^−1^ ampicillin (pET21-a(+)). After incubation for 4 h at 37 °C with shaking (180 r.p.m.), the temperature was reduced to 20 °C and incubation was continued for a further 18 h (180 r.p.m.). The cultures were then harvested by centrifugation (3,000*g*, 4 °C, 10 min) and cells were resuspended in 80 ml of PBS, transferred into two 50-ml falcon tubes, pelleted by centrifugation (3,000*g*, 4 °C, 15 min) and then frozen until further usage. CysE (with C-terminal histidine tag) produced mainly insoluble protein. A small amount of soluble CysE was, however, obtained (~5 mg of protein from 1 L of cell culture) after the standard protein purification method described below. For the three fused bifunctional CysFE enzymes tested, only BhCysFE was produced in soluble form. MsCysFE afforded a very low level of expression, whilst MpCysFE showed good expression but formed inclusion bodies.

### Protein purification

Frozen cell pellets were thawed and resuspended in 40 ml of lysis buffer (50 mM NaH_2_PO_4_, 300 mM NaCl, 10 mM imidazole and 10% glycerol; pH 7.8) and lysed by sonication (10 min, 50% pulse, 60% amplitude). The lysate was then cleared by centrifugation (23,224*g*, 40 min, 4 °C), 1 ml of pre-equilibrated Ni-NTA resin was added and the tube was incubated at 4 °C for 30 min. The lysate with resin was loaded onto a gravity-flow column (Bio-Rad) and flowthrough was collected. The resin was washed with 10 ml of lysis buffer, followed by two washes with 10 ml of wash buffer (50 mM NaH_2_PO_4_, 300 mM NaCl, 40 mM imidazole and 10% glycerol; pH 7.8), and eluted with 10 ml of elution buffer (50 mM NaH_2_PO_4_, 300 mM NaCl, 100 mM imidazole and 10% glycerol; pH 7.8). The eluent from the column was monitored by Bio-Rad protein assay and elution continued until no further protein could be seen eluting. Samples of wash and elution fractions were analyzed by SDS–PAGE. The wash and elution fractions that contained the protein of interested (with good purity) were combined and concentrated down to 2.5 ml using a centrifugal concentrator (Sartorius, Vivaspin 20; mol. wt. cutoff, 30 kDa or 10 kDa depending on the size of the protein of interest). The 2.5-ml solution was applied to an equilibrated PD-10 desalting column (GE Healthcare, performed according to the manufacturer’s instructions). The column was eluted with 3.5 ml of protein storage buffer (20 mM NaH_2_PO_4_, 300 mM NaCl and 10% glycerol; pH 7.8). If necessary, the resulting eluent was further concentrated to approximately 10 mg ml^−1^ or more. Protein was aliquoted into single-use tubes, flash-frozen using liquid nitrogen and stored at −80 °C.

### Further purification for protein crystallization

For the protein crystallization, the plasmid encoding the *CysF* gene was used to transform NiCo21(DE3) competent *E*. *coli* cells. Following transformation, glycerol stocks were prepared from the overnight cultures of a single colony (sequencing confirmed). The enzyme was expressed and first purified using Ni-NTA column according to the general procedure described above. The wash and elution protein fractions with good purity (judged by SDS–PAGE) were combined and concentrated using a Vivaspin centrifugal concentrator (mol. wt. cutoff, 30 kDa) up to 20 mg ml^−1^. For further purification, approximately 0.6 ml of this concentrated protein solution was injected into a 2-ml loop attached to the ӒKTA pure protein purification system with GE Healthcare UNICORN software (version 7.3). The protein was passed down a Superdex 200 Increase 10/300 GL gel filtration column that was pre-equilibrated with gel filtration buffer (50 mM Tris-HCl, 300 mM NaCl, 10 mM MgCl_2_ and 10% glycerol; pH 7.5). CysF was eluted with gel filtration buffer and buffer-exchanged to crystallization buffer (50 mM Tris-HCl, 100 mM NaCl, 10 mM MgCl_2_ and 5% glycerol; pH 7.5) using a PD-10 desalting column (pre-equilibrated with crystallization buffer). The resulting fraction was concentrated to 8–10 mg ml^−1^ using an Amicon Ultra 0.5-ml concentrator (mol. wt. cutoff, 30 kDa).

### Crystallogenesis

Single crystals of CysF were prepared by mixing 200 nl of 8 mg ml^−1^ protein in crystallization buffer with equal volumes of precipitant. All trials were conducted by sitting-drop vapor diffusion and incubated at 4 °C. Crystals of the apo protein were formed in 0.1 M sodium citrate pH 5.5, 20% w/v polyethylene glycol (PEG) 3000 (JCSG + Eco A2, Molecular Dimensions). Individual crystals were cryoprotected in mother liquor supplemented with 25% PEG 200 before flash-cooling in liquid nitrogen. Data were collected from single crystals at the Diamond Light Source (MX31850-i04) and subsequently scaled and reduced with Xia2. Preliminary phasing was performed by molecular replacement in Phaser using a search model generated in AlphaFold^61,62^. Iterative cycles of rebuilding and refinement were performed in Coot and Phenix.refine, respectively. Structure validation with MolProbity and PDBREDO was integrated into the iterative rebuild and refinement process. The resolution cut of the data was determined by paired refinement as implemented in PDBREDO. Complete data collection and refinement statistics can be found in Supplementary Table 4. Coordinates and structure factors were deposited in the Protein Data Bank (PDB) under accession code 8RA0.

### Bioinformatic analysis of CysF and discovery of fused CysFE

Bioinformatic analysis was conducted on the cys and bel pathway enzymes using the Enzyme Function Initiative tools^63^. The analyses used databases UniProt 2022-04, InterPro 91 and corresponding information from the European Nucleotide Archive. The identification of the fused CysFE configuration began with a basic local alignment search tool query using UniProt A0A1W6R555 for CysF to generate a sequence similarity network (SSN) with default settings. An alignment score threshold of 10^6^ was chosen to identify a subnetwork containing both CysF and BelH as they are isofunctional in their enzymatic capabilities. The obtained SSN was further analyzed with the Genome Neighborhood Tool, leading to the obtention of genome neighborhood diagrams (Supplementary Fig. 6). Manual inspection of these diagrams revealed three genomes exhibiting a CysF and CysE fusion, indicating a bifunctional enzyme. The structure of BhCysFE was modeled using AlphaFold (AF-A0A562R406-F1-model_v4, https://www.alphafold.ebi.ac.uk/entry/A0A562R406)^61,62^.

### Modeling of CysF closed conformation

The crystal structure of CysF was used to search for homologs through Foldseek and Dali. Identified homologs were observed that displayed structures in both open and closed conformations. Crystal structures of 4-coumarate CoA ligase in both open and closed conformations (PDB 5BSM, open; PDB 5BSR, closed) were used as templates upon which to model a closed state of CysF. The 4-coumarate CoA ligase structure revealed that domain closure is achieved through a hinge-like rigid-body reorganization of the two domains. A closed model of CysF was, therefore, modeled by superposition of the N-terminal domain of CysF with the corresponding N-terminal domain of the template (root-mean-square deviation (r.m.s.d.) of 1.86 Å after secondary-structure alignment). Subsequently, the C-terminal domain of CysF was superimposed with the C-terminal domain of the template in the closed conformation (r.m.s.d. of 3.03 Å after secondary-structure alignment) followed by an energy minimization (Yasara).

### CysF docking studies

A model of **2a**-AMP was constructed and docked into the crystal structure of CysF. The pocket for docking was identified using ICM Pocket Finder as implemented in ICM-Pro^64^. The top hit from the docking as ranked by the RTCNN (radial convolution neural network with topological convolutions) score (−29.78) is presented in Extended Data Fig. 7. The docked pose was compared to structures of similar enzymes (PDB 5WM3, 5IE3, 4FUT, 4GXR and 4GXQ). A multiple-sequence alignment of these enzymes is presented in Extended Data Fig. 7.

### Chromatography

LC–MS analysis was performed with a Kinetex XB-C18 Core Shell column (100 mm × 4.6 mm, 5 µm, Phenomenex), using a flow rate of 1.0 ml min^−1^, column oven temperature of 40 °C, mobile phase A of water with 0.1 (v/v) formic acid and mobile phase B of acetonitrile with 0.1% (v/v) formic acid. The mobile phase program for method 1 was as follows: 0–6 min, 5–95% B; 6–7 min, 95% B; 7–7.5 min, 95–5% B; 7.5–11 min, 5% B. The mobile phase program for method 2 was as follows: 0–15 min, 5–95% B; 15–16 min, 95% B; 16–16.5 min, 95–5% B; 16.5–20 min, 5% B.

GC–MS analysis was performed with a VF-5ht column (30 m × 0.25 mm, 0.1 µm, J&W), using a flow rate of 1 ml min^−1^, inlet temperature of 240 °C, split ratio of 100:1, inlet pressure of 7.6 psi and helium as the carrier gas. The oven temperature program was an initial temperature of 50 °C, held for 2 min, followed by a temperature ramp at 30 °C min^−1^ to 350 °C, held for 3 min.

### Enzyme assays for testing the proposed pathway^32^

CysC was initially assayed for the lactonization of **1a** to **4a** (Fig. 2a) in a reaction mixture (100 µl) containing 2 mM **1a**, 5 mM ATP, with or without 5 mM CoASH, 10 mM MgCl_2_ and 20 µM CysC in 100 mM potassium phosphate buffer (pH 7.8) incubated at 25 °C for 12 h. The reaction was quenched by addition of 100 µl of acetonitrile and the precipitated protein was removed by centrifugation. The supernatant was subjected to LC–MS analysis (method 1).

CysGE and BelIR were tested for methylation and lactonization activity (**1a** to **4a**, Fig. 2a) in reactions (100 µl) containing 2 mM **1a**, 5 mM SAM, 10 mM MgCl_2_, 20 µM CysG or BelI and 20 µM CysE or BelR in 100 mM potassium phosphate buffer (pH 7.8) incubated at 25 °C for 12 h. The reactions were quenched and analyzed by LC–MS analysis (as above).

The proposed amide-bond-forming activities of CysF and BelH (Fig. 4a,d) were tested in reactions (100 µl) containing 2 mM **4a** (CysF) or **4b** (BelH), 5 mM l-valine-l-valine (CysF) or l-alanine-l-ornithine (**12**, BelH), 5 mM ATP, 10 mM MgCl_2_ and 20 µM CysF or BelH in 100 mM potassium phosphate buffer (pH 7.8, with 300 mM NaCl and 10% v/v glycerol) incubated at 25 °C for 12 h. The reactions were quenched and subjected to LC–MS analysis (as above).

The proposed amide-bond-forming activities of CysD and BelU (Fig. 4a,d) were also tested in reactions (100 µl) with 2 mM l-valine (for CysD) or 2 mM l-ornithine and 2 mM l-alanine (for BelU), 5 mM ATP, 10 mM MgCl_2_ and 20 µM CysD or BelU in 100 mM potassium phosphate buffer (pH 7.8) incubated at 25 °C for 12 h. The reactions were quenched and analyzed (as above).

### Enzyme assays for the new pathways (Figs. 2 and 4)

The CysG and BelI assay mixtures (100 µL) containing 2 mM **1a** or **1b**, 5 mM SAM, 10 mM MgCl_2_, and 20 µM CysG or BelI in 100 mM potassium phosphate buffer (pH 7.8) were incubated at 25 °C for 12 h. The reactions were quenched by addition of 100 µL acetonitrile and the precipitated protein was removed by centrifugation. The supernatant was subjected to LC–MS analysis (method 1).

The CysGF and BelIH cascade reactions (100 µl) containing 2 mM **1a** or **1b**, 5 mM SAM, 5 mM ATP, 10 mM MgCl_2_, 20 µM CysG or BelI and 20 µM CysF or BelH in 100 mM HEPES or potassium phosphate buffer (pH 7.8) were incubated at 25 °C for 12 h. The reaction was quenched by addition of 100 µl of acetonitrile and the precipitated protein was removed by centrifugation. The supernatant was subjected to LC–MS analysis (method 1). For GC–MS analysis, the reaction was scaled up to 200 µl and the reaction time was 5 h. The reaction was quenched by extracting with ethyl acetate (200 µl). The organic layer was dried over MgSO_4_ and analyzed by GC–MS.

The CysFE and BelHR cascade reactions (100 µl) containing 2 mM **2a** or **2b**, 5 mM ATP, 10 mM MgCl_2_, 20 µM CysF or BelH and 20 µM CysE or BelR in 100 mM potassium phosphate buffer (pH 7.8) were incubated at 25 °C for 12 h. The reaction was quenched by addition of 100 µl of acetonitrile and the precipitated protein was removed by centrifugation. The supernatant was subjected to LC–MS analysis (method 1).

The CysGFE and BelIHR cascade reactions (100 µl) containing 2 mM **1a** or **1b**, 5 mM SAM, 5 mM ATP, 10 mM MgCl_2_, 20 µM CysG or BelI, 20 µM CysF or BelH and 20 µM CysE or BelR in 100 mM potassium phosphate buffer (pH 7.8) were incubated at 25 °C for 12 h. The reaction was quenched by addition of 100 µl of acetonitrile and the precipitated protein was removed by centrifugation. The supernatant was subjected to LC–MS analysis (method 1).

The CysC and BelV assays (100 µl) containing 2 mM **4a** or **4b**, 4 mM l-valine or l-ornithine, 5 mM ATP, 10 mM MgCl_2_ and 20 µM CysC or BelV in 100 mM potassium phosphate buffer (pH 7.8) were incubated at 25 °C for 2 h. The reaction was quenched and analyzed (see directly above).

The CysD and BelU assays (100 µl) containing 2 mM **5** or **7**, 4 mM l-valine or l-alanine, 5 mM ATP, 10 mM MgCl_2_ and 20 µM CysD or BelU in 100 mM potassium phosphate buffer (pH 7.8) were incubated at 25 °C for 2 h. The reaction was quenched (as above) and subjected to LC–MS analysis (method 1 for CysD and method 2 for BelU reactions). We noted that potassium ions served as an activator for CysD catalysis and were essential for the activity of BelU.

The CysCD and BelVU cascade reactions (100 µl) containing 2 mM **4a** or **4b**, 6 mM l-valine (CysCD) or 4 mM l-ornithine with 4 mM l-alanine (BelVU), 10 mM ATP, 10 mM MgCl_2_, 20 µM CysC or BelV and 20 µM CysD or BelU in 100 mM potassium phosphate buffer (pH 7.8) at 25 °C for 12 h. The reaction was quenched (as above) and subjected to LC–MS analysis (method 1 for CysCD and method 2 for BelVU reactions).

### Substrate scope analysis (Fig. 5)

The CysGFECD enzyme cascade reactions (100 µl) containing 2 mM **1a**, 3 mM SAM, 9 mM ATP, 6 mM l-valine (or other amino acids), 10 mM MgCl_2_, 20 µM CysG, 20 µM BhCysFE, 20 µM CysC and 20 µM CysD in 100 mM potassium phosphate buffer (pH 7.8) were incubated at 25 °C for 6 h. The reaction was quenched by addition of 100 µl of acetonitrile and the precipitated protein was removed by centrifugation. The supernatant was subjected to LC–MS analysis (method 1). Reaction yields (average of three replicates) were determined from calibration curves using synthetic product standards.

The BelIHRVU enzyme cascade reactions (100 µl) containing 2 mM **1b**, 3 mM SAM, 9 mM ATP, 5 mM l-ornithine (or other amino acids), 5 mM l-alanine (or other amino acids), 10 mM MgCl_2_, 15 µM BelI, 15 µM BelH, 15 µM BelR, 15 µM BelV and 15 µM BelU in 100 mM potassium phosphate buffer (pH 7.8) were incubated at 25 °C for 6 h. The reactions were the quenched and analyzed (as above).

The CysC and BelV reaction mixtures (100 µl) containing 2 mM **4a** or **4b**, 3 mM amino acid, 3 mM ATP, 10 mM MgCl_2_ and 20 µM CysC or BelV in 100 mM potassium phosphate buffer (pH 7.8) were incubated at 25 °C for 5 h. The reaction was quenched by addition of 100 µl of acetonitrile and the precipitated protein was removed by centrifugation. The supernatant was diluted ten times with acetonitrile–H_2_O (50:50) and subjected to LC–MS analysis (method 1). Product yields for CysC reactions (average of three replicates) were determined from calibration curves using synthetic product standards. The yield of **25** was determined by ^19^F NMR.

The CysD and BelU reaction mixtures (100 µl) containing 2 mM **5** or **7**, 3 mM amino acid, 3 mM ATP, 10 mM MgCl_2_ and 20 µM CysD or BelU in 100 mM potassium phosphate buffer (pH 7.8) were incubated at 25 °C for 5 h. The reaction was quenched as described above. The supernatant was diluted ten times with acetonitrile–H_2_O (50:50) and subjected to LC–MS analysis (method 1). Product yields for CysD reactions (average of three replicates) were determined using calibration curves. The yield of **36** was determined by ^19^F NMR.

### Reporting summary

Further information on research design is available in the Nature Portfolio Reporting Summary linked to this article.

## Online content

Any methods, additional references, Nature Portfolio reporting summaries, source data, extended data, supplementary information, acknowledgements, peer review information; details of author contributions and competing interests; and statements of data and code availability are available at 10.1038/s41589-024-01657-7.

## Supplementary information

Supplementary Information Supplementary Figs. 1–8, Tables 1–4, methods and references.

Reporting Summary

Supplementary Data 1 DNA and protein sequences.

## Data Availability

The coordinates of the CysF X-ray crystal structures were deposited to PDB 8RA0. The AlphaFold structure of BhCysFE can be accessed at https://www.alphafold.ebi.ac.uk/entry/A0A562R406 (AF-A0A562R406-F1-model_v4). Structures used for modeling and docking studies can be accessed from PDB 5BSM, 5BSR, 5WM3, 5IE3, 4FUT, 4GXR and 4GXQ. All proteins characterized in this study can be accessed from UniProt using the accession codes presented in Supplementary Tables 1–3 and their synthetic gene sequences are provided in Supplementary Data 1. The remaining data are available in the main text or the Supplementary Information. Correspondence and requests for materials should be addressed to J.M.
