# Supplementary material for: Cryptic enzymatic assembly of peptides armed with β-lactone warheads
Source: Nat Chem Biol. 2024 Jul 1;20(10):1371–9. doi: 10.1038/s41589-024-01657-7 (PMC11427300; doi:10.1038/s41589-024-01657-7)
Supplement: Supplementary file 1 — Supplementary Figs. 1–8, Tables 1–4, methods and references. [file 41589_2024_1657_MOESM1_ESM.pdf]

nature chemical biology

Supplementary information https://doi.org/10.1038/s41589-024-01657-7

Cryptic enzymatic assembly of peptides armed with β-lactone warheads

In the format provided by the authors and unedited

Table of Contents

Supplementary figures and tables 2
Supplementary methods 13
Preparative scale enzymatic reactions 13
Synthesis of substrates and standards 17
NMR spectra of compounds synthesised enzymatically 39
NMR spectra of chemically synthesised standards 51
LC-MS/MS data for assays described in Extended Data Fig. 9-10 103
Supplementary references 115

Supplementary figures and tables

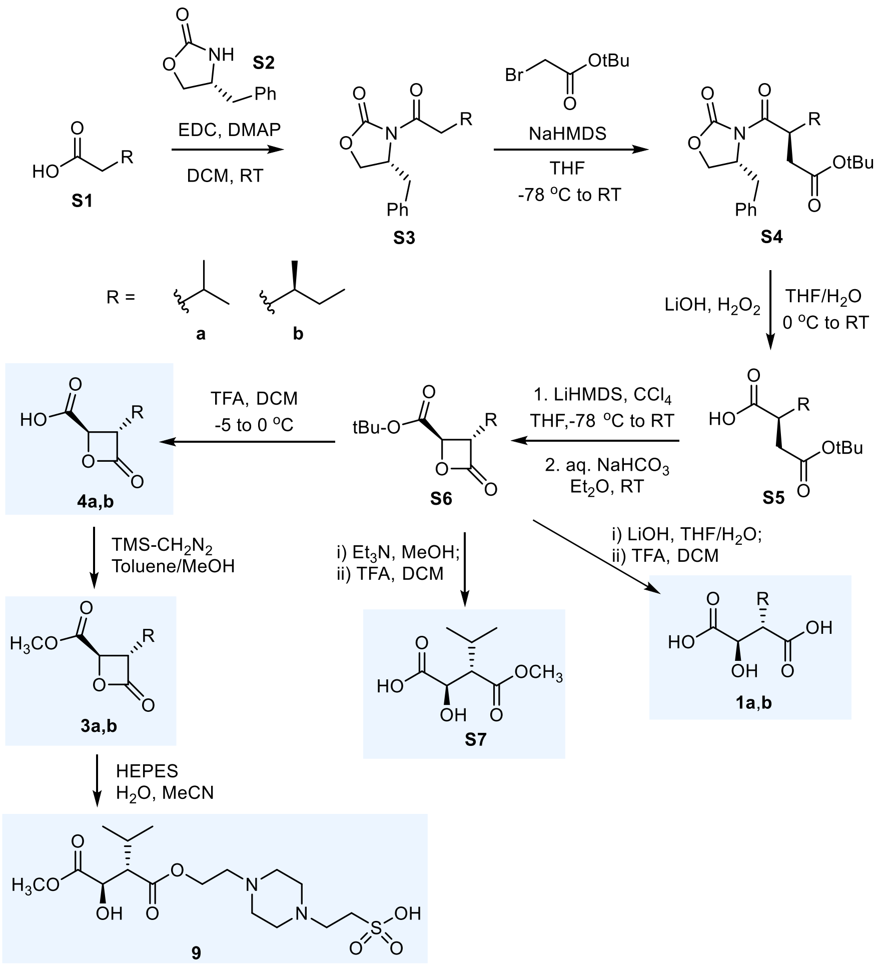

Supplementary Fig. 1. Synthesis of standards. Compound **1a/b**, **3a/b**, **4a/b**, **9**, and **S7** were synthesised for the validation of β-lactone warhead assembly pathway. **1a** is also commercially available. For detailed synthesis procedure see supplementary method section: synthesis of substrates and standards.

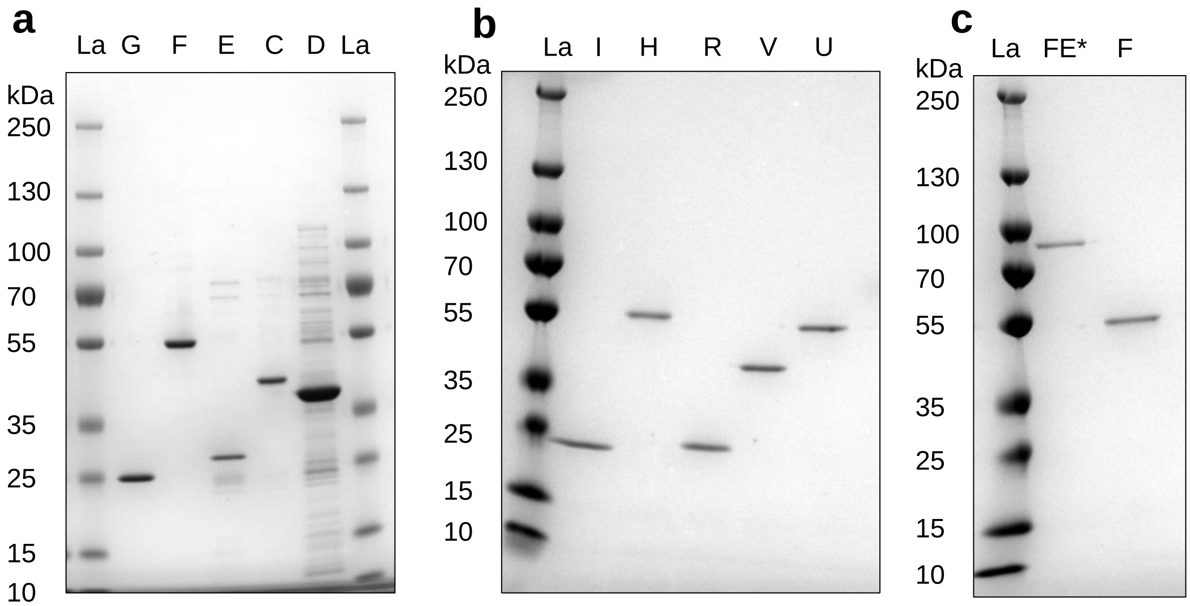

Supplementary Fig. 2. SDS-PAGE gel image of all purified enzymes in this study. a, All purified enzyme in the Cys pathway. **b,** All purified enzyme in the Bel pathway. **c,** Comparison of fused-CysFE and CysF. La = protein ladder, G = CysG (26.5 kDa), F = CysF (54.3 kDa), E = CysE (30.5 kDa), C = CysC (43.0 kDa), D = CysD (37.9 kDa), I = BelI (25.3 kDa), H = BelH (54.3 kDa), R = BelR (29.3 kDa), V = BelV (43.1 kDa), U = BelU (47.4 kDa), FE* = *Bh*CysFE fusion (84.4 kDa). All enzymes were expressed and purified at least twice and results are highly reproducible. The original scans are presented in Supplementary Fig. 107.

Supplementary Table 1. Cys pathway[1] proteins

Name	AA[2]	Function[4]	Expression vector	Uniprot ID	His-tag terminal	MW (kDa)[3]
CysG	228	Methyltransferase	pET-28a(+)	A0A1W6R556	C	26.53
CysF	491	Lactone synthetase	pET-21a(+)	A0A1W6R555	C	54.28
CysE	271	Hydrolase	pET-28a(+)	A0A1W6R564	C	30.48
CysC	392	Amide bond synthetase	pET-28a(+)	A0A1W6R559	C	43.04
CysD	333	ATP-grasp ligase	pET-21a(+)	A0A1W6R558	C	37.87

[1] Organism: *Kitasatospora cystarginea* NRRL B16505. [2] Amino acid length of the original protein without His-tag. [3] Molecular weight of the protein with His-tag. [4] Functions determined in this study.

Supplementary Table 2. Bel pathway[1] proteins

Name	AA[2]	Function[4]	Expression vector	UniProt ID	His-tag terminal	MW (kDa)[3]
BelI	228	Methyltransferase	pET-28a(+)	A0A1W6R583	C	25.32
BelH	491	Lactone synthetase	pET-28a(+)	A0A1W6R584	N	54.26
BelR	269	Hydrolase	pET-28a(+)	A0A1W6R592	C	29.27
BelV	389	Amide bond synthetase	pET-28a(+)	A0A1W6R589	C	43.10
BelU	420	ATP-grasp ligase	pET-21a(+)	A0A1W6R594	C	47.43

[1] Organism: *Streptomyces* sp. UCK 14. [2] Amino acid length of the original protein without His-tag. [3] Molecular weight of the protein with His-tag. [4] Functions determined in this study.

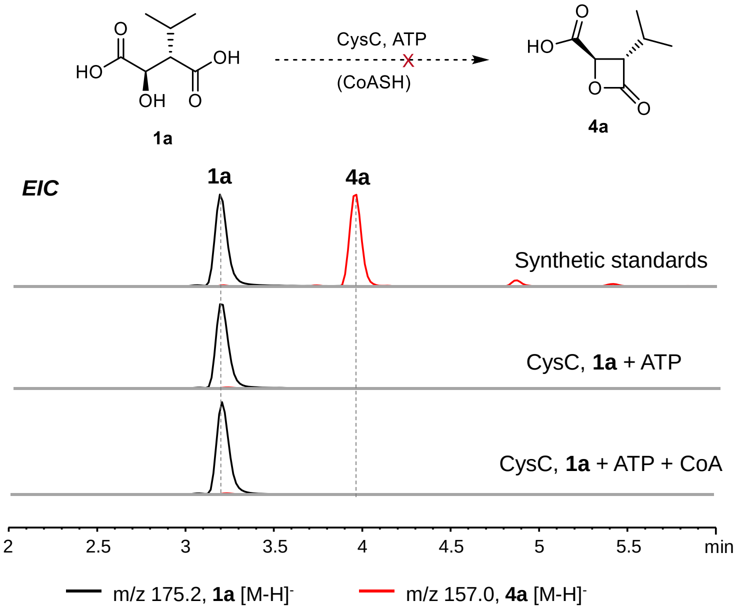

Supplementary Fig. 3. Testing the proposed lactonisation activity of CysC. For reaction conditions see method (enzyme assays).

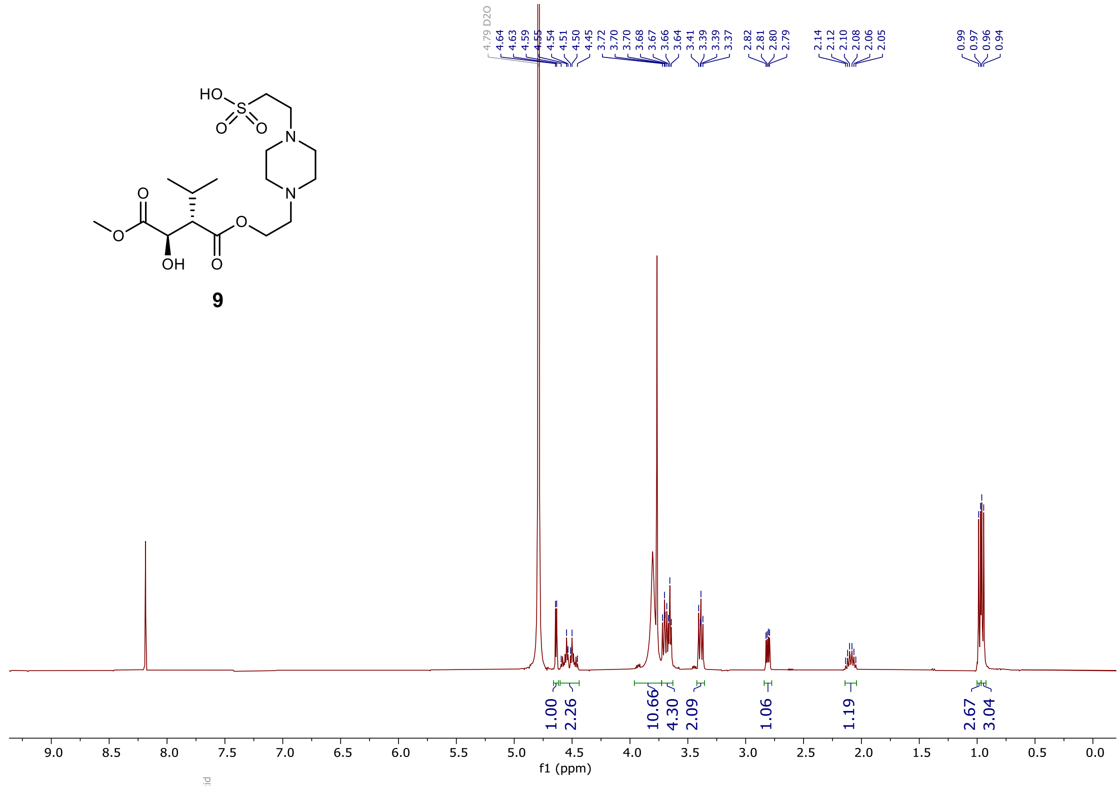

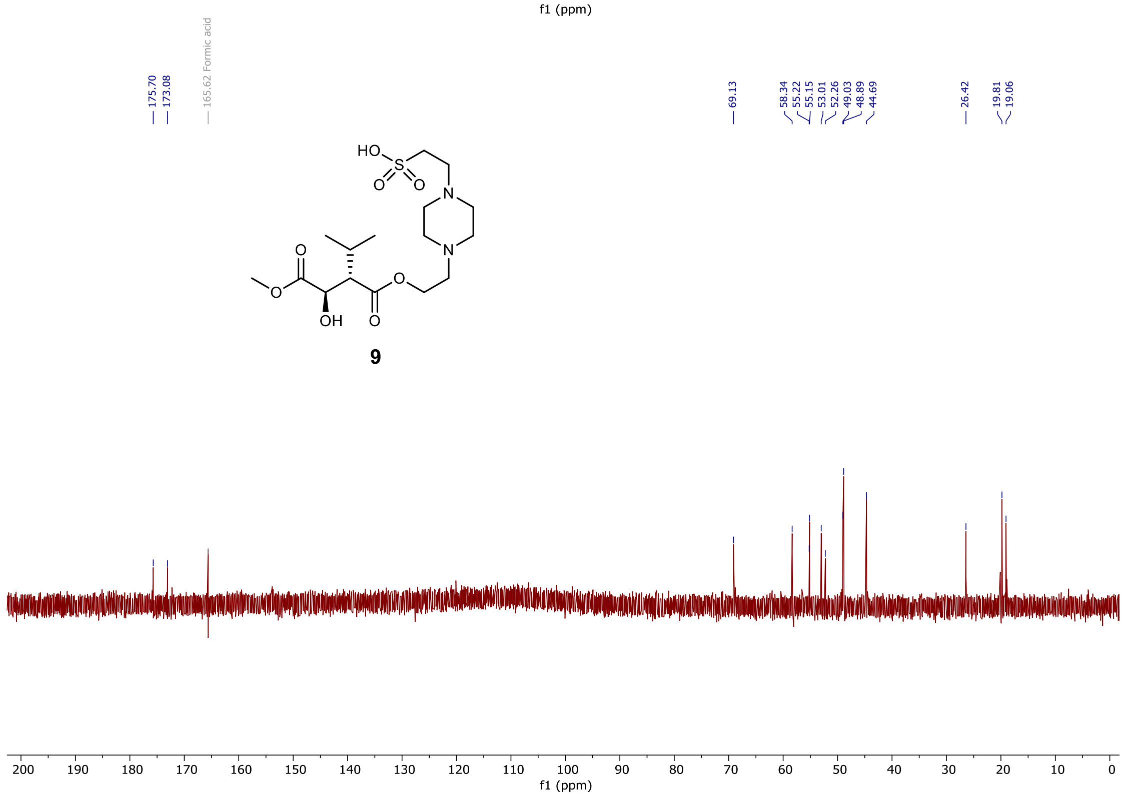

Supplementary Fig. 4. ^{1}H and ^{13}C NMR spectra of the HEPES buffer adduct 9.

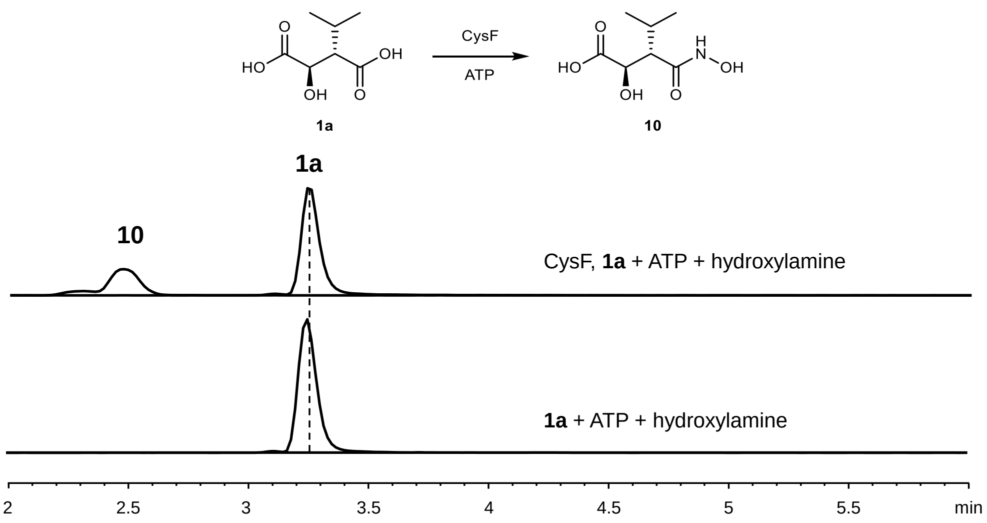

Supplementary Fig. 5. CysF catalysed reaction of hydroxylamine with 1a.

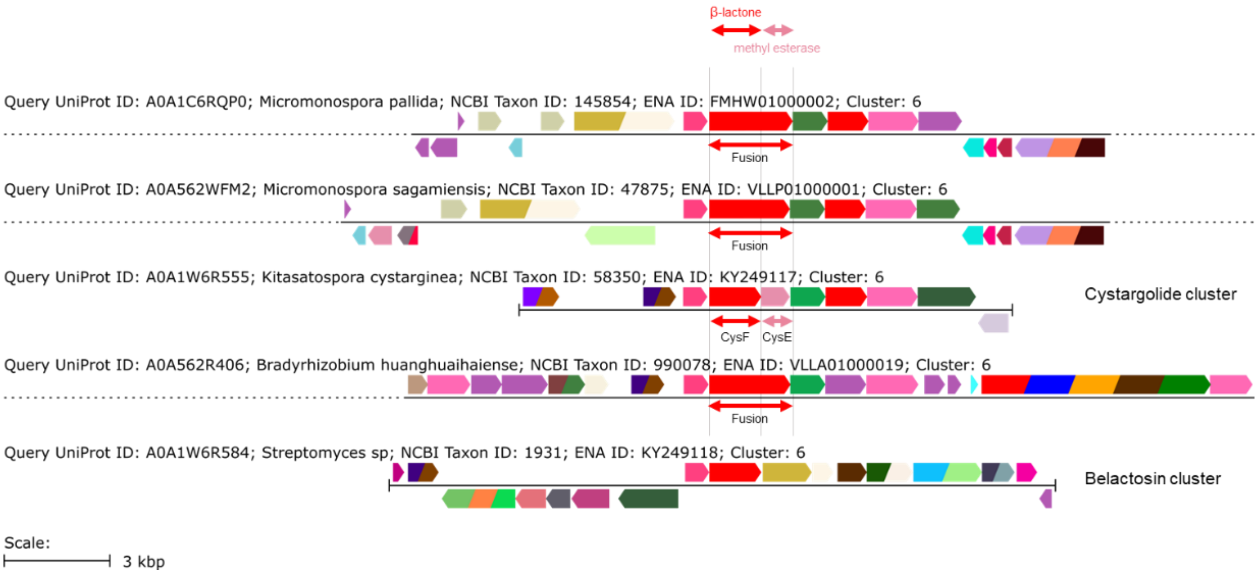

Supplementary Fig. 6. Examples of BGCs that contain fused-CysFE in nature.

Supplementary Table 3. Bifunctional fused CysFE explored in this study.

Name[1]	AA[2]	Organism	UniProt ID	MW[3] (*k*Da)
*Bh*CysFE	768	*Bradyrhizobium huanghuaihaiense*	A0A562R406	84.3
*Ms*CysFE	762	*Micromonospora sagamiensis*	A0A562WFM2	83.3
*Mp*CysFE	788	*Micromonospora pallida*	A0A1C6RQP0	85.8

[1] bifunctional lactone synthetase-hydrolase. [2]: Amino acid length of the original protein without His-tag. [3] Molecular weight of the protein with His-tag.

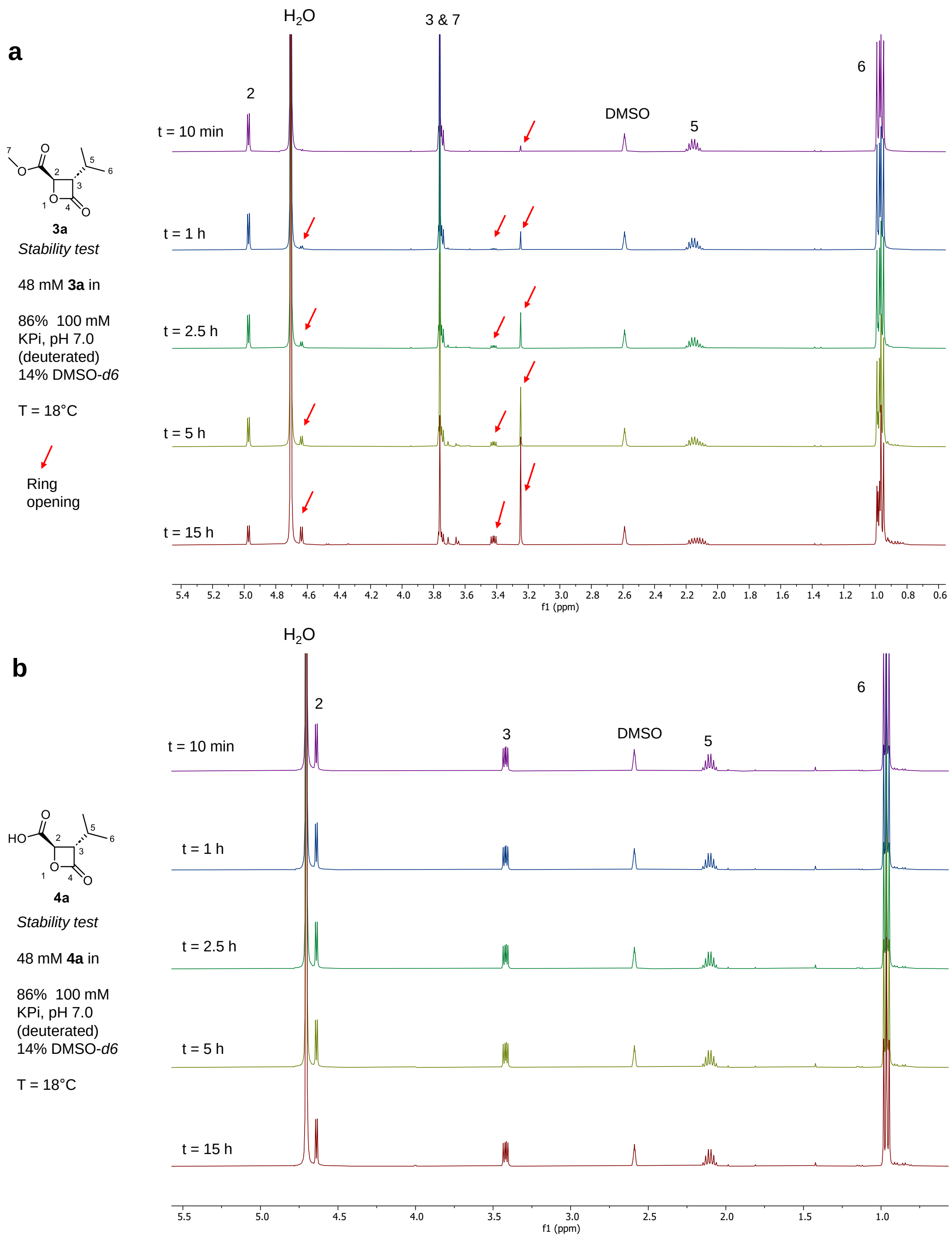

Supplementary Fig. 7. Stability comparison of 3a and 4a in KPi buffer. 0.02 mmol of **3a**/**4a** was dissolved in 90 µL of DMSO-*d6* and mixed with 540 µL of 100 mM deuterated KPi buffer. The mixture was transferred to NMR tube immediately and ^{1}H-NMR was recorded at different time points (T = 18 °C). **a,** ^{1}H-NMR spectra of 3a; hydrolysis product formation is indicated by the red arrows. **b,** 1H-NMR spectra of 4a; no change in the spectra was observed.

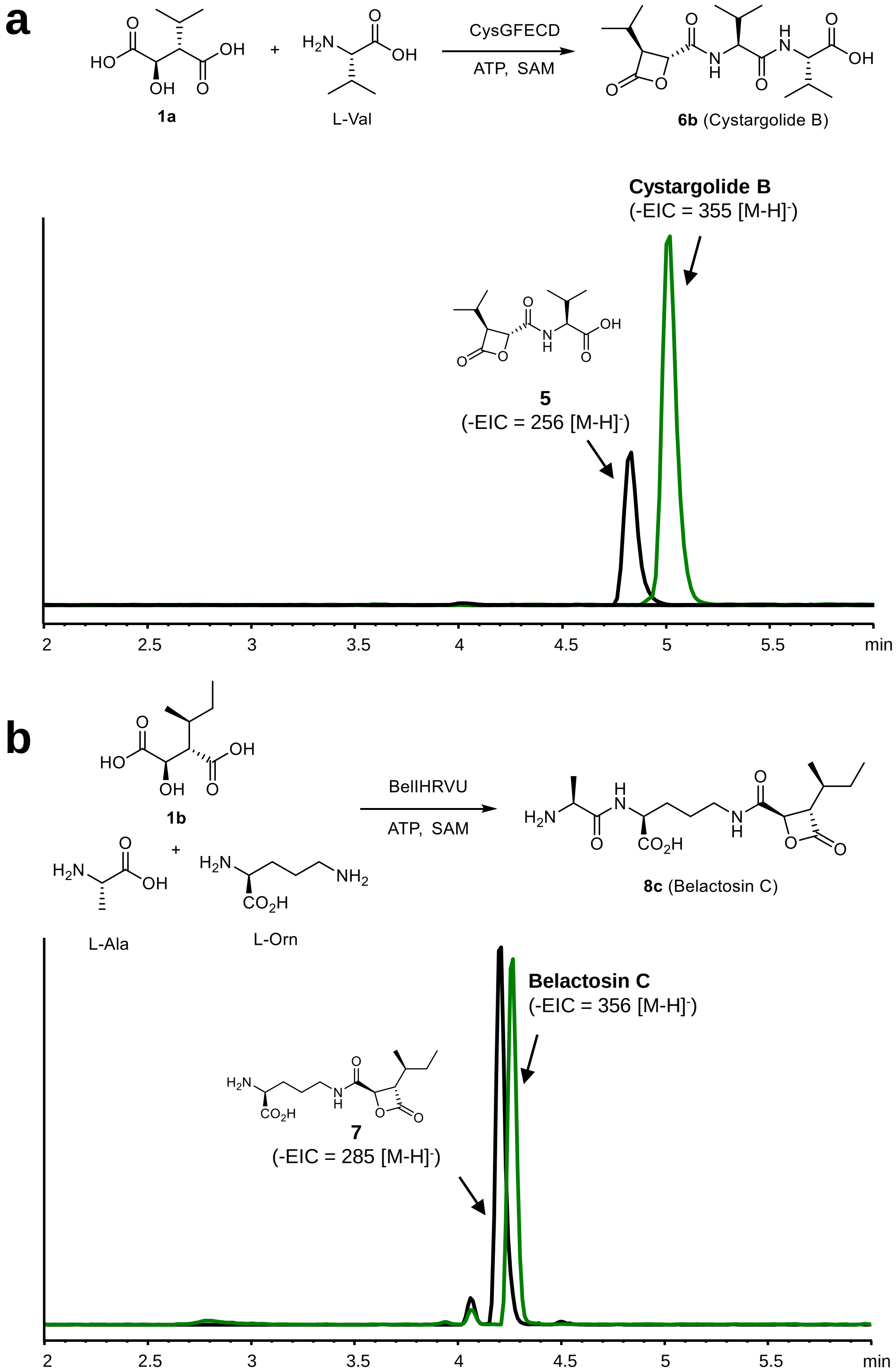

Supplementary Fig. 8. Reconstitution of the Cys and Bel pathway *in vitro*. a, Cys pathway. **b,** Bel pathway. LC-MS data. For detailed experimental procedure, see method section (enzyme assay).

Supplementary Table 4. **Crystallographic data collection and refinement statistics.** Statistics for the highest-resolution shell are shown in parentheses.

	CysF
Wavelength	0.9537
Resolution range	50.65 - 1.89 (1.94 - 1.89)
Space group	P 1 21 1
Unit cell	44.1 68.8 75.4 90 96.8 90
Total reflections	248200 (18265)
Unique reflections	35831 (2630)
Multiplicity	6.9 (6.9)
Completeness (%)	99.46 (95.43)
Mean I/sigma(I)	5.03 (0.60)
Wilson B-factor	26.08
R-merge	0.2418 (2.187)
R-meas	0.2616 (2.368)
R-pim	0.099 (0.89)
CC1/2	0.99 (0.38)
CC*	0.998 (0.74)
Reflections used in refinement	35745 (2612)
Reflections used for R-free	1763 (142)
R-work	0.1928 (0.3186)
R-free	0.2374 (0.3521)
Number of non-hydrogen atoms	4033
macromolecules	3752
ligands	26
solvent	255
Protein residues	485
RMS(bonds)	0.008
RMS(angles)	0.85
Ramachandran favored (%)	97.30
Ramachandran allowed (%)	2.7
Ramachandran outliers (%)	0.00
Rotamer outliers (%)	0.26
Clashscore	2.38
Average B-factor	33.15
macromolecules	33.13
ligands	39.93
solvent	32.75

Supplementary methods

Preparative scale enzymatic reactions

<u>Preparative scale CysG catalysed methylation of **1a**</u>

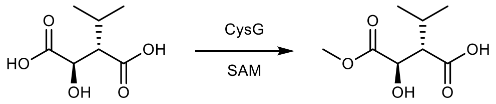

1a **2a**

A reaction mixture (20 mL) consisting of **1a** (35.2 mg, 10 mM), SAM (Sigma, ≥ 75% purity, 12 mM), $MgCl_2$ (10 mM), purified CysG (5 µM) in 100 mM KPi buffer (pH 7.5) was incubated at 30 °C with 200 rpm shaking for 18 h. A sample (50 µL) was taken for LC-MS analysis and indicated reaction completion before acidifying the reaction with 2 mL 1 M HCl to pH 2-3. The reaction was then extracted three times with EtOAc (20 mL, 10 mL, 10 mL). The organic layers were combined, washed with brine, dried over $MgSO_4$, and concentrated *in vacuo* to obtain 30.7 mg of **2a** as colourless oil in 81% yield.
^{1}H NMR (400 MHz, $CDCl_3$) δ 4.42 (d, *J* = 3.4 Hz, 1H), 3.79 (s, 3H), 2.62 (dd, *J* = 8.6, 3.4 Hz, 1H), 2.30 – 2.16 (m, 1H), 1.07 (dd, *J* = 11.1, 6.7 Hz, 6H).
^{13}C NMR (101 MHz, $CDCl_3$) δ 177.8, 174.3, 69.9, 55.0, 52.9, 27.4, 21.0, 20.3.
HR-MS, *m/z* (ESI+) calcd for $C_8H_{14}O_5$: 213.0739 $[M+Na]^+$, found 213.0732.
The NMR spectra agree with literature.[1]

<u>Preparative scale BelI catalysed methylation of **1b**</u>

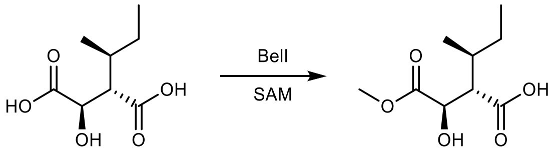

1b **2b**

A reaction mixture (10 mL) consisting of **1b** (19 mg, 10 mM), SAM (≥ 75% purity, treated as 75%, 12 mM), $MgCl_2$ (10 mM), purified BelI (5 µM) in 100 mM KPi buffer (pH 7.5) was incubated at 30 °C with 200 rpm shaking for 18 h. A sample (10 µL) was taken for LC-MS analysis and indicated reaction completion before acidifying the reaction with 2 mL 1 M HCl to pH 2-3. The reaction was then extracted with EtOAc three times (20 mL, 10 mL, 10 mL). The organic layers were combined, washed with brine, dried over $MgSO_4$ and concentrated *in vacuo* to obtain 15.6 mg **2b** as colourless oil in 76% yield.
^{1}H NMR (400 MHz, $CDCl_3$) δ 4.42 (d, *J* = 3.1 Hz, 1H), 3.80 (s, 3H), 2.77 (dd, *J* = 8.3, 3.0 Hz, 1H), 2.13 – 1.98 (m, 1H), 1.59 – 1.46 (m, 1H), 1.33 – 1.23 (m, 1H), 1.06 (d, *J* = 6.7 Hz, 3H), 0.94 (t, *J* = 7.4 Hz, 3H).
^{13}C NMR (101 MHz, $CDCl_3$) δ 178.3, 174.4, 69.5, 53.2, 53.0, 33.7, 27.6, 16.4, 11.4.
HR-MS, *m/z* (ESI+) calcd for $C_9H_{16}O_5$: 227.0890 $[M+Na]^+$, found 227.0889.

Preparative scale CysF catalysed lactonisation of **2a**

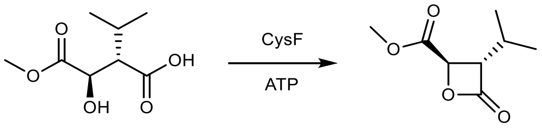

2a **3a**

A reaction mixture (15 mL) consisting of **2a** (28.5 mg, 10 mM), ATP (50 mM), $MgCl_2$ (10 mM), purified CysF (10 µM) in 100 mM KPi buffer (pH 7.5) was incubated at 30 °C with 200 rpm shaking for 18 h before extracting three times with Et_2O (3 x 15 mL). The organic layers were combined, washed with brine, dried over $MgSO_4$, and concentrated *in vacuo* to obtain 5 mg crude product. The crude product was subjected to ^{1}H-NMR analysis (see Extended Data Fig. 4).

Preparative scale CysGFE enzymatic cascade for the synthesis of **4a**

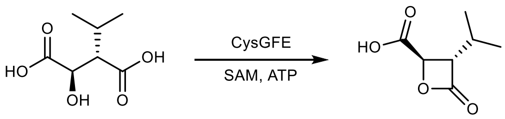

1a **4a**

A reaction mixture (25 mL) consisting of **1a** (22.0 mg, 5 mM), SAM (≥ 75% purity, 6 mM), ATP (10 mM), $MgCl_2$ (10 mM), purified CysG (20 µM) and purified *Bh*CysFE (20 µM) in 100 mM KPi buffer (containing 10% glycerol v/v, pH 7.5) was incubated at 25 °C with 200 rpm shaking for 24 h. A sample (50 µL) was taken for LC-MS analysis and indicated reaction completion before acidifying the reaction with 5 mL 1 M HCl to pH 2-3. The reaction was then extracted with EtOAc (3 x 15 mL). The organic layers were combined, washed with brine (15 mL), dried over $MgSO_4$, and concentrated *in vacuo* to obtain 18.5 mg **4a** as light-yellow oil in 94% yield.
^{1}H NMR (400 MHz, $CDCl_3$) δ 4.69 (d, *J* = 4.4 Hz, 1H), 3.62 (dd, *J* = 8.4, 4.4 Hz, 1H), 2.29 – 2.17 (m, 1H), 1.14 (d, *J* = 6.7 Hz, 3H), 1.10 (d, *J* = 6.7 Hz, 3H).
^{13}C NMR (101 MHz, $CDCl_3$) δ 173.4, 168.2, 69.3, 64.8, 28.0, 20.1, 19.7.
The NMR spectra agree with chemically synthesised **4a**.
HR-MS, *m/z* (ESI-) calcd for $C_7H_{10}O_4$: 157.0506 $[M-H]^-$, found 157.0508.

Preparative scale BelIHR enzymatic cascade for the synthesis of **4b**

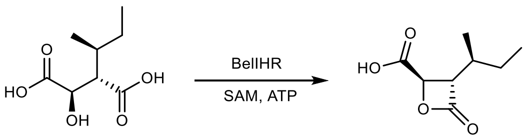

1b **4b**

A reaction mixture (25 mL) consisting of **1b** (23.7 mg, 5 mM), SAM (≥ 75% purity, 6 mM), ATP (10 mM), $MgCl_2$ (10 mM), purified BelI (20 µM), BelH (20 µM), and BelR (20 µM) in 100 mM KPi buffer (pH 7.5) was incubated at 25 °C with 200 rpm shaking for 24 h. A sample (50 µL) was taken for LC-MS analysis and indicated reaction completion before acidifying the reaction with 5 mL 1 M HCl to pH 2-3. The reaction was then extracted with EtOAc (3 x 15 mL). The organic layers were combined, washed with brine (15 mL), dried over $MgSO_4$, and concentrated *in vacuo* to obtain 18.7 mg **4b** as light-yellow oil in 87% yield.

^{1}H NMR (400 MHz, $CDCl_3$) δ 4.71 (d, *J* = 4.5 Hz, 1H), 3.76 (dd, *J* = 7.9, 4.5 Hz, 1H), 2.08 – 1.98 (m, 1H), 1.72 – 1.59 (m, 1H), 1.39 – 1.29 (m, 1H), 1.07 (d, *J* = 6.7 Hz, 3H), 0.95 (t, *J* = 7.5 Hz, 3H).
^{13}C NMR (101 MHz, $CDCl_3$) δ 173.75, 168.51, 68.79, 63.33, 33.83, 26.85, 16.37, 11.10.
The NMR spectra agree with chemically synthesised **4b**.
HR-MS, *m/z* (ESI-) calcd for $C_8H_{12}O_4$: 171.0663 $[M-H]^-$, found 171.0667.

<u>Preparative scale CysCD enzymatic cascade (stepwise)</u>

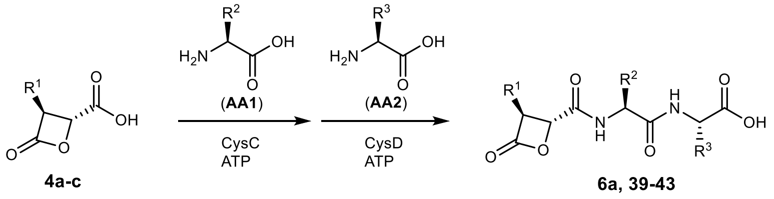

General method: A reaction mixture (20 mL) consisting of **4a-c** (4 mM, 1.0 equiv, 0.08 mmol), **AA1** (4 mM, 1.0 equiv), ATP (4.4 mM, 1.1 equiv), $MgCl_2$ (10 mM), 100 mM NaCl, and purified CysC (20 µM) in 100 mM KPi buffer (pH 7.8) was incubated at 25 °C with 200 rpm shaking. After 12 h of incubation, additional ATP (1.1 equiv, 0.88 mL of 100 mM stock solution in KPi buffer), **AA2** (1.25 equiv, 1.0 mL of 100 mM stock solution in KPi buffer), and purified CysD (20 µM) were then added. The reaction mixture was incubated at 25 °C with 200 rpm shaking for another 12 h. The reaction mixture was then cooled to 0 °C on ice before acidifying with 1.0 M HCl (1.8 mL) to pH 5-6. The resulting mixture was extracted with ice-cold EtOAc (3 x 40 mL). During extraction, the mixture was kept ice-cold to minimise acid catalysed β-lactone ring opening in water. The organic layers were combined, washed with brine (20 mL), dried over $MgSO_4$, and concentrated *in vacuo* to obtain crude product. The crude product was purified by silica gel column (DCM/MeOH with 0.1% AcOH, 1-4% MeOH) to obtain purified warhead-peptides.

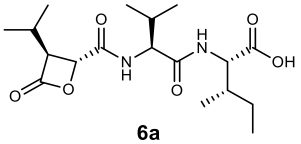

6a
(Cystargolide A)
Enzymatic

6a was obtained as white solid, 16 mg, 54% yield over two steps.
^{1}H NMR (500 MHz, MeOD) δ 4.81 (d, *J* = 4.3 Hz, 1H), 4.37 (d, *J* = 5.8 Hz, 1H), 4.30 (d, *J* = 7.9 Hz, 1H), 3.56 (dd, *J* = 8.5, 4.4 Hz, 1H), 2.23 – 2.15 (m, 1H), 2.14 – 2.07 (m, 1H), 1.95 – 1.86 (m, 1H), 1.58 – 1.48 (m, 1H), 1.29 – 1.21 (m, 1H), 1.10 (d, *J* = 6.8 Hz, 3H), 1.06 (d, *J* = 6.7 Hz, 3H), 1.00 – 0.91 (m, 12H).
^{13}C NMR (126 MHz, MeOD) δ 174.5, 173.3, 170.6, 170.4, 72.2, 65.2, 60.2, 58.22, 38.3, 32.0, 28.8, 26.2, 20.4, 19.8, 19.7, 18.8, 16.0, 11.8.
HR-MS, *m/z* (ESI-) calcd for $C_{18}H_{30}N_2O_6$: 369.2031 $[M-H]^-$, found 369.2054.

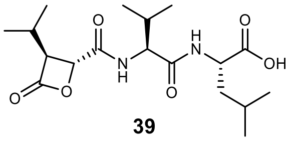

39
Enzymatic

39 was obtained as white solid, 8 mg, 27% yield over two steps.
^{1}H NMR (500 MHz, MeOD) δ 4.81 (d, *J* = 4.4 Hz, 1H), 4.45 (t, *J* = 7.5 Hz, 1H), 4.26 (d, *J* = 7.8 Hz, 1H), 3.55 (dd, *J* = 8.5, 4.4 Hz, 1H), 2.22 – 2.08 (m, 2H), 1.72 (d, *J* = 6.7 Hz, 1H), 1.65 (t, *J* = 7.3 Hz, 2H), 1.10 (d, *J* = 6.7 Hz, 3H), 1.06 (d, *J* = 6.7 Hz, 3H), 1.00 (d, *J* = 6.7 Hz, 3H), 0.97 (d, *J* = 1.4 Hz, 3H), 0.95 (d, *J* = 1.6 Hz, 3H), 0.91 (d, *J* = 6.4 Hz, 3H).
^{13}C NMR (126 MHz, MeOD) δ 175.7, 173.2, 170.6, 170.4, 72.2, 65.3, 60.2, 52.0, 41.6, 32.0, 28.8, 25.9, 23.4, 21.8, 20.4, 19.8, 19.7, 18.8.
HR-MS, *m/z* (ESI-) calcd for $C_{18}H_{30}N_2O_6$: 369.2031 [M-H]$^-$, found 369.2096.

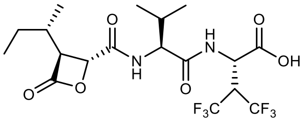

40
Enzymatic

40 was obtained as a light yellow solid, 24 mg, 63% yield over two steps. Unlike other examples, the racemic amino acid (**AA2**, 2.5 equiv) was used in this case. We observed the formation of only one diastereomer, indicating a complete kinetic resolution by the enzyme.
^{1}H NMR (500 MHz, MeOD) δ 5.41 (s, 1H), 4.82 (d, *J* = 4.4 Hz, 1H), 4.38 (d, *J* = 8.3 Hz, 1H), 4.36 – 4.27 (m, 1H), 3.66 (dd, *J* = 8.0, 4.4 Hz, 1H), 2.20 – 2.12 (m, 1H), 2.02 – 1.93 (m, 1H), 1.68 – 1.59 (m, 1H), 1.36 – 1.30 (m, 1H), 1.04 (d, *J* = 6.7 Hz, 3H), 0.99 (d, *J* = 6.7 Hz, 3H), 0.97 – 0.91 (m, 6H).
^{13}C NMR (126 MHz, MeOD) δ 173.5, 170.8, 170.5, 71.6, 63.8, 60.2, 34.8, 31.6, 27.7, 19.6, 18.6, 16.6, 11.3.
^{19}F NMR (471 MHz, MeOD) δ -63.6 (q, *J* = 9.3 Hz), -67.1 (q, *J* = 8.9 Hz).
HR-MS, *m/z* (ESI-) calcd for $C_{18}H_{24}F_6N_2O_6$: 477.1460 [M-H]$^-$, found 477.1453.

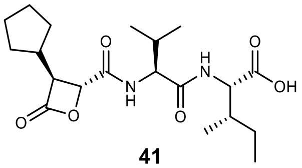

41
Enzymatic

41 was obtained as a light yellow solid, 10 mg, 32% yield over two steps.
^{1}H NMR (500 MHz, MeOD) δ 4.76 (d, *J* = 4.3 Hz, 1H), 4.37 (d, *J* = 5.8 Hz, 1H), 4.29 (d, *J* = 7.9 Hz, 1H), 3.70 (dd, *J* = 8.8, 4.3 Hz, 1H), 2.42 – 2.33 (m, 1H), 2.15 – 2.06 (m, 1H), 1.97 – 1.83 (m, 3H), 1.75 – 1.59 (m, 4H), 1.57 – 1.50 (m, 1H), 1.46 – 1.36 (m, 2H), 1.27 – 1.23 (m, 1H), 1.01 – 0.91 (m, 12H).
^{13}C NMR (126 MHz, MeOD) δ 174.6, 173.2, 170.9, 170.5, 72.9, 62.9, 60.2, 58.3, 39.5, 38.3, 31.9, 31.2, 30.8, 26.2, 26.0, 25.7, 19.7, 18.9, 16.0, 11.8.
HR-MS, *m/z* (ESI-) calcd for $C_{20}H_{32}N_2O_6$: 395.2182 [M-H]$^-$, found 395.2147.

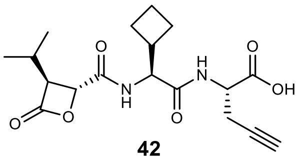

42
Enzymatic

42 was obtained as a white solid, 10 mg, 34% yield over two steps.
^{1}H NMR (500 MHz, MeOD) δ 4.79 (d, *J* = 4.4 Hz, 1H), 4.48 – 4.43 (m, 2H), 3.62 (dd, *J* = 8.4, 4.4 Hz, 1H), 2.79 – 2.67 (m, 3H), 2.32 (t, *J* = 2.7 Hz, 1H), 2.22 – 2.13 (m, 1H), 2.08 – 1.97 (m, 3H), 1.93 – 1.81 (m, 3H), 1.10 (d, *J* = 6.7 Hz, 3H), 1.06 (d, *J* = 6.7 Hz, 3H).
^{13}C NMR (126 MHz, MeOD) δ 172.4, 170.7, 170.6, 80.3, 72.2, 72.1, 65.4, 59.0, 53.0, 38.6, 28.9, 26.2, 26.2, 22.5, 20.4, 19.8, 18.8.
HR-MS, *m/z* (ESI-) calcd for $C_{18}H_{24}N_2O_6$: 363.1556 [M-H]$^-$, found 363.1570.

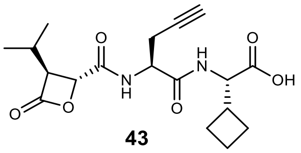

43
Enzymatic

43 was obtained as a white solid, 4 mg, 14% yield over two steps.
^{1}H NMR (500 MHz, MeOD) δ 4.79 (d, *J* = 4.3 Hz, 1H), 4.64 (dd, *J* = 8.7, 5.2 Hz, 1H), 4.34 (d, *J* = 8.4 Hz, 1H), 3.61 (dd, *J* = 8.4, 4.4 Hz, 1H), 2.80 – 2.63 (m, 3H), 2.39 (t, *J* = 2.7 Hz, 1H), 2.22 – 2.14 (m, 1H), 2.07 – 1.95 (m, 3H), 1.93 – 1.79 (m, 3H), 1.11 (d, *J* = 6.7 Hz, 3H), 1.07 (d, *J* = 6.7 Hz, 3H).
^{13}C NMR (126 MHz, MeOD) δ 172.0, 170.7, 170.6, 80.1, 72.4, 72.2, 65.4, 57.9, 53.4, 38.5, 28.9, 26.3, 25.9, 22.6, 20.5, 19.8, 18.7.
HR-MS, *m/z* (ESI-) calcd for $C_{18}H_{24}N_2O_6$: 363.1556 [M-H]$^-$, found 363.1508.

Synthesis of substrates and standards

<u>Synthesis of β-lactones **4a-c** (adapted from literature with modifications)</u>[2,3]

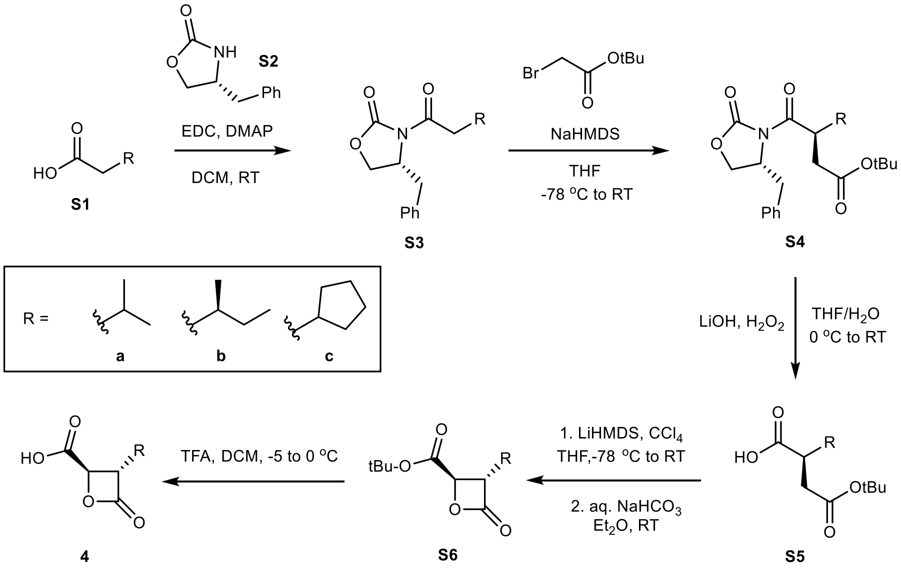

General procedure synthesis of ***S3*** *(Step 1):* To a solution of Evans' chiral auxiliary **S2** (751 mg, 4.2 mmol, 1.0 equiv) in 10 mL DCM, at room temperature, was added EDC hydrochloride (1.6 g, 8.4 mmol, 2.0 equiv), carboxylic acid **S1** (5.0 mmol, 1.2 equiv), and DMAP (513 mg, 4.2 mmol, 1.0 equiv). The mixture was stirred at RT for 16-20 h. The reaction was diluted with DCM and washed with 1 M HCl, followed by sat. $NaHCO_3$, sat. NH_4Cl, and finally brine. The organic layer was dried over $MgSO_4$ and concentrated *in vacuo*. The crude product was purified by silica gel column (EtOAc/Hexane, 20-30% EtOAc) to obtain **S3b** and **S3c**. A sample of **S3a** was also purchased from Fluorochem (UK) and used for the next step directly.

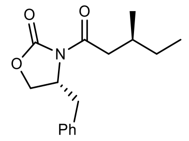

S3b

S3b was obtained as a white solid, 4.36 g, 89% yield.
^{1}H NMR (400 MHz, $CDCl_3$) δ 7.41 – 7.21 (m, 5H), 4.79 – 4.65 (m, 1H), 4.28 – 4.15 (m, 2H), 3.35 (dd, *J* = 13.3, 3.4 Hz, 1H), 2.99 – 2.71 (m, 3H), 2.12 – 1.96 (m, 1H), 1.55 – 1.41 (m, 1H), 1.38 – 1.22 (m, 1H), 1.03 (d, *J* = 6.7 Hz, 3H), 0.96 (t, *J* = 7.4 Hz, 3H).
^{13}C NMR (101 MHz, $CDCl_3$) δ 173.1, 153.6, 135.5, 129.6, 129.1, 127.5, 66.2, 55.4, 42.3, 38.1, 31.4, 29.5, 19.4, 11.5.
The NMR spectra is in agreement with published data.[4]
HR-MS, *m/z* (ESI+) calcd for $C_{16}H_{21}NO_3$: 298.1414 $[M+Na]^+$, found 298.1410.

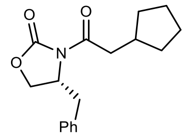

S3c

S3c was obtained as a white solid, 960 mg, 79% yield,
^{1}H NMR (400 MHz, $CDCl_3$) δ 7.42 – 7.20 (m, 5H), 4.76 – 4.63 (m, 1H), 4.28 – 4.12 (m, 2H), 3.33 (dd, *J* = 13.4, 3.3 Hz, 1H), 3.05 (dd, *J* = 16.6, 6.9 Hz, 1H), 2.93 (dd, *J* = 16.6, 7.4 Hz, 1H), 2.78 (dd, *J* = 13.4, 9.6 Hz, 1H), 2.44 – 2.28 (m, 1H), 1.99 – 1.84 (m, 2H), 1.74 – 1.53 (m, 4H), 1.33 – 1.15 (m, 2H).
^{13}C NMR (101 MHz, $CDCl_3$) δ 173.2, 153.6, 135.5, 129.6, 129.1, 127.4, 66.2, 55.3, 41.5, 38.1, 35.9, 32.7, 32.6, 25.1, 25.1.
The NMR spectra is in agreement with published data.[5]
HR-MS, *m/z* (ESI+) calcd for $C_{17}H_{21}NO_3$: 310.1419 $[M+Na]^+$, found 310.1422.

General procedure for the synthesis of ***S4****:* To a solution of **S3** (10.0 mmol, 1.0 equiv) in 50 mL of dry THF (cooled to -78 °C under N_2 atmosphere) was added NaHMDS (7.5 mL of 2 M solution in THF, 30 mmol, 1.5 equiv) dropwise and the solution was kept at -78 °C for 1 h before *tert*-butyl bromoacetate (3.9 g, 20 mmol, 2.0 equiv) was added dropwise. The resulting solution was stirred for 16 h and gradually reached room temperature before quenching with 1.3 mL of acetic acid. The mixture was concentrated *in vacuo* and the residue was added to 100 mL ethyl acetate, followed by washing with 100 mL water and 50 mL brine. The organic layer was dried

over $MgSO_4$ and concentrated *in vacuo* to give crude product, which was either recrystallised in EtOAc/hexane (1/20) or purified by silica gel column to give **S4**.

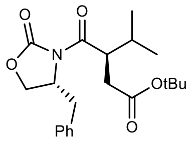

S4a

S4a was obtained as light-yellow needle shape crystal, 3.0 g, 80% yield.
^{1}H NMR (400 MHz, $CDCl_3$) δ 7.37 – 7.26 (m, 5H), 4.72 – 4.60 (m, 1H), 4.20 – 4.08 (m, 3H), 3.35 (dd, J = 13.5, 3.3 Hz, 1H), 2.88 – 2.67 (m, 2H), 2.45 (dd, J = 16.9, 3.6 Hz, 1H), 2.06 – 1.92 (m, 1H), 1.42 (s, 9H), 1.01 (d, J = 6.8 Hz, 3H), 0.91 (d, J = 6.9 Hz, 3H).
^{13}C NMR (101 MHz, $CDCl_3$) δ 175.7, 172.0, 136.1, 129.7, 129.1, 127.3, 80.8, 65.9, 55.9, 44.6, 37.6, 33.7, 29.9, 28.2, 20.9, 18.5.
The NMR spectra is in agreement with published data.[6]
HR-MS, *m/z* (ESI+) calcd for $C_{21}H_{29}NO_5$: 398.1943 $[M+Na]^+$, found 398.1962.

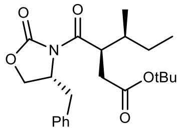

S4b

S4b was obtained as light-yellow needle shape crystal, 4.0 g, 64% yield.
^{1}H NMR (400 MHz, $CDCl_3$) δ 7.36 – 7.19 (m, 5H), 4.68 – 4.54 (m, 1H), 4.24 – 4.15 (m, 1H), 4.15 – 4.09 (m, 2H), 3.32 (dd, J = 13.5, 3.3 Hz, 1H), 2.88 – 2.65 (m, 2H), 2.34 (dd, J = 16.9, 3.5 Hz, 1H), 1.82 – 1.68 (m, 1H), 1.50 – 1.31 (m, 10H), 1.31 – 1.17 (m, 1H), 0.91 (t, J = 7.4 Hz, 3H), 0.83 (d, J = 6.8 Hz, 3H).
^{13}C NMR (101 MHz, $CDCl_3$) δ 175.7, 172.1, 153.1, 136.0, 129.7, 129.0, 127.3, 80.8, 65.9, 55.9, 43.6, 37.6, 35.8, 32.4, 28.2, 28.0, 14.9, 12.0.
HR-MS, *m/z* (ESI+) calcd for $C_{22}H_{31}NO_5$: 412.2100 $[M+Na]^+$, found 412.2110.

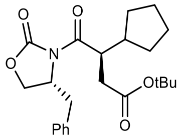

S4c

S4c was obtained as white needle shape crystal, 1.0 g, 79% yield.
^{1}H NMR (400 MHz, $CDCl_3$) δ 7.35 – 7.21 (m, 5H), 4.73 – 4.54 (m, 1H), 4.27 – 4.17 (m, 1H), 4.14 – 4.08 (m, 2H), 3.36 (dd, J = 13.5, 3.3 Hz, 1H), 2.82 (dd, J = 16.8, 11.3 Hz, 1H), 2.69 (dd, J = 13.5, 10.3 Hz, 1H), 2.50 (dd, J = 16.8, 3.7 Hz, 1H), 2.06 – 1.89 (m, 1H), 1.80 – 1.45 (m, 6H), 1.39 (s, 9H), 1.31 – 1.16 (m, 2H).
^{13}C NMR (101 MHz, $CDCl_3$) δ 176.4, 171.6, 153.3, 136.2, 129.6, 129.0, 127.3, 80.8, 65.9, 56.0, 42.9, 42.7, 37.5, 37.0, 30.2, 30.0, 28.2, 25.0, 24.9.
HR-MS, *m/z* (ESI+) calcd for $C_{23}H_{31}NO_5$: 424.2100 $[M+Na]^+$, found 424.2102.
The NMR spectra is in agreement with published data.[5]

General Procedure for the synthesis of ***S5****:* A solution of **S4a** (3.0 g, 8.0 mmol, 1.0 equiv) in 40 mL of THF and 7 mL of water was cooled to 0 °C and treated with 5.6 mL of a 30% wt. H_2O_2 solution. The mixture was stirred for 5 min, followed by addition of LiOH (monohydrate, 0.85 g, 20 mmol, 2.5 equiv, freshly prepared in 13 mL of water) dropwise. The reaction mixture was stirred and allowed to gradually reach room temperature for 16 h before cooling back to 0 °C and quenching with 9 mL of a 2 M $Na_2S_2O_3$ solution. The reaction mixture was concentrated *in vacuo* to remove THF and the resulting solution was diluted with 20 mL water. The pH was adjusted to 8-9 using 2 M NaOH solution (if necessary). The aqueous solution was washed with EtOAc to remove the Evans' chiral auxiliary. The aqueous layer was then acidified with 2 M HCl solution (to pH 1-2) and extracted with EtOAc three times. The organic layers were combined, washed with brine, dried over $MgSO_4$, and concentrated *in vacuo*. The crude product was purified by silica gel column (EtOAc/Hexane, 20% EtOAc) to give 1.5 g of **S5a** in 87% yield.

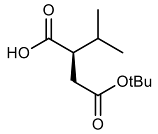

S5a

S5a was obtained as a colourless oil, 1.5 g, 87% yield.
^{1}H NMR (400 MHz, $CDCl_3$) δ 2.75 – 2.57 (m, 2H), 2.36 (dd, *J* = 16.2, 3.7 Hz, 1H), 2.10 – 1.96 (m, 1H), 1.43 (s, 8H), 0.97 (dd, *J* = 11.5, 6.8 Hz, 6H).
^{13}C NMR (101 MHz, $CDCl_3$) δ 171.7, 81.1, 47.6, 34.2, 30.0, 28.1, 20.3, 19.6.
HR-MS, *m/z* (ESI+) calcd for $C_{11}H_{20}O_4$: 239.1259 $[M+Na]^+$, found 239.1266.
The NMR spectra is in agreement with published data.[2]

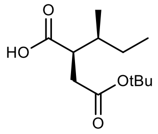

S5b

S5b was obtained as a colourless oil, 1.2 g, 62% yield.
^{1}H NMR (400 MHz, $CDCl_3$) δ 2.92 – 2.80 (m, 1H), 2.58 (dd, *J* = 16.6, 11.0 Hz, 1H), 2.26 (dd, *J* = 16.6, 3.8 Hz, 1H), 1.92 – 1.75 (m, 1H), 1.50 – 1.32 (m, 10H), 1.30 – 1.16 (m, 1H), 0.96 – 0.81 (m, 6H).
^{13}C NMR (101 MHz, $CDCl_3$) δ 181.3, 171.8, 81.0, 45.8, 36.3, 32.8, 28.1, 27.3, 15.9, 11.9.
HR-MS, *m/z* (ESI+) calcd for $C_{12}H_{22}O_4$: 253.1416 $[M+Na]^+$, found 253.1410.
The NMR spectra is in agreement with published data.[7]

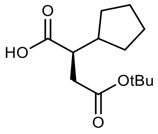

S5c

S5c was obtained as colourless oil, 480 mg, 80% yield.
^{1}H NMR (400 MHz, $CDCl_3$) δ 2.69 – 2.57 (m, 2H), 2.49 – 2.37 (m, 1H), 2.02 – 1.90 (m, 1H), 1.84 – 1.70 (m, 2H), 1.68 – 1.59 (m, 2H), 1.58 – 1.49 (m, 2H), 1.42 (s, 9H), 1.38 – 1.29 (m, 1H), 1.27 – 1.14 (m, 1H).

^{13}C NMR (101 MHz, $CDCl_3$) δ 181.1, 181.1, 171.4, 81.2, 46.5, 42.2, 36.8, 30.6, 30.6, 28.1, 25.1, 25.0.
HR-MS, *m/z* (ESI-) calcd for $C_{13}H_{22}O_4$: 241.1445 $[M-H]^-$, found 241.1448.
The NMR spectra is in agreement with published data.[5]

General Procedure for synthesis of ***S6:*** A solution of **S5a** (1.5 g, 6.9 mmol, 1 equiv) in 20 mL of dry THF was cooled to -78 °C and treated with 14 mL of 1 M LiHMDS (14 mmol, 2 equiv) dropwise. The solution was kept at -78 °C for 1 h before dry CCl_4 (1.3 g, 8.3 mmol, 1.2 equiv) was added dropwise. The reaction mixture was stirred and allowed to gradually reach room temperature for 12 h before concentrating *in vacuo*. The resulting gummy solid residue was suspended in 100 mL of Et_2O and 50 mL of 5% (w/v) $NaHCO_3$ solution. The mixture was vigorously stirred at room temperature for 24 h and then diluted with Et_2O, washed with sat. $NaHCO_3$ solution and brine, dried over $MgSO_4$ and concentrated *in vacuo*. The crude product was purified by silica gel column (EtOAc/Hexane, 2%-10% EtOAc) to give **S6a**.

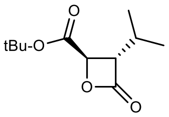

S6a

S6a was obtained as a white solid, 1.0 g, 67% yield.
^1H NMR (400 MHz, $CDCl_3$) δ 4.52 (d, *J* = 4.4 Hz, 1H), 3.45 (dd, *J* = 8.4, 4.4 Hz, 1H), 2.27 – 2.08 (m, 1H), 1.51 (s, 9H), 1.12 (d, *J* = 6.7 Hz, 3H), 1.07 (d, *J* = 6.7 Hz, 3H).
^{13}C NMR (101 MHz, $CDCl_3$) δ 168.9, 167.6, 83.7, 70.3, 64.2, 28.1, 27.8, 20.1, 19.8.
HR-MS, *m/z* (ESI+) calcd for $C_{11}H_{18}O_4$: 237.1103 $[M+Na]^+$, found 237.1105.
The NMR spectra is in agreement with published data.[2]

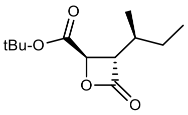

S6b

S6b was obtained as a white solid, 760 mg, 64% yield.
^1H NMR (400 MHz, $CDCl_3$) δ 4.53 (d, *J* = 4.4 Hz, 1H), 3.60 (dd, *J* = 7.7, 4.5 Hz, 1H), 2.05 – 1.90 (m, 1H), 1.68 – 1.56 (m, 1H), 1.50 (s, 9H), 1.39 – 1.26 (m, 1H), 1.03 (d, *J* = 6.7 Hz, 3H), 0.93 (t, *J* = 7.5 Hz, 3H).
^{13}C NMR (101 MHz, $CDCl_3$) δ 169.2, 167.6, 83.6, 69.6, 62.5, 33.6, 28.0, 26.9, 16.4, 11.2.
The NMR spectra is in agreement with published data.[3]
HR-MS, *m/z* (ESI+) calcd for $C_{12}H_{20}O_4$: 251.1259 $[M+Na]^+$, found 251.1254.

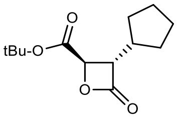

S6c

S6c was obtained as a light-yellow solid, 172 mg, 36% yield.
^1H NMR (400 MHz, $CDCl_3$) δ 4.49 (d, *J* = 4.3 Hz, 1H), 3.60 (dd, *J* = 8.6, 4.3 Hz, 1H), 2.41 – 2.27 (m, 1H), 1.99 – 1.80 (m, 2H), 1.74 – 1.58 (m, 4H), 1.50 (s, 9H), 1.45 – 1.32 (m, 2H).

^{13}C NMR (101 MHz, $CDCl_3$) δ 169.2, 167.6, 83.6, 71.0, 61.7, 38.3, 30.2, 29.9, 28.1, 25.1, 24.9.
The NMR spectra is in agreement with published data.[5]
HR-MS, *m/z* (ESI+) calcd for $C_{13}H_{20}O_4$: 263.1259 $[M+Na]^+$, found 263.1260.

General Procedure for the synthesis of ***4:*** A solution of **S6a** (262 mg, 1.2 mmol) in 1 mL of DCM was cooled to 0 °C and treated with 1 mL of TFA dropwise. The mixture was stirred at -5 °C to 0 °C for 12 h before removal of the DCM and TFA at 0 °C under high vacuum to give **4a**. (Note that removal of DCM and TFA at higher temperature can lead to partial epimerisation of product). The crude product was purified (in the case of **4c**) by silica gel column (DCM/MeOH with 0.1% acetic acid, 0-5% MeOH).

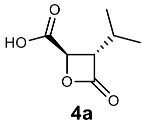

4a

4a was obtained as a viscous oil, 190 mg, in quantitative yield.
^1H NMR (400 MHz, $CDCl_3$) δ 4.69 (d, J = 4.4 Hz, 1H), 3.63 (dd, J = 8.4, 4.4 Hz, 1H), 2.30 – 2.17 (m, 1H), 1.14 (d, J = 6.7 Hz, 3H), 1.10 (d, J = 6.7 Hz, 3H).
^{13}C NMR (101 MHz, $CDCl_3$) δ 173.7, 168.1, 69.2, 64.8, 28.0, 20.1, 19.7.
HR-MS, *m/z* (ESI-) calcd for $C_7H_{10}O_4$: 157.0506 $[M-H]^-$, found 157.0507.
The NMR spectra is in agreement with published data.[2]

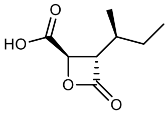

4b

4b was obtained as a viscous oil, 264 mg, in quantitative yield.
^1H NMR (400 MHz, $CDCl_3$) δ 4.71 (d, J = 4.5 Hz, 1H), 3.76 (dd, J = 7.9, 4.5 Hz, 1H), 2.11 – 1.95 (m, 1H), 1.71 – 1.59 (m, 1H), 1.40 – 1.25 (m, 1H), 1.07 (d, J = 6.7 Hz, 3H), 0.95 (t, J = 7.5 Hz, 3H).
^{13}C NMR (101 MHz, $CDCl_3$) δ 173.9, 168.4, 68.8, 63.4, 33.8, 26.9, 16.4, 11.1.
The NMR data is in agreement with published data.[8]
HR-MS, *m/z* (ESI-) calcd for $C_8H_{12}O_4$: 171.0663 $[M-H]^-$, found 171.0668.

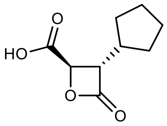

4c

4c was obtained as a viscous light-yellow oil, 45 mg, 60% yield.
^1H NMR (400 MHz, $CDCl_3$) δ 4.67 (d, J = 4.3 Hz, 1H), 3.78 (dd, J = 8.5, 4.2 Hz, 1H), 2.46 – 2.34 (m, 1H), 2.02 – 1.83 (m, 2H), 1.77 – 1.55 (m, 4H), 1.47 – 1.33 (m, 2H).
^{13}C NMR (101 MHz, $CDCl_3$) δ 173.6, 168.5, 70.1, 62.4, 38.3, 30.2, 30.0, 25.2, 25.0.
HR-MS, *m/z* (ESI-) calcd for $C_9H_{12}O_4$: 183.0657 $[M-H]^-$, found 183.0651.

Synthesis of standards for validation of β-lactone assembly pathway

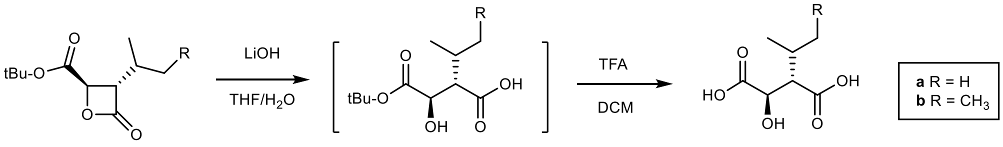

Synthesis of ***1***: To a solution of **S6** (1.4 mmol, 1.0 equiv) in 14 mL THF was added LiOH solution dropwise (2.0 mmol, 1.4 equiv, dissolved in 7 mL of water). The mixture was stirred at room temperature for 4-6 h until reaction complete (judged by TLC). The reaction was diluted with water (40 mL) and concentrated *in vacuo* to remove THF. The resulting aqueous solution was washed with 40 mL EtOAc (this organic layer was discarded), and then adjusted to pH 2 using 1M HCl. The acidified aqueous solution was extracted with EtOAc three times. The organic layers were combined, dried over $MgSO_4$, and concentrated *in vacuo*. The residue was dissolved in 1 mL DCM and cooled to 0 °C. To this solution was added 1 mL of TFA dropwise. The reaction was kept at 0 °C for 16 h before solvent and excess reagent was removed by a stream of nitrogen gas at 0 °C. The residue was dissolved in water and lyophilised to obtain **1**.

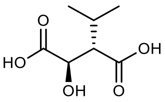

1a

(2*R*,3*S*)-2-hydroxy-3-isopropylsuccinic acid (**1a**) was obtained as a white powder, 201 mg, 82%.
^{1}H NMR (400 MHz, D_2O) δ 4.55 (d, *J* = 4.4 Hz, 1H), 2.64 (dd, *J* = 9.0, 4.4 Hz, 1H), 2.17 – 1.99 (m, 1H), 1.02 (d, *J* = 6.7 Hz, 3H), 0.99 (d, *J* = 6.7 Hz, 3H).
^{13}C NMR (101 MHz, D_2O) δ 176.8, 176.6, 69.3, 55.4, 26.4, 19.9, 19.3.
HR-MS, *m/z* (ESI+) calcd for $C_7H_{12}O_5$: 199.0582 $[M+Na]^+$, found 199.0578.
The NMR spectra is in agreement with published data.[9]

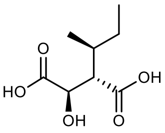

1b

(2*S*,3*R*)-2-((*S*)-*sec*-butyl)-3-hydroxysuccinic acid (**1b**) was a white powder, 72 mg, 89% yield.
^{1}H NMR (400 MHz, D_2O) δ 4.57 (d, *J* = 4.2 Hz, 1H), 2.76 (dd, *J* = 8.9, 4.1 Hz, 1H), 1.98 – 1.86 (m, 1H), 1.55 – 1.43 (m, 1H), 1.29 – 1.16 (m, 1H), 1.01 (d, *J* = 6.8 Hz, 3H), 0.91 (t, *J* = 7.1 Hz, 3H).
^{13}C NMR (101 MHz, D_2O) δ 177.1, 176.8, 69.1, 53.8, 32.7, 26.8, 15.4, 10.4.
The NMR spectra is in agreement with published data.[10]
HR-MS, *m/z* (ESI+) calcd for $C_8H_{14}O_5$: 191.0914 $[M+H]^+$, found 191.0915.

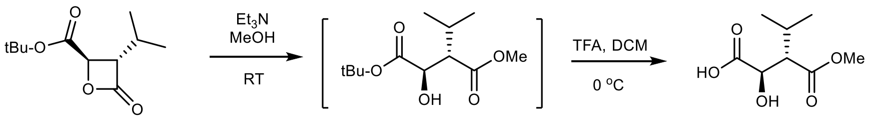

S6a **S7**

*Synthesis of **S7**:* This procedure adapted from literature.[11] To a solution of **S6a** (43 mg, 0.2 mmol, 1.0 equiv) in 2 mL MeOH was added triethyl amine (81 mg, 0.8 mmol, 4.0 equiv). The reaction mixture was stirred at room temperature for 16 h before concentrating *in vacuo*. The residue was dissolved in 0.5 mL DCM and cooled to 0 °C. To this solution was added 0.5 mL of TFA dropwise. The reaction mixture was kept at 0 °C for 16 h. The DCM and TFA was removed by a stream of nitrogen gas at 0 °C and the residue was further dried under high vacuum to obtain 39 mg of **S7** in quantitative yield.
^{1}H NMR (400 MHz, $CDCl_3$) δ 4.44 (d, *J* = 3.1 Hz, 1H), 3.72 (s, 3H), 2.72 (dd, *J* = 8.6, 2.0 Hz, 1H), 2.30 – 2.15 (m, 1H), 1.08 (d, *J* = 6.8 Hz, 3H), 1.00 (d, *J* = 6.8 Hz, 3H).
^{13}C NMR (101 MHz, $CDCl_3$) δ 177.4, 174.9, 69.9, 54.2, 52.3, 28.0, 20.9, 20.5.
HR-MS, *m/z* (ESI-) calcd for $C_8H_{14}O_5$: 189.0763 $[M-H]^-$, found 189.0719.

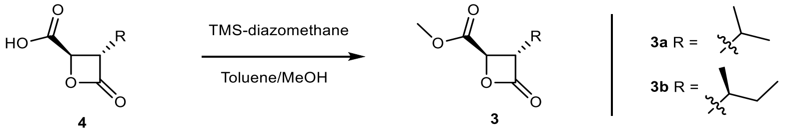

4 **3**

*Synthesis of **3***: To a solution of **4a** (as an example, 0.21 mmol, 1.0 equiv) in 1.25 mL MeOH and 5 mL Toluene (MeOH/Toluene = 1:4, v/v) was added TMS-diazomethane solution (2 M in Hexane, 300 µL, 3.3 equiv) dropwise at RT. The mixture was stirred at RT for 5-10 min until N_2 bubbling ceased and the yellow colour of the solution persists. The reaction was quenched by adding 50 µL acetic acid and the yellow colour of the solution disappear. The solvent was removed *in vacuo* and the crude product was purified by silica gel column (EtOAc/Hexane, 5-10% EtOAc) to obtain **3** as colourless oil.

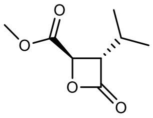

3a

3a was obtained as a colourless oil, 21 mg, 58% yield.
^{1}H NMR (400 MHz, $CDCl_3$) δ 4.64 (d, *J* = 4.4 Hz, 1H), 3.84 (s, 3H), 3.55 (dd, *J* = 8.4, 4.4 Hz, 1H), 2.26 – 2.13 (m, 1H), 1.11 (d, *J* = 6.7 Hz, 3H), 1.07 (d, *J* = 6.8 Hz, 3H).
^{13}C NMR (101 MHz, $CDCl_3$) δ 168.9, 168.4, 69.8, 64.3, 53.1, 27.8, 20.1, 19.6.
HR-MS by ESI was not successful.

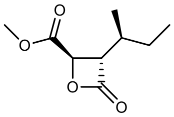

3b

3b was obtained as a colourless oil, 19 mg, 88% yield.
^{1}H NMR (400 MHz, $CDCl_3$) δ 4.66 (d, *J* = 4.4 Hz, 1H), 3.85 (s, 3H), 3.68 (dd, *J* = 7.8, 4.4 Hz, 1H), 2.07 – 1.93 (m, 1H), 1.71 – 1.56 (m, 1H), 1.39 – 1.25 (m, 1H), 1.05 (d, *J* = 6.7 Hz, 3H), 0.94 (t, *J* = 7.5 Hz, 3H).

^{13}C NMR (101 MHz, $CDCl_3$) δ 169.0, 168.8, 69.3, 62.9, 53.1, 33.7, 26.9, 16.4, 11.1.
HR-MS by ESI was not successful.

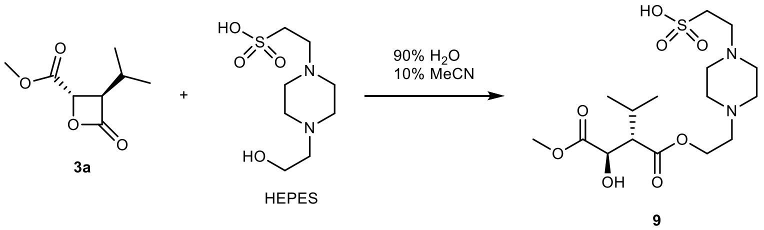

*Synthesis of **9** (buffer adduct)*: A 100 µL solution of **3a** (200 mM, 3.4 mg, 0.02 mmol) in MeCN was mixed with 900 µL HEPES buffer (200 mM, pH 7.0). The mixture was incubated at room temperature for 3 h and then concentrated in vacuo. The residue was purified by preparative HPLC on a C18 column (mobile phase A = H_2O with 0.1% formic acid, mobile phase B = MeCN with 0.1% formic acid, gradient B: 2-50%) to obtain 1.5 mg of **9** as white solid in 37% isolated yield.
^{1}H NMR (400 MHz, D_2O with 0.3% DCl) δ 4.64 (d, *J* = 4.2 Hz, 1H), 4.60 – 4.44 (m, 2H), 3.80 (brs, 8H), 3.77 (s, 3H), 3.73 – 3.63 (m, 4H), 3.39 (dd, *J* = 8.4, 6.3 Hz, 2H), 2.81 (dd, *J* = 8.3, 4.2 Hz, 1H), 2.14 – 2.04 (m, 1H), 0.98 (d, *J* = 6.8 Hz, 3H), 0.95 (d, *J* = 6.7 Hz, 3H).
^{13}C NMR (201 MHz, D_2O with 0.3% DCl) δ 175.7, 173.1, 69.1, 58.3, 55.2, 55.2, 53.0, 52.3, 49.0, 48.9, 44.7, 26.4, 19.8, 19.1.
HR-MS, *m/z* (ESI-) calcd for $C_{16}H_{30}N_2O_8S$: 409.1645 $[M-H]^-$, found 409.1602.

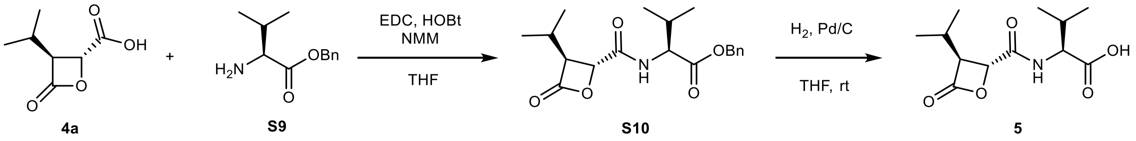

*Synthesis of CysC product **5***: At 0 °C, to a solution of lactone acid **4a** (80 mg, 0.5 mmol, 1.0 equiv) and N-methyl morpholine (NMM, 152 mg, 1.5 mmol, 3.0 equiv) in 10 mL THF was added H-Val-OBn hydrochloride **S9** (122 mg, 0.5 mmol, 1.0 equiv). The solution was stirred at 0 °C for 5 min before EDC·HCl (105 mg, 0.55 mmol, 1.1 equiv), and HOBt·H_2O (74 mg, 0.55 mmol, 1.1 equiv) were quickly added in one portion. The reaction was flushed with nitrogen and the mixture stirred and allowed to gradually reach room temperature for 16 h. The reaction mixture was concentrated *in vacuo*. The residue was taken up with EtOAc (50 mL), washed with 5% (wt) citric acid solution, sat. $NaHCO_3$, brine, dried over $MgSO_4$ and concentrated *in vacuo*. The crude product was purified by silica gel column (EtOAc/Hexane, 5-20% EtOAc) to obtain 95 mg **S10** as colourless oil in 55% yield.
^{1}H NMR (400 MHz, $CDCl_3$) δ 7.42 – 7.29 (m, 5H), 6.73 (d, *J* = 9.0 Hz, 1H), 5.24 (d, *J* = 12.2 Hz, 1H), 5.12 (d, *J* = 12.1 Hz, 1H), 4.62 (d, *J* = 4.5 Hz, 1H), 4.58 (dd, *J* = 8.9, 4.8 Hz, 1H), 3.42 (dd, *J* = 8.2, 4.6 Hz, 1H), 2.35 – 2.10 (m, 2H), 1.09 (dd, *J* = 9.3, 6.7 Hz, 6H), 0.95 (d, *J* = 6.8 Hz, 3H), 0.89 (d, *J* = 7.0 Hz, 3H).
^{13}C NMR (101 MHz, $CDCl_3$) δ 170.9, 168.6, 168.1, 135.2, 128.8, 128.8, 128.6, 71.2, 67.4, 64.6, 57.1, 31.0, 28.0, 20.1, 19.5, 19.2, 17.7.
HR-MS, *m/z* (ESI+) calcd for $C_{19}H_{25}NO_5$: 370.1630 $[M+Na]^+$, found 370.1625.

A solution of **S10** (95 mg, 0.27 mmol) in 5 mL of dry THF was treated with Pd/C (45 mg, 10% wt.). The reaction flask was flushed with hydrogen gas and the mixture stirred under hydrogen atmosphere at room temperature for 16 h. The mixture was diluted with THF and filtered through a celite plug, rinsing with THF. The filtrate was concentrated *in vacuo* to give 60 mg of **5** as white solid (yield 87%).
^{1}H NMR (400 MHz, $CDCl_3$) δ 6.77 (d, *J* = 8.8 Hz, 1H), 4.67 (d, *J* = 4.6 Hz, 1H), 4.57 (dd, *J* = 8.9, 4.8 Hz, 1H), 3.55 (dd, *J* = 8.2, 4.5 Hz, 1H), 2.42 – 2.11 (m, 2H), 1.12 (dd, *J* = 10.3, 6.6 Hz, 6H), 1.01 (d, *J* = 6.8 Hz, 3H), 0.98 (d, *J* = 6.8 Hz, 3H).
^{13}C NMR (101 MHz, $CDCl_3$) δ 175.5, 168.8, 168.7, 71.1, 64.6, 57.1, 30.8, 28.0, 20.1, 19.5, 19.2, 17.7.
HR-MS, *m/z* (ESI) calcd for $C_{12}H_{19}NO_5$: 280.1161 $[M+Na]^+$, found 280.1150.

Synthesis of Cystargolide A (**6a**) and B (**6b**)

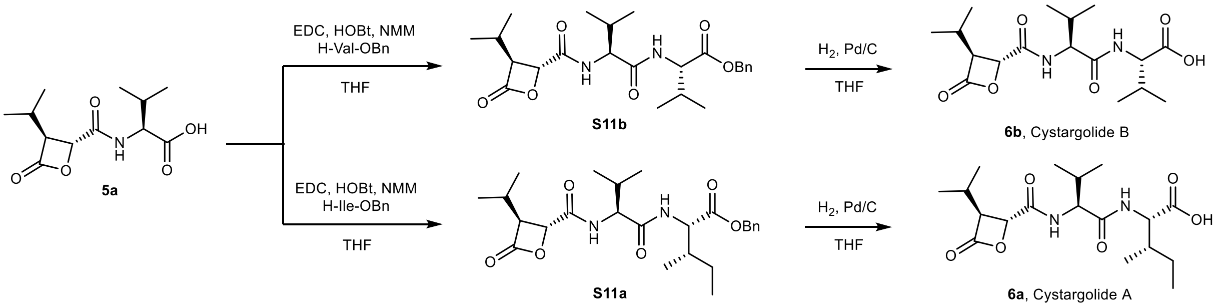

The procedure used for the synthesis of **6a/b** Cystargolide A and B were the same as the synthesis of **5a** mentioned above. Briefly, **5a** was coupled with either H-Val-OBn or H-Ile-OBn to obtain **S11a/b**, which underwent deprotection to obtain **6a/b**.

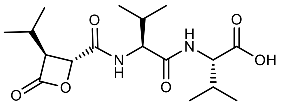

6b
(Cystargolide B)

6b was obtained as white solid, 30 mg, 54% yield over two steps.
^{1}H NMR (400 MHz, DMSO-d6) δ 12.56 (brs, 1H), 8.55 (d, *J* = 9.0 Hz, 1H), 8.14 (d, *J* = 8.2 Hz, 1H), 5.02 (d, *J* = 4.4 Hz, 1H), 4.42 – 4.35 (m, 1H), 4.16 – 4.07 (m, 1H), 3.51 (dd, *J* = 8.3, 4.2 Hz, 1H), 2.19 – 1.99 (m, 3H), 1.00 (d, *J* = 6.7 Hz, 3H), 0.97 (d, *J* = 6.7 Hz, 3H), 0.92 – 0.80 (m, 12H).
^{13}C NMR (101 MHz, DMSO-d6) δ 172.8, 170.8, 170.1, 167.3, 70.1, 62.7, 57.33, 57.26, 30.90, 29.60, 26.68, 19.42, 19.29, 19.09, 18.09, 17.88.
HR-MS, *m/z* (ESI-) calcd for $C_{17}H_{28}N_2O_6$: 355.1875 $[M-H]^-$, found 355.1879.
The NMR spectra is in agreement with published data.[2]

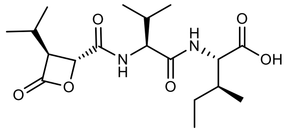

6a
(Cystargolide A)

6a was obtained as white solid, 20 mg, 40% yield over two steps.
^{1}H NMR (400 MHz, MeOD) δ 4.81 (d, *J* = 4.3 Hz, 1H), 4.41 – 4.35 (m, 1H), 4.30 (d, *J* = 7.9 Hz, 1H), 3.56 (dd, *J* = 8.5, 4.4 Hz, 1H), 2.23 – 2.05 (m, 2H), 1.96 – 1.86 (m, 1H), 1.59 – 1.47 (m, 1H), 1.28 (d, *J* = 16.6 Hz, 1H), 1.10 (d, *J* = 6.7 Hz, 3H), 1.06 (d, *J* = 6.7 Hz, 3H), 1.01 – 0.88 (m, 12H).
^{13}C NMR (126 MHz, MeOD) δ 174.6, 173.2, 170.6, 170.4, 72.2, 65.3, 60.2, 58.3, 38.3, 32.0, 28.8, 26.2, 20.4, 19.8, 19.7, 18.8, 16.0, 11.8.
HR-MS, *m/z* (ESI-) calcd for $C_{18}H_{30}N_2O_6$: 369.2031 [M-H]$^-$, found 369.2036.

Synthesis of **7**

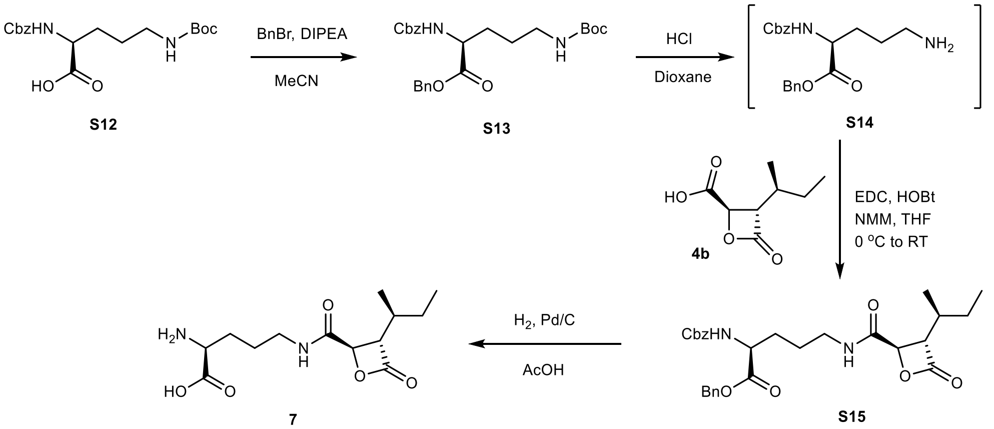

*Synthesis of **S13***: Cbz-Orn-(N-Boc) **S12** (672.8 mg, 2.0 mmol, 1.0 equiv) was dissolved in MeCN (17.5 mL). To this solution was added DIPEA (271.4 mg, 1.05 equiv, 2.1 mmol) and benzyl bromide (359.2 mg, 1.05 equiv, 2.1 mmol). The reaction was stirred at room temperature for 14 h, before solvent was removed *in vacuo*, and the residue was re-dissolved in ethyl acetate (25 mL) and washed with water and brine. The organic layer was dried over $MgSO_4$ and concentrated *in vacuo*. The crude product was purified by silica gel column to obtain 680 mg of **S13** in 75% yield.

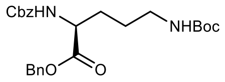

S13

^{1}H NMR (400 MHz, $CDCl_3$) δ 7.41 – 7.28 (m, 10H), 5.37 (d, *J* = 7.1 Hz, 1H), 5.20 – 5.03 (m, 4H), 4.58 – 4.34 (m, 2H), 3.15 – 2.99 (m, 2H), 1.94 – 1.78 (m, 1H), 1.74 – 1.63 (m, 1H), 1.43 (s, 11H).
^{13}C NMR (101 MHz, $CDCl_3$) δ 172.2, 156.0, 136.3, 135.3, 128.8, 128.7, 128.5, 128.3, 128.2, 79.4, 67.4, 67.2, 53.8, 40.1, 30.1, 28.5, 26.1.
The NMR spectra is in agreement with published data.[12]
HR-MS, *m/z* (ESI+) calcd for $C_{25}H_{32}N_2O_6$: 479.2158 [M+Na]$^+$, found 479.2150.

Synthesis of ***S15***: A solution of **S13** (137 mg, 0.3 mmol, 1.0 equiv) in 3 mL 4 M HCl in dioxane was stirred at room temperature for over 3 h. Afterwards, TLC indicated complete removal of Boc protecting group and the reaction was concentrated *in vacuo*. The residue (**S14**) was taken up in 1 mL THF and added to a solution of lactone acid **4b** (52 mg, 0.3 mmol, 1.0 equiv) and N-methyl morpholine (NMM, 91 mg, 0.9 mmol, 3.0 equiv) in 4 mL THF at 0 °C. The solution was stirred at 0 °C for 5 min before EDC·HCl (69 mg, 0.36 mmol, 1.2 equiv), and HOBt·H_2O (49 mg, 0.36 mmol, 1.2 equiv) were quickly added in one portion. The reaction was flushed with nitrogen and the mixture stirred and allowed to gradually reach room temperature for 16 h. The reaction mixture was concentrated *in vacuo* and the residue was taken up with EtOAc (50 mL), washed with 5% (wt) citric acid solution, sat. $NaHCO_3$, brine, dried over $MgSO_4$ and concentrated *in vacuo*. The crude product was purified by silica gel column (EtOAc/Hexane, 20-50% EtOAc) to give 80 mg **S15** as white solid in 52% yield over two steps.

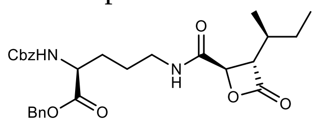

S15

^{1}H NMR (400 MHz, $CDCl_3$) δ 7.45 – 7.28 (m, 10H), 6.38 (s, 1H), 5.38 (d, *J* = 8.9 Hz, 1H), 5.23 – 5.07 (m, 4H), 4.56 (d, *J* = 4.5 Hz, 1H), 4.49 – 4.36 (m, 1H), 3.57 (dd, *J* = 7.6, 4.6 Hz, 1H), 3.37 – 3.21 (m, 2H), 2.03 – 1.83 (m, 2H), 1.76 – 1.62 (m, 2H), 1.57 – 1.42 (m, 2H), 1.33 (d, *J* = 21.2 Hz, 1H), 1.07 (d, *J* = 6.7 Hz, 3H), 0.95 (t, *J* = 7.5 Hz, 3H).
^{13}C NMR (101 MHz, $CDCl_3$) δ 172.0, 169.3, 168.2, 156.0, 136.2, 135.2, 128.8, 128.7, 128.7, 128.5, 128.4, 128.3, 70.8, 67.5, 67.2, 63.0, 53.5, 38.6, 33.9, 30.1, 26.8, 25.3, 16.4, 11.1.
HR-MS, *m/z* (ESI+) calcd for $C_{28}H_{34}N_2O_7$: 533.2264 $[M+Na]^+$, found 533.2261.

Synthesis of ***7***: A solution of **S15** (79 mg, 0.15 mmol) in acetic acid (10 mL) was treated with Pd/C (45 mg, 10% wt.). The reaction was flushed with hydrogen gas and the mixture stirred under a hydrogen atmosphere (balloon) at room temperature for 16 h. The mixture was filtered through a short celite plug, rinsing with acetic acid. The collected filtrate was concentrated *in vacuo* and the residual was purified by Bond Elut™ C18 column (Agilent, eluent H_2O/MeCN, 0-30% MeCN). The fractions containing pure product were combined, concentrated *in vacuo* to remove MeCN and then lyophilised to obtain 28 mg **7** as white powder in 63% yield.

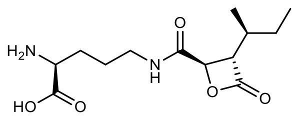

7

^{1}H NMR (400 MHz, D_2O) δ 4.93 (d, *J* = 4.3 Hz, 1H), 3.86 (dd, *J* = 7.5, 4.4 Hz, 1H), 3.76 (t, *J* = 6.1 Hz, 1H), 3.34 (t, *J* = 6.8 Hz, 2H), 2.13 – 1.99 (m, 1H), 1.98 – 1.80 (m, 2H), 1.77 – 1.49 (m, 3H), 1.41 – 1.26 (m, 1H), 1.04 (d, *J* = 6.7 Hz, 3H), 0.92 (t, *J* = 7.5 Hz, 3H).
^{13}C NMR (101 MHz, D_2O) δ 174.3, 172.6, 170.4, 71.0, 62.0, 54.4, 38.6, 32.8, 27.7, 26.2, 24.2, 15.4, 10.3.
HR-MS, *m/z* (ESI+) calcd for $C_{13}H_{22}N_2O_5$: 287.1607 $[M+H]^+$, found 287.1609.

Synthesis of **Ala-Orn** (**S17**) and belactosin C (**8c**)

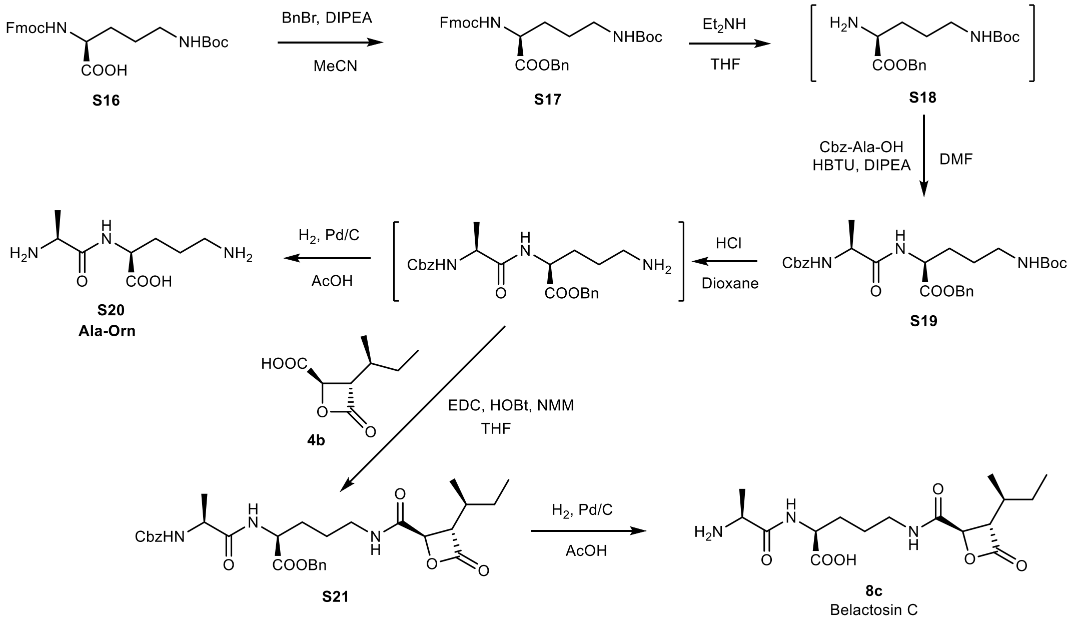

*Synthesis of **S17***: Fmoc-Orn-(N-Boc) **S16** (909 mg, 2.0 mmol, 1.0 equiv) was added to MeCN (17.5 mL). To the mixture was added DIPEA (271.4 mg, 1.05 equiv, 2.1 mmol) and benzyl bromide (359.2 mg, 1.05 equiv, 2.1 mmol). The reaction was stirred at room temperature for 14 h before solvent was removed *in vacuo*, and the residue was taken up by ethyl acetate (25 mL) and washed with 5% wt. citric acid and brine. The organic layer was dried over $MgSO_4$ and concentrated *in vacuo*. The crude product was purified by silica gel column (EtOAc/Hexane, 20-50% EtOAc) to obtain 710 mg of **S17** in 65% yield.

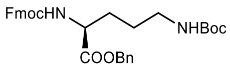

S17

^{1}H NMR (400 MHz, $CDCl_3$) δ 7.77 (d, J = 7.5 Hz, 2H), 7.60 (d, J = 7.6 Hz, 2H), 7.48 – 7.27 (m, 9H), 5.48 (d, J = 7.7 Hz, 1H), 5.26 – 5.10 (m, 2H), 4.60 – 4.32 (m, 4H), 4.21 (t, J = 7.1 Hz, 1H), 3.18 – 2.96 (m, 2H), 1.97 – 1.82 (m, 1H), 1.77 – 1.66 (m, 1H), 1.58 – 1.45 (m, 2H), 1.44 (s, 9H).
^{13}C NMR (101 MHz, $CDCl_3$) δ 172.3, 156.1, 144.0, 143.8, 141.4, 135.3, 128.8, 128.7, 128.5, 127.8, 127.2, 125.2, 120.1, 79.4, 67.4, 67.1, 53.8, 47.3, 40.0, 30.0, 28.5, 26.2.
HR-MS, *m/z* (ESI+) calcd for $C_{32}H_{36}N_2O_6$: 567.2471 $[M+Na]^+$, found 567.2465.

*Synthesis of **S19***: A solution of **S17** (700 mg, 1.3 mmol, 1.0 equiv) in 2.5 mL THF was treated with 2.5 mL diethylamine. The reaction was stirred at room temperature for 1 h before another 1 mL diethylamine was added. The mixture was stirred at room temperature for additional 2 h before concentrating *in vacuo*. The residue was azeotroped with toluene (2 × 2 mL) under reduced pressure, and the crude product was taken up in DMF (2 mL). To this solution was added Cbz-Alanine (290 mg, 1.3 mmol, 1.0 equiv), HBTU (606 mg, 1.6 mmol, 1.2 equiv) and DIPEA (504 mg, 3.9 mmol, 3.0 equiv). The reaction mixture was stirred at room temperature for 16 h before diluting with DCM (50 mL). The resulting solution was washed with 5% wt. citric acid, brine,

dried over $MgSO_4$ and concentrated *in vacuo*. The crude product was purified by silica gel column (EtOAc/Hexane, 20-50% EtOAc) to give 531 mg **S19** as white solid in 78% yield over two steps.

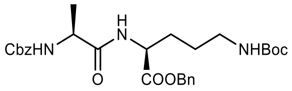

S19

^{1}H NMR (400 MHz, $CDCl_3$) δ 7.39 – 7.27 (m, 10H), 6.90 (s, 1H), 5.50 (s, 1H), 5.23 – 5.02 (m, 4H), 4.70 – 4.53 (m, 2H), 4.34 – 4.22 (m, 1H), 3.14 – 2.96 (m, 2H), 1.96 – 1.74 (m, 2H), 1.73 – 1.60 (m, 1H), 1.55 – 1.42 (m, 2H), 1.41 (s, 9H), 1.37 (d, *J* = 7.0 Hz, 3H).
^{13}C NMR (101 MHz, $CDCl_3$) δ 172.4, 171.9, 156.3, 156.1, 136.4, 135.3, 128.8, 128.6, 128.5, 128.3, 128.2, 79.5, 67.4, 67.1, 52.3, 50.6, 39.9, 29.0, 28.5, 26.2, 18.6.
HR-MS, *m/z* (ESI+) calcd for $C_{28}H_{37}N_3O_7$: 550.2529 $[M+Na]^+$, found 550.2523.

*Synthesis of Ala-Orn (**S20**)*: A solution of **S19** (200 mg, 0.38 mmol, 1.0 equiv) in 5 mL 4 M HCl (in dioxane) was stirred at room temperature for over 3 h. After TLC indicated complete removal of Boc protecting group the reaction was concentrated *in vacuo*. The residue was taken up in acetic acid (5 mL). To this solution was treated with Pd/C (60 mg, 10% wt.). The reaction was flushed with hydrogen gas and the mixture stirred under a hydrogen atmosphere (balloon) at room temperature for 16 h. The mixture was filtered through a short celite plug, rinsing with acetic acid. The collected filtrate was concentrated *in vacuo* and the residual was purified by Bond Elut™ C18 column (Agilent, eluent H_2O/MeCN with 0.1% HCl, 0-30% MeCN). The fractions containing pure product were combined, concentrated to remove MeCN and then lyophilised to obtain 80 mg Ala-Orn (**S20**) di-hydrochloride salt as yellow solid in 76% yield.

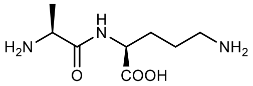

S20

^{1}H NMR (400 MHz, D_2O) δ 4.45 (dd, *J* = 8.4, 5.3 Hz, 1H), 4.15 (q, *J* = 7.0 Hz, 1H), 3.05 (t, *J* = 7.5 Hz, 2H), 2.07 – 1.95 (m, 1H), 1.91 – 1.71 (m, 3H), 1.57 (d, *J* = 7.2 Hz, 3H).
^{13}C NMR (101 MHz, D_2O) δ 174.7, 170.9, 52.4, 48.9, 38.8, 27.3, 23.2, 16.4.
HR-MS, *m/z* (ESI+) calcd for $C_8H_{17}N_3O_3$: 202.1197 $[M-H]^-$, found 202.1204.

*Synthesis of **S21**:* A solution of **S19** (158 mg, 0.3 mmol, 1.0 equiv) in 3 mL 4M HCl (in dioxane) was stirred at room temperature for over 3 h. After TLC indicated complete removal of Boc protecting group the reaction was concentrated *in vacuo*. The residue was taken up in 1 mL THF and added to a solution of lactone acid **4b** (52 mg, 0.3 mmol, 1.0 equiv) and N-methyl morpholine (NMM, 91 mg, 0.9 mmol, 3.0 equiv) in 4 mL THF at 0 °C. The solution was stirred at 0 °C for 5 min before EDC·HCl (69 mg, 0.36 mmol, 1.2 equiv), and HOBt·H_2O (50 mg, 0.36 mmol, 1.2 equiv) were quickly added in one portion. The reaction was flushed with nitrogen and the mixture stirred and allowed to gradually reach room temperature for 16 h. The reaction mixture was concentrated *in vacuo* and the residue was taken up with EtOAc (50 mL), washed with 5% (wt) citric acid solution, sat. $NaHCO_3$, brine, dried over $MgSO_4$ and concentrated *in vacuo*. The crude product was purified by silica gel column (EtOAc/Hexane, 50-60% EtOAc) to give 85 mg **S21** as white solid in 49% yield over two steps.

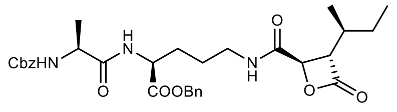

S21

^{1}H NMR (400 MHz, $CDCl_3$) δ 7.45 – 7.25 (m, 10H), 6.76 (d, *J* = 8.2 Hz, 1H), 6.58 (s, 1H), 5.41 (d, *J* = 6.8 Hz, 1H), 5.25 – 5.03 (m, 4H), 4.67 – 4.57 (m, 1H), 4.54 (d, *J* = 4.5 Hz, 1H), 4.32 – 4.18 (m, 1H), 3.56 (dd, *J* = 7.7, 4.5 Hz, 1H), 3.34 – 3.13 (m, 2H), 2.03 – 1.81 (m, 2H), 1.78 – 1.58 (m, 4H), 1.57 – 1.41 (m, 2H), 1.37 (d, *J* = 7.1 Hz, 3H), 1.34 – 1.25 (m, 1H), 1.05 (d, *J* = 6.7 Hz, 3H), 0.93 (t, *J* = 7.4 Hz, 3H).
^{13}C NMR (101 MHz, $CDCl_3$) δ 172.7, 171.7, 169.4, 168.3, 156.2, 136.2, 135.2, 128.8, 128.7, 128.6, 128.4, 128.3, 128.1, 70.9, 67.4, 67.2, 62.9, 51.9, 50.5, 38.5, 33.8, 29.3, 26.7, 25.4, 18.7, 16.4, 11.1.
HR-MS, *m/z* (ESI) calcd for $C_{31}H_{39}N_3O_8$: 604.2635 $[M+Na]^+$, found 604.2610.
The NMR spectra is in agreement with published data[8].

*Synthesis of **8c**:* A solution of **S21** (80 mg, 0.14 mmol) in acetic acid (10 mL) was treated with Pd/C (45 mg, 10% wt.). The reaction was flushed with hydrogen gas and the mixture stirred under a hydrogen atmosphere (balloon) at room temperature for 16 h. The mixture was filtered through a short celite plug, rinsing with acetic acid. The collected filtrate was concentrated *in vacuo* and the residual was purified by Bond Elut™ C18 column (Agilent, eluent H_2O/MeCN, 0-30% MeCN). The fractions containing pure product were combined, concentrated to remove MeCN and then lyophilised to obtain 26 mg Belactosin C **(8c)** as white powder in 53% yield.

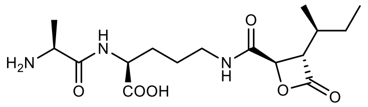

8c
(Belactosin C)

^{1}H NMR (400 MHz, D_2O) δ 4.92 (d, *J* = 4.4 Hz, 1H), 4.17 (dd, *J* = 8.1, 5.2 Hz, 1H), 4.11 (q, *J* = 7.1 Hz, 1H), 3.85 (dd, *J* = 7.5, 4.4 Hz, 1H), 3.31 (t, *J* = 6.9 Hz, 2H), 2.13 – 1.98 (m, 1H), 1.89 – 1.78 (m, 1H), 1.78 – 1.67 (m, 1H), 1.66 – 1.49 (m, 6H), 1.40 – 1.28 (m, 1H), 1.03 (d, *J* = 6.7 Hz, 3H), 0.91 (t, *J* = 7.5 Hz, 3H).
^{13}C NMR (101 MHz, D_2O) δ 178.1, 172.7, 170.2, 170.0, 71.0, 62.0, 55.1, 49.0, 38.8, 32.8, 28.6, 26.2, 24.9, 16.4, 15.4, 10.3.
HR-MS, *m/z* (ESI) calcd for $C_{16}H_{27}N_3O_6$: 358.1978 $[M+H]^+$, found 358.1985.
The NMR spectra is in agreement with published data[13].

Synthesis of Cystargolide analogues

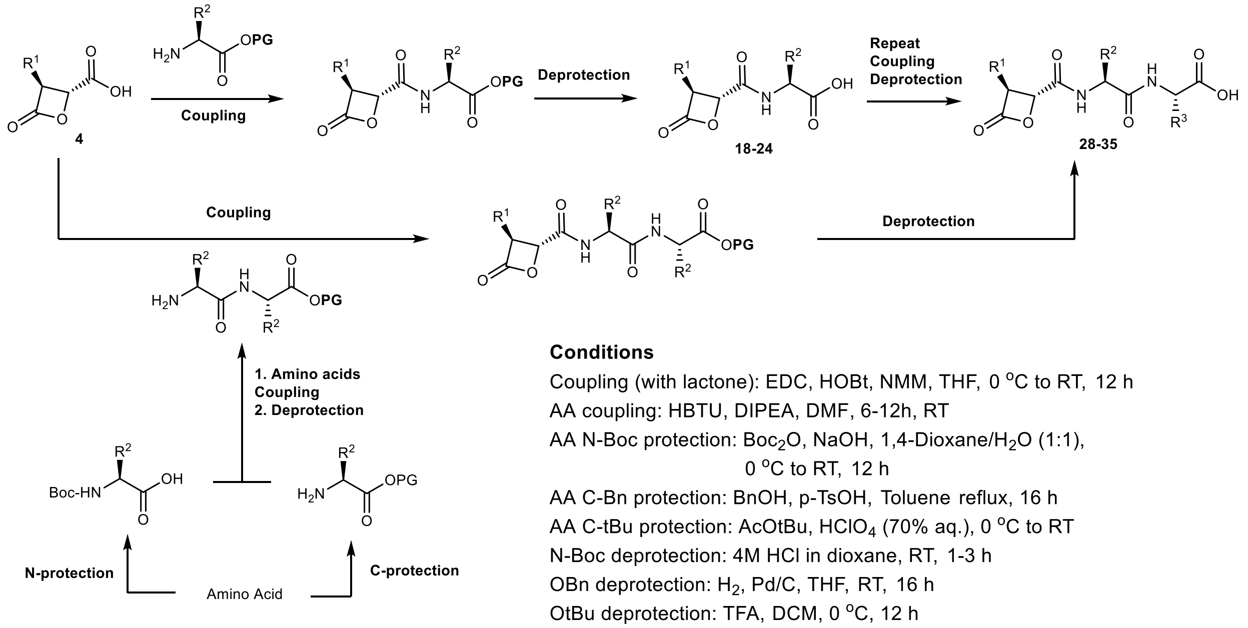

General procedure A: Bn Protection of amino acids. To a solution of amino acid (**AA**) (2.1 mmol, 1.0 equiv) in 5 mL Toluene was added TsOH monohydrate (590 mg, 3.1 mmol, 1.5 equiv) and BnOH (2.2 mL, 21 mmol, 10.0 equiv). The mixture was stirred and heated under reflux for 18 h before diluting with 50 mL water and washed with Et_2O. The aqueous layer was basified with saturated aqueous $NaHCO_3$ and extracted with EtOAc (3 times). The organic layers were combined, dried over $MgSO_4$ and concentrated *in vacuo* to afford the crude **H-AA-OBn**, which was used in the coupling step without further purification.

General procedure B: tBu Protection of amino acids. To a solution of amino acid (**AA**) (0.88 mmol) in AcO*t*Bu (2.2 mL), 70% aqueous $HClO_4$ (2.64 mmol, 3.0 equiv) was added dropwise at 0 °C. After completion of the addition of $HClO_4$, the reaction mixture was stirred for 12 h at room temperature, and then quenched with 1 M aqueous HCl. The aqueous layer was diluted with H_2O and washed with Et_2O. Then the aqueous layer was basified with saturated aqueous $NaHCO_3$ (pH 9) and extracted with DCM (3 times). The organic layer was dried over $MgSO_4$ and concentrated to afford the crude **H-AA-O*t*Bu**, which was used in the next reaction without further purification.

General procedure C: N-Boc protection of amino acids. At 0 °C, to a solution of amino acid (**AA**, 4.0 mmol) and NaOH (6.0 mmol, 1.5 equiv) in H_2O/1,4-dioxane (H_2O/dioxane = 1:1, 30 mL) was added Boc_2O (6.0 mmol dissolved in 8 mL 1,4-dioxane). The reaction was then allowed to reach room temperature and kept stirring for 12 h. The mixture was diluted with water and washed with EtOAc. The aqueous layer was adjusted to pH 2-3 with 5% citric acid, extracted with EtOAc, dried and concentrated *in vacuo* to give **Boc-AA-OH**.

General procedure D: coupling of protected amino acids. To a solution of **Boc-AA-OH** (1.0 mmol) and C-protected amino acid (**H-AA-OBn/O*t*Bu**, 1.0 mmol) in 2.5 mL DMF was added

HBTU (1.0 mmol) and DIPEA (2.0 mmol). After stirring at RT for 12 h, the reaction was diluted with EtOAc, washed with sat. $NaHCO_3$, 1 M HCl, brine, dried over $MgSO_4$, and concentrated *in vacuo* to give protected dipeptide.

The coupling of β-lactone containing acid with C-protected amino acids or dipeptides followed the same procedure as the synthesis of CysC product **5** (using EDC, HOBt, NMM, THF). The crude product was deprotected using either H_2, Pd/C (in the case of OBn protection, following the same procedure as described in the synthesis of **5**), or TFA/DCM (in the case of O*t*Bu protection, following the same procedure as described in the synthesis of **4**).

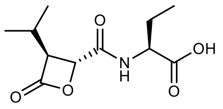

18

18 was prepared as a white solid, 22.3 mg, 29% yield over two steps.
^{1}H NMR (400 MHz, MeOD) δ 4.78 (d, *J* = 4.3 Hz, 1H), 4.42 – 4.32 (m, 1H), 3.58 (dd, *J* = 8.4, 4.3 Hz, 1H), 2.24 – 2.11 (m, 1H), 2.03 – 1.89 (m, 1H), 1.85 – 1.70 (m, 1H), 1.10 (d, *J* = 6.7 Hz, 3H), 1.07 (d, *J* = 6.7 Hz, 3H), 0.98 (t, *J* = 7.4 Hz, 3H).
^{13}C NMR (101 MHz, MeOD) δ 174.7, 170.7, 170.7, 72.2, 65.2, 55.0, 28.8, 25.5, 20.4, 19.8, 10.6.
HR-MS, *m/z* (ESI-) calcd for $C_{11}H_{17}NO_5$: 242.1034 [M-H]$^-$, found 242.1082.

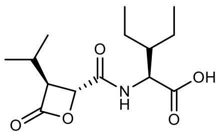

21

21 was prepared as a white solid, 18.2 mg, 32% yield over two steps.
^{1}H NMR (400 MHz, MeOD) δ 4.85 (d, *J* = 4.4 Hz, 1H), 4.66 (d, *J* = 4.6 Hz, 1H), 3.55 (dd, *J* = 8.5, 4.3 Hz, 1H), 2.24 – 2.12 (m, 1H), 1.88 – 1.78 (m, 1H), 1.52 – 1.40 (m, 2H), 1.34 – 1.24 (m, 2H), 1.08 (dd, *J* = 16.5, 6.7 Hz, 6H), 1.01 – 0.87 (m, 6H).
^{13}C NMR (101 MHz, MeOD) δ 169.3, 169.2, 70.6, 63.9, 53.7, 43.6, 27.4, 22.8, 22.4, 22.1, 19.0, 18.5, 10.6, 10.5.
HR-MS, *m/z* (ESI-) calcd for $C_{14}H_{23}NO_5$: 284.1503 [M-H]$^-$, found 284.1569.

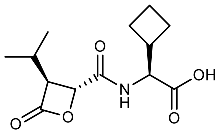

19

19 was prepared as a white solid, 30 mg, 35% yield.
^{1}H NMR (400 MHz, MeOD) δ 4.79 (d, *J* = 4.3 Hz, 1H), 4.40 (d, *J* = 9.1 Hz, 1H), 3.57 (dd, *J* = 8.4, 4.4 Hz, 1H), 2.90 – 2.58 (m, 1H), 2.24 – 2.13 (m, 1H), 2.12 – 1.78 (m, 6H), 1.10 (d, *J* = 6.7 Hz, 3H), 1.06 (d, *J* = 6.7 Hz, 3H).
^{13}C NMR (101 MHz, MeOD) δ 174.0, 170.8, 170.7, 72.1, 65.3, 57.9, 38.1, 28.8, 26.7, 26.2, 20.4, 19.8, 18.7.
HR-MS, *m/z* (ESI-) calcd for $C_{13}H_{19}NO_5$: 268.1190 [M-H]$^-$, found 268.1187.

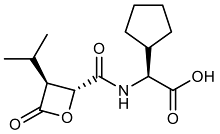

22

22 was prepared as a white solid, 32 mg, 38% yield.
^{1}H NMR (400 MHz, MeOD) δ 4.80 (d, *J* = 4.3 Hz, 1H), 4.39 – 4.32 (m, 1H), 3.56 (dd, *J* = 8.5, 4.3 Hz, 1H), 2.41 – 2.29 (m, 1H), 2.24 – 2.12 (m, 1H), 1.84 – 1.73 (m, 2H), 1.71 – 1.53 (m, 4H), 1.47 – 1.31 (m, 2H), 1.10 (d, *J* = 6.7 Hz, 3H), 1.06 (d, *J* = 6.7 Hz, 3H).
^{13}C NMR (101 MHz, MeOD) δ 174.6, 170.6, 170.5, 72.1, 65.2, 57.3, 42.9, 30.4, 30.0, 28.8, 26.3, 25.9, 20.4, 19.8.
HR-MS, *m/z* (ESI-) calcd for $C_{14}H_{21}NO_5$: 282.1347 [M-H]$^-$, found 282.1393.

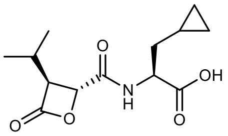

20

20 was prepared as a white solid, 10 mg, 13% yield.
^{1}H NMR (400 MHz, MeOD) δ 4.79 (d, *J* = 4.4 Hz, 1H), 4.53 (dd, *J* = 8.1, 5.5 Hz, 1H), 3.59 (dd, *J* = 8.4, 4.4 Hz, 1H), 2.28 – 2.08 (m, 1H), 1.89 – 1.63 (m, 2H), 1.11 (d, *J* = 6.7 Hz, 3H), 1.07 (d, *J* = 6.7 Hz, 3H), 0.89 – 0.68 (m, 1H), 0.59 – 0.35 (m, 2H), 0.26 – 0.02 (m, 2H).
^{13}C NMR (101 MHz, MeOD) δ 174.7, 170.7, 170.6, 72.3, 65.2, 54.2, 37.2, 28.9, 20.4, 19.8, 8.6, 5.2, 4.5.
HR-MS, *m/z* (ESI-) calcd for $C_{13}H_{19}NO_5$: 268.1185 [M-H]$^-$, found 268.1166.

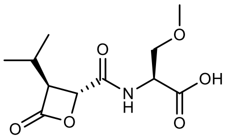

23

23 was prepared as a white solid, 3 mg, 5% yield.
^{1}H NMR (400 MHz, MeOD) δ 4.82 (d, *J* = 4.4 Hz, 1H), 4.65 (dd, *J* = 5.2, 3.6 Hz, 1H), 3.83 (dd, *J* = 9.8, 5.2 Hz, 1H), 3.70 (dd, *J* = 9.8, 3.6 Hz, 1H), 3.60 (dd, *J* = 8.3, 4.4 Hz, 1H), 3.36 (s, 3H), 2.25 – 2.10 (m, 1H), 1.10 (d, *J* = 6.7 Hz, 3H), 1.07 (d, *J* = 6.8 Hz, 3H).
^{13}C NMR (126 MHz, MeOD) δ 172.6, 170.6, 170.6, 72.6, 72.2, 65.3, 59.3, 53.9, 28.9, 20.4, 19.8.
HR-MS, *m/z* (ESI-) calcd for $C_{11}H_{17}NO_6$: 258.0978 [M-H]$^-$, found 258.0959.

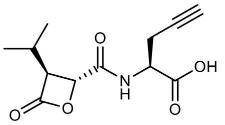

24

24 was prepared as a white solid, 35 mg, 42% yield.
^{1}H NMR (400 MHz, MeOD) δ 4.82 (d, *J* = 4.4 Hz, 1H), 4.62 (dd, *J* = 7.6, 5.0 Hz, 1H), 3.59 (dd, *J* = 8.4, 4.4 Hz, 1H), 2.88 – 2.67 (m, 2H), 2.39 (t, *J* = 2.6 Hz, 1H), 2.26 – 2.12 (m, 1H), 1.11 (d, *J* = 6.7 Hz, 3H), 1.08 (d, *J* = 6.7 Hz, 3H).
^{13}C NMR (101 MHz, MeOD) δ 172.7, 170.6, 79.9, 72.3, 72.1, 65.4, 52.3, 28.9, 22.0, 20.4, 19.8.

HR-MS, *m/z* (ESI-) calcd for $C_{12}H_{15}NO_5$: 252.0877 [M-H]$^-$, found 252.0889.

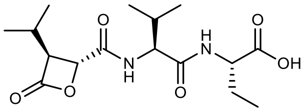

28

28 was prepared as a light-yellow oil, 15.8 mg, 24% yield over two steps.
^{1}H NMR (400 MHz, MeOD) δ 4.81 (d, *J* = 4.3 Hz, 1H), 4.36 – 4.26 (m, 2H), 3.57 (dd, *J* = 8.5, 4.3 Hz, 1H), 2.22 – 2.07 (m, 2H), 1.93 – 1.86 (m, 1H), 1.78 – 1.69 (m, 1H), 1.10 (d, *J* = 6.7 Hz, 3H), 1.06 (d, *J* = 6.7 Hz, 3H), 1.03 – 0.93 (m, 9H).
^{13}C NMR (101 MHz, MeOD) δ 173.6, 171.8, 169.2, 169.0, 70.8, 63.8, 58.7, 53.7, 30.7, 27.4, 24.5, 19.0, 18.4, 18.3, 17.4, 9.2.
HR-MS, *m/z* (ESI-) calcd for $C_{16}H_{26}N_2O_6$: 341.1718 [M-H]$^-$, found 341.1788.

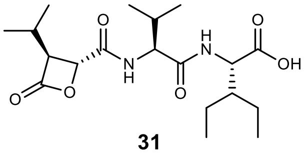

31

31 was prepared as a white solid, 20 mg, 35% yield.
^{1}H NMR (400 MHz, MeOD) δ 4.80 (d, *J* = 4.4 Hz, 1H), 4.61 (d, *J* = 4.8 Hz, 1H), 4.30 (d, *J* = 8.0 Hz, 1H), 3.54 (dd, *J* = 8.5, 4.3 Hz, 1H), 2.23 – 2.03 (m, 2H), 1.81 – 1.68 (m, 1H), 1.50 – 1.26 (m, 4H), 1.10 (d, *J* = 6.7 Hz, 3H), 1.06 (d, *J* = 6.7 Hz, 3H), 1.03 – 0.86 (m, 12H).
^{13}C NMR (101 MHz, MeOD) δ 175.1, 173.6, 170.6, 170.4, 72.2, 65.3, 60.2, 55.2, 45.0, 31.8, 28.8, 23.6, 23.4, 20.4, 19.9, 19.7, 18.8, 12.0, 12.0.
HR-MS, *m/z* (ESI-) calcd for $C_{19}H_{32}N_2O_6$: 383.2182 [M-H]$^-$, found 383.2167.

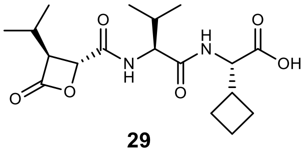

29

29 was prepared as a white solid, 30 mg, 53% yield.
^{1}H NMR (400 MHz, MeOD) δ 4.81 (d, *J* = 4.4 Hz, 1H), 4.34 (d, *J* = 8.6 Hz, 1H), 4.30 (d, *J* = 7.7 Hz, 1H), 3.56 (dd, *J* = 8.4, 4.4 Hz, 1H), 2.82 – 2.63 (m, 1H), 2.23 – 1.78 (m, 8H), 1.10 (d, *J* = 6.7 Hz, 3H), 1.06 (d, *J* = 6.7 Hz, 3H), 1.00 (d, *J* = 6.8 Hz, 3H), 0.96 (d, *J* = 6.7 Hz, 3H).
^{13}C NMR (101 MHz, MeOD) δ 174.4, 173.3, 170.6, 170.4, 72.2, 65.2, 60.1, 58.0, 38.4, 32.0, 28.8, 26.3, 26.1, 20.4, 19.8, 19.7, 18.7, 18.7.
HR-MS, *m/z* (ESI-) calcd for $C_{18}H_{28}N_2O_6$: 367.1875 [M-H]$^-$, found 367.1869.

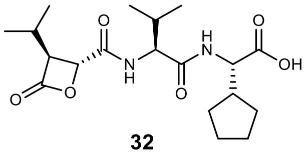

32

32 was prepared as a white solid, 30 mg, 44% yield.
^{1}H NMR (400 MHz, MeOD) δ 4.80 (d, *J* = 4.4 Hz, 1H), 4.30 (dd, *J* = 9.7, 7.9 Hz, 2H), 3.55 (dd, *J* = 8.5, 4.3 Hz, 1H), 2.35 – 2.24 (m, 1H), 2.21 – 2.04 (m, 2H), 1.82 – 1.71 (m, 2H), 1.70 – 1.62

(m, 2H), 1.61 – 1.52 (m, 2H), 1.46 – 1.31 (m, 2H), 1.10 (d, *J* = 6.7 Hz, 3H), 1.06 (d, *J* = 6.7 Hz, 3H), 0.99 (d, *J* = 6.7 Hz, 3H), 0.95 (d, *J* = 6.7 Hz, 3H).
^{13}C NMR (101 MHz, MeOD) δ 174.9, 173.2, 170.6, 170.4, 72.2, 65.3, 60.1, 57.2, 43.0, 32.0, 30.2, 29.9, 28.8, 26.3, 26.0, 20.4, 19.8, 19.7, 18.8.
HR-MS, *m/z* (ESI-) calcd for $C_{19}H_{30}N_2O_6$: 381.2026 $[M-H]^-$, found 381.2081.

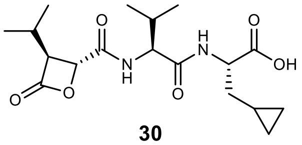

30

30 was prepared as a white solid, 30 mg, 42% yield over two steps.
^{1}H NMR (400 MHz, MeOD) δ 4.81 (d, *J* = 4.3 Hz, 1H), 4.51 – 4.44 (m, 1H), 4.30 (d, *J* = 7.7 Hz, 1H), 3.56 (dd, *J* = 8.4, 4.4 Hz, 1H), 2.24 – 2.06 (m, 2H), 1.78 – 1.61 (m, 2H), 1.10 (d, *J* = 6.7 Hz, 3H), 1.06 (d, *J* = 6.7 Hz, 3H), 1.01 (d, *J* = 6.7 Hz, 3H), 0.96 (d, *J* = 6.8 Hz, 3H), 0.88 – 0.77 (m, 1H), 0.53 – 0.40 (m, 2H), 0.21 – 0.05 (m, 2H).
^{13}C NMR (101 MHz, MeOD) δ 175.0, 173.0, 170.6, 170.4, 72.2, 65.2, 60.1, 54.2, 37.7, 32.1, 28.8, 20.4, 19.8, 19.7, 18.8, 8.6, 5.2, 4.7.
HR-MS, *m/z* (ESI-) calcd for $C_{18}H_{28}N_2O_6$: 367.1869 $[M-H]^-$, found 367.1845.

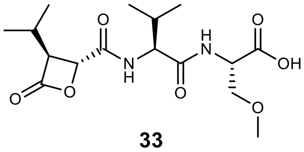

33

33 was prepared as a white solid, 5 mg, 9% yield.
^{1}H NMR (400 MHz, MeOD) δ 4.82 (d, *J* = 4.4 Hz, 1H), 4.52 (s, 1H), 4.35 (d, *J* = 7.2 Hz, 1H), 3.79 (dd, *J* = 9.7, 4.5 Hz, 1H), 3.65 (dd, *J* = 9.7, 3.0 Hz, 1H), 3.60 (dd, *J* = 8.5, 4.3 Hz, 1H), 3.34 (s, 3H), 2.23 – 2.09 (m, 2H), 1.11 (d, *J* = 6.7 Hz, 3H), 1.07 (d, *J* = 6.7 Hz, 3H), 1.00 (d, *J* = 6.8 Hz, 3H), 0.96 (d, *J* = 6.8 Hz, 3H).
^{13}C NMR (126 MHz, MeOD) δ 172.8, 170.6, 170.5, 73.3, 72.2, 65.2, 60.0, 59.3, 32.1, 28.9, 20.4, 19.8, 19.7, 18.6.
HR-MS, *m/z* (ESI-) calcd for $C_{16}H_{26}N_2O_7$: 357.1662 $[M-H]^-$, found 357.1506.

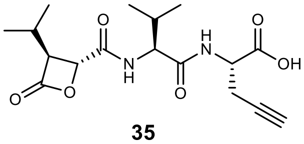

35

35 was prepared as a white solid, 25 mg, 32% yield.
^{1}H NMR (400 MHz, MeOD) δ 8.47 (d, *J* = 7.8 Hz, 1H), 8.19 (d, *J* = 8.7 Hz, 1H), 4.82 (d, *J* = 4.4 Hz, 1H), 4.59 – 4.49 (m, 1H), 4.39 – 4.30 (m, 1H), 3.59 (dd, *J* = 8.5, 4.3 Hz, 1H), 2.80 – 2.65 (m, 2H), 2.34 (s, 1H), 2.24 – 2.09 (m, 2H), 1.10 (d, *J* = 6.7 Hz, 3H), 1.07 (d, *J* = 6.8 Hz, 3H), 1.00 (d, *J* = 6.7 Hz, 3H), 0.96 (d, *J* = 6.7 Hz, 3H).
^{13}C NMR (101 MHz, MeOD) δ 173.0, 170.6, 170.5, 80.1, 72.2, 72.2, 65.3, 60.0, 52.6, 32.2, 28.9, 22.3, 20.4, 19.8, 19.7, 18.6.
HR-MS, *m/z* (ESI-) calcd for $C_{17}H_{24}N_2O_6$: 351.1562 $[M-H]^-$, found 351.1553.

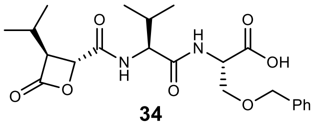

34

34 was prepared as a white solid, 20 mg, 23% yield.

^{1}H NMR (400 MHz, MeOD) δ 7.39 – 7.21 (m, 5H), 4.81 (d, *J* = 4.4 Hz, 1H), 4.67 (t, *J* = 4.4 Hz, 1H), 4.59 – 4.49 (m, 2H), 4.42 – 4.31 (m, 1H), 3.89 (dd, *J* = 9.9, 5.0 Hz, 1H), 3.75 (dd, *J* = 9.9, 3.6 Hz, 1H), 3.57 (dd, *J* = 8.6, 4.4 Hz, 1H), 2.25 – 2.05 (m, 2H), 1.09 (d, *J* = 6.6 Hz, 3H), 1.06 (d, *J* = 6.8 Hz, 3H), 1.00 (d, *J* = 6.8 Hz, 3H), 0.96 (d, *J* = 6.8 Hz, 3H).

^{13}C NMR (101 MHz, MeOD) δ 171.6, 171.4, 169.2, 169.0, 137.8, 128.0, 127.4, 127.3, 72.8, 70.8, 69.2, 63.8, 58.5, 52.7, 48.2, 48.0, 47.8, 47.6, 47.4, 47.2, 47.0, 30.8, 27.4, 19.0, 18.4, 18.3, 17.2.

HR-MS, *m/z* (ESI-) calcd for $C_{22}H_{30}N_2O_7$: 433.1975 [M-H]$^-$, found 433.1964.

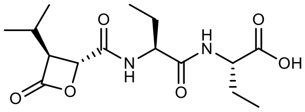

11

11 was prepared as an oil, 39.3 mg, 43% yield over three steps.

^{1}H NMR (400 MHz, MeOD) δ 4.77 (d, *J* = 4.4 Hz, 1H), 4.41 – 4.25 (m, 2H), 3.61 (dd, *J* = 8.4, 4.3 Hz, 1H), 2.24 – 2.09 (m, 1H), 1.93 – 1.85 (m, 2H), 1.81 – 1.66 (m, 2H), 1.10 (d, *J* = 6.7 Hz, 3H), 1.06 (d, *J* = 6.7 Hz, 3H), 0.98 (t, *J* = 7.5 Hz, 6H).

^{13}C NMR (101 MHz, MeOD) δ 175.0, 173.8, 170.6, 170.5, 72.2, 65.2, 56.0, 55.0, 28.8, 26.4, 25.9, 24.0, 20.4, 19.8, 10.6.

HR-MS, *m/z* (ESI-) calcd for $C_{15}H_{24}N_2O_6$: 327.1562 [M-H]$^-$, found 327.1583.

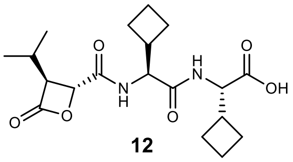

12

12 was prepared as a white solid, 18.2 mg, 32% yield over three steps.

^{1}H NMR (400 MHz, MeOD) δ 4.78 (d, *J* = 4.4 Hz, 1H), 4.44 (d, *J* = 9.3 Hz, 1H), 4.34 (d, *J* = 8.4 Hz, 1H), 3.58 (dd, *J* = 8.4, 4.4 Hz, 1H), 2.78 – 2.64 (m, 2H), 2.24 – 2.10 (m, 1H), 2.09 – 1.76 (m, 12H), 1.07 (dd, *J* = 16.3, 6.7 Hz, 6H).

^{13}C NMR (101 MHz, MeOD) δ 172.8, 171.4, 169.2, 169.2, 70.8, 63.9, 57.6, 56.3, 37.2, 37.1, 27.4, 25.0, 24.8, 24.5, 24.5, 19.0, 18.4, 17.3, 17.3.

HR-MS, *m/z* (ESI-) calcd for $C_{19}H_{28}N_2O_6$: 379.1875 [M-H]$^-$, found 379.1908.

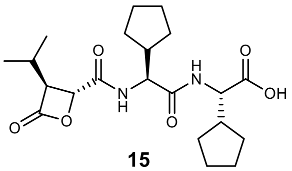

15

15 was prepared as a white solid, 5 mg, 8% yield over three steps.

^{1}H NMR (400 MHz, MeOD) δ 4.77 (d, *J* = 4.4 Hz, 1H), 4.31 (d, *J* = 7.6 Hz, 1H), 4.28 (d, *J* = 9.6 Hz, 1H), 3.56 (dd, *J* = 8.5, 4.4 Hz, 1H), 2.35 – 2.23 (m, 2H), 2.22 – 2.11 (m, 1H), 1.84 – 1.50 (m, 13H), 1.47 – 1.33 (m, 4H), 1.10 (d, *J* = 6.7 Hz, 3H), 1.06 (d, *J* = 6.7 Hz, 3H).

^{13}C NMR (126 MHz, MeOD) δ 173.4, 170.6, 170.3, 72.2, 65.3, 59.0, 57.4, 43.4, 43.2, 30.4, 30.2, 29.7, 28.8, 26.3, 26.2, 26.0, 25.9, 20.4, 19.8.
HR-MS, *m/z* (ESI-) calcd for $C_{21}H_{32}N_2O_6$: 407.2182 $[M-H]^-$, found 407.2104.

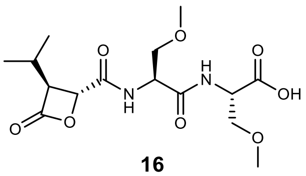

16

16 was prepared as a white solid, 30 mg, 53% yield.
^1H NMR (400 MHz, MeOD) δ 4.80 (d, *J* = 4.4 Hz, 1H), 4.69 (t, *J* = 5.6 Hz, 1H), 4.60 (t, *J* = 4.0 Hz, 1H), 3.82 (dd, *J* = 9.8, 4.4 Hz, 1H), 3.71 (d, *J* = 5.6 Hz, 2H), 3.68 – 3.60 (m, 2H), 3.37 (s, 3H), 3.36 (s, 3H), 2.24 – 2.11 (m, 1H), 1.11 (d, *J* = 6.7 Hz, 3H), 1.07 (d, *J* = 6.7 Hz, 3H).
^{13}C NMR (101 MHz, MeOD) δ 172.7, 171.4, 170.7, 170.6, 72.9, 72.7, 72.3, 65.3, 59.4, 59.3, 54.3, 54.1, 28.9, 20.4, 19.8.
HR-MS, *m/z* (ESI-) calcd for $C_{15}H_{24}N_2O_8$: 359.1454 $[M-H]^-$, found 359.1436.

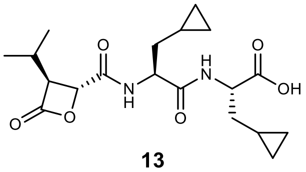

13

13 was prepared as a white solid, 29 mg, 24% yield.
^1H NMR (400 MHz, MeOD) δ 4.77 (d, *J* = 4.4 Hz, 1H), 4.60 – 4.45 (m, 2H), 3.61 (dd, *J* = 8.4, 4.4 Hz, 1H), 2.24 – 2.11 (m, 1H), 1.74 – 1.65 (m, 4H), 1.10 (d, *J* = 6.6 Hz, 3H), 1.06 (d, *J* = 6.7 Hz, 3H), 0.88 – 0.73 (m, 2H), 0.55 – 0.41 (m, 4H), 0.21 – 0.07 (m, 4H).
^{13}C NMR (101 MHz, MeOD) δ 173.6, 173.5, 170.6, 170.4, 72.3, 65.2, 55.2, 54.2, 37.9, 37.8, 28.9, 20.4, 19.8, 8.5, 8.5, 5.2, 5.1, 4.7, 4.6.
HR-MS, *m/z* (ESI-) calcd for $C_{19}H_{28}N_2O_6$: 379.1875 $[M-H]^-$, found 379.1912.

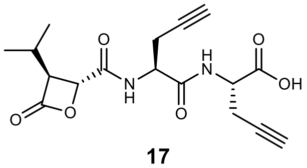

17

17 was prepared as a white solid, 20 mg, 30% yield.
^1H NMR (400 MHz, MeOD) δ 8.59 (d, *J* = 8.0 Hz, 1H), 8.37 (d, *J* = 7.8 Hz, 1H), 4.81 (d, *J* = 4.4 Hz, 1H), 4.74 – 4.62 (m, 1H), 4.60 – 4.49 (m, 1H), 3.63 (dd, *J* = 8.4, 4.4 Hz, 1H), 2.85 – 2.54 (m, 4H), 2.39 (t, *J* = 2.7 Hz, 1H), 2.35 (t, *J* = 2.6 Hz, 1H), 2.27 – 2.06 (m, 1H), 1.11 (d, *J* = 6.7 Hz, 3H), 1.08 (d, *J* = 6.7 Hz, 3H).
^{13}C NMR (126 MHz, MeOD) δ 172.9, 171.7, 170.7, 170.6, 80.0, 79.9, 72.5, 72.4, 72.2, 65.5, 53.2, 52.6, 28.9, 22.5, 22.4, 20.5, 19.8.
HR-MS, *m/z* (ESI-) calcd for $C_{17}H_{20}N_2O_6$: 347.1243 $[M-H]^-$, found 347.1263.

NMR spectra of compounds synthesised enzymatically

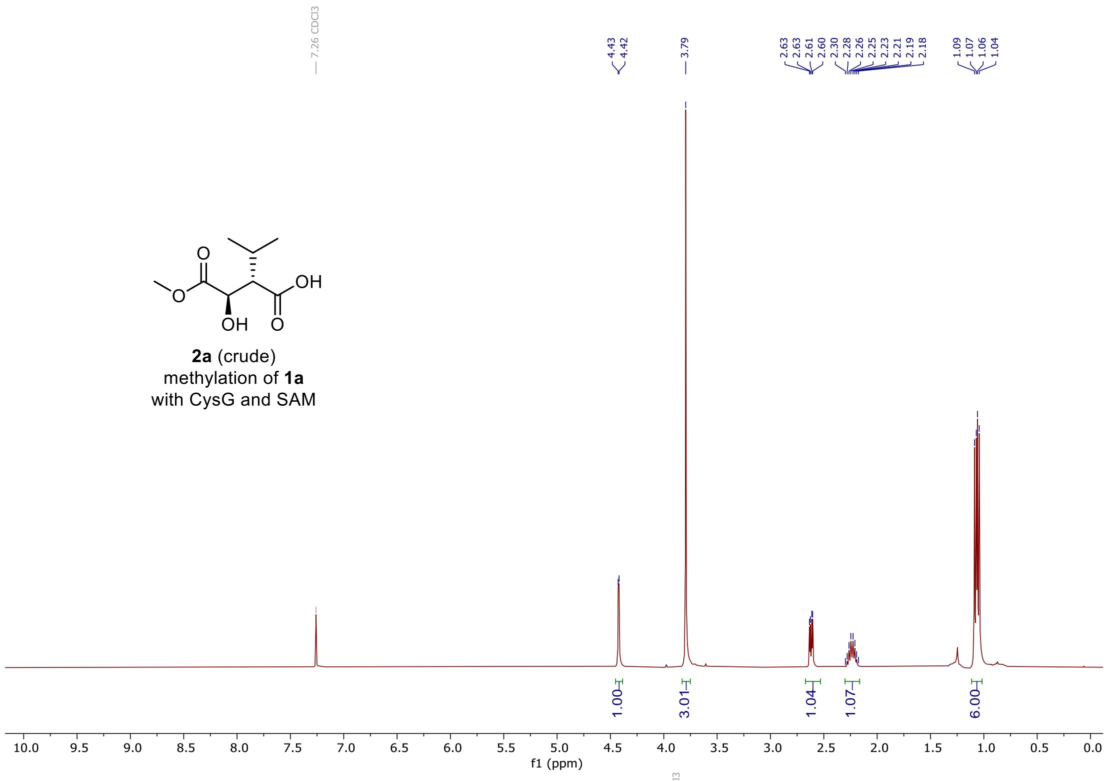

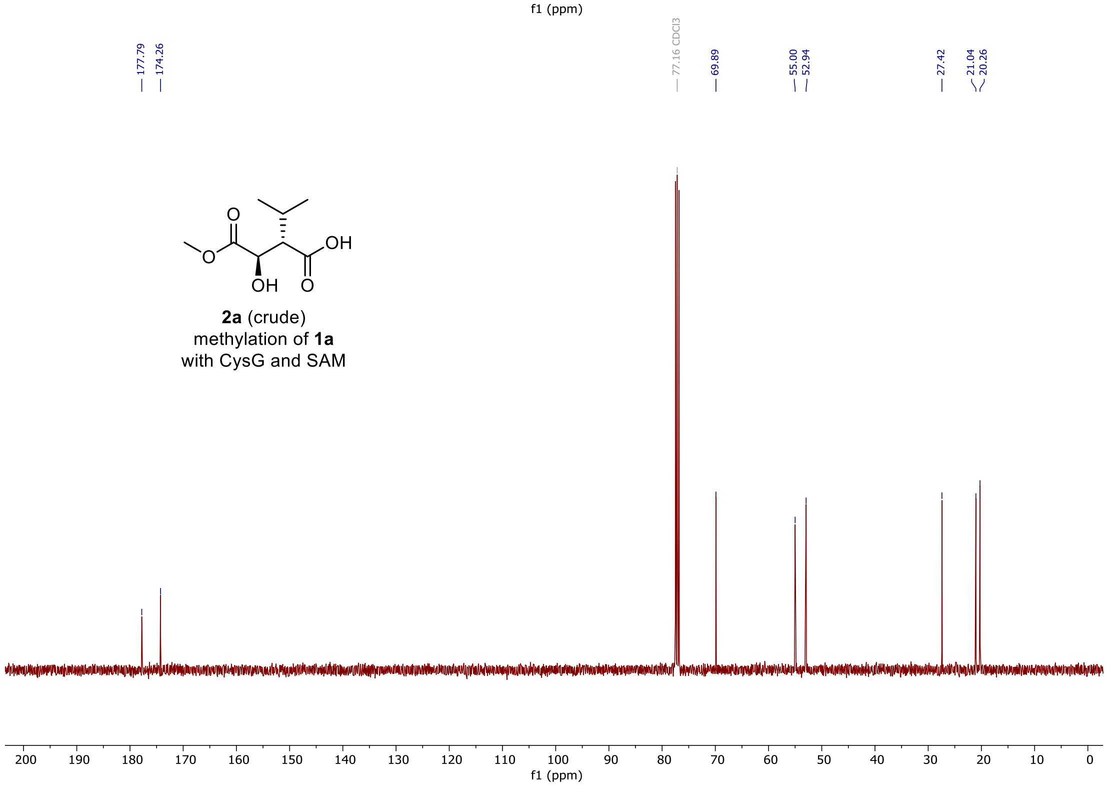

Supplementary Fig. 9. NMR of compound 2a prepared enzymatically.

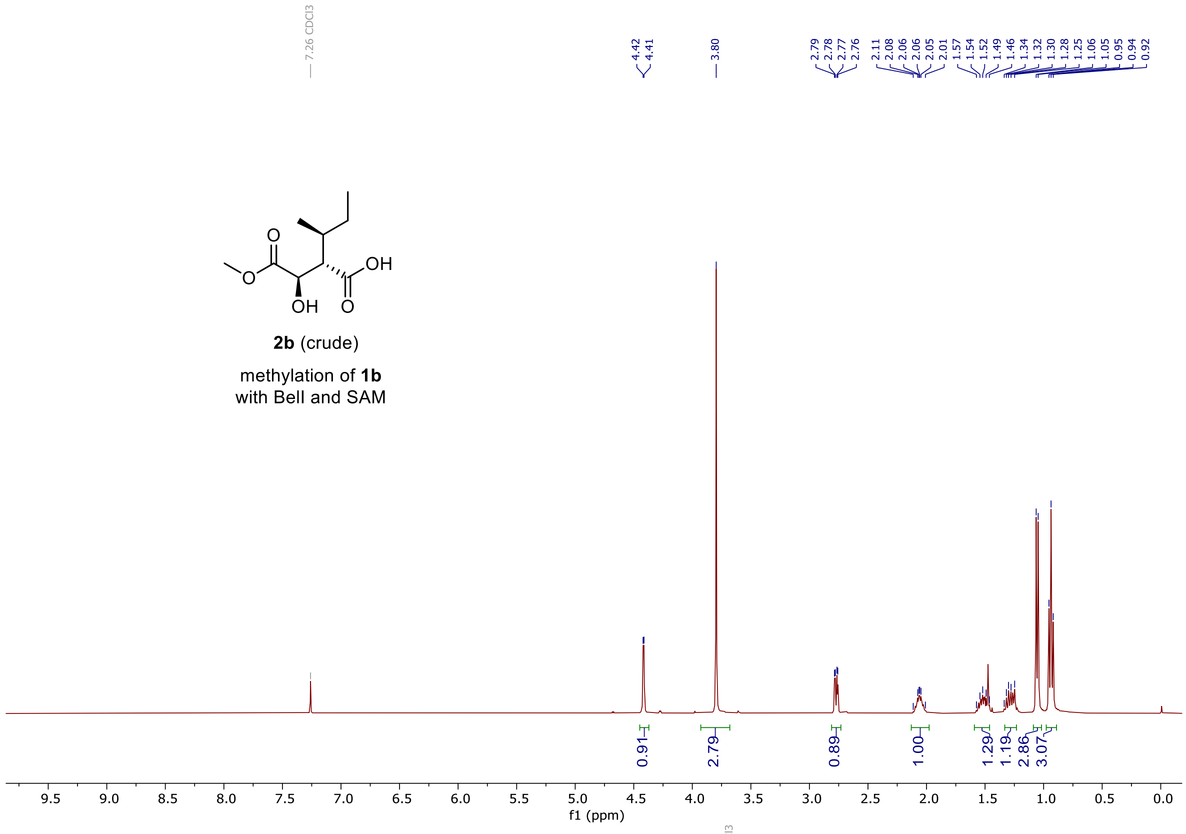

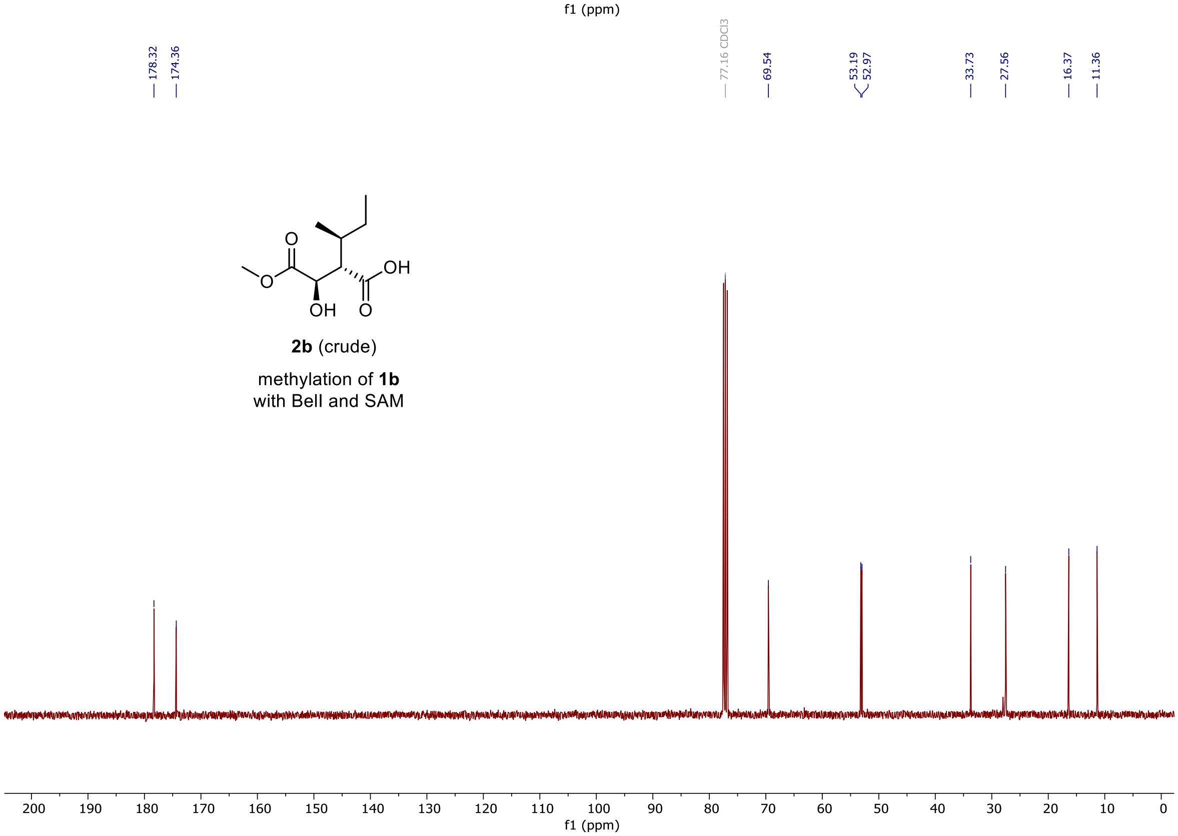

Supplementary Fig. 10. NMR of compound 2b prepared enzymatically.

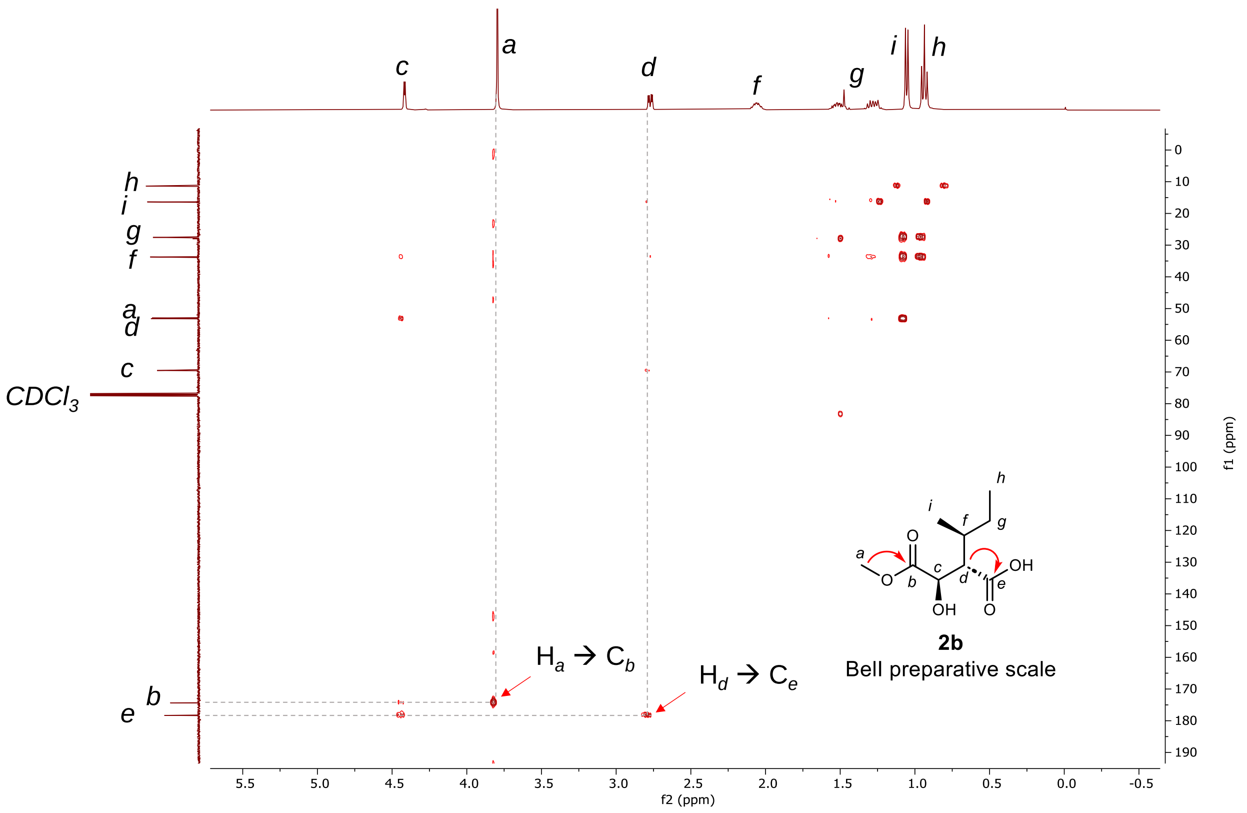

Supplementary Fig. 11. HMBC spectra of compound 2b prepared enzymatically.

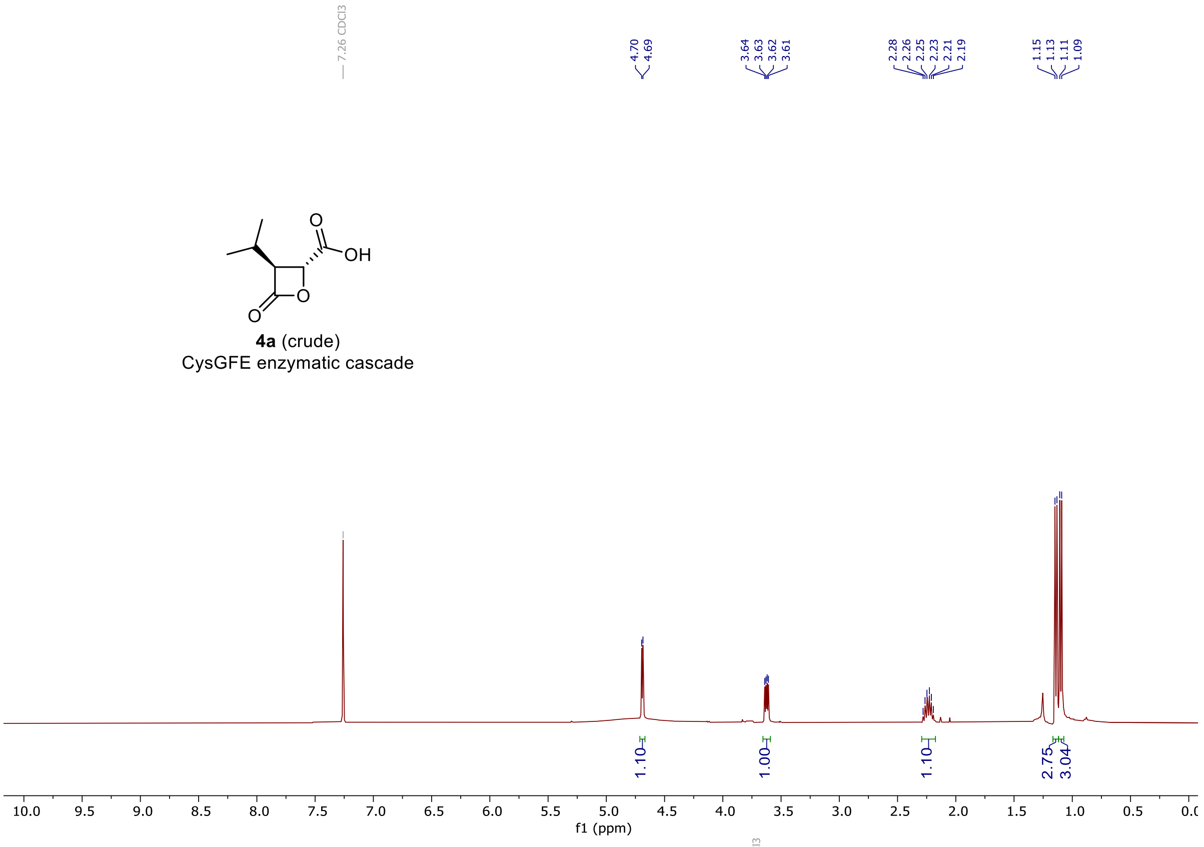

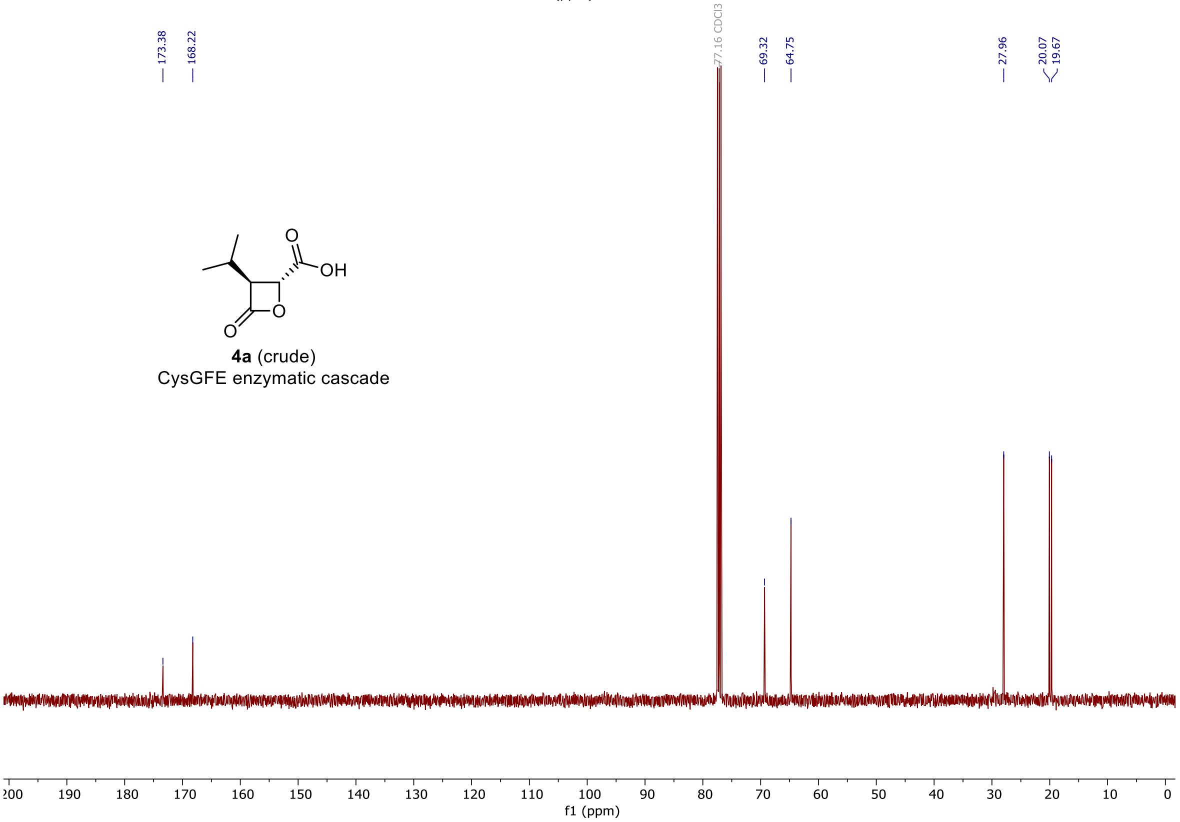

Supplementary Fig. 12. NMR of compound 4a prepared enzymatically.

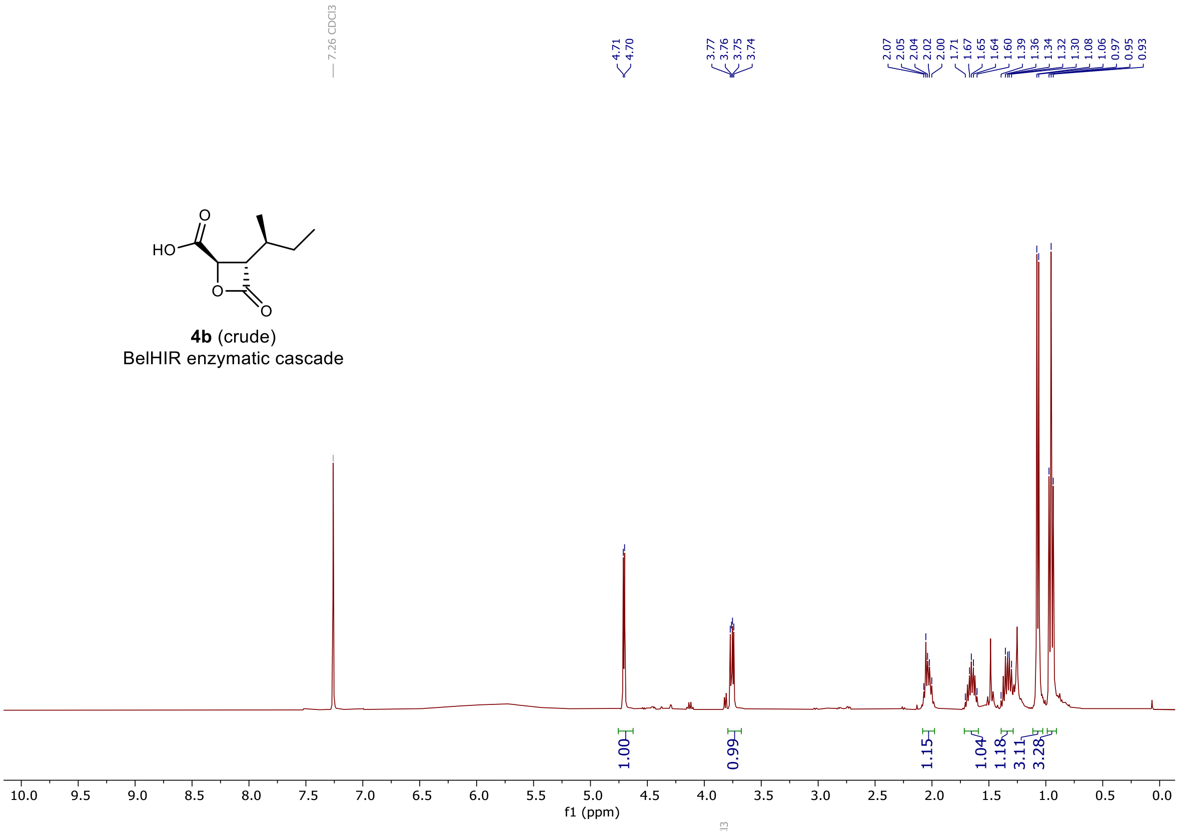

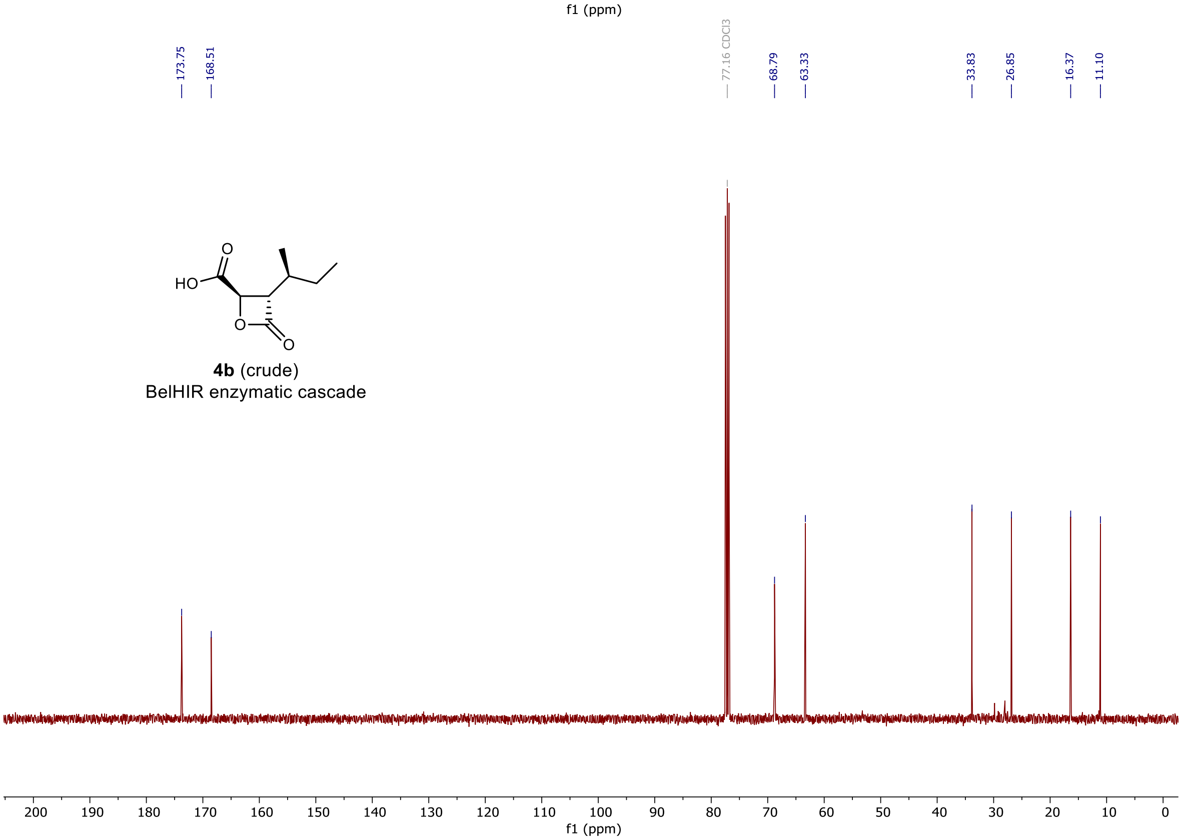

Supplementary Fig. 13. NMR of compound 4b prepared enzymatically.

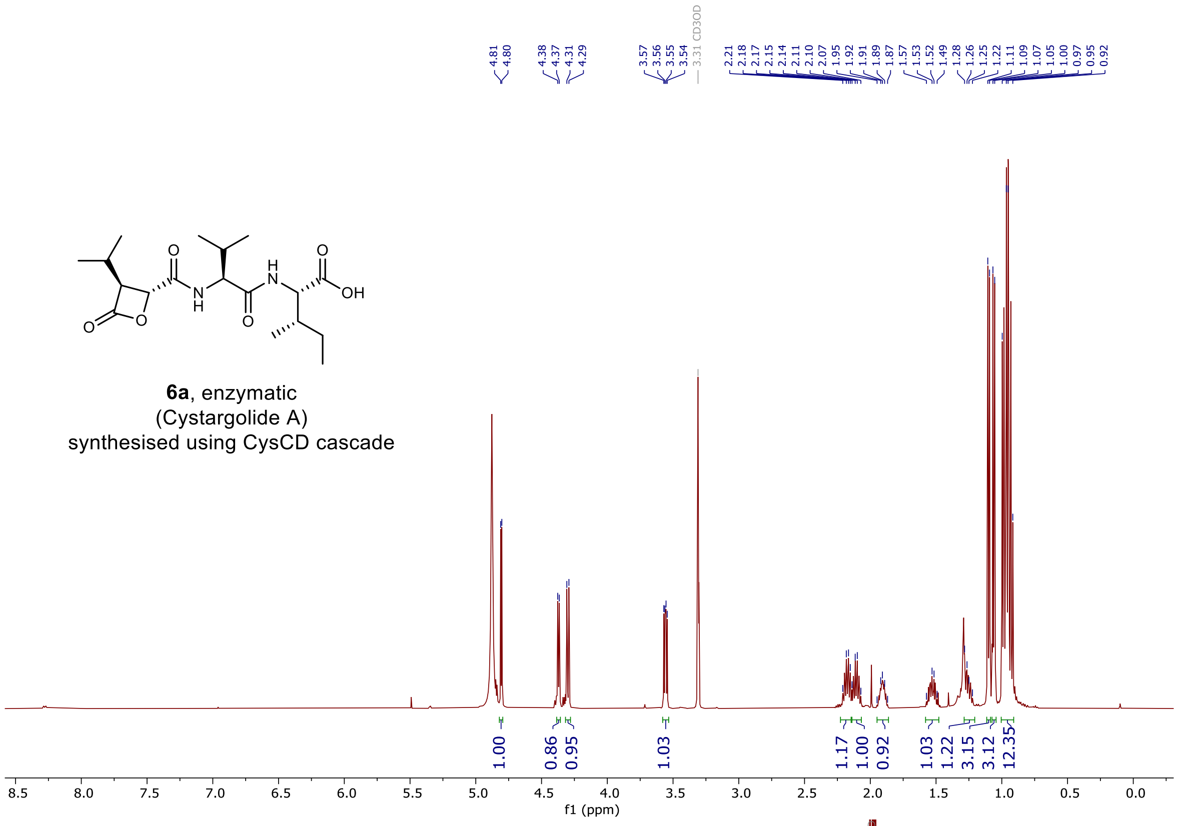

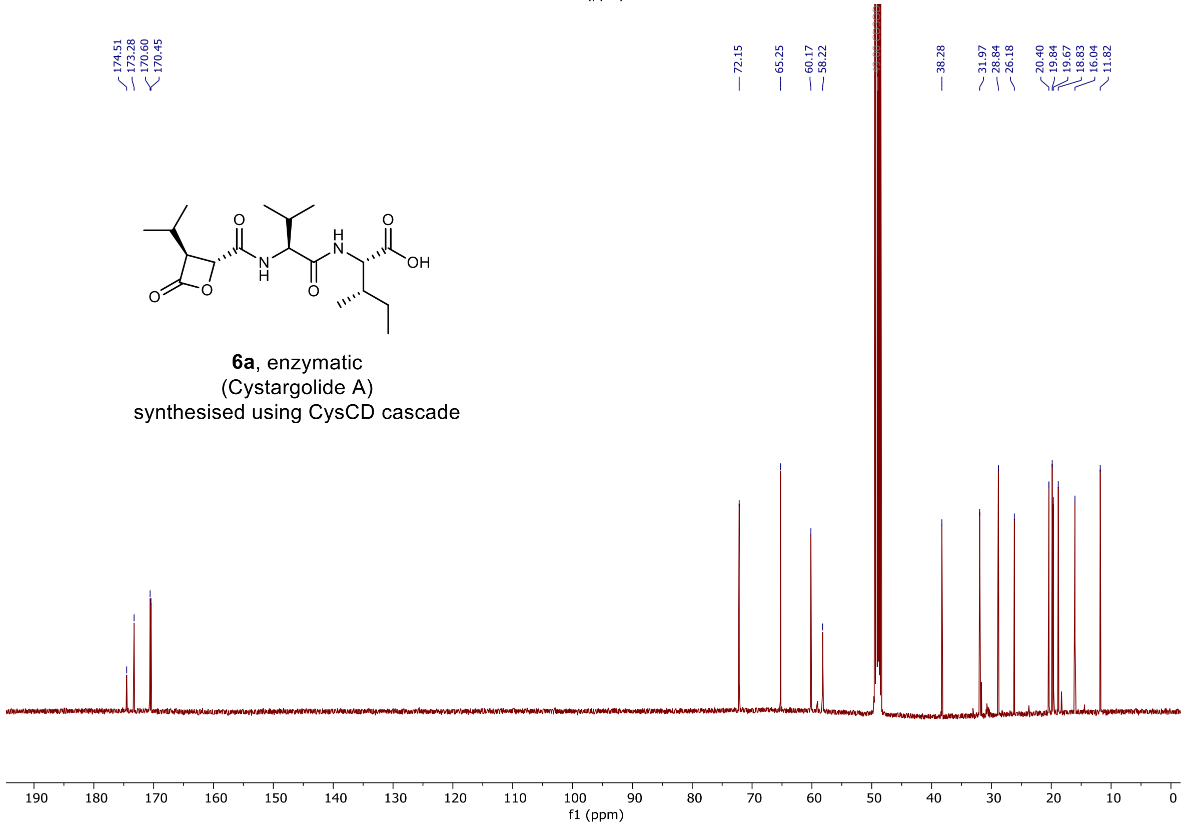

Supplementary Fig. 14. NMR of compound 6a prepared enzymatically.

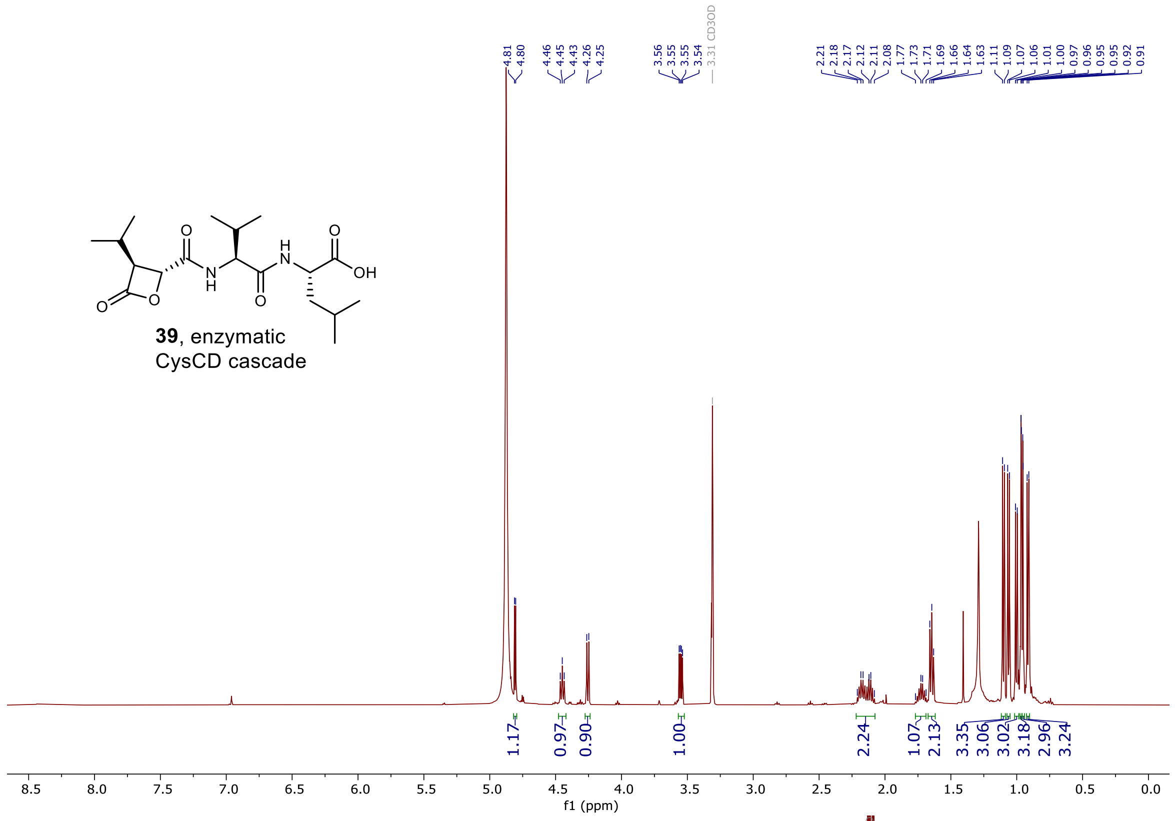

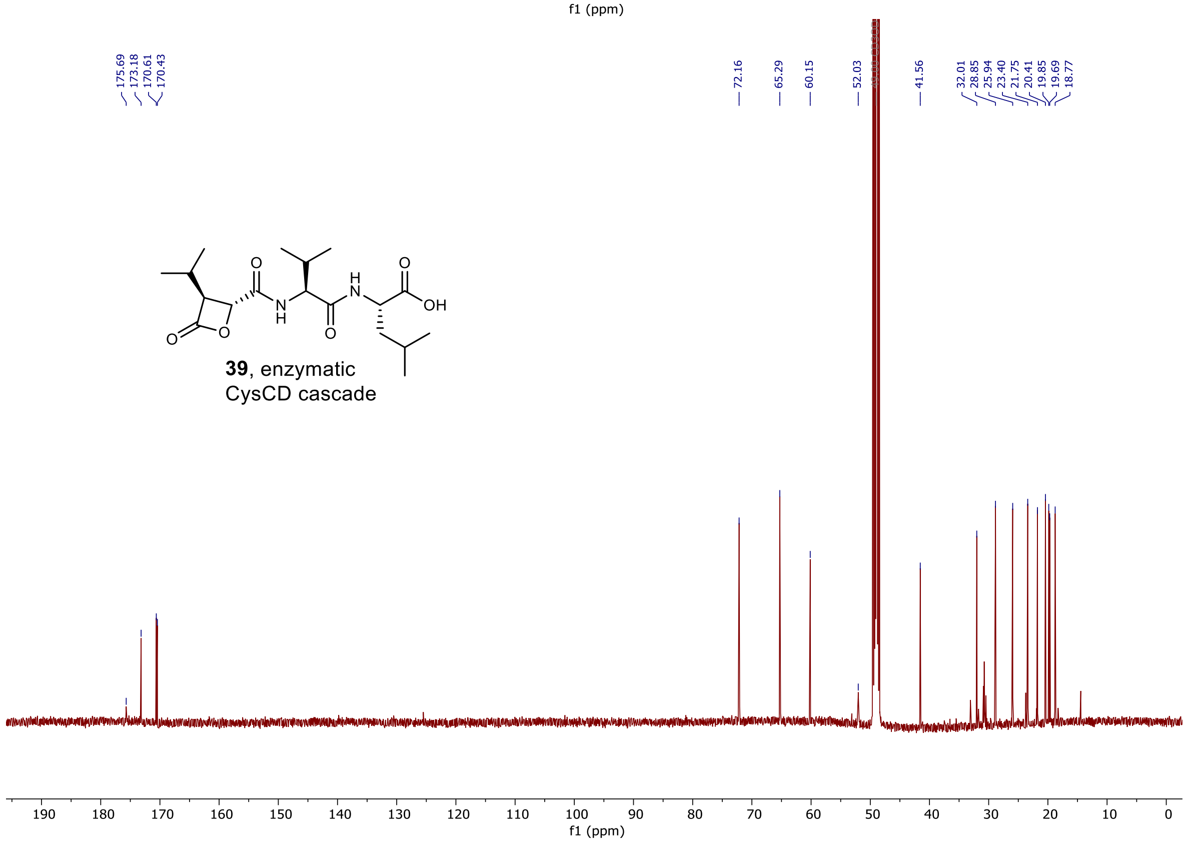

Supplementary Fig. 15. NMR of compound 39 prepared enzymatically.

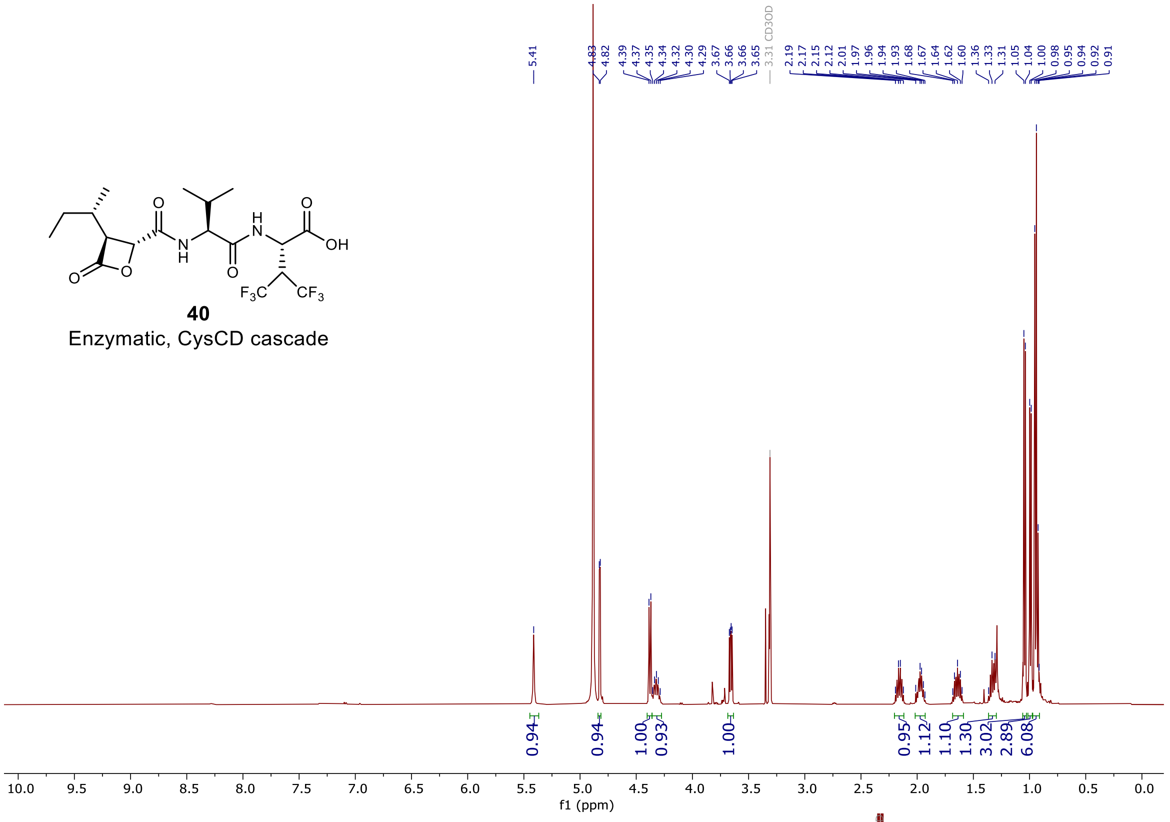

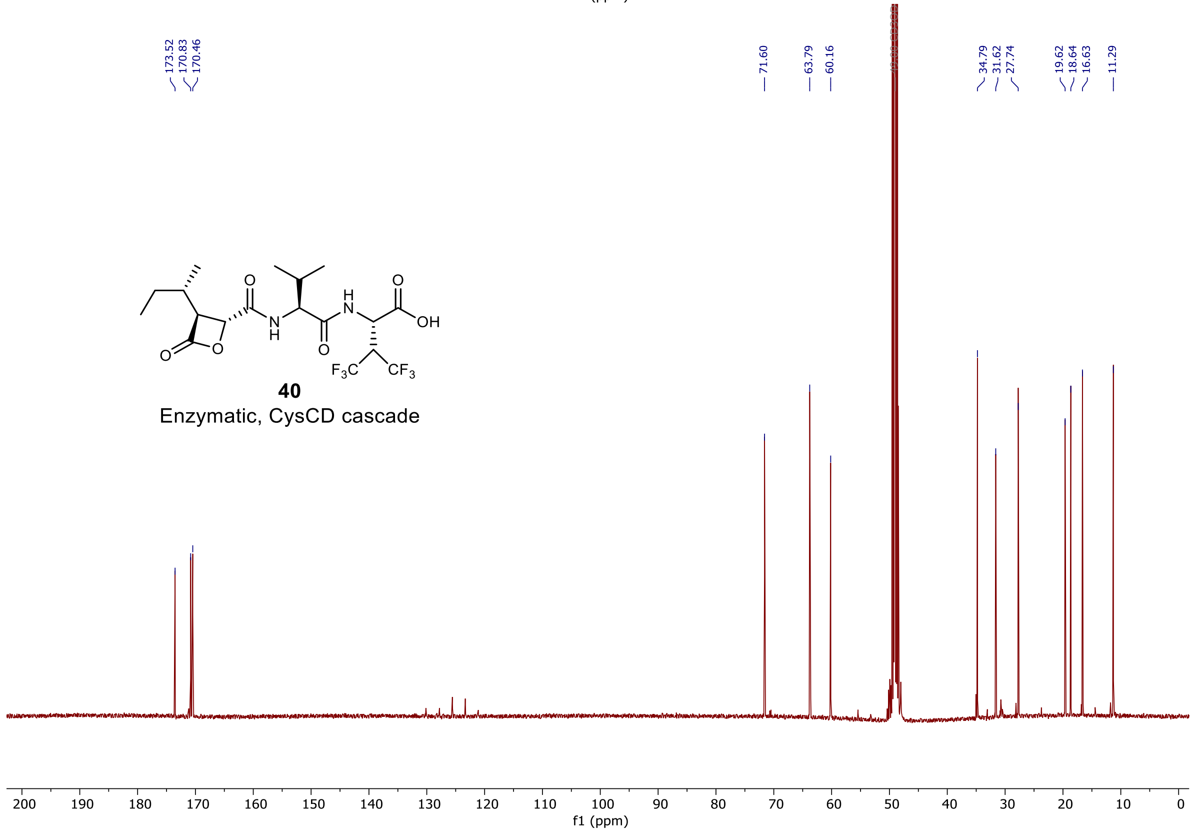

Supplementary Fig. 16. NMR of compound 40 prepared enzymatically.

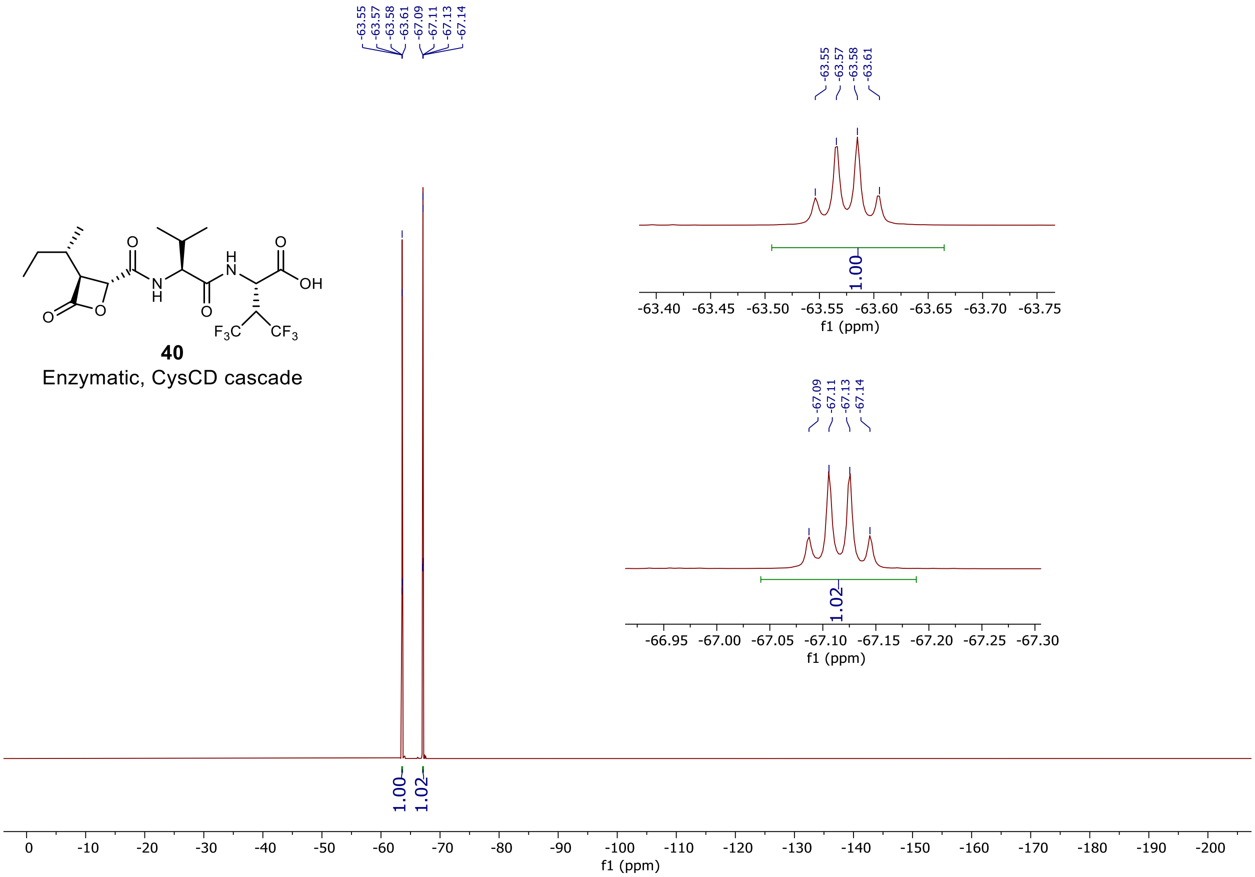

Supplementary Fig. 17. ^{19}F-NMR of compound 40 prepared enzymatically.

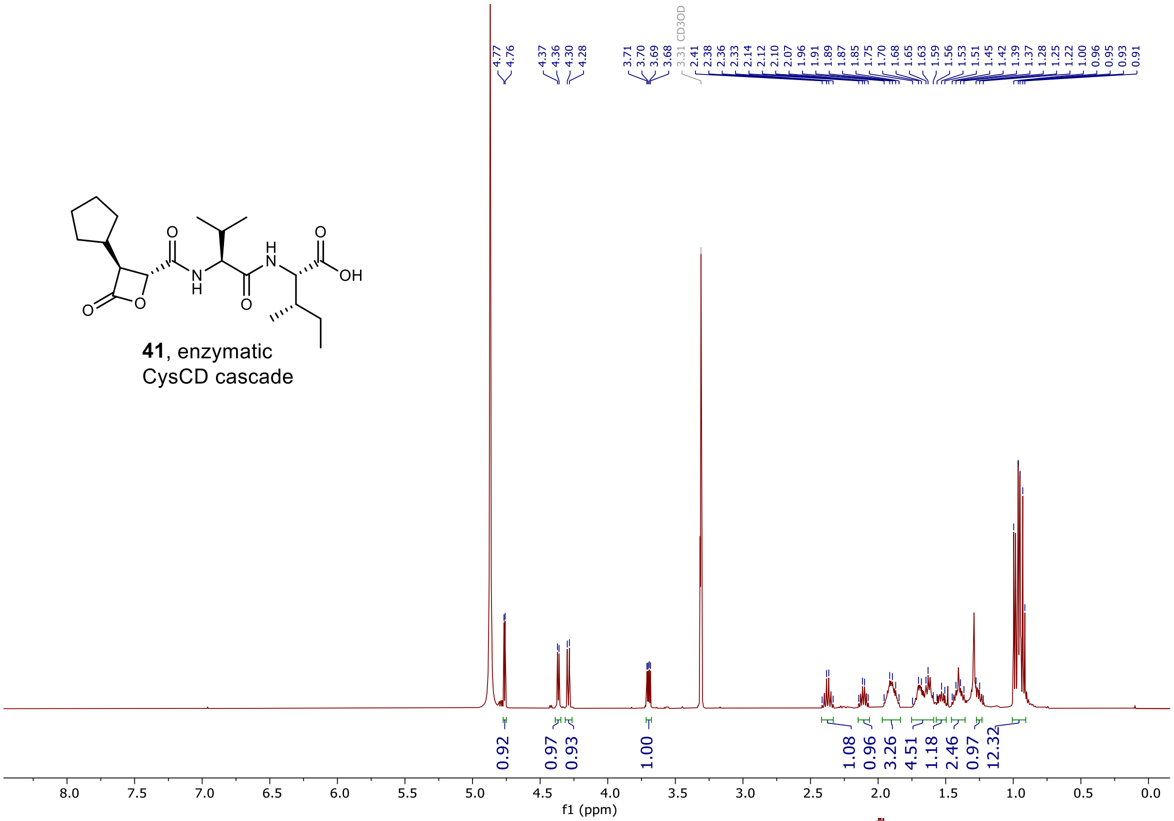

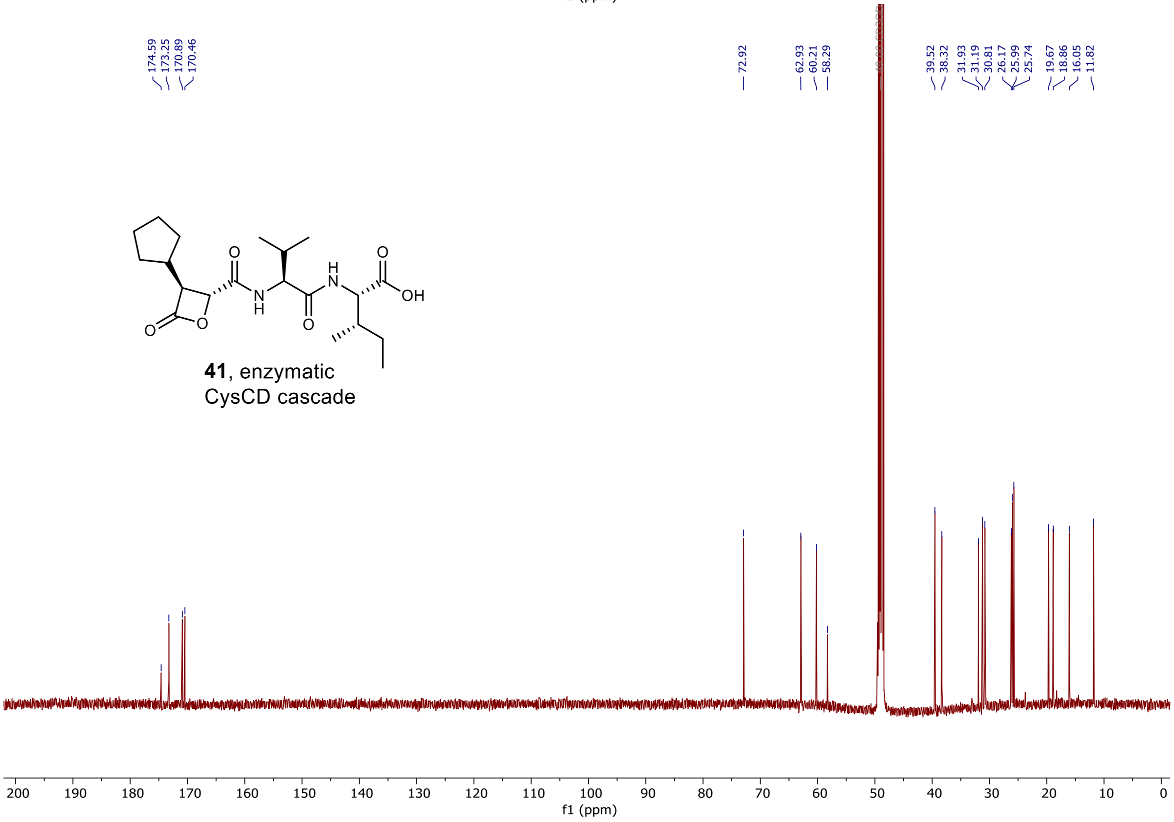

Supplementary Fig. 18. NMR of compound 41 prepared enzymatically.

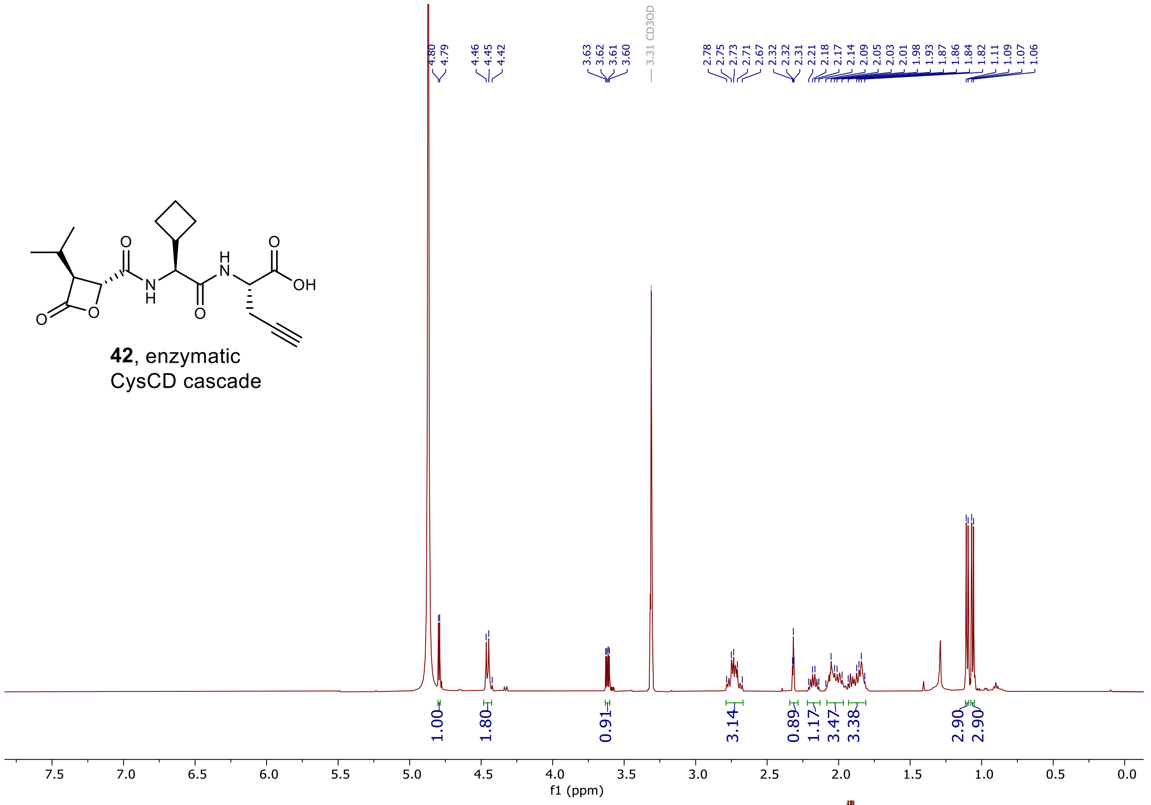

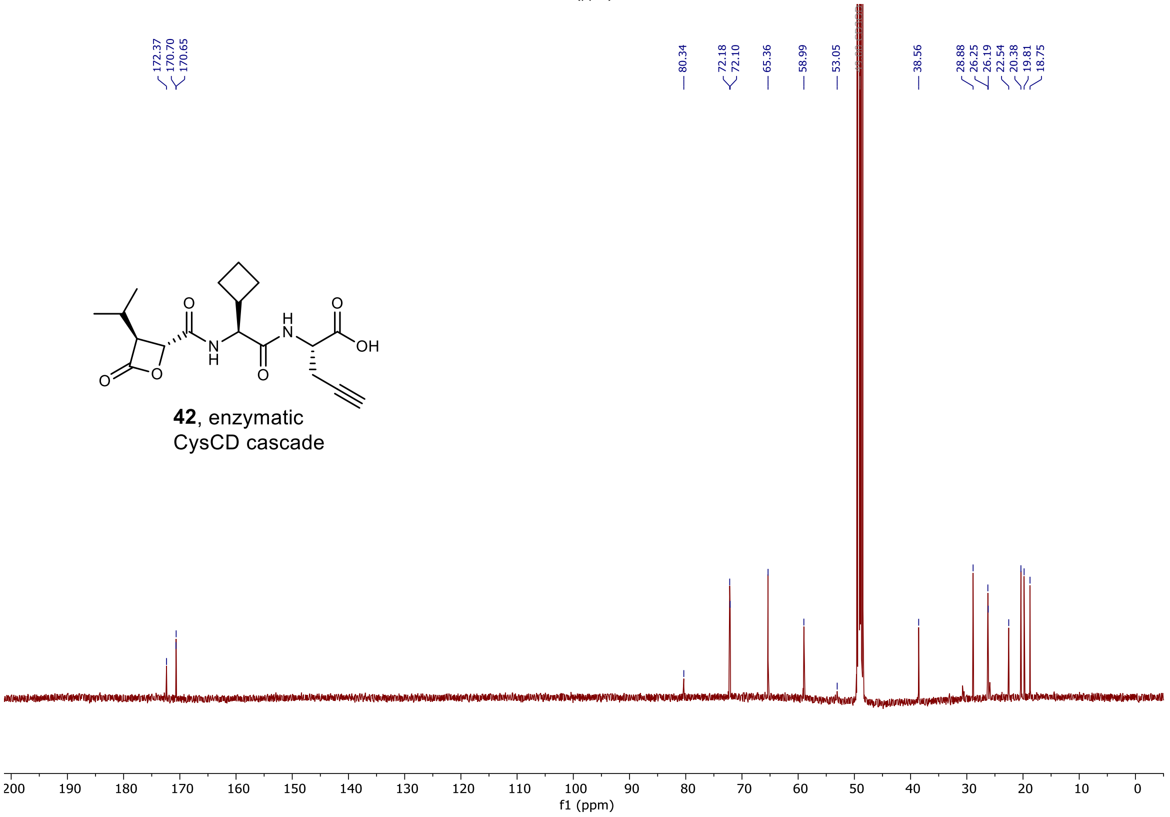

Supplementary Fig. 19. NMR of compound 42 prepared enzymatically.

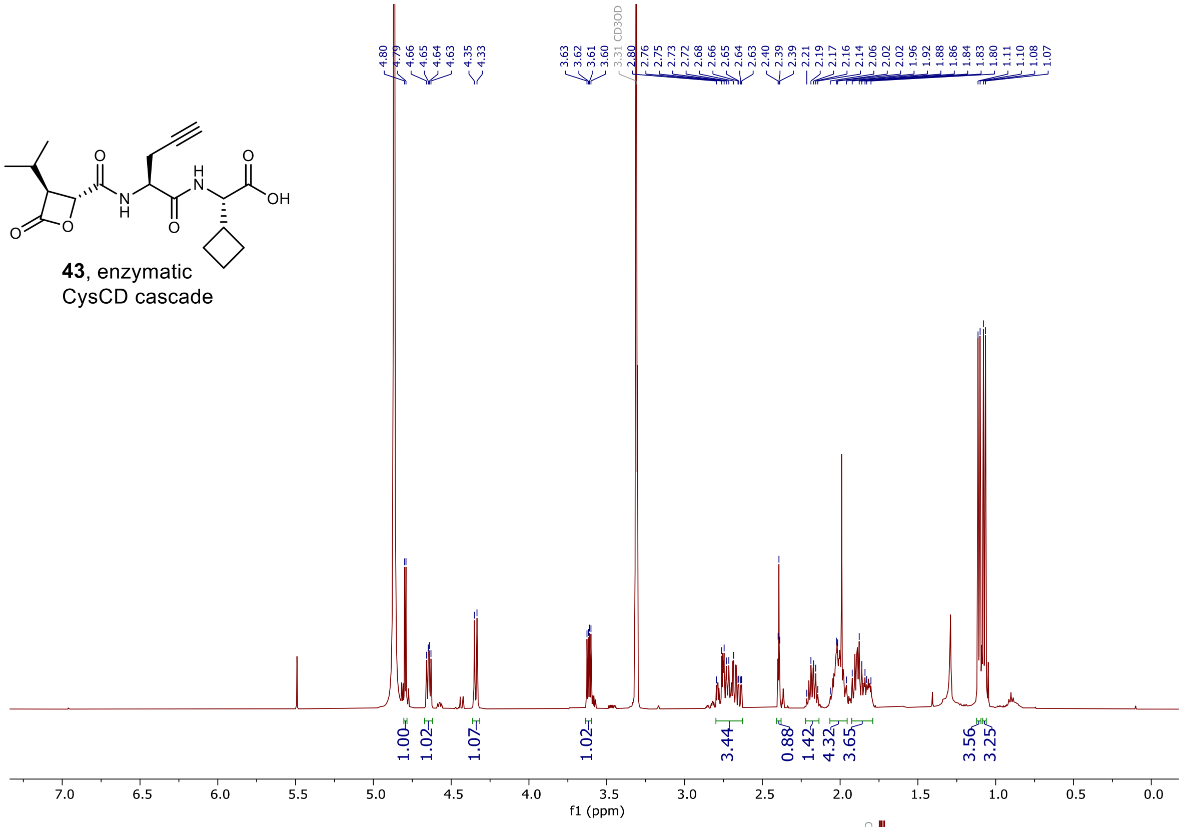

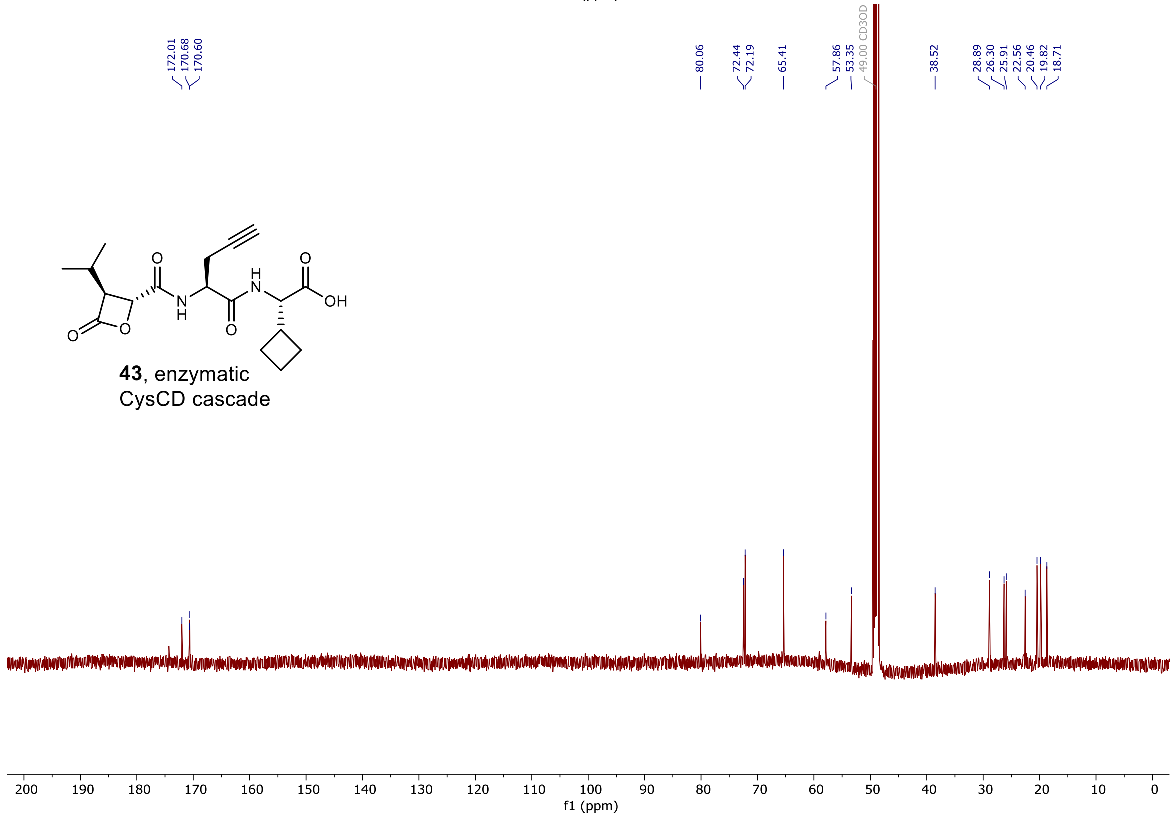

Supplementary Fig. 20. NMR of compound 43 prepared enzymatically.

NMR spectra of chemically synthesised standards

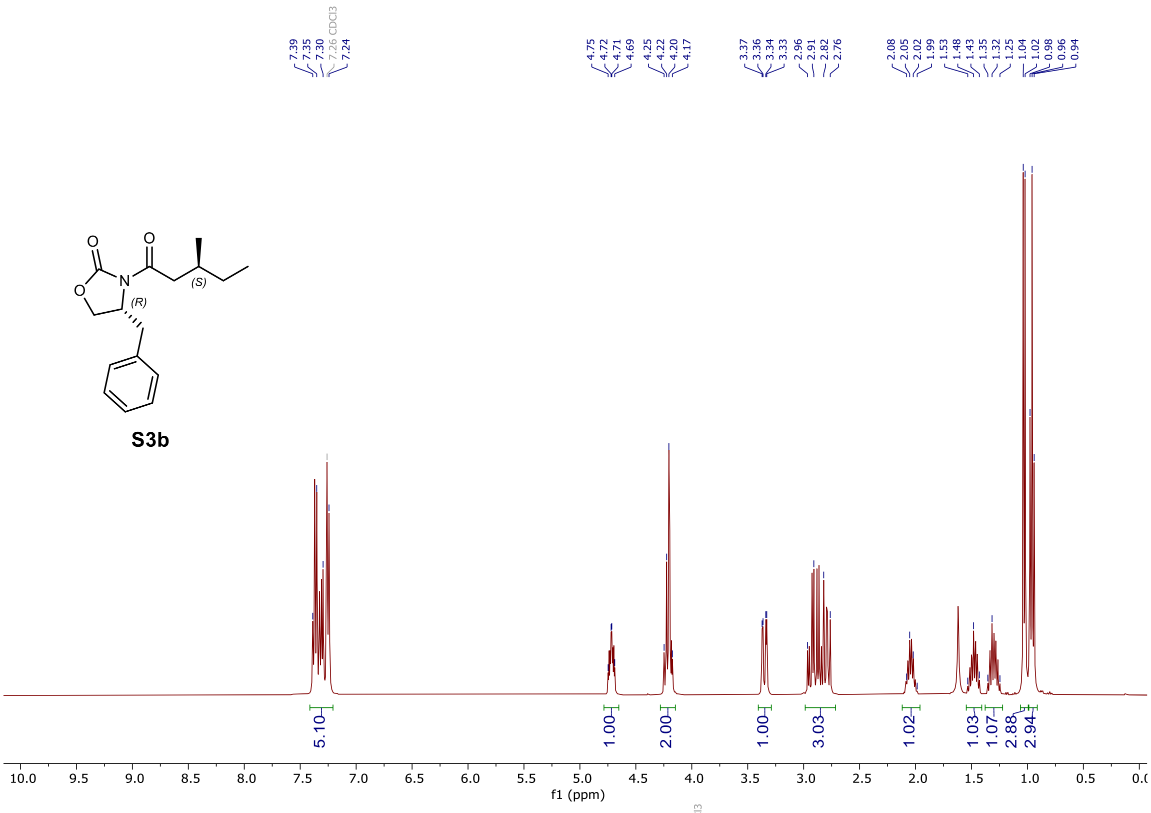

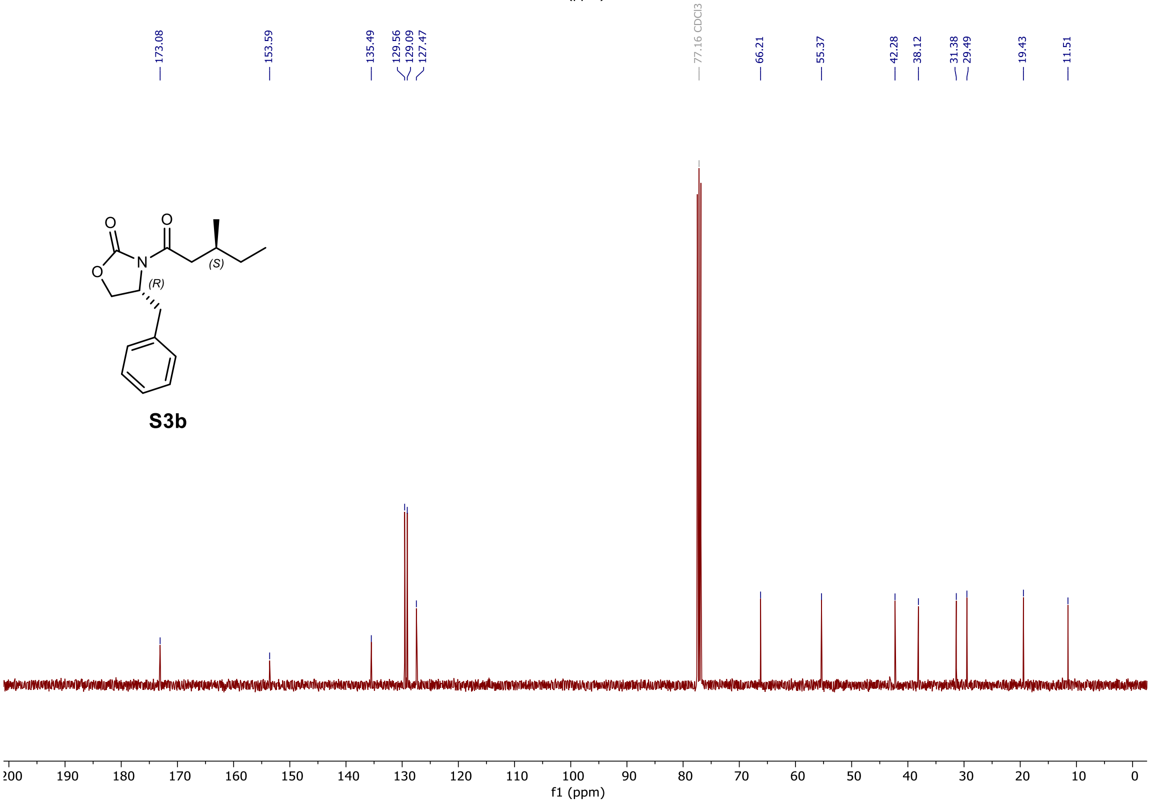

Supplementary Fig. 21. NMR of compound S3b.

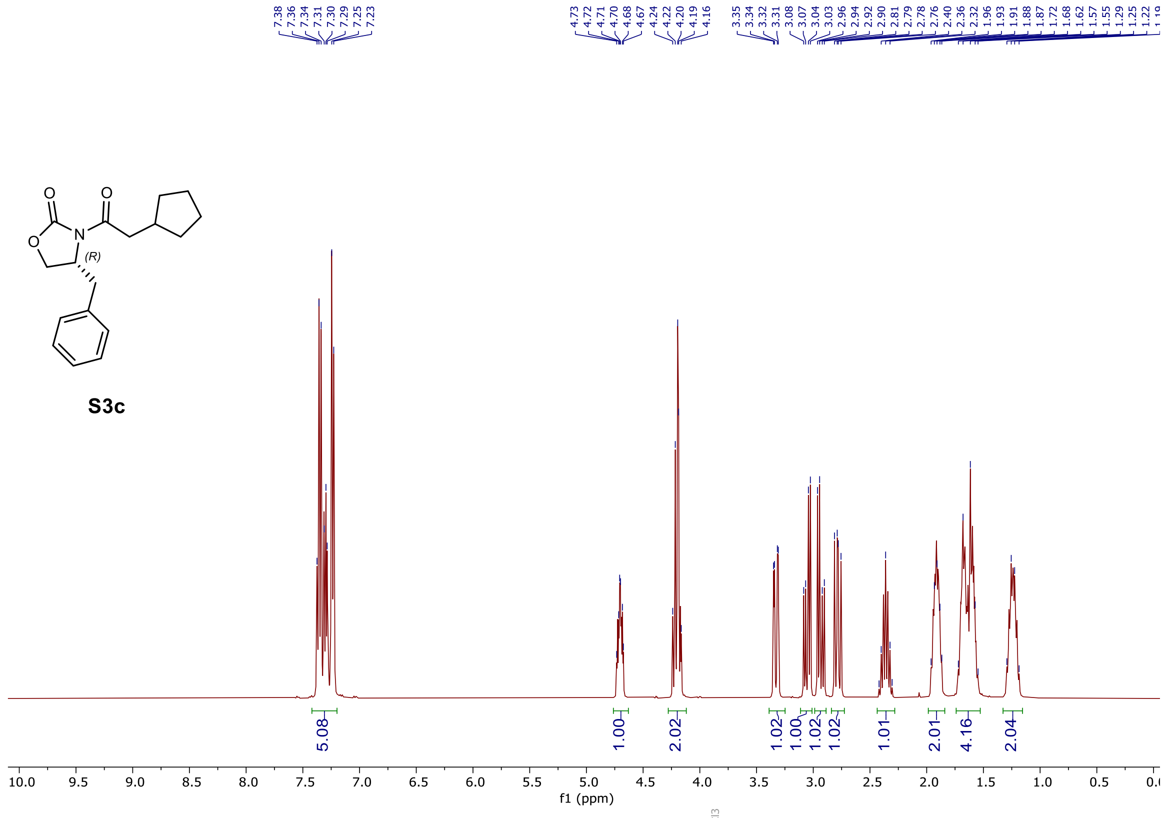

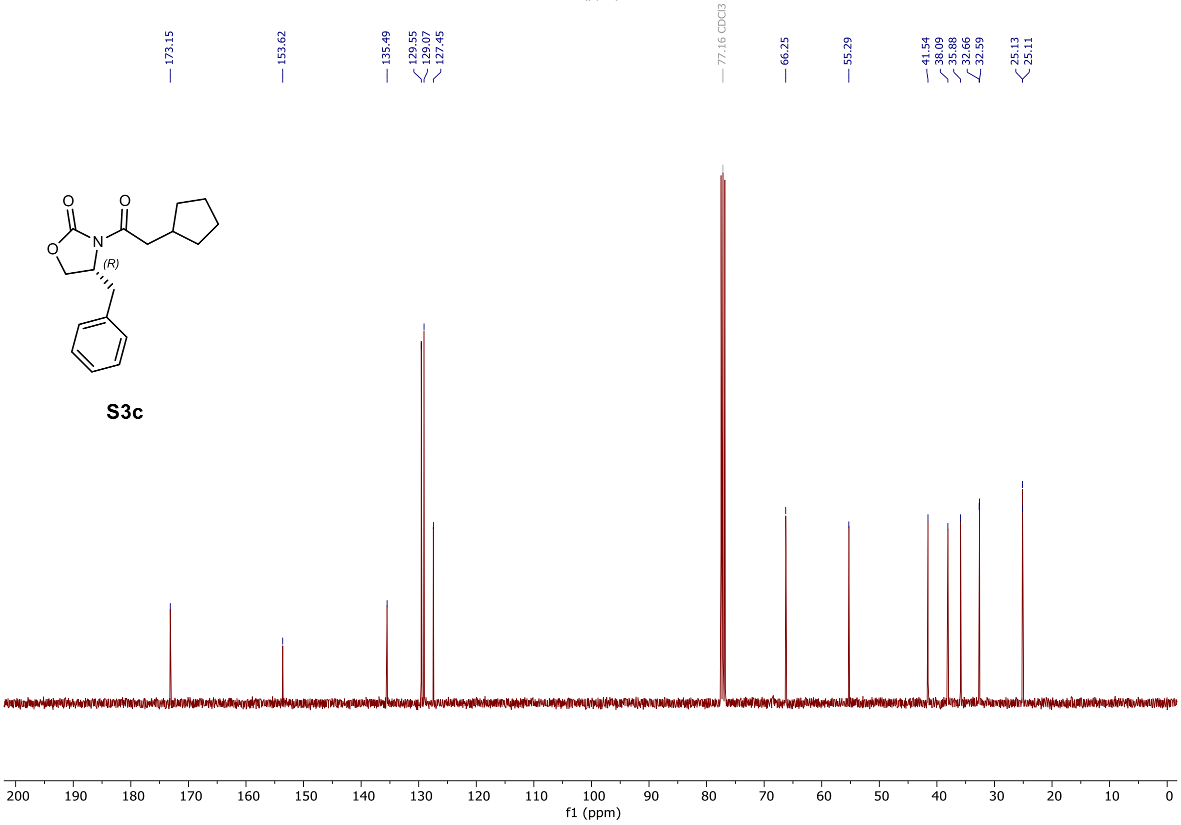

Supplementary Fig. 22. NMR of compound S3c.

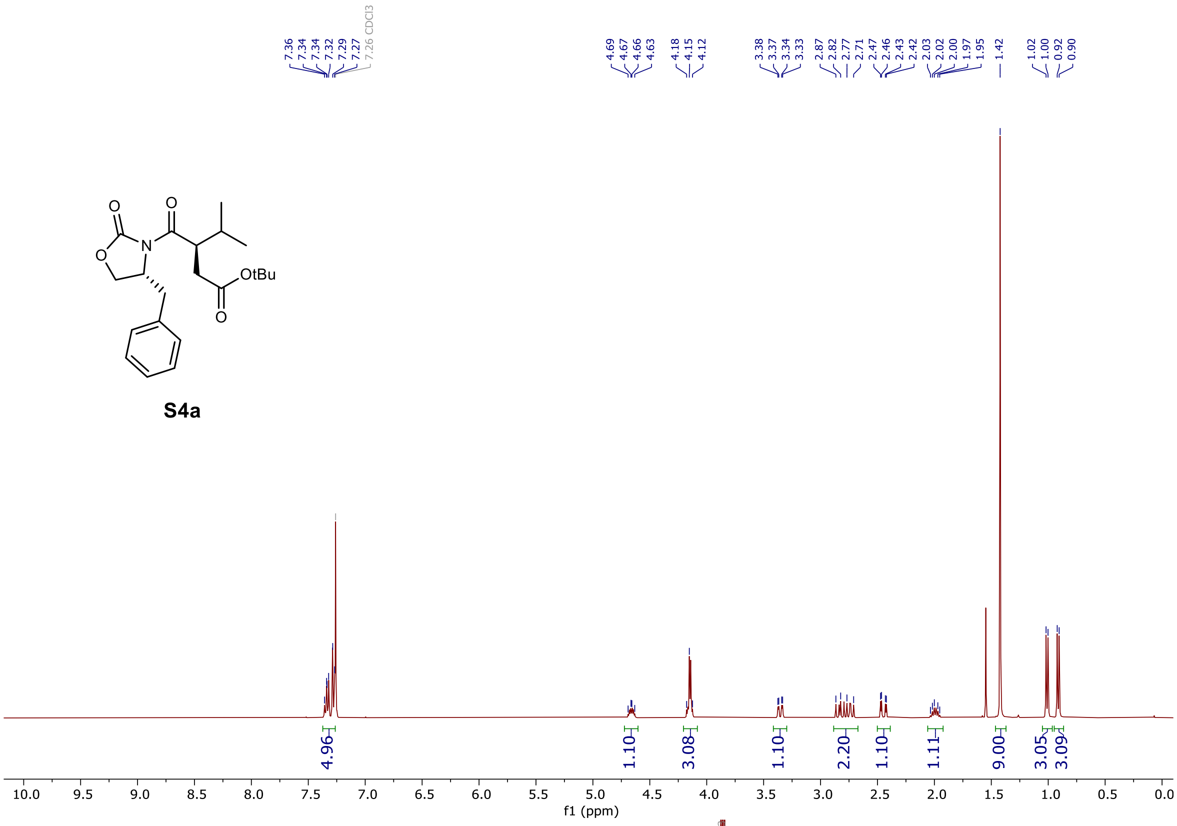

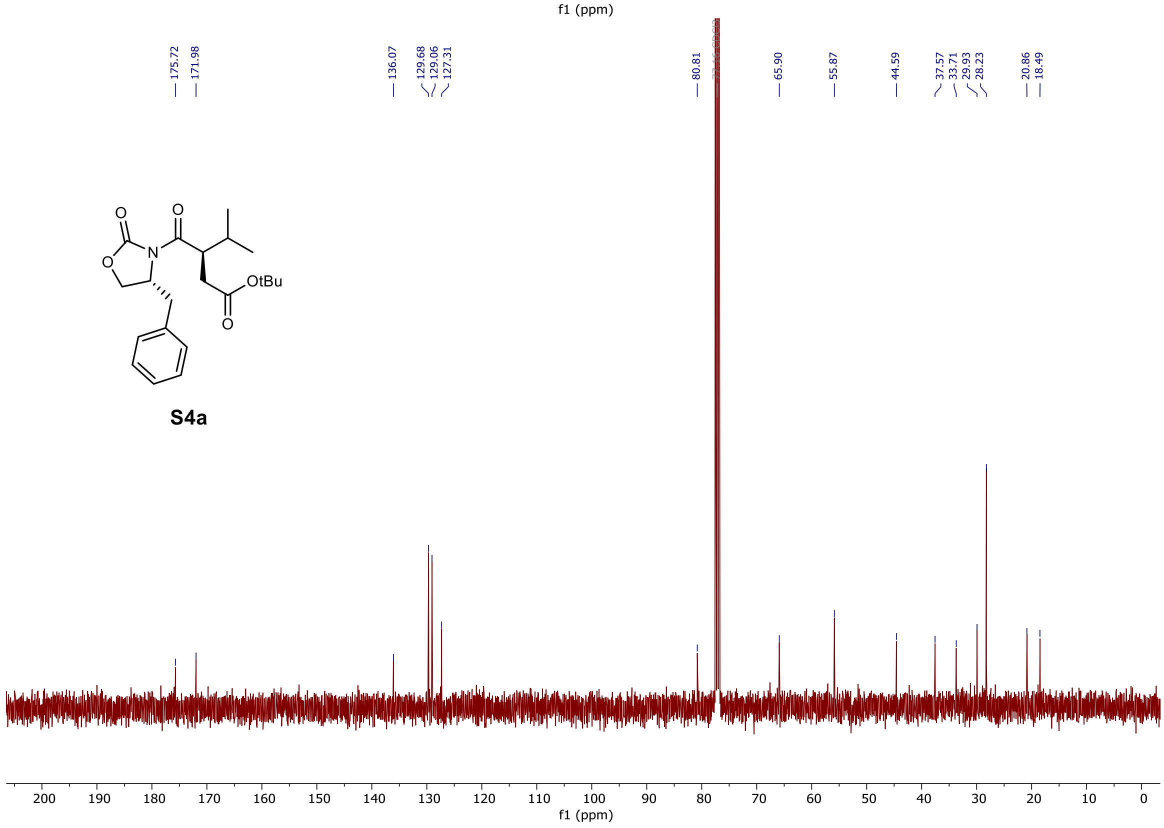

Supplementary Fig. 23. NMR of compound S4a.

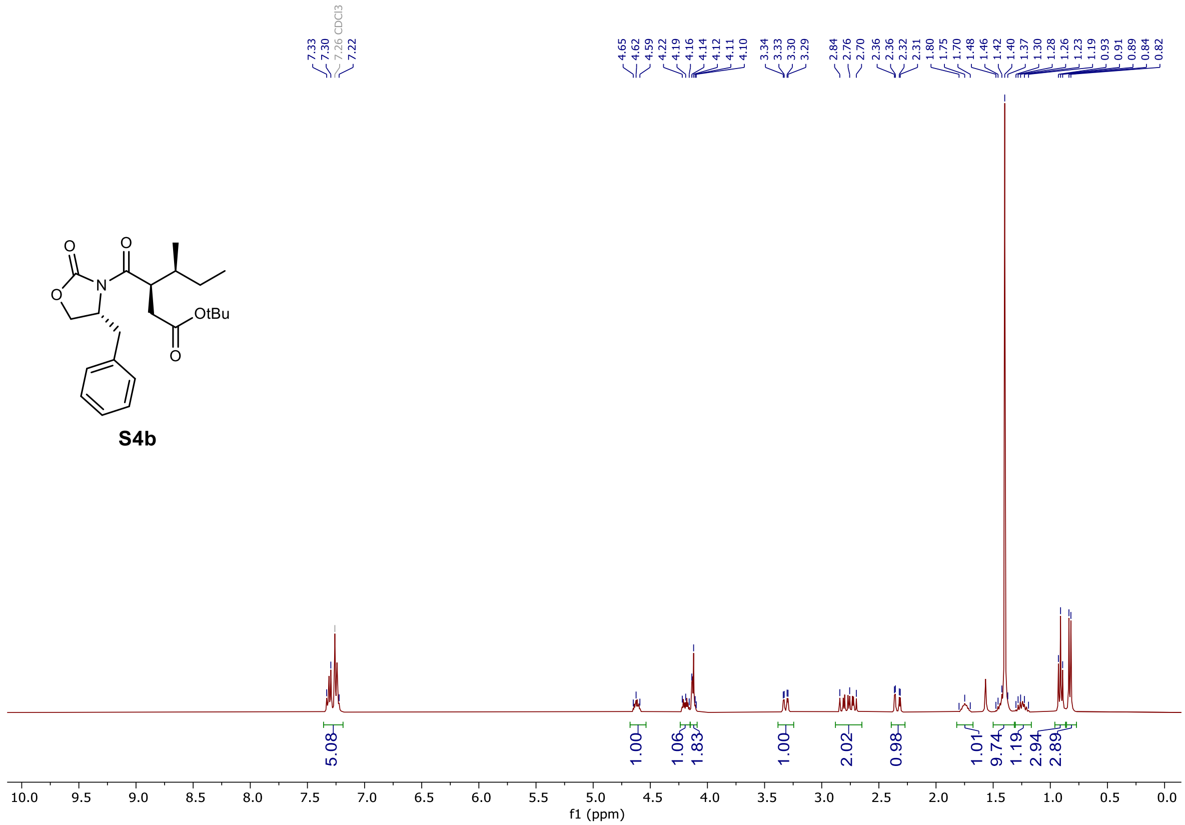

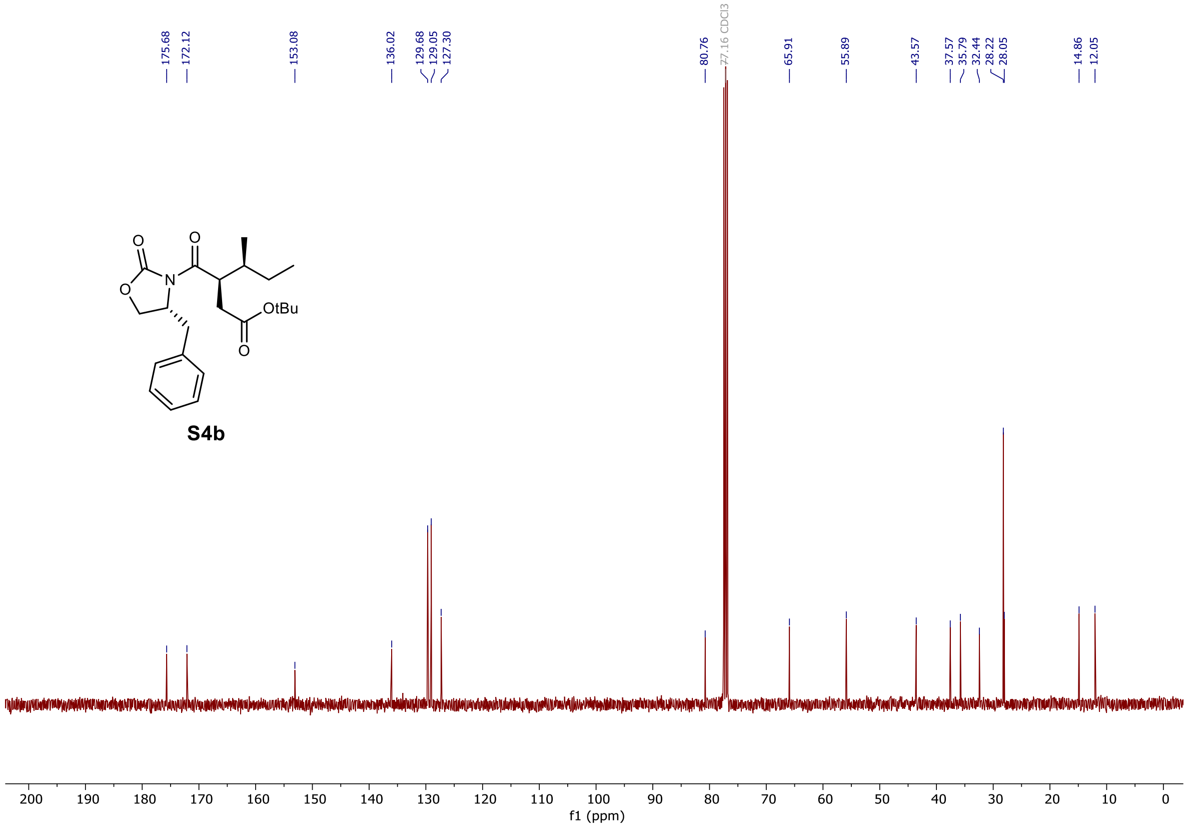

Supplementary Fig. 24. NMR of compound S4b.

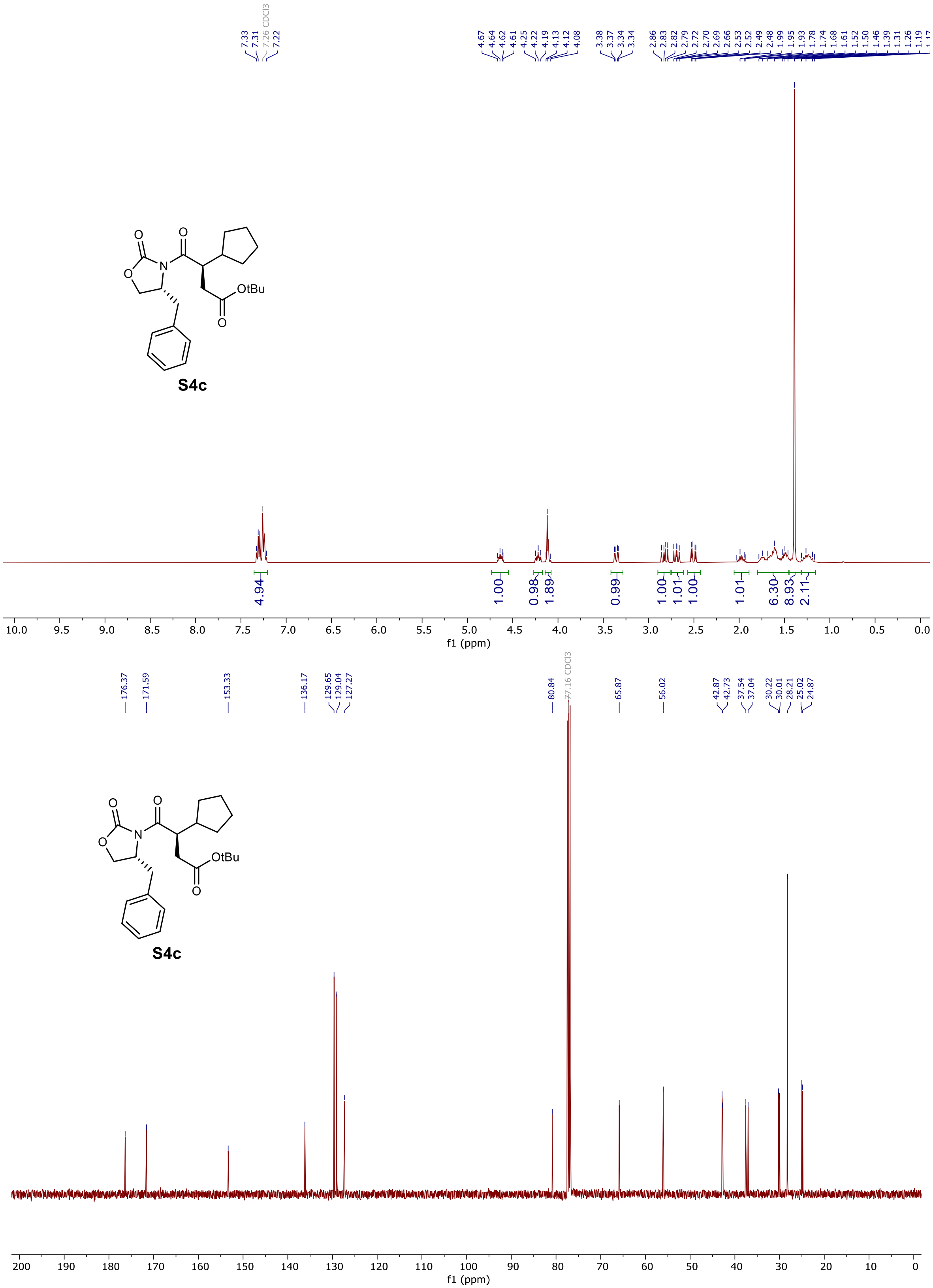

Supplementary Fig. 25. NMR of compound S4c.

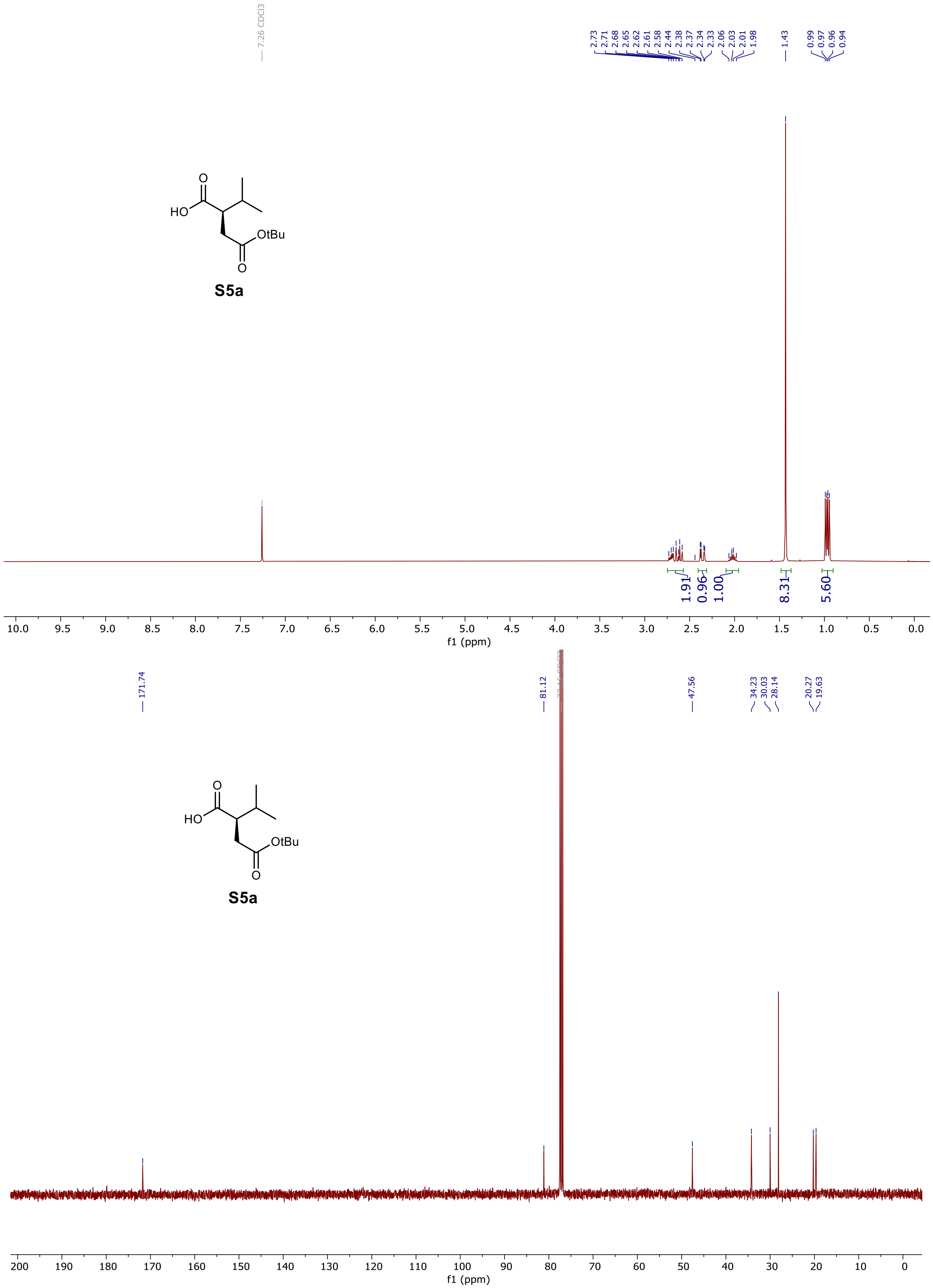

Supplementary Fig. 26. NMR of compound S5a.

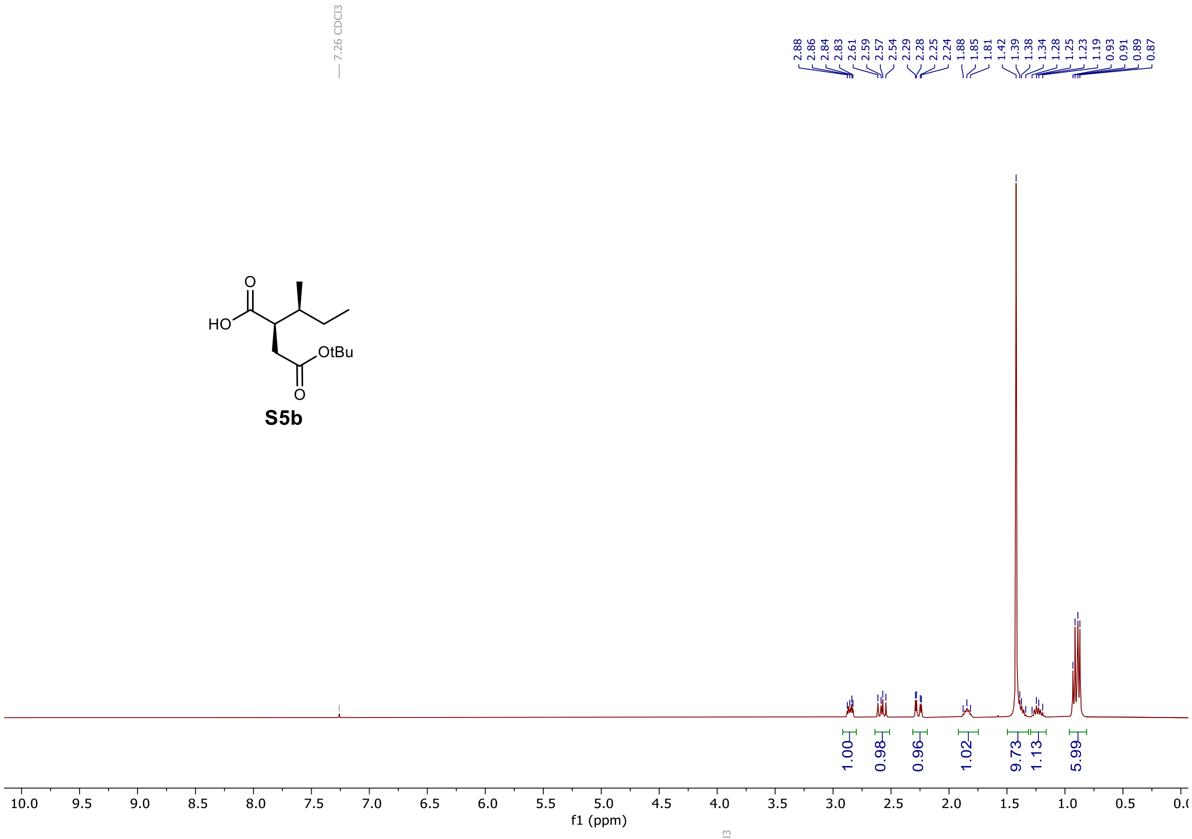

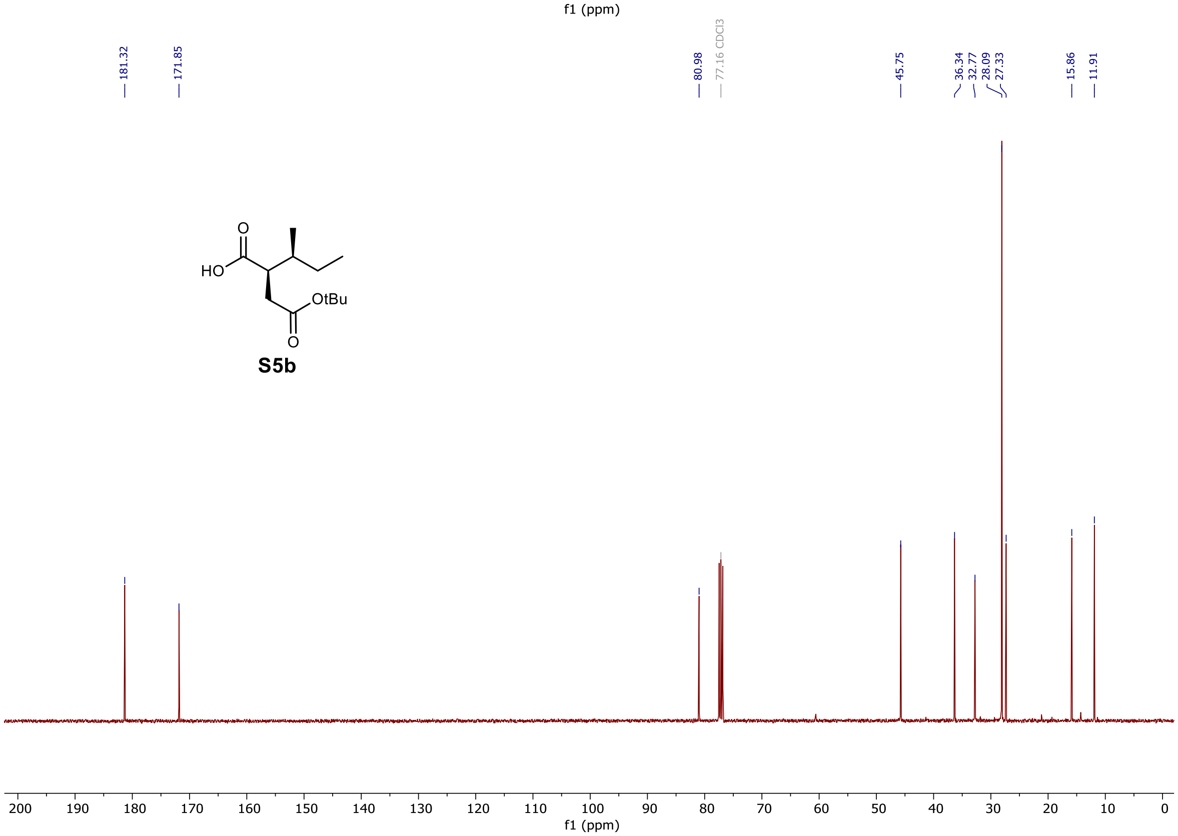

Supplementary Fig. 27. NMR of compound S5b.

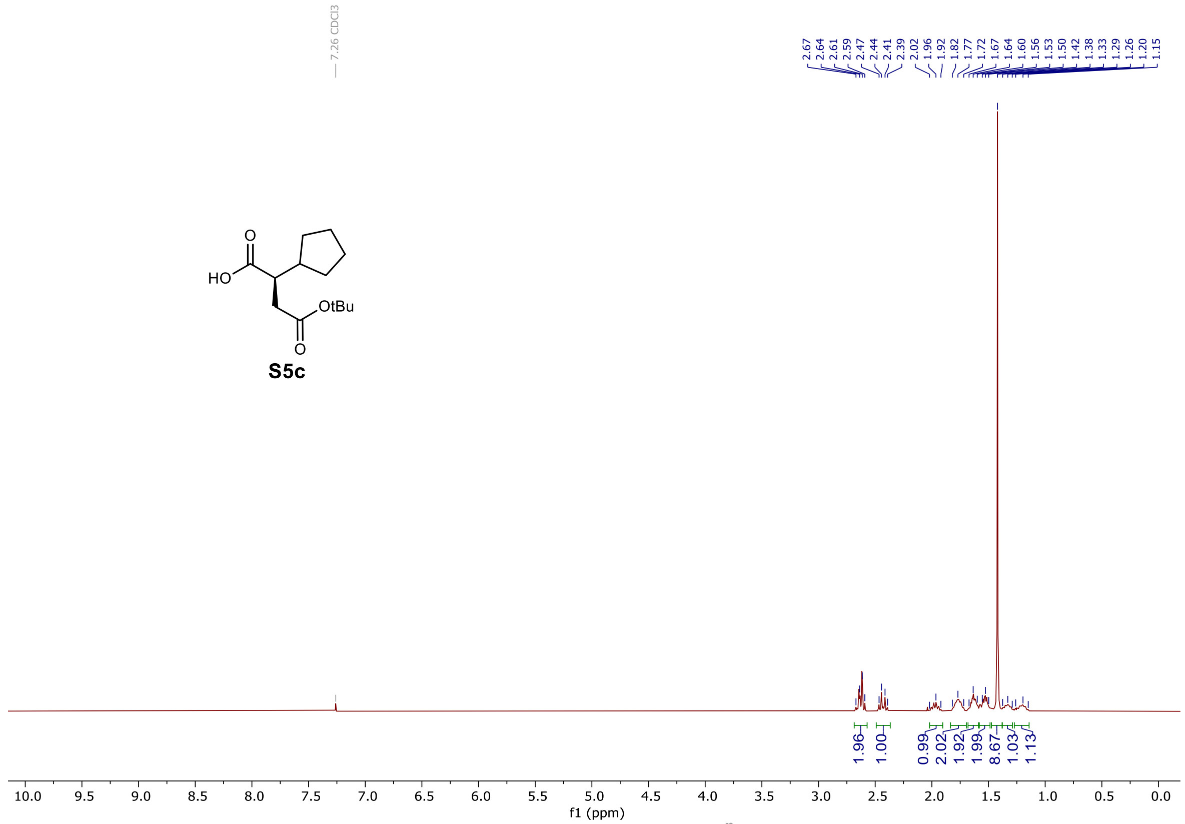

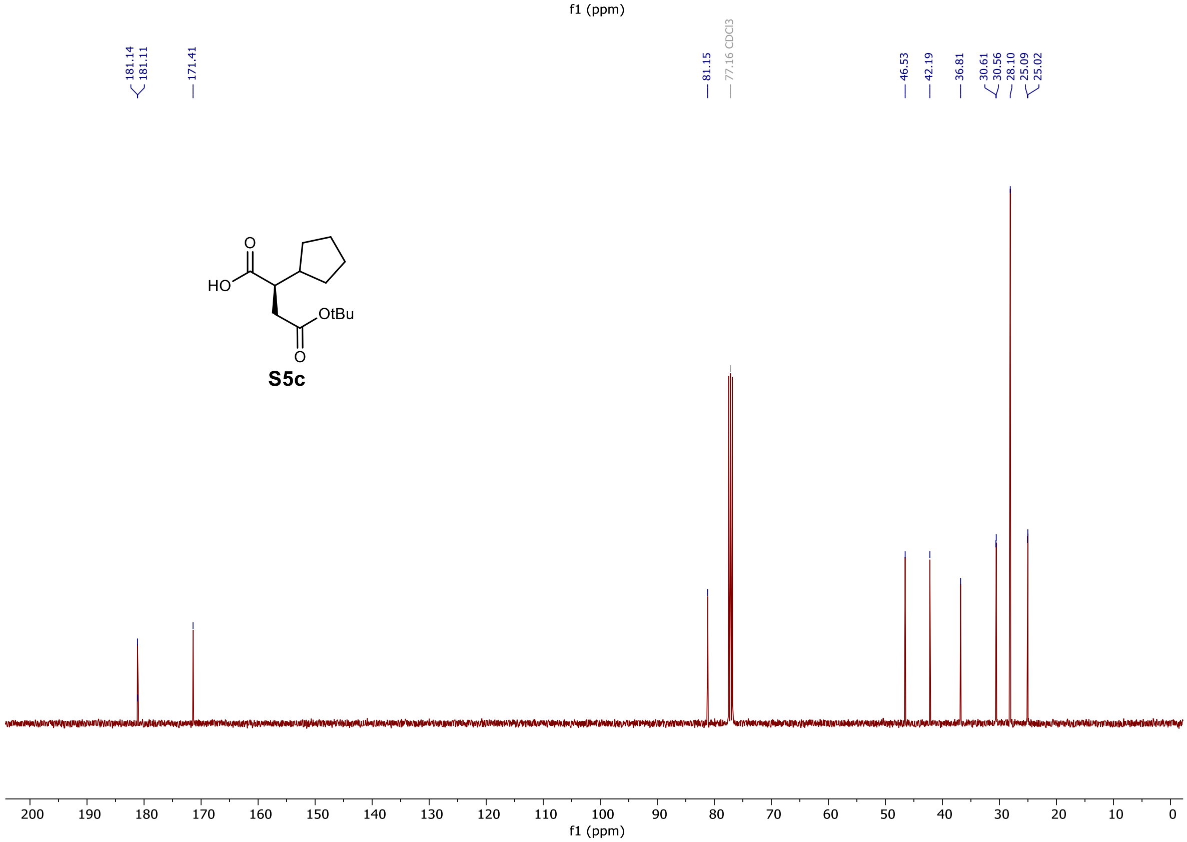

Supplementary Fig. 28. NMR of compound S5c.

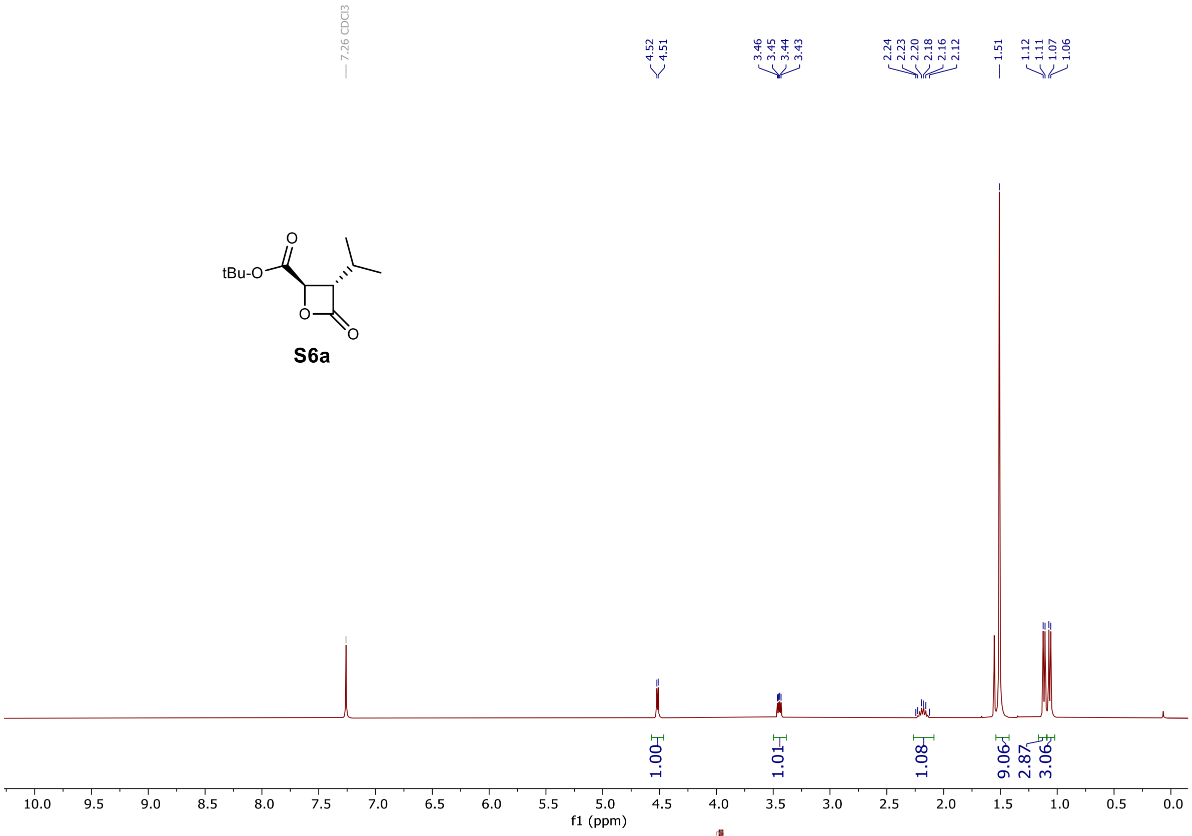

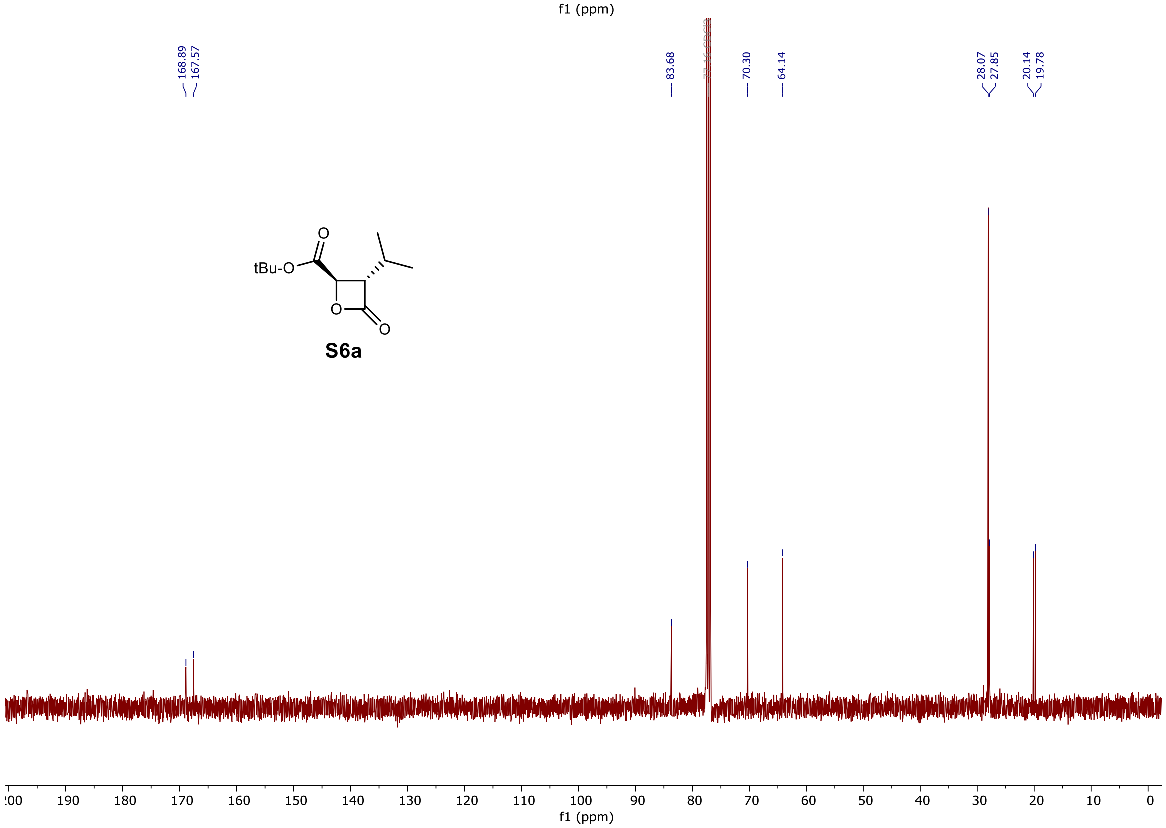

Supplementary Fig. 29. NMR of compound S6a.

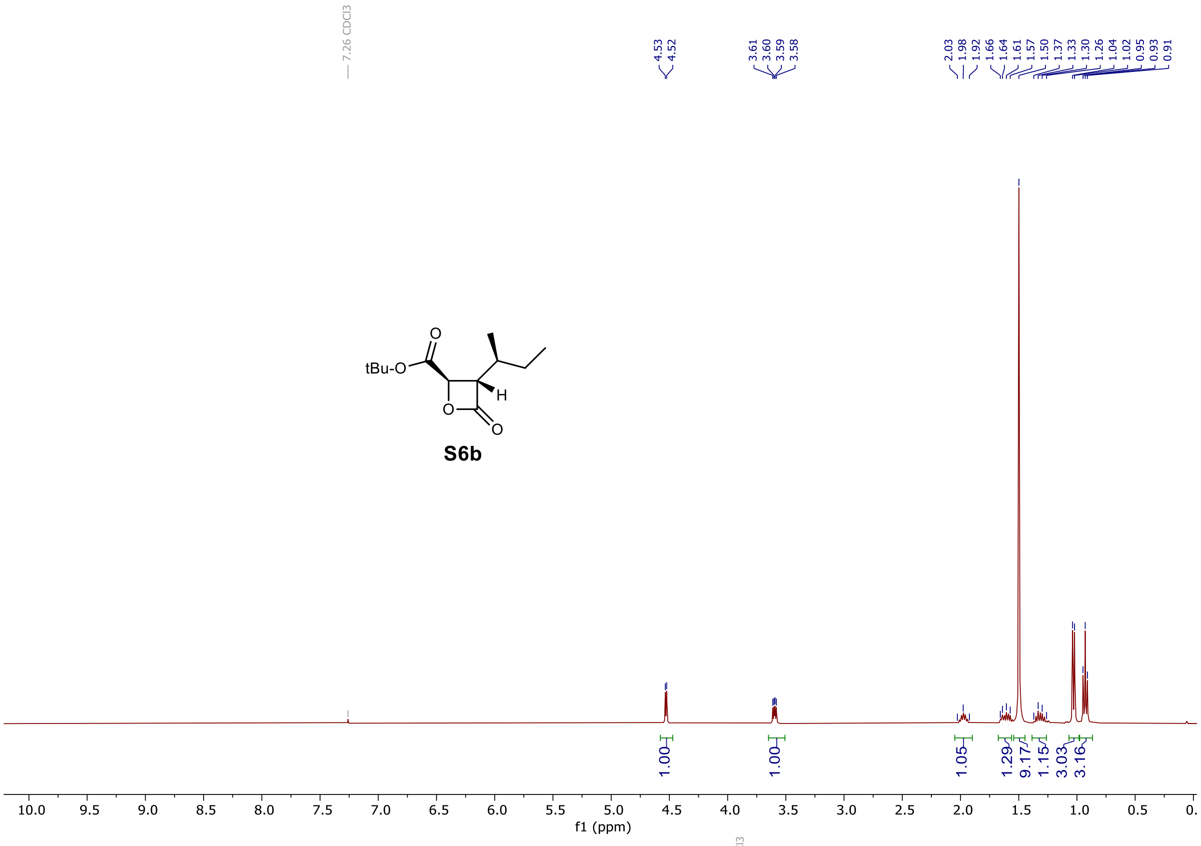

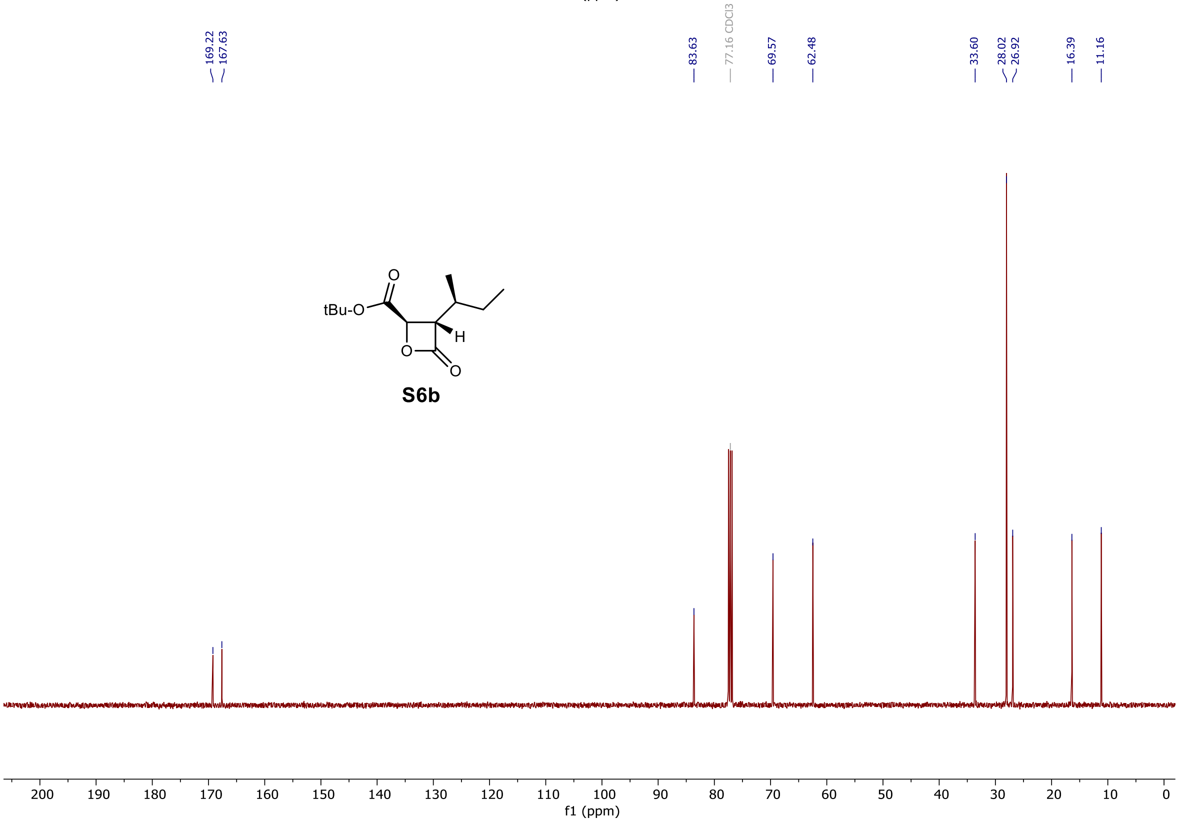

Supplementary Fig. 30. NMR of compound S6b.

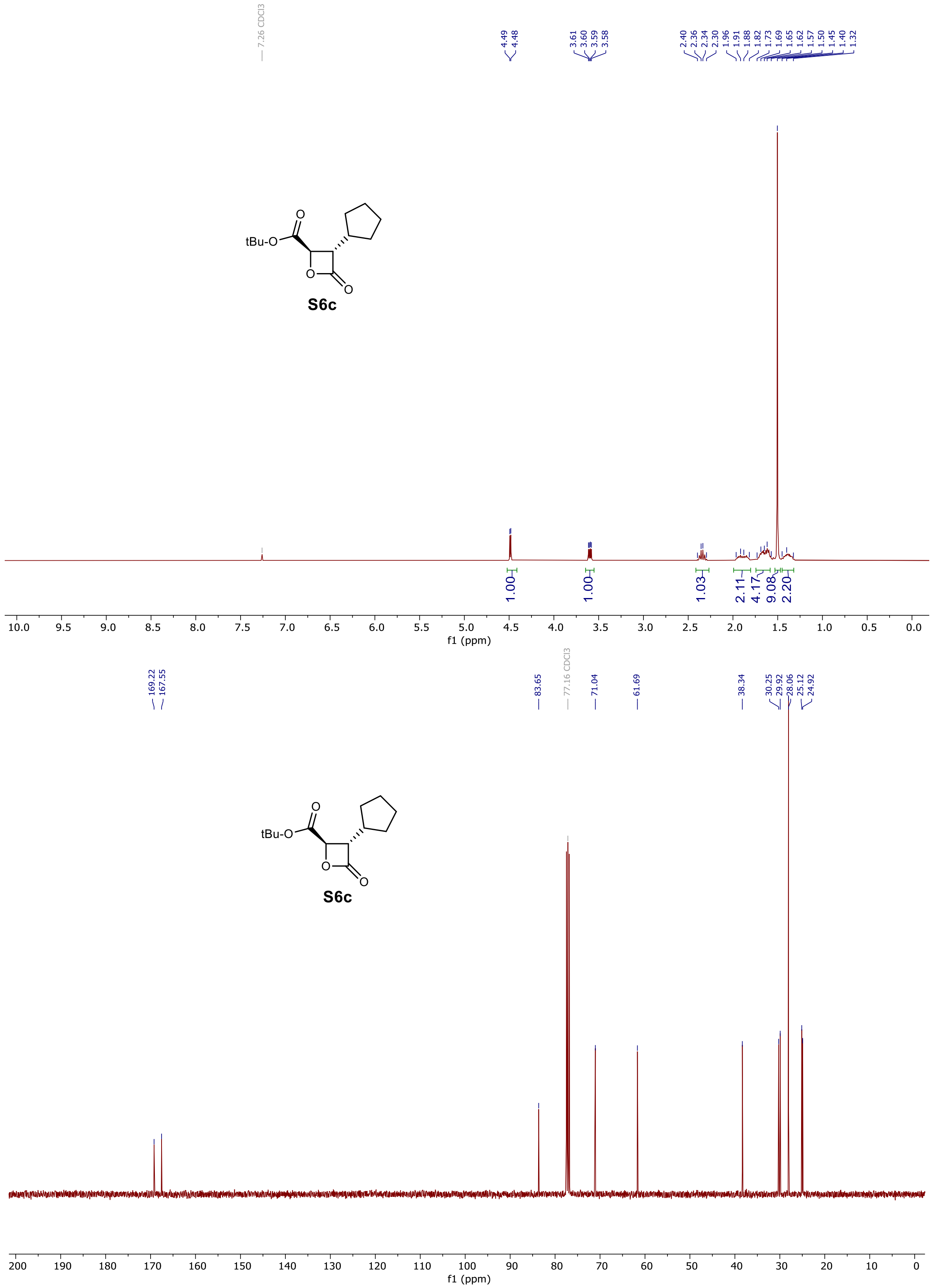

Supplementary Fig. 31. NMR of compound S6c.

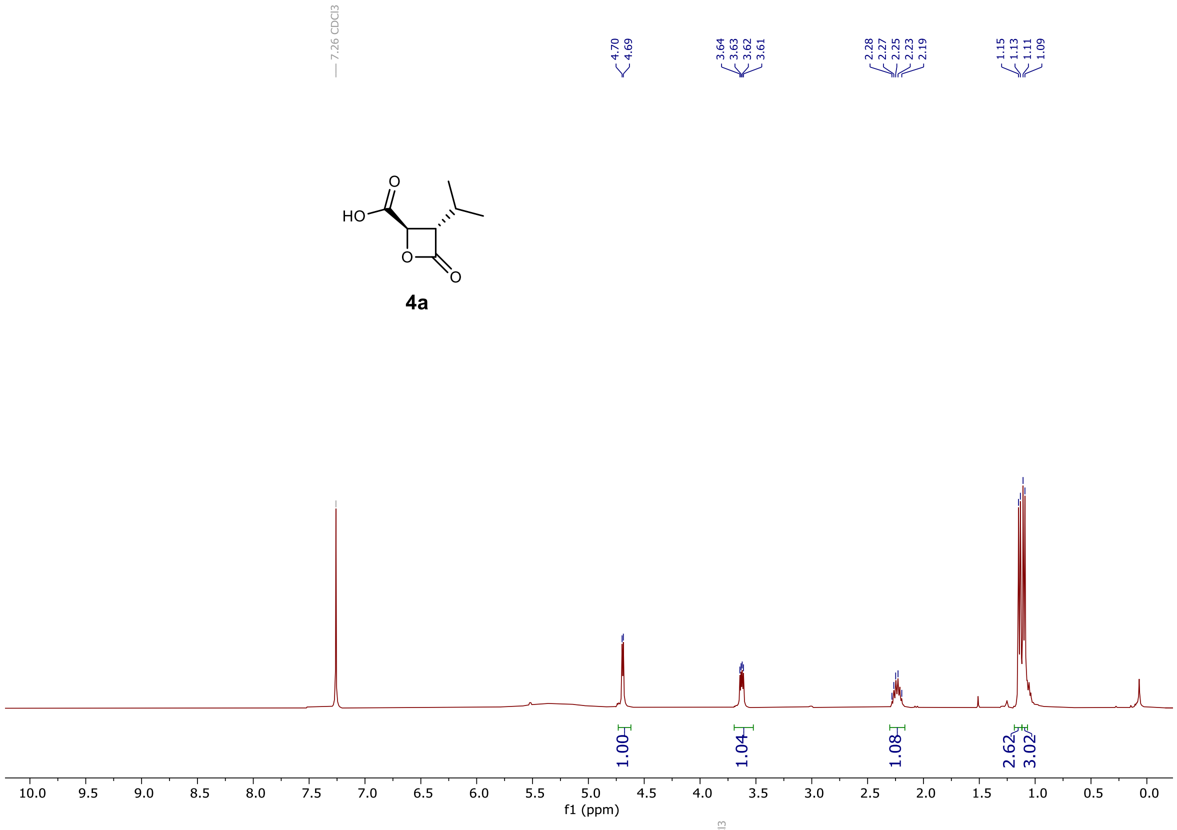

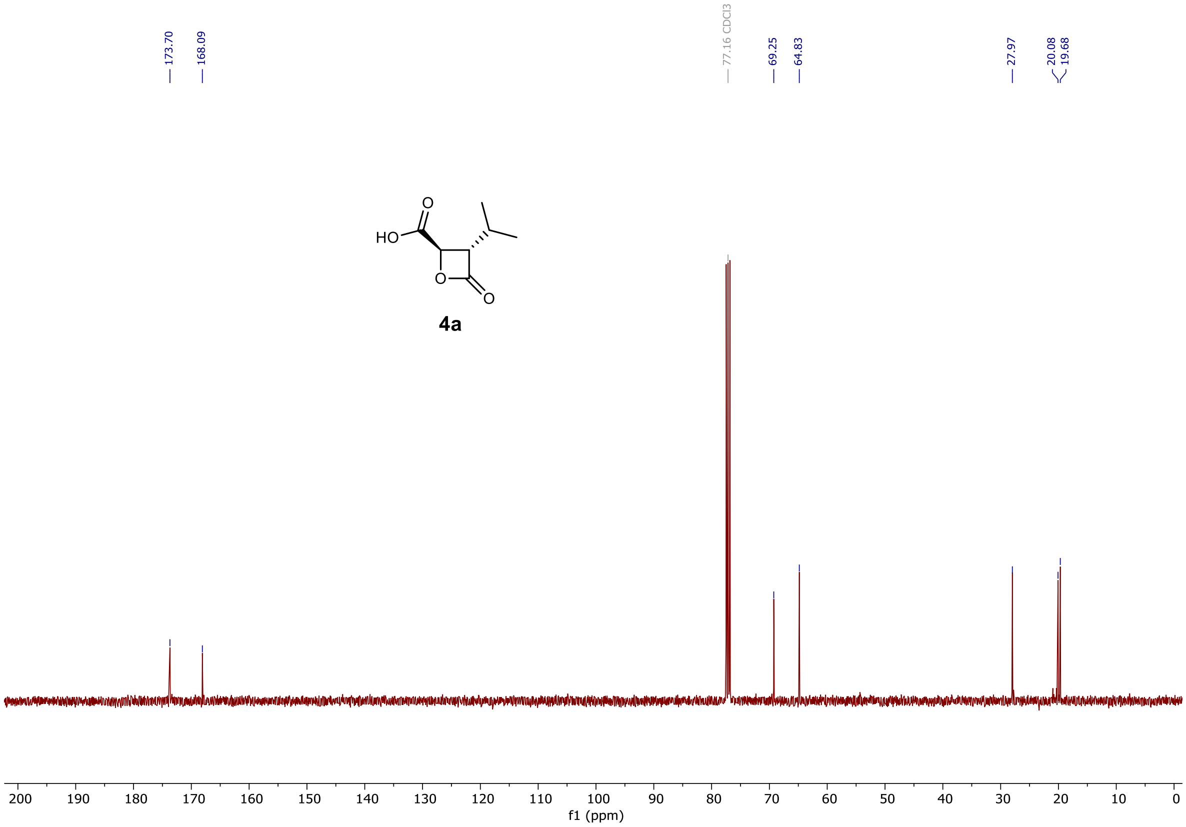

Supplementary Fig. 32. NMR of compound 4a.

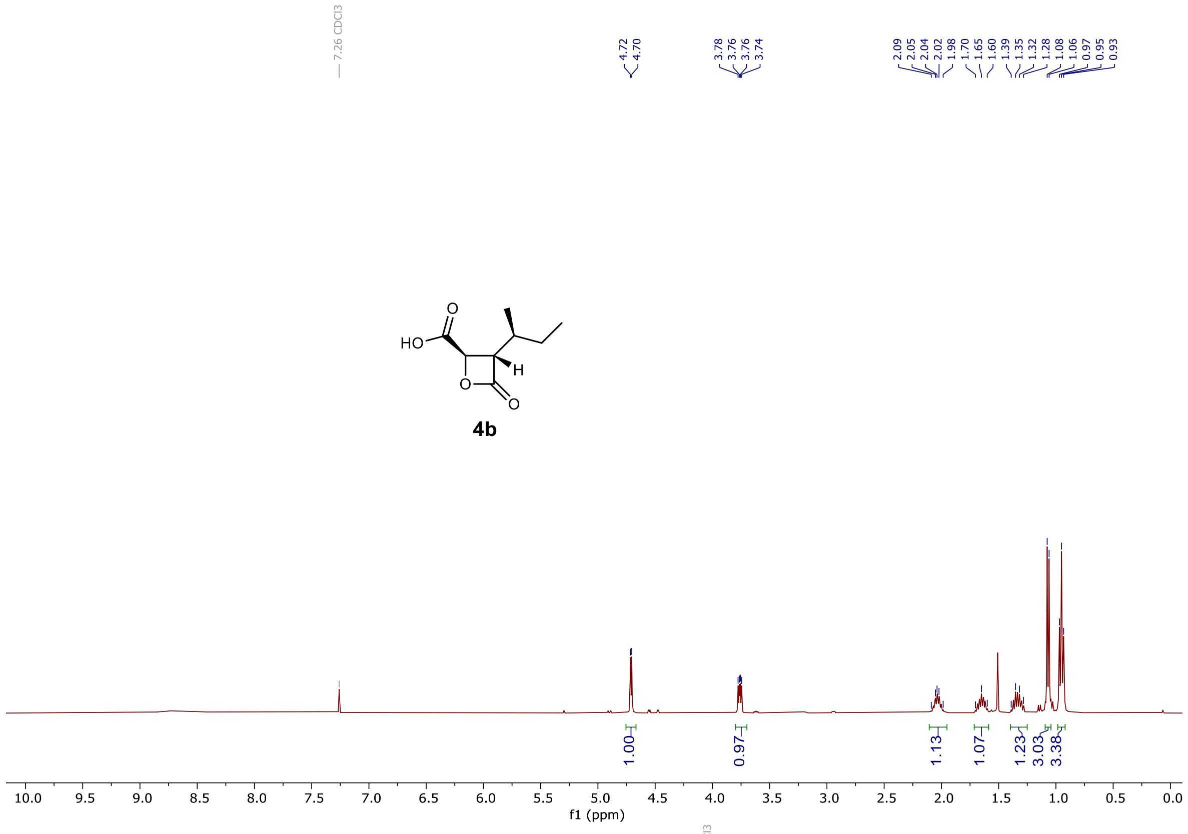

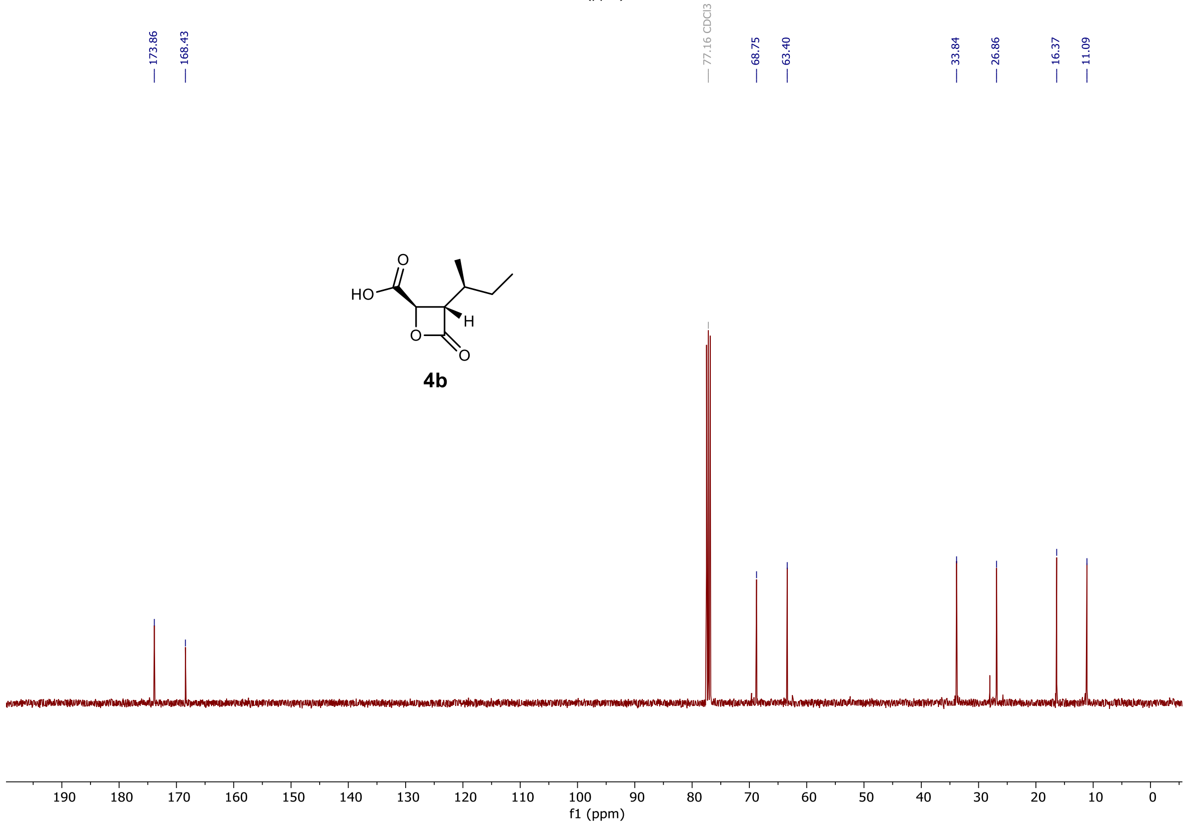

Supplementary Fig. 33. NMR of compound 4b.

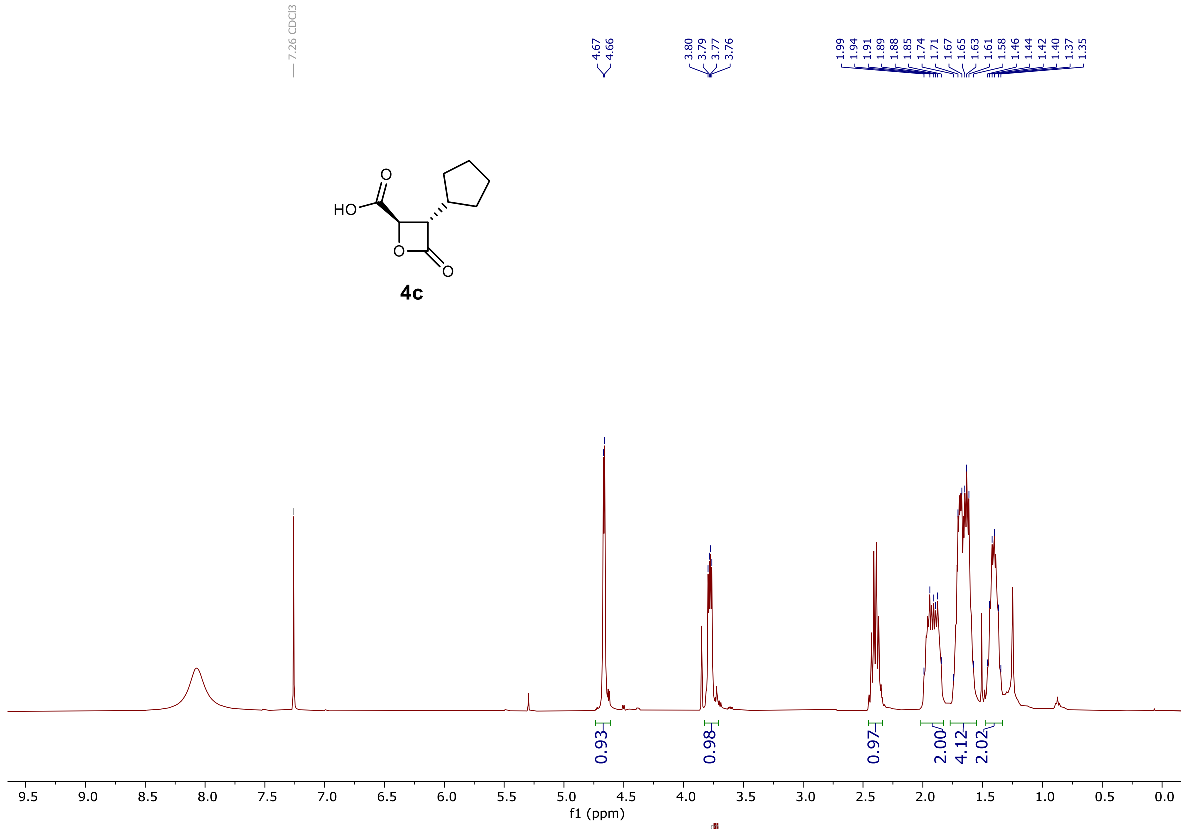

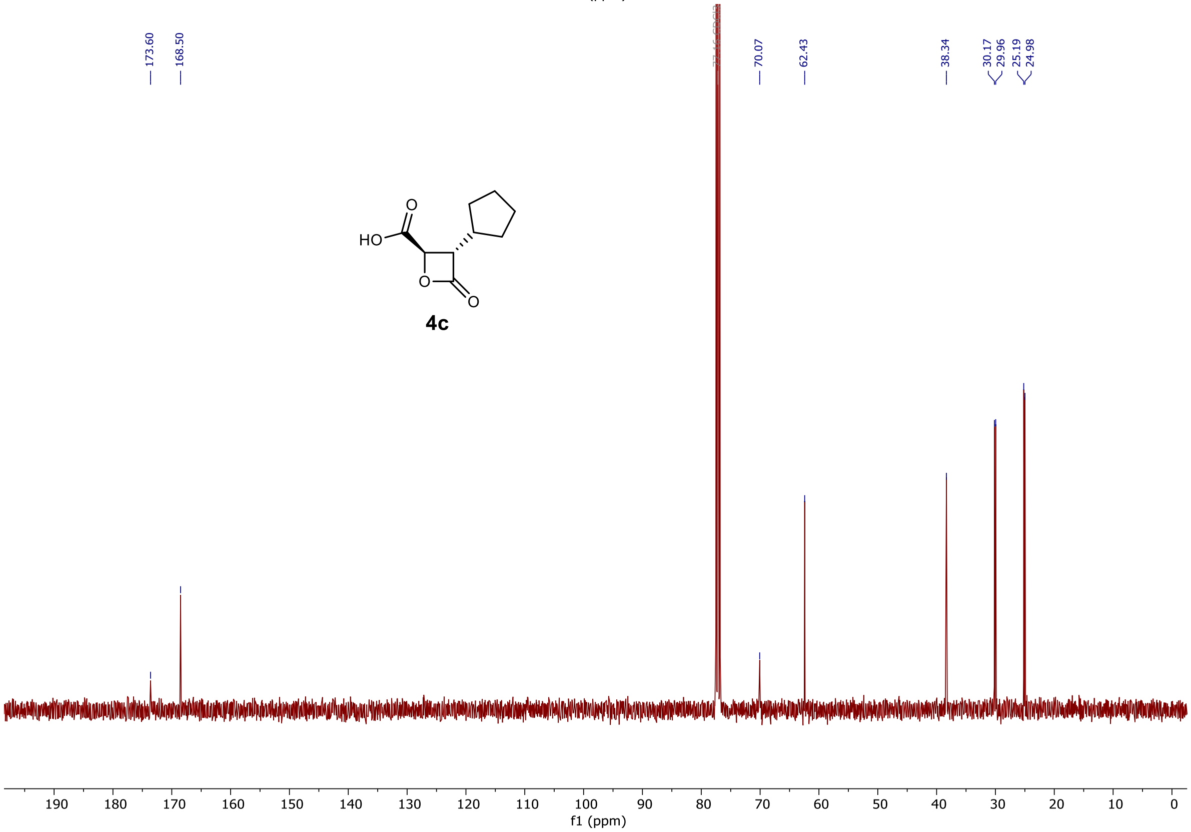

Supplementary Fig. 34. NMR of compound 4c.

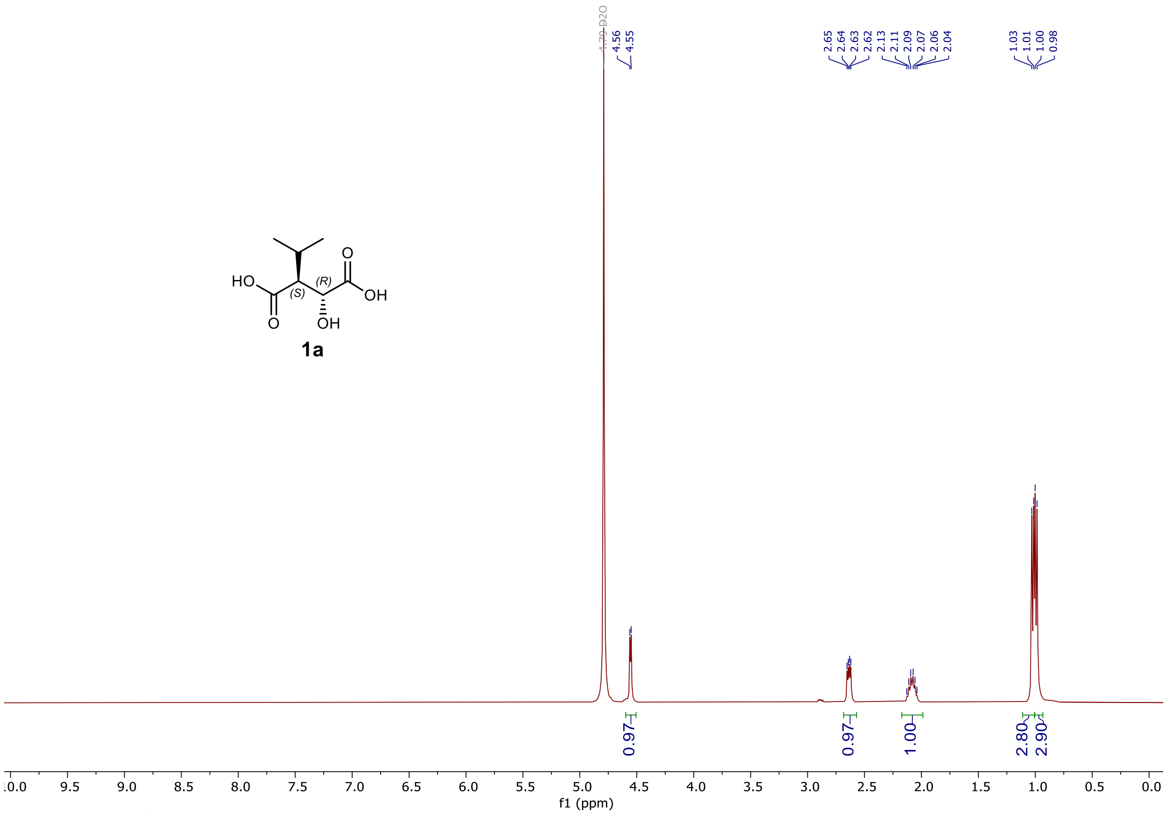

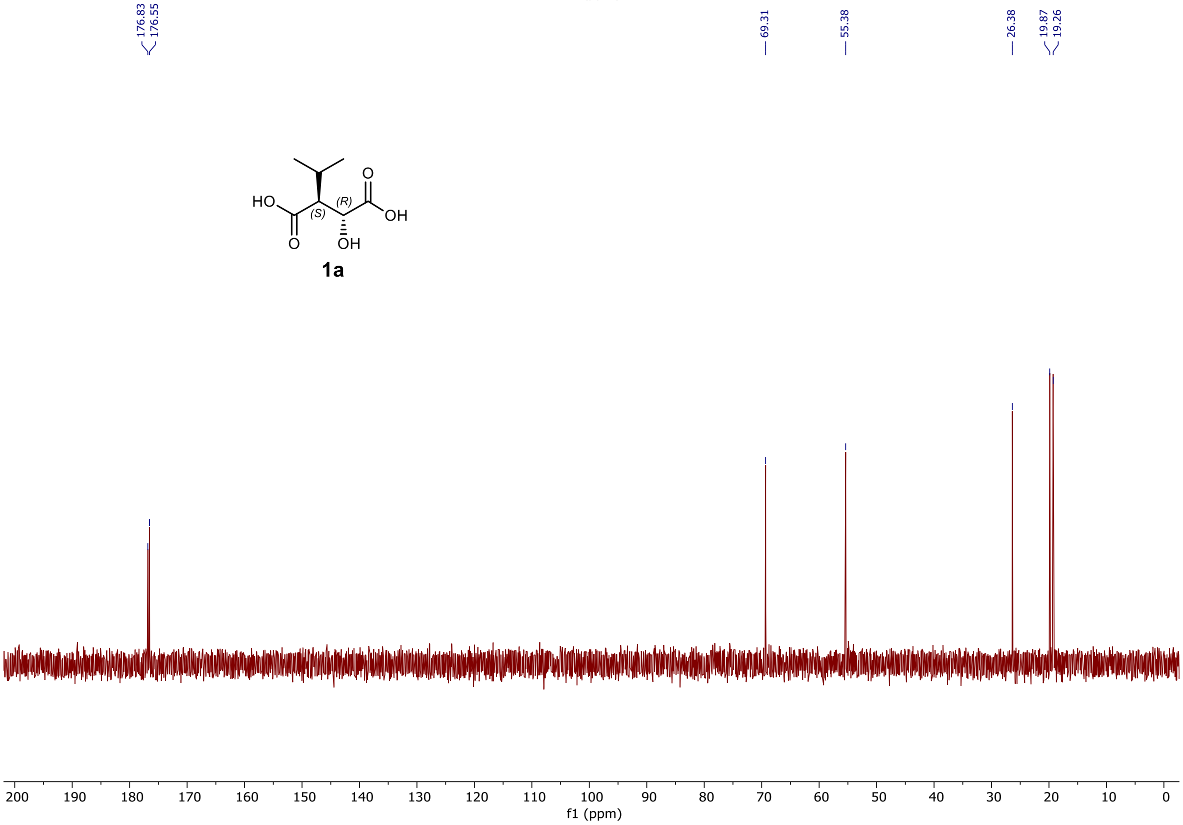

Supplementary Fig. 35. NMR of compound 1a.

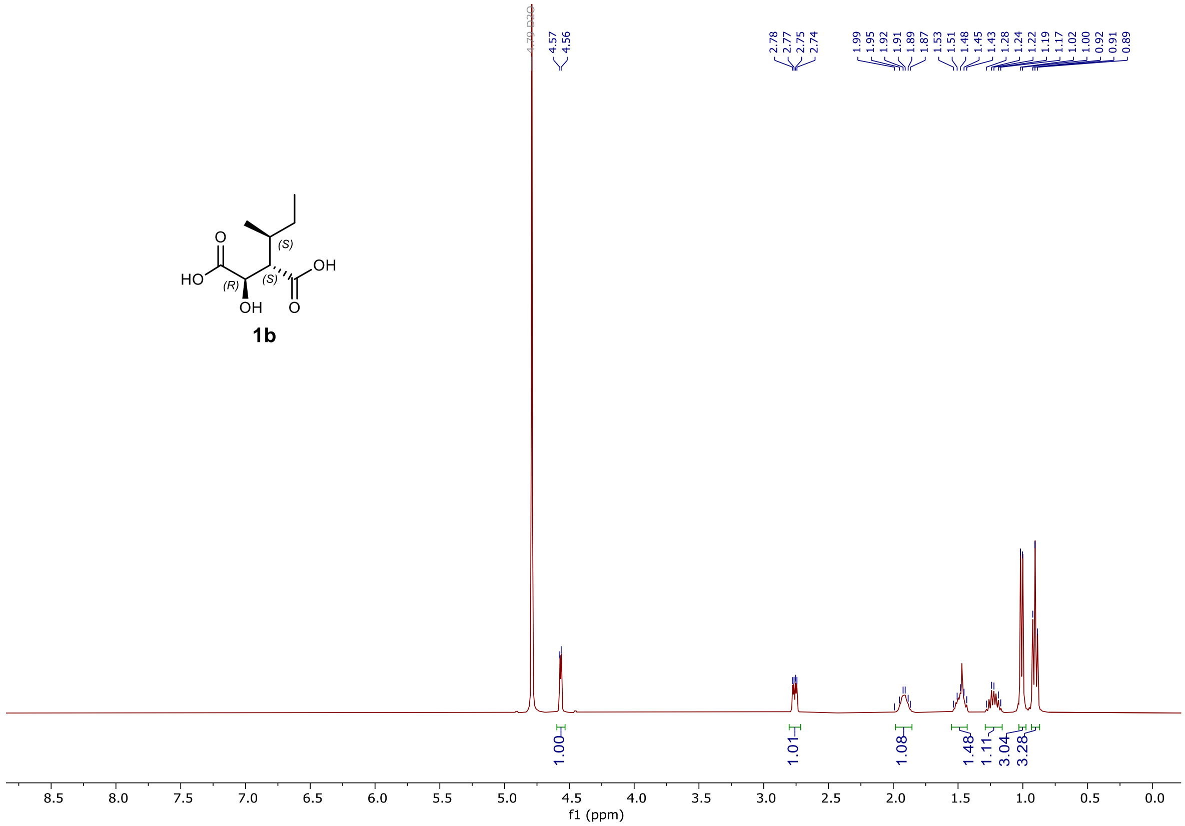

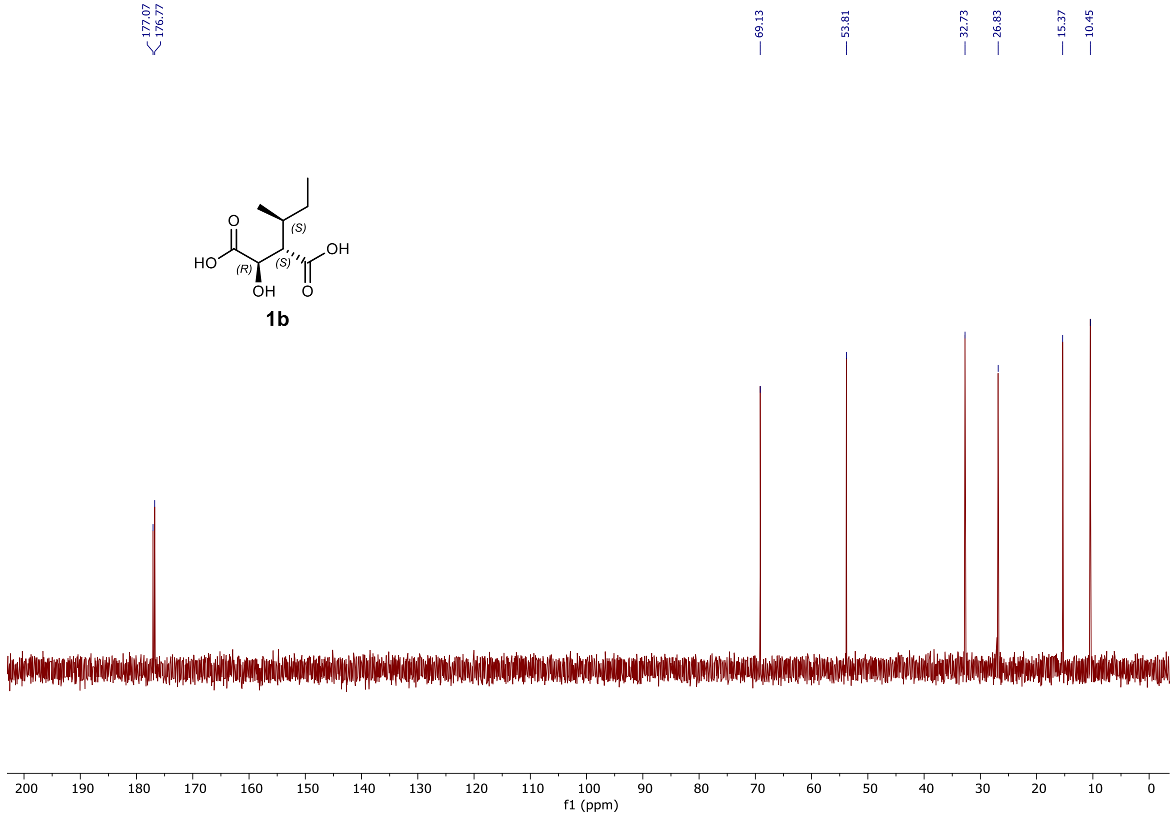

Supplementary Fig. 36. NMR of compound 1b.

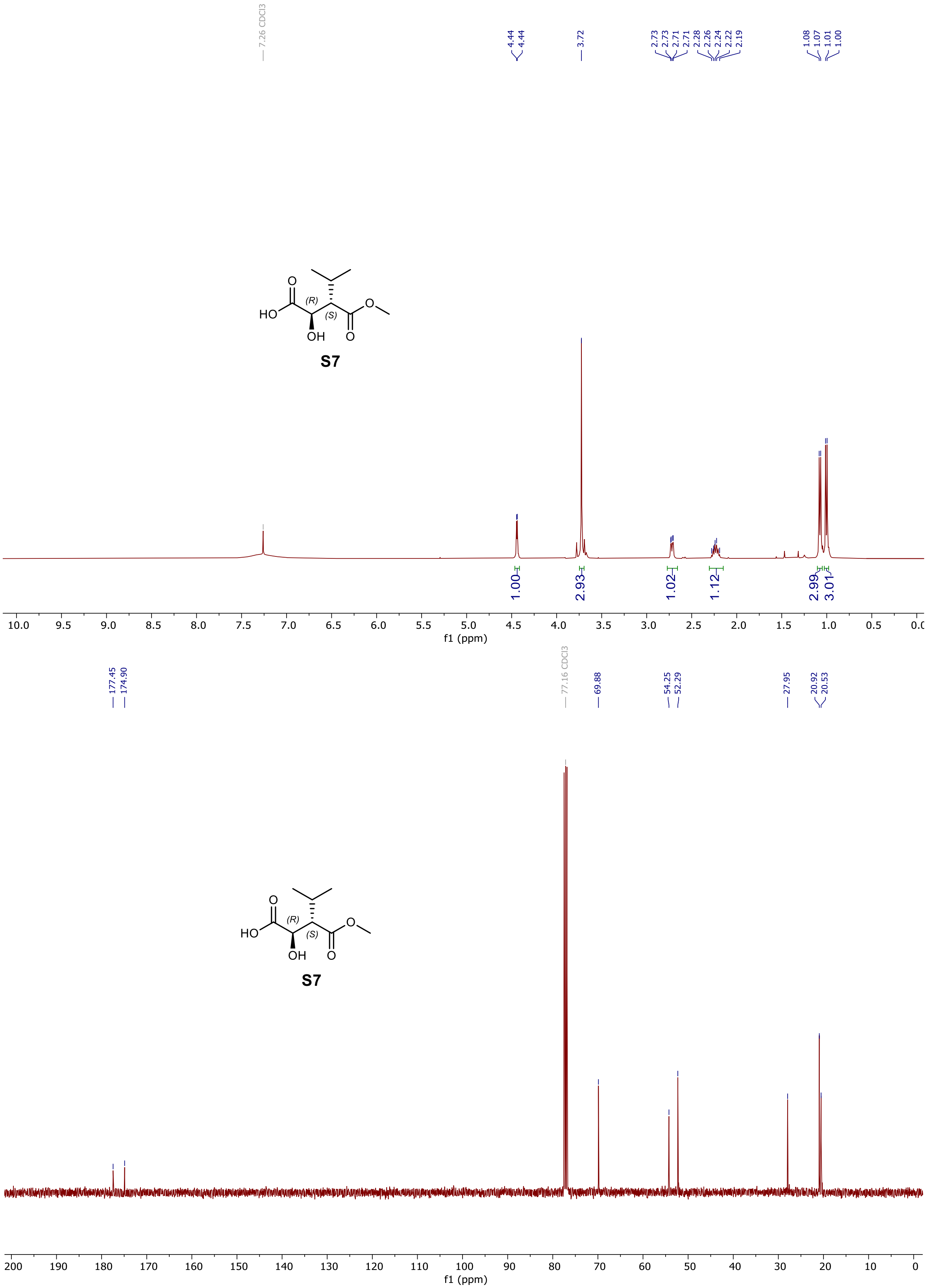

Supplementary Fig. 37. NMR of compound S7.

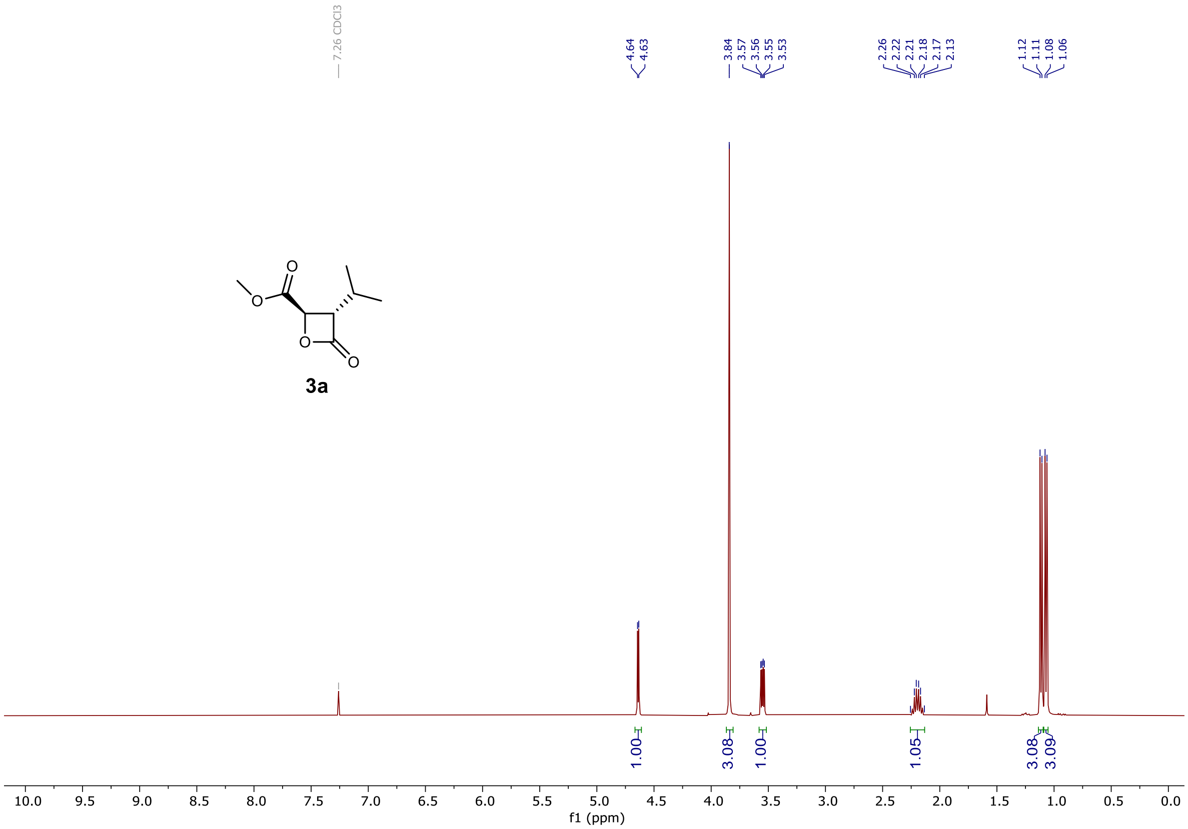

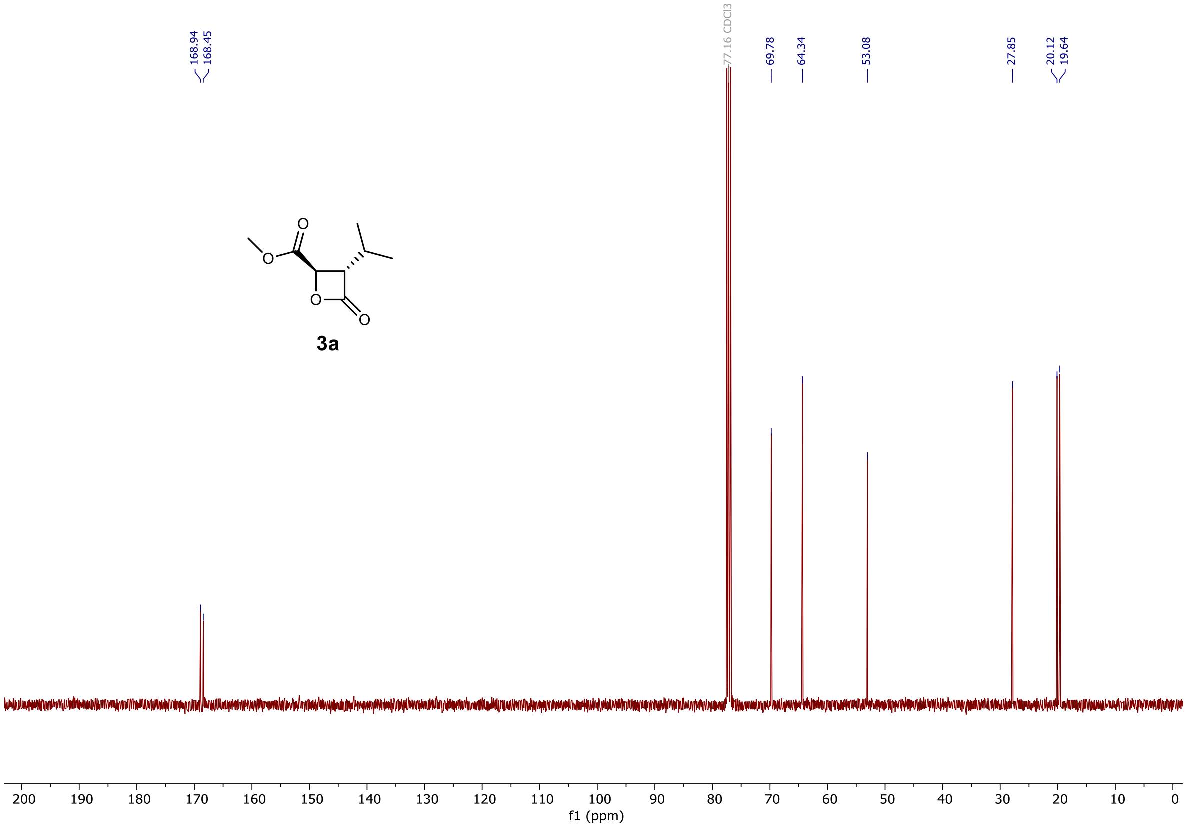

Supplementary Fig. 38. NMR of compound 3a.

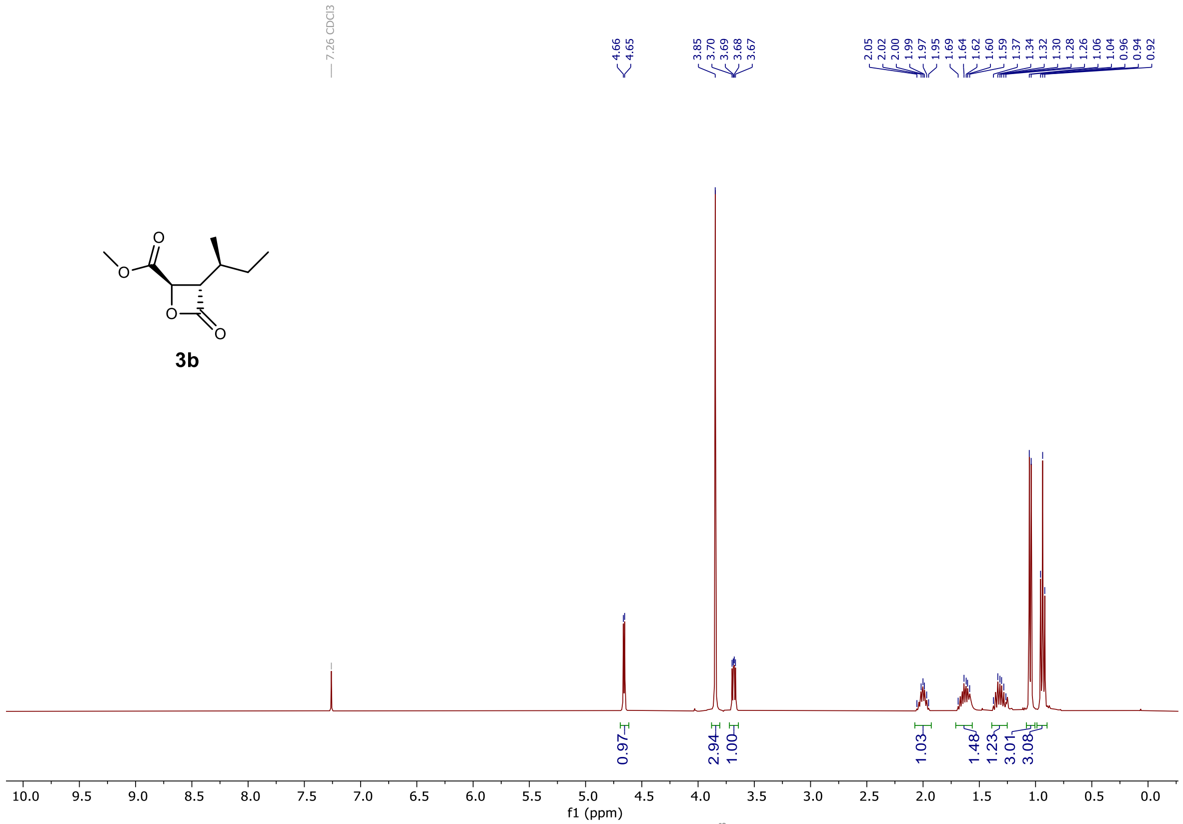

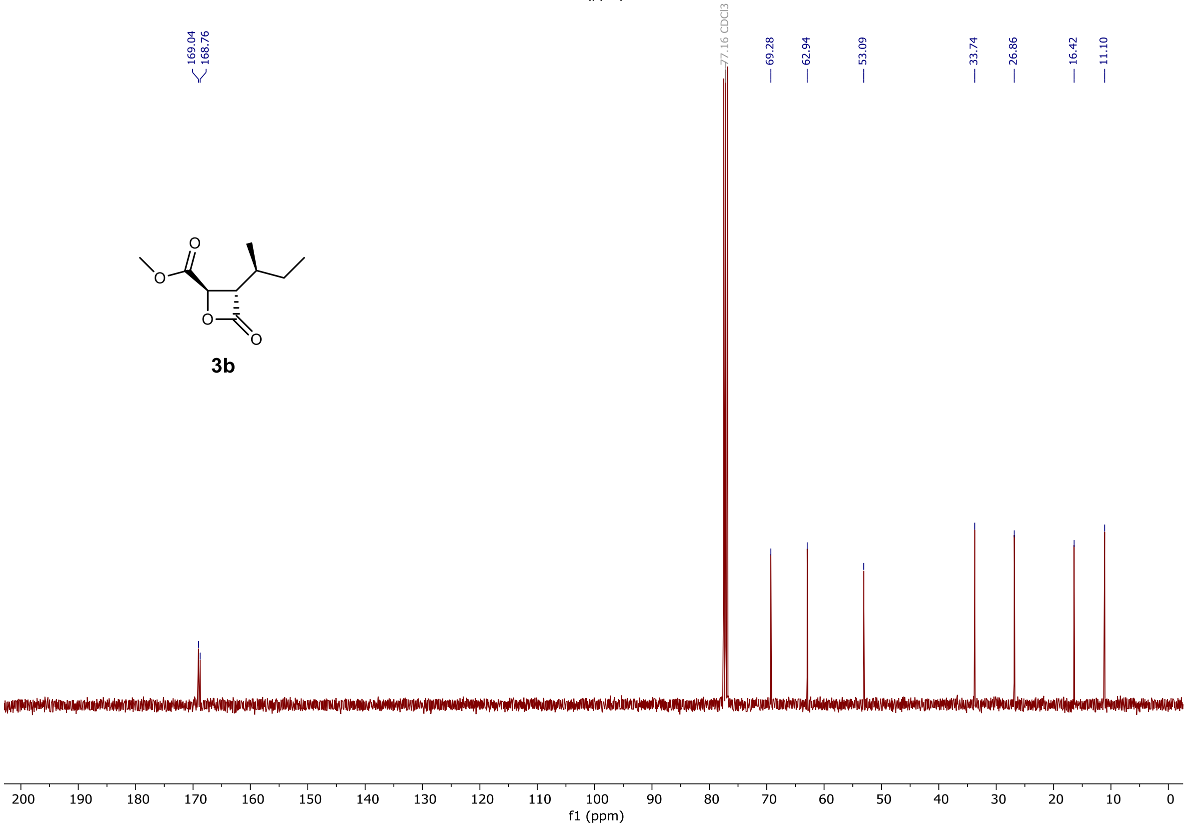

Supplementary Fig. 39. NMR of compound 3b.

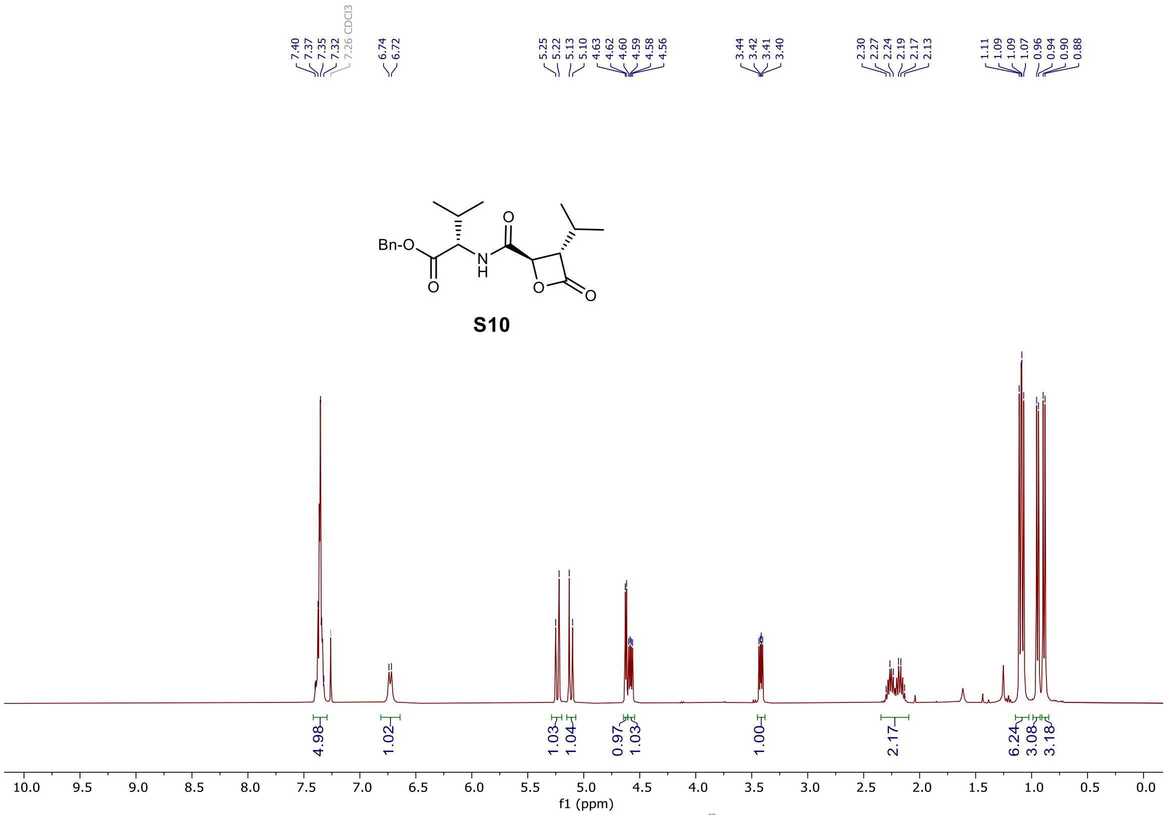

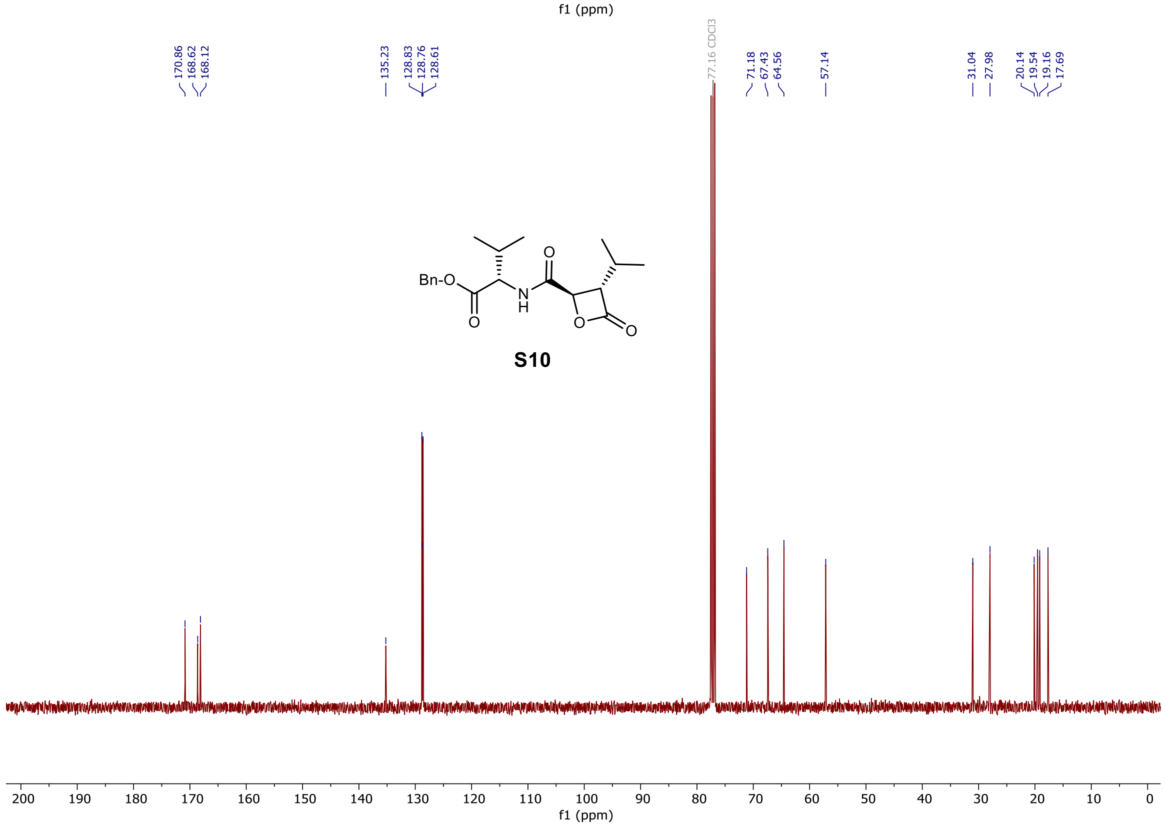

Supplementary Fig. 40. NMR of compound S10.

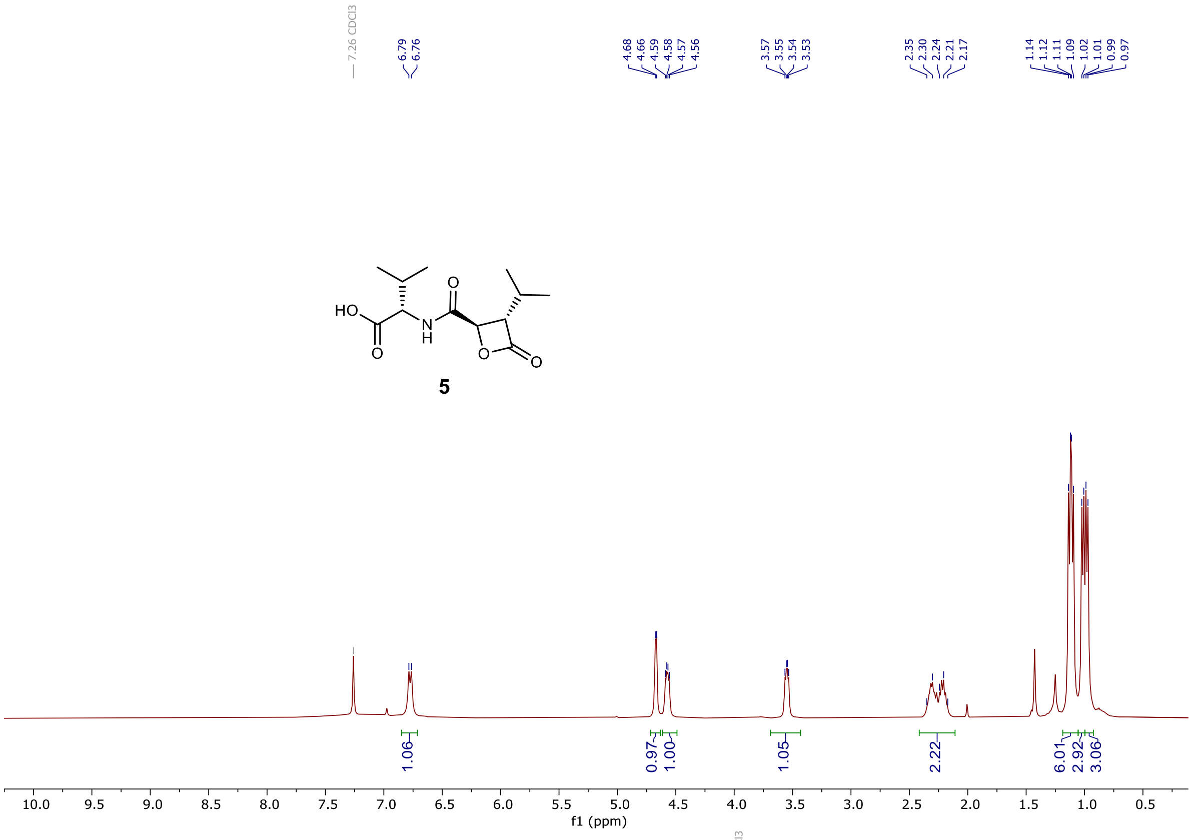

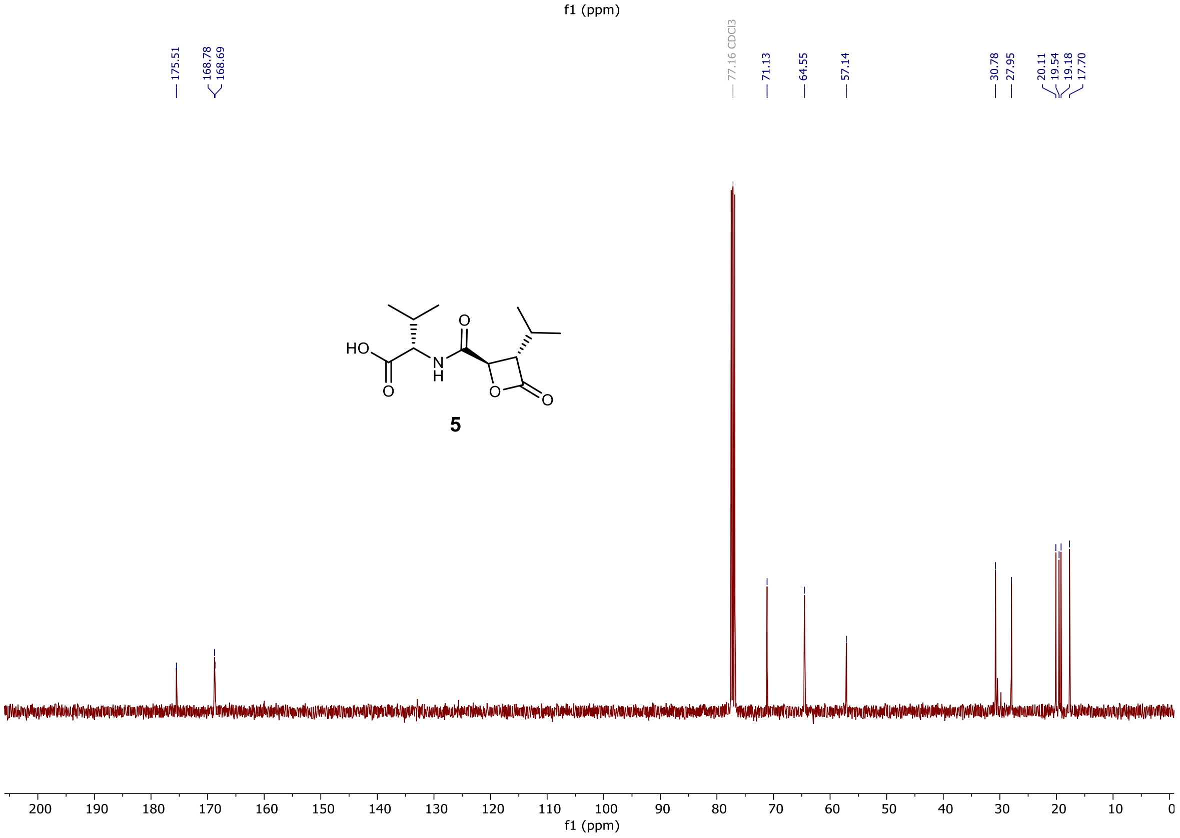

Supplementary Fig. 41. NMR of compound 5.

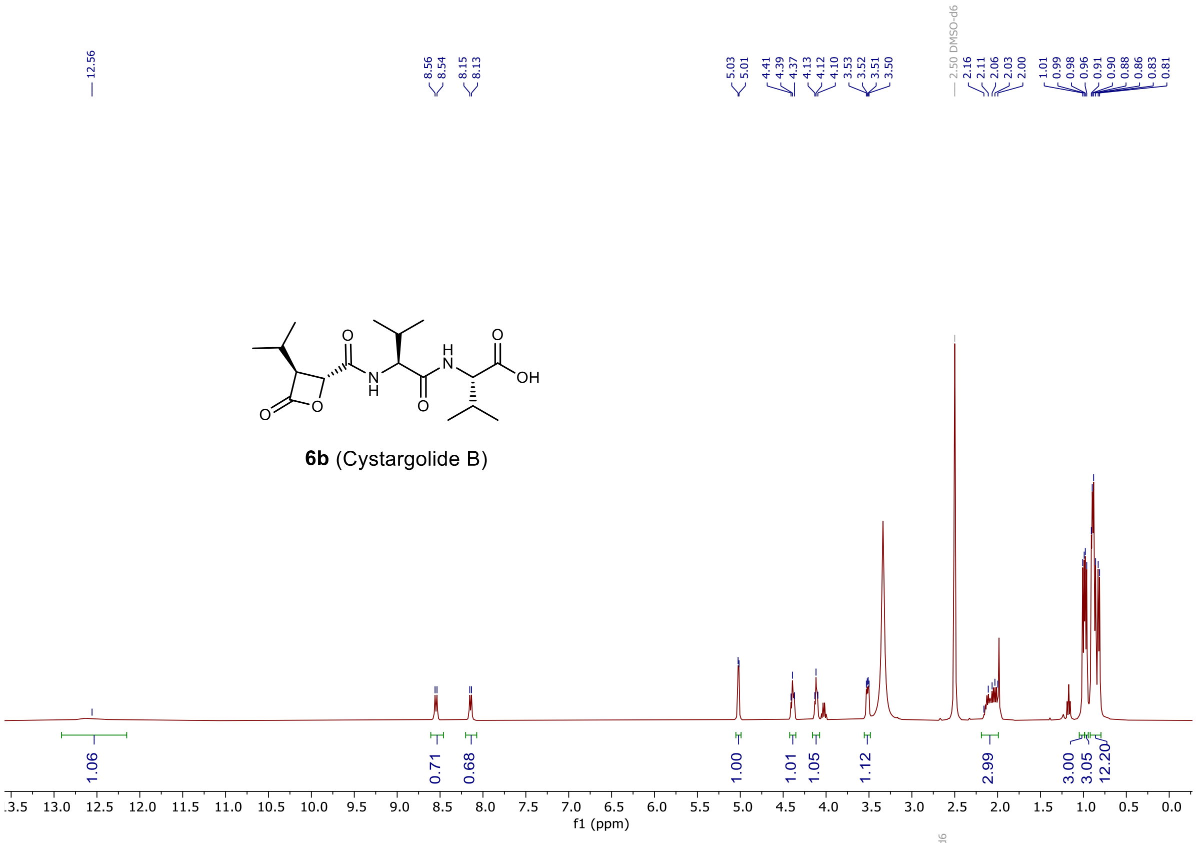

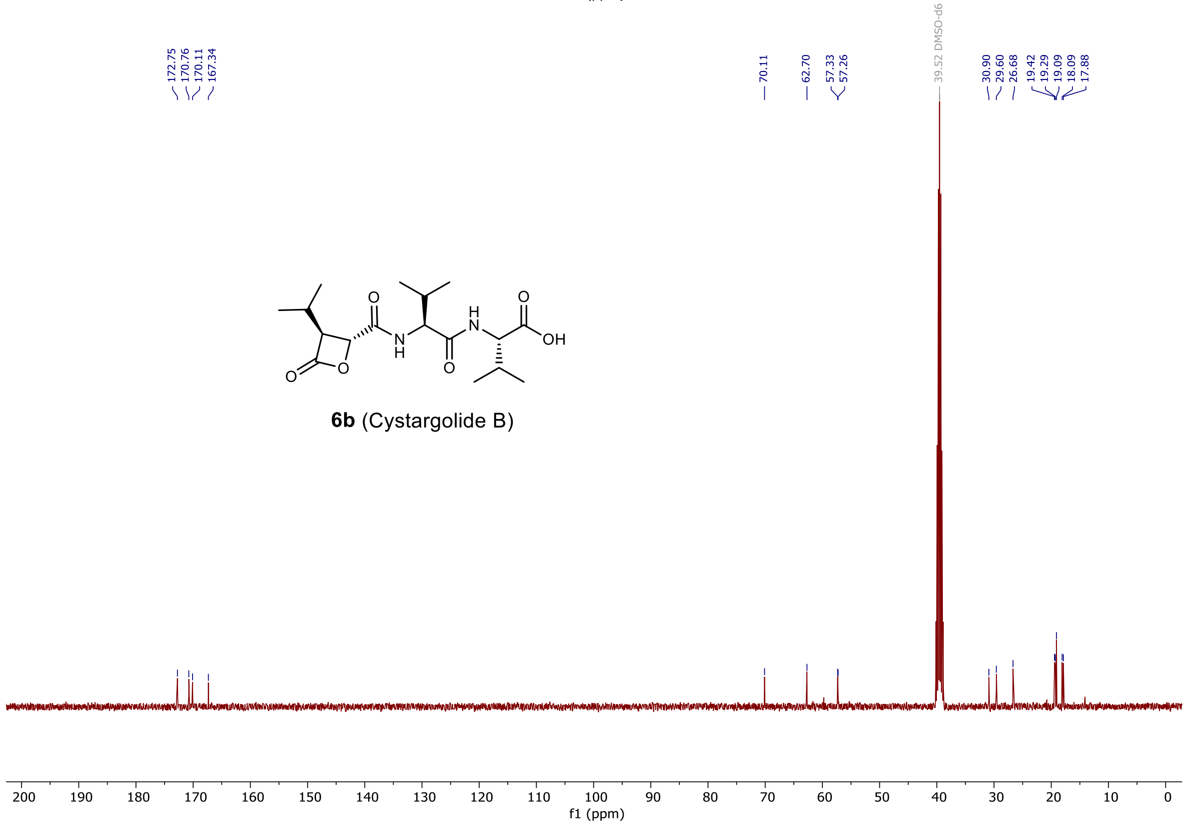

Supplementary Fig. 42. NMR of compound 6b.

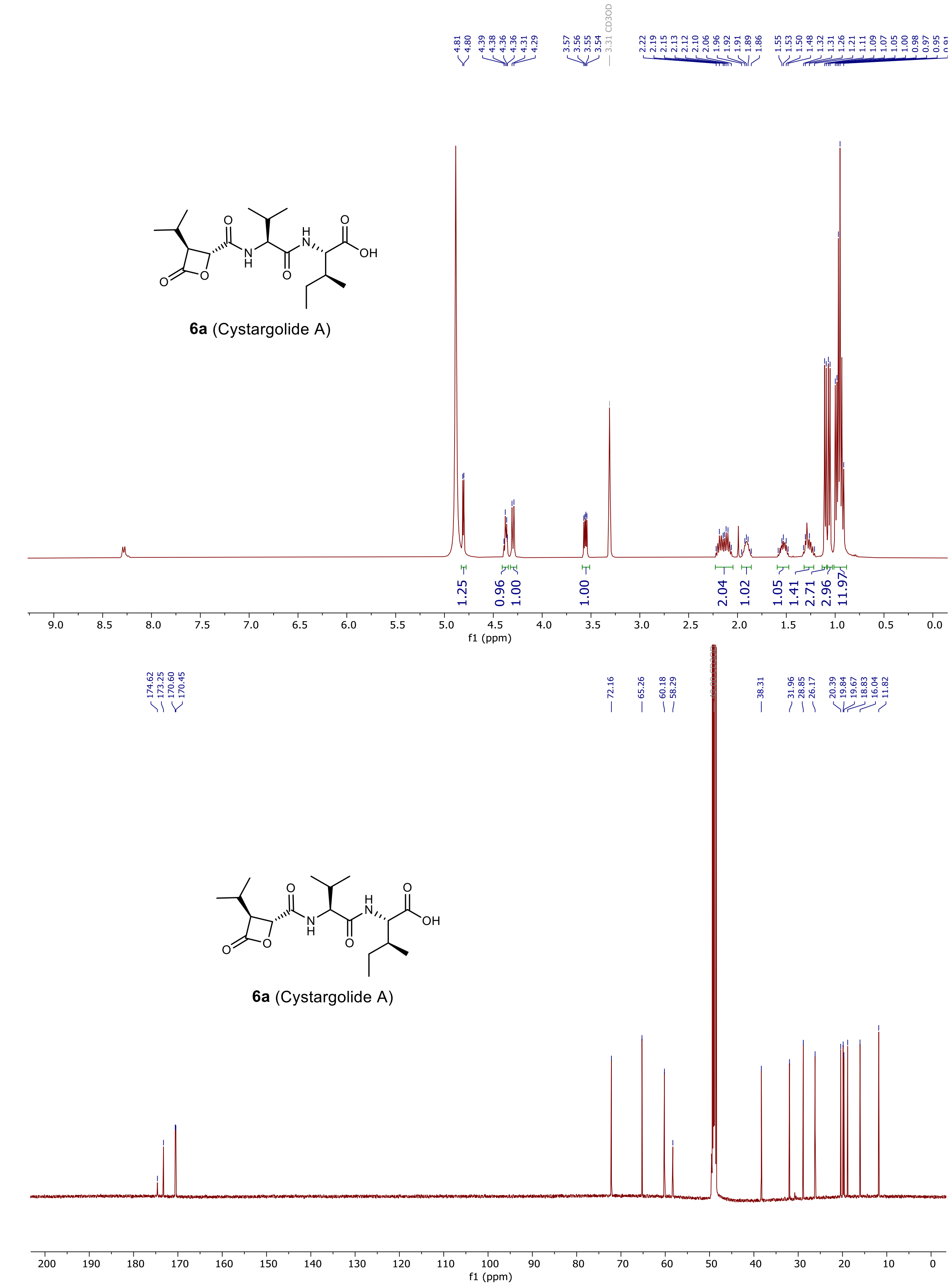

Supplementary Fig. 43. NMR of compound 6a.

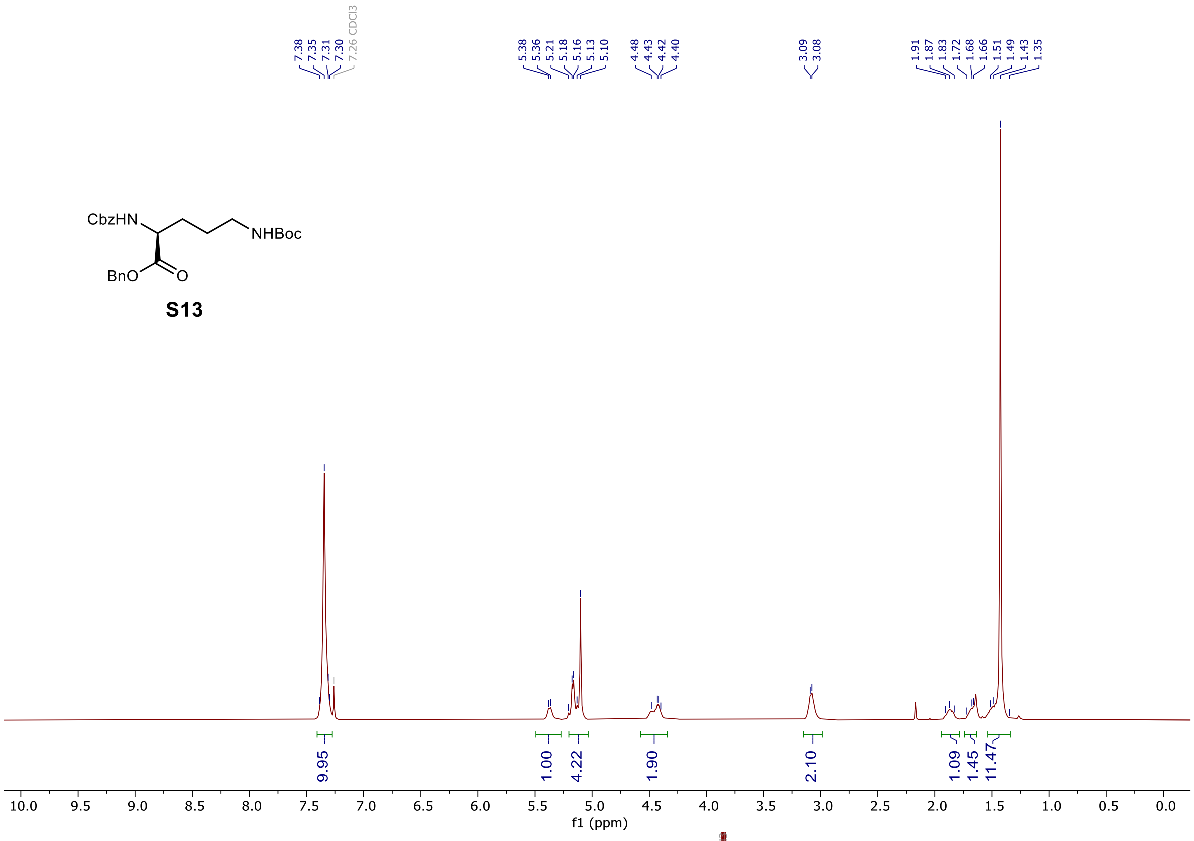

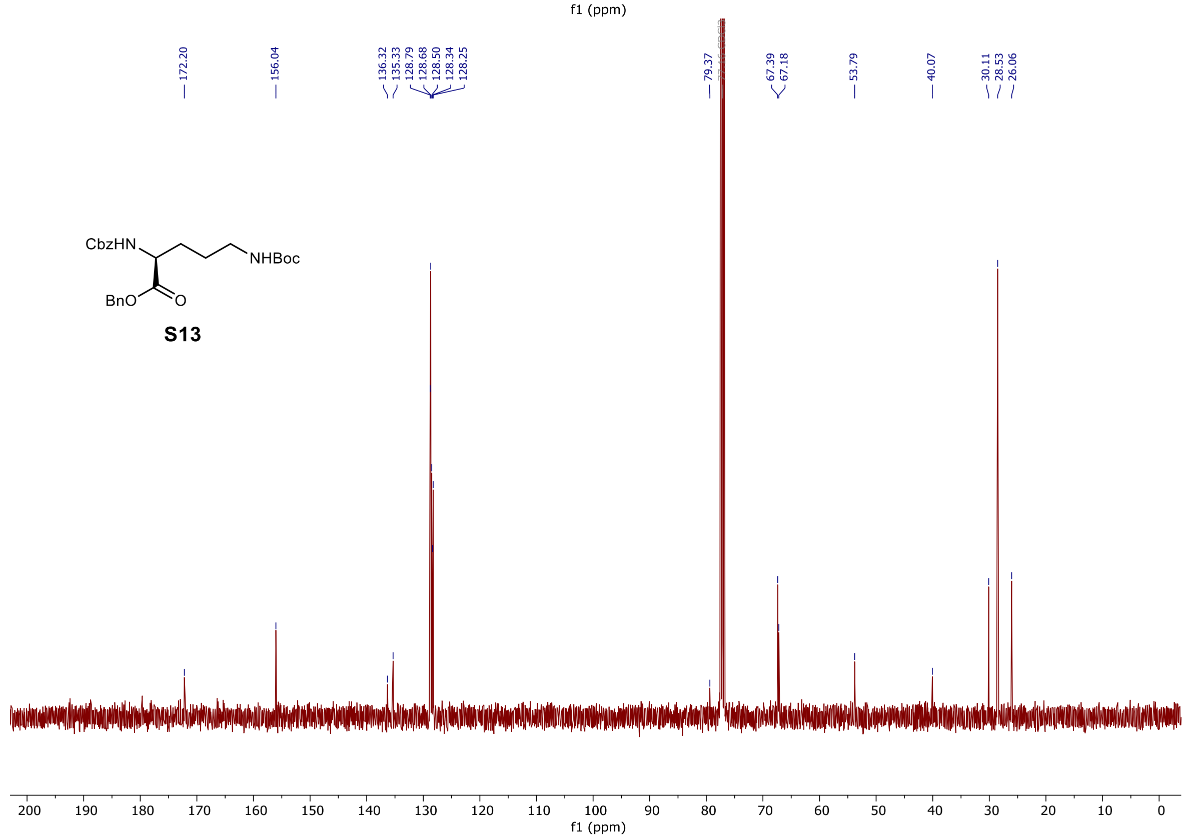

Supplementary Fig. 44. NMR of compound S13.

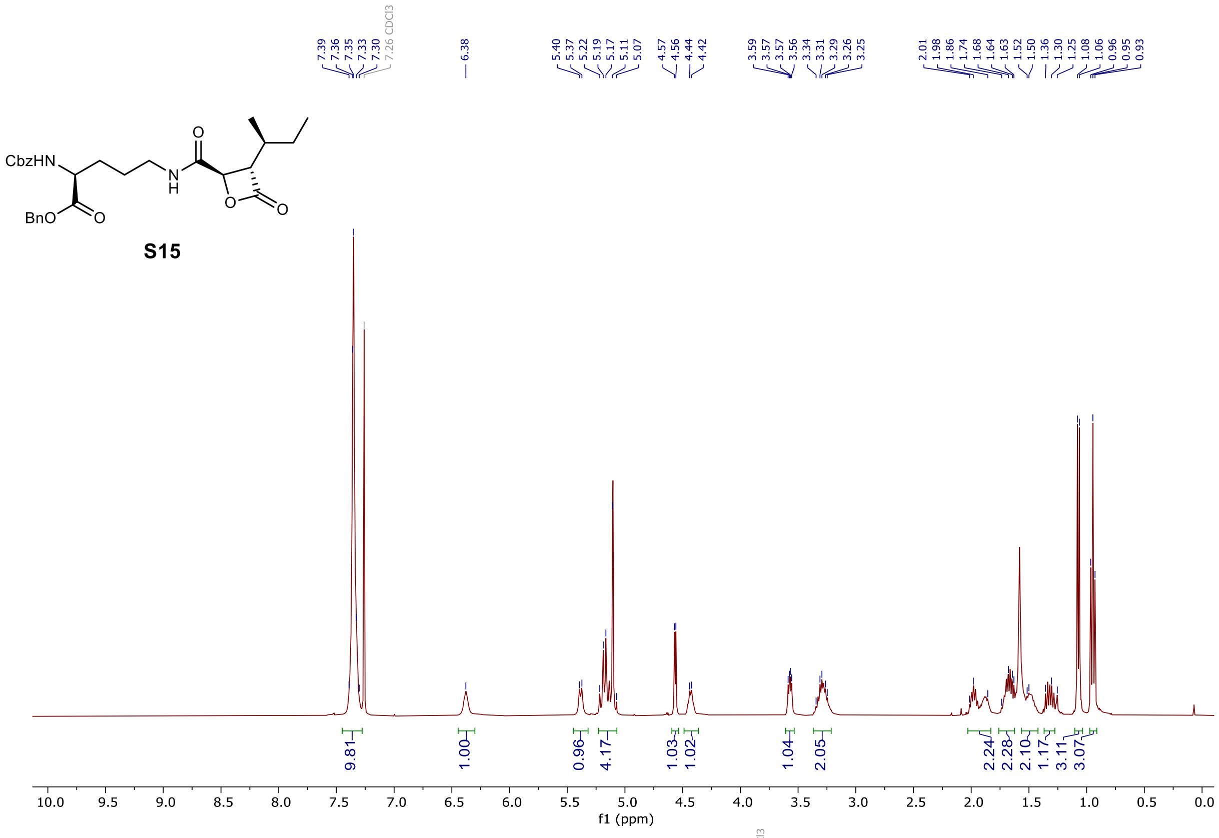

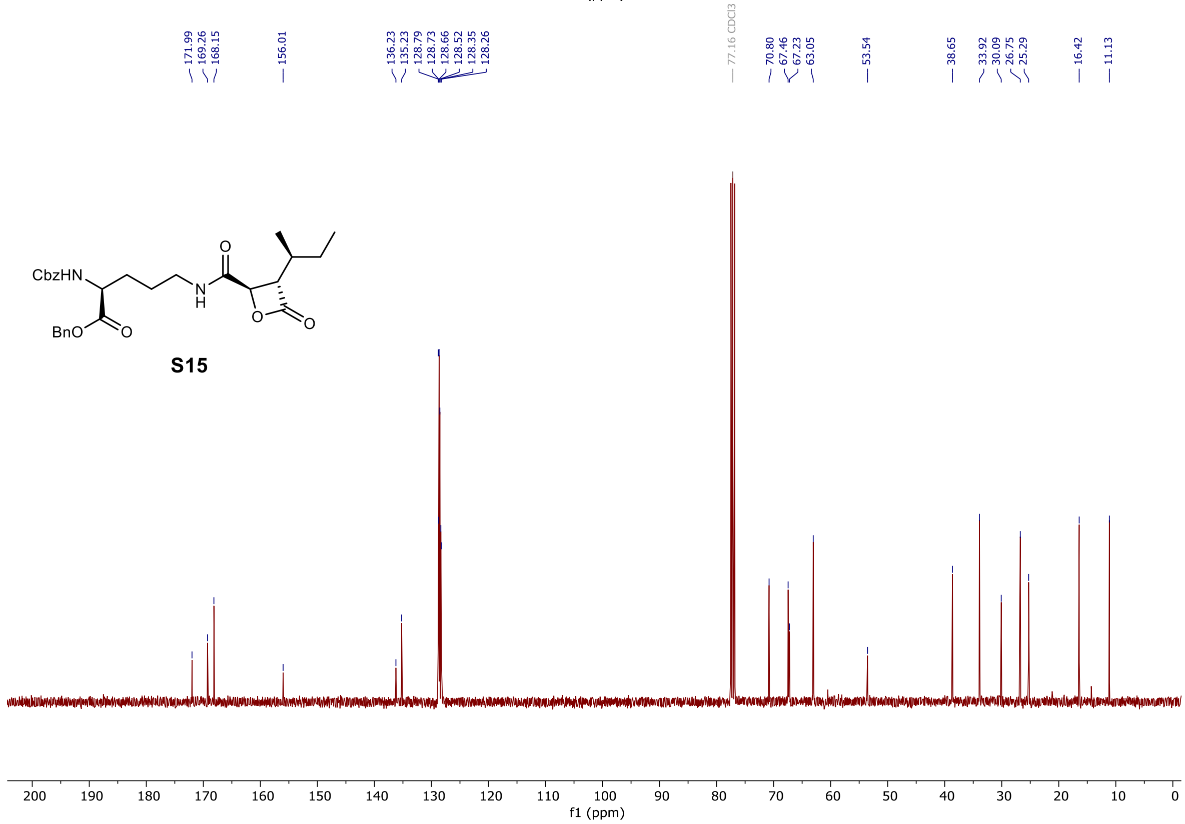

Supplementary Fig. 45. NMR of compound S15.

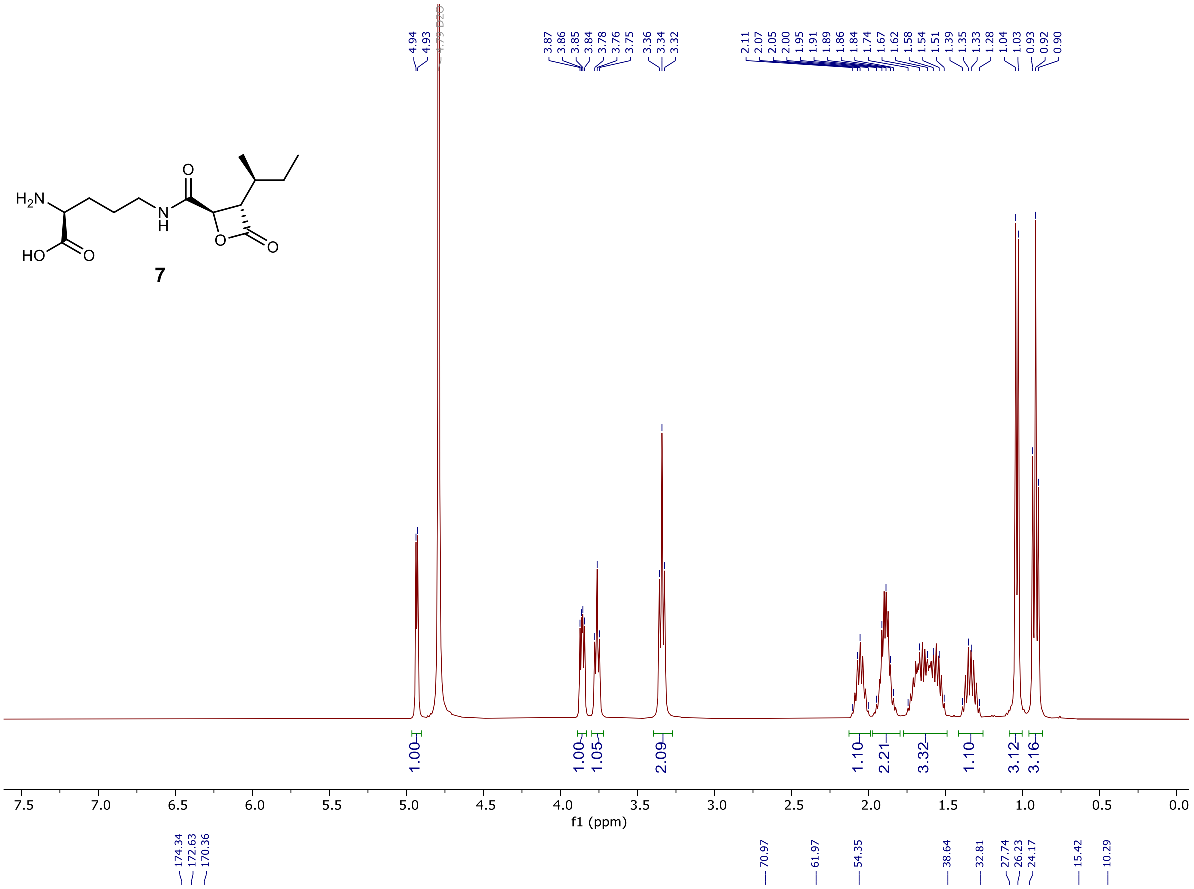

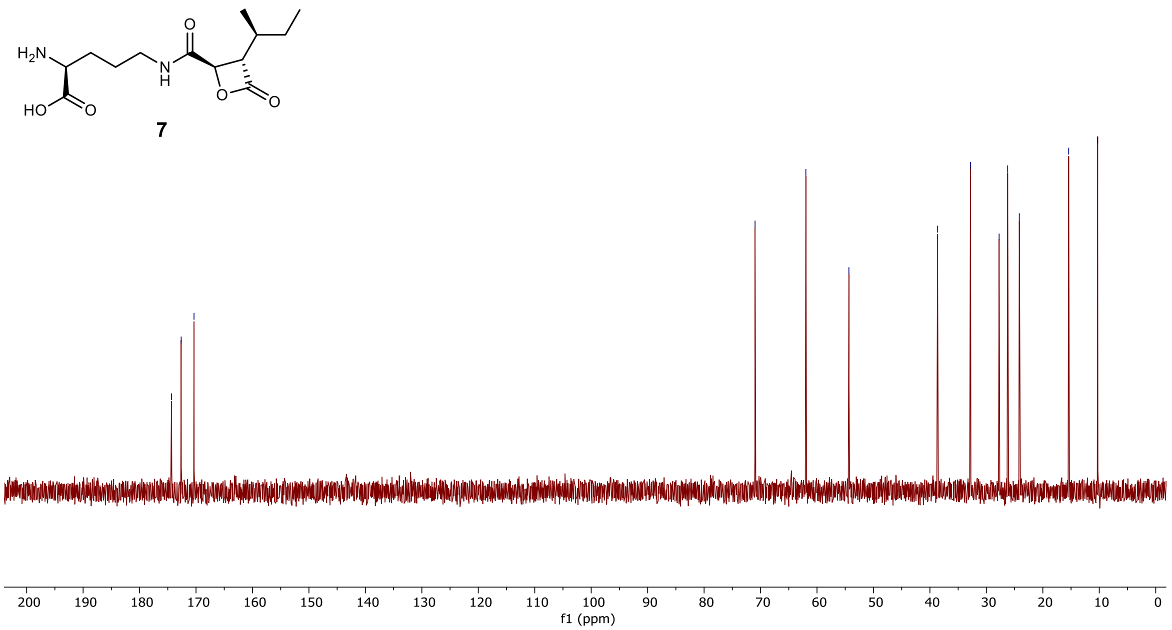

Supplementary Fig. 46. NMR of compound 7.

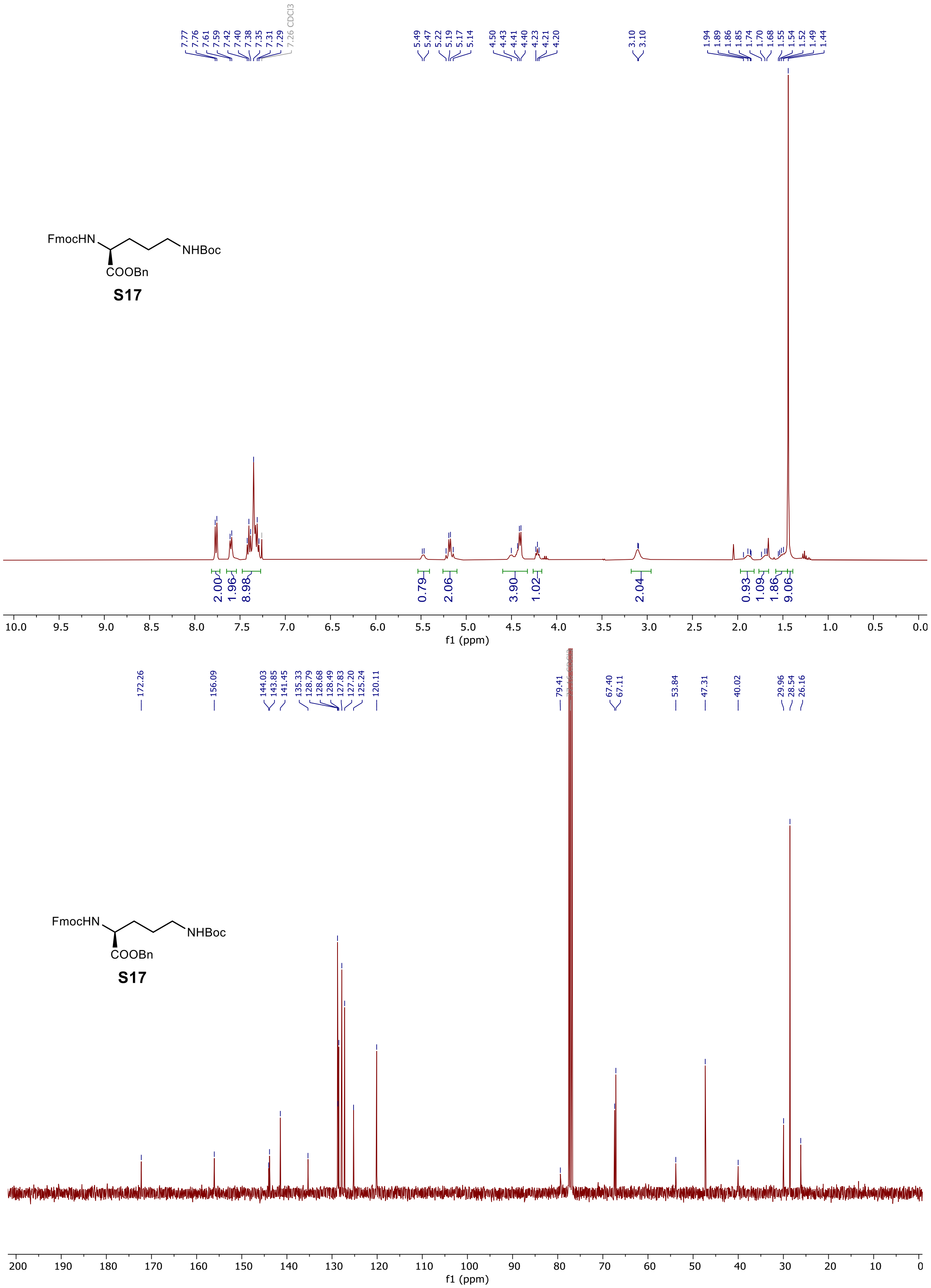

Supplementary Fig. 47. NMR of compound S17.

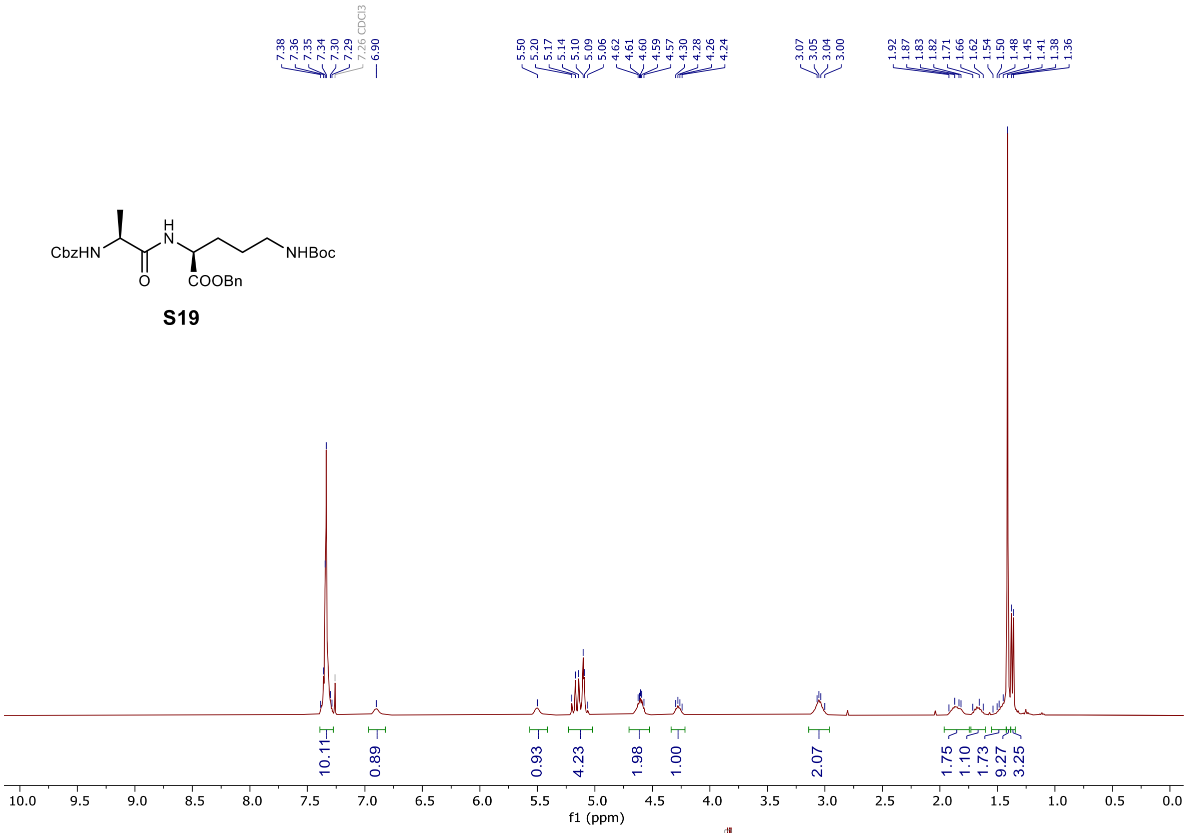

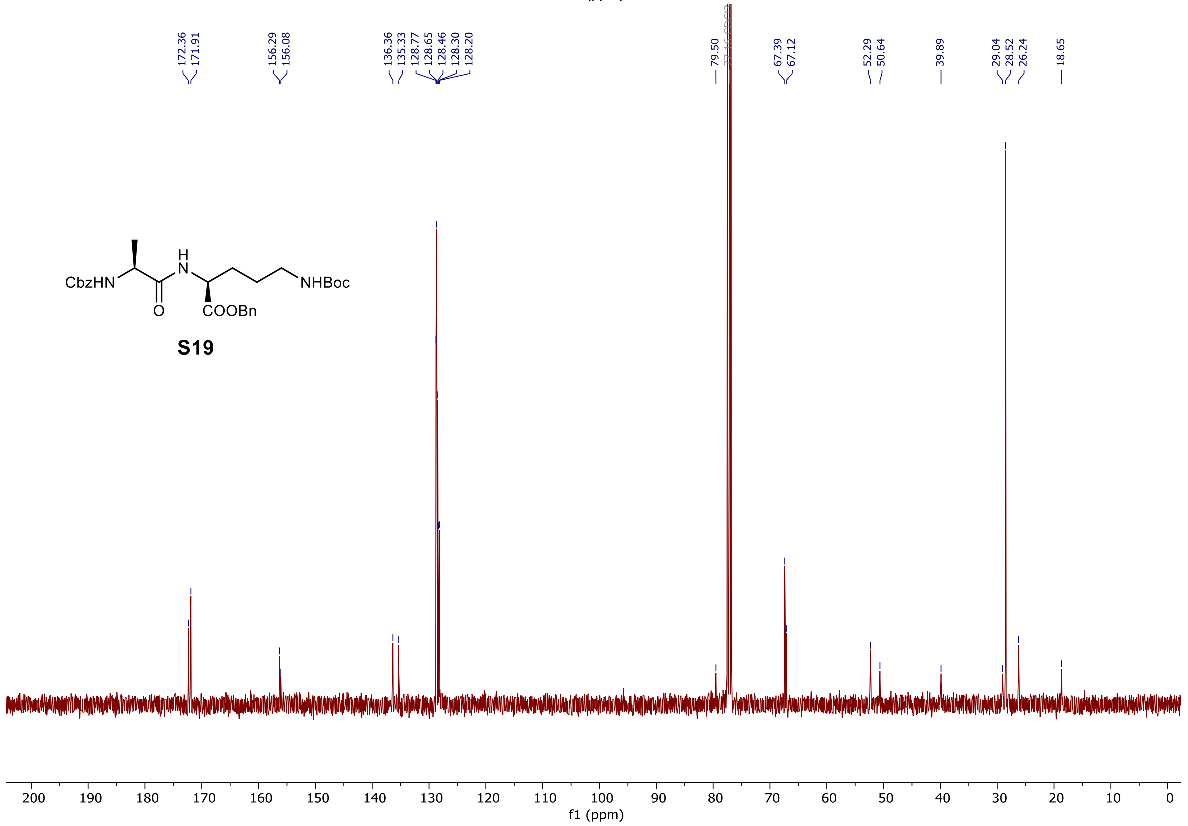

Supplementary Fig. 48. NMR of compound S19.

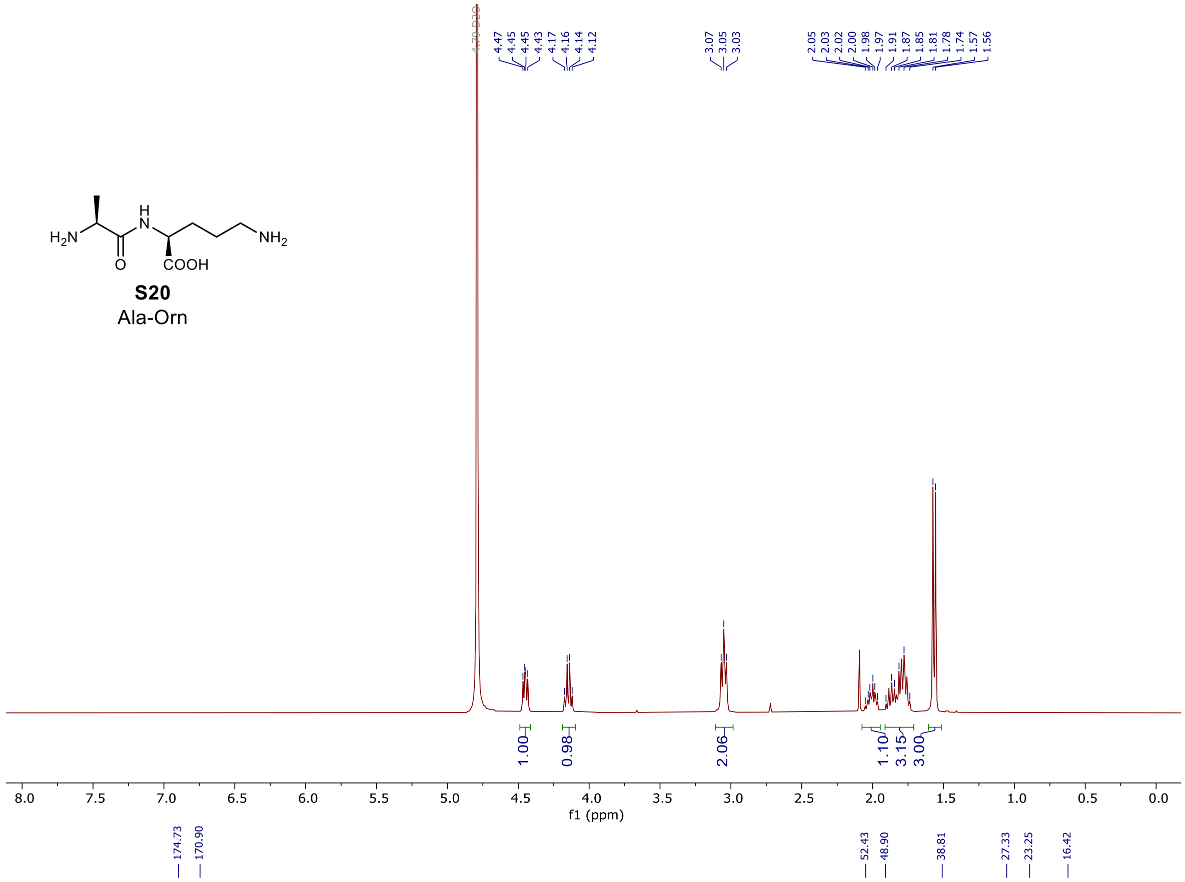

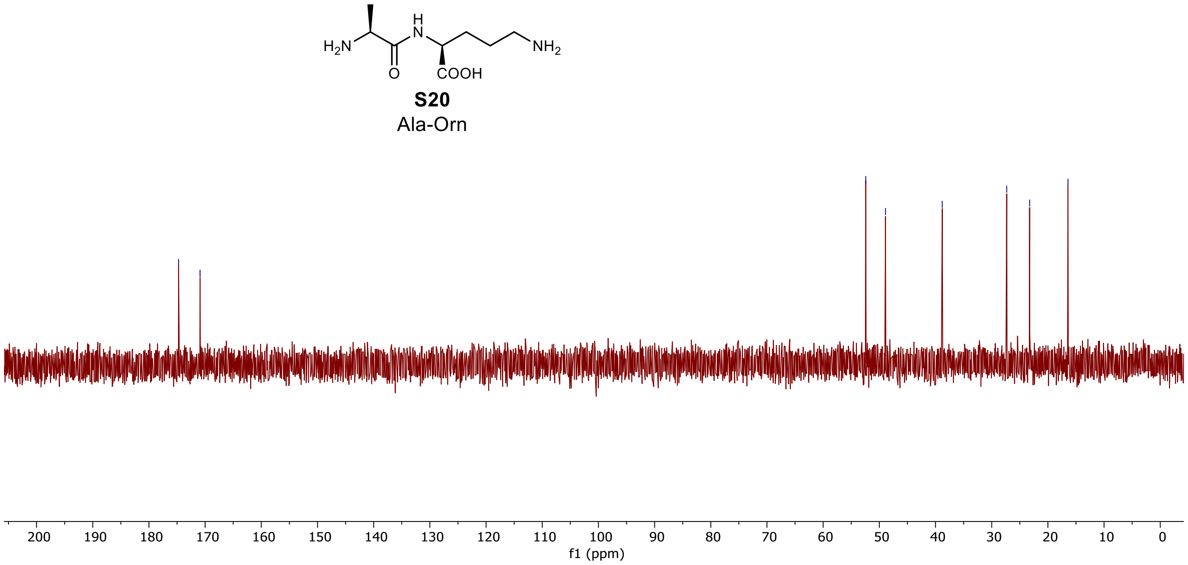

Supplementary Fig. 49. NMR of compound S20.

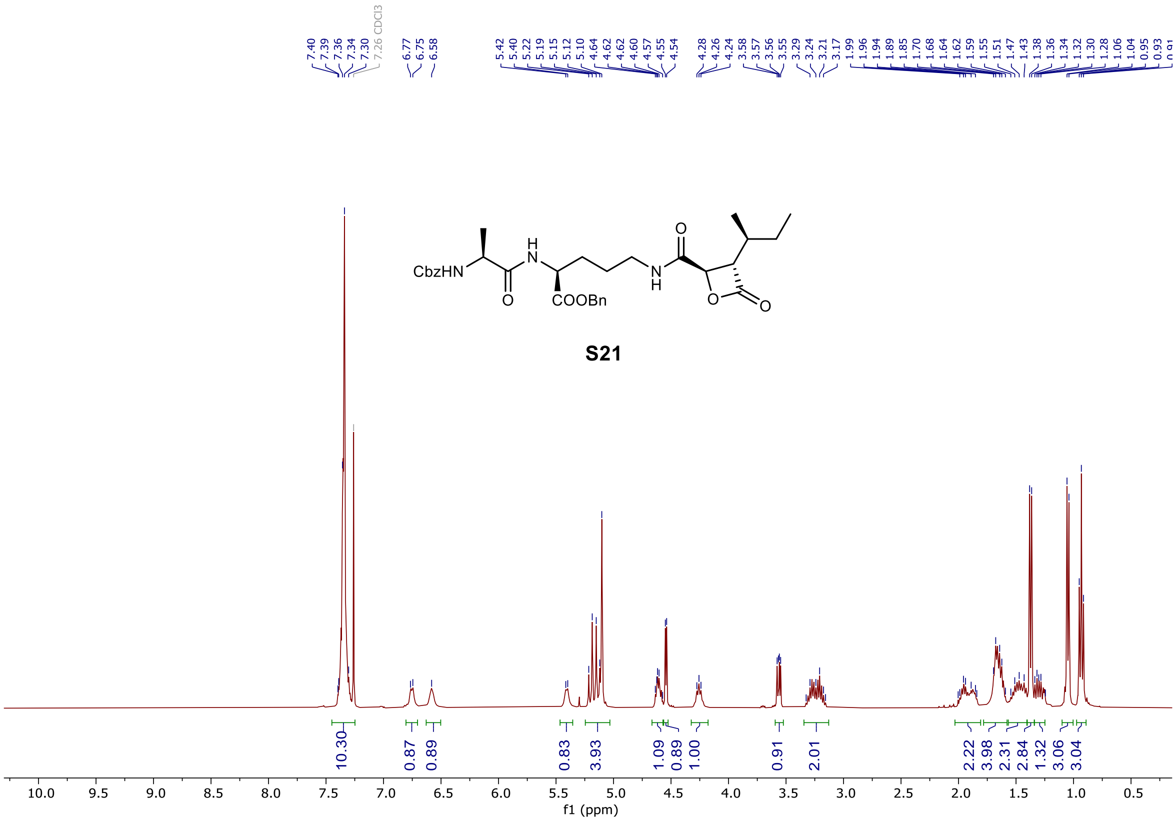

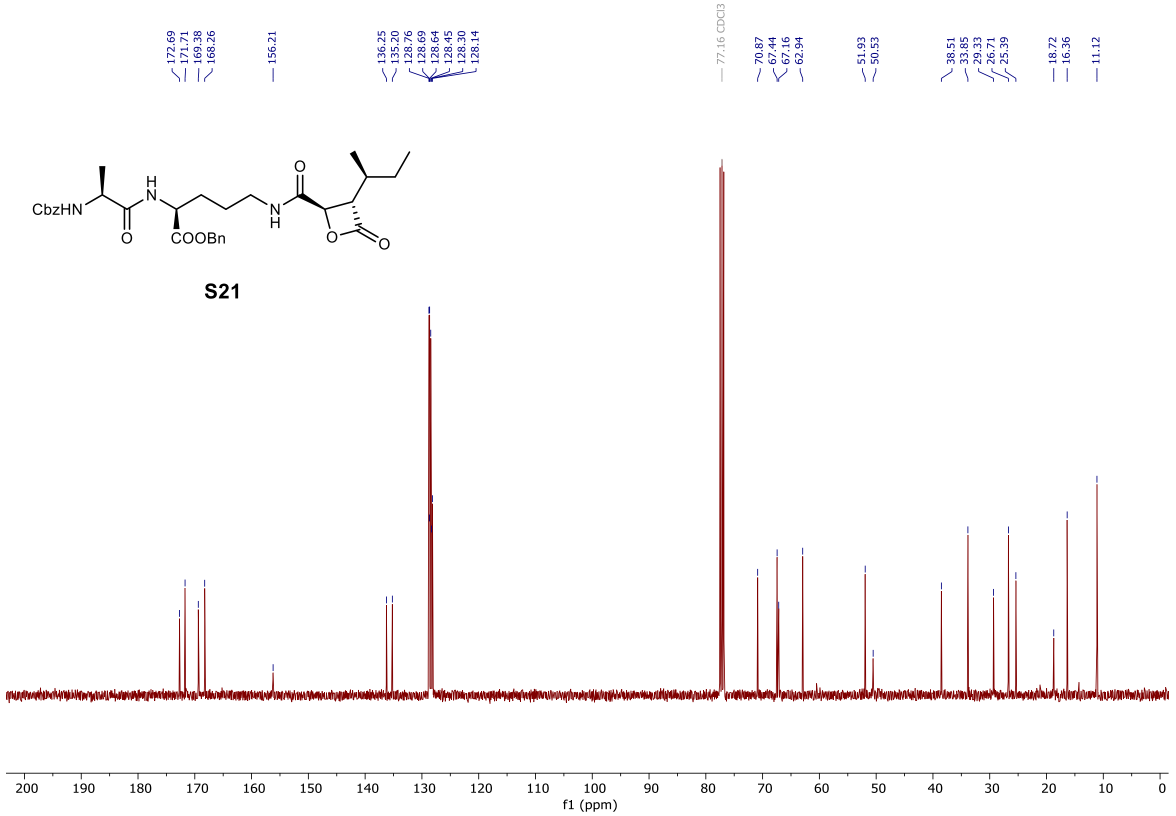

Supplementary Fig. 50. NMR of compound S21.

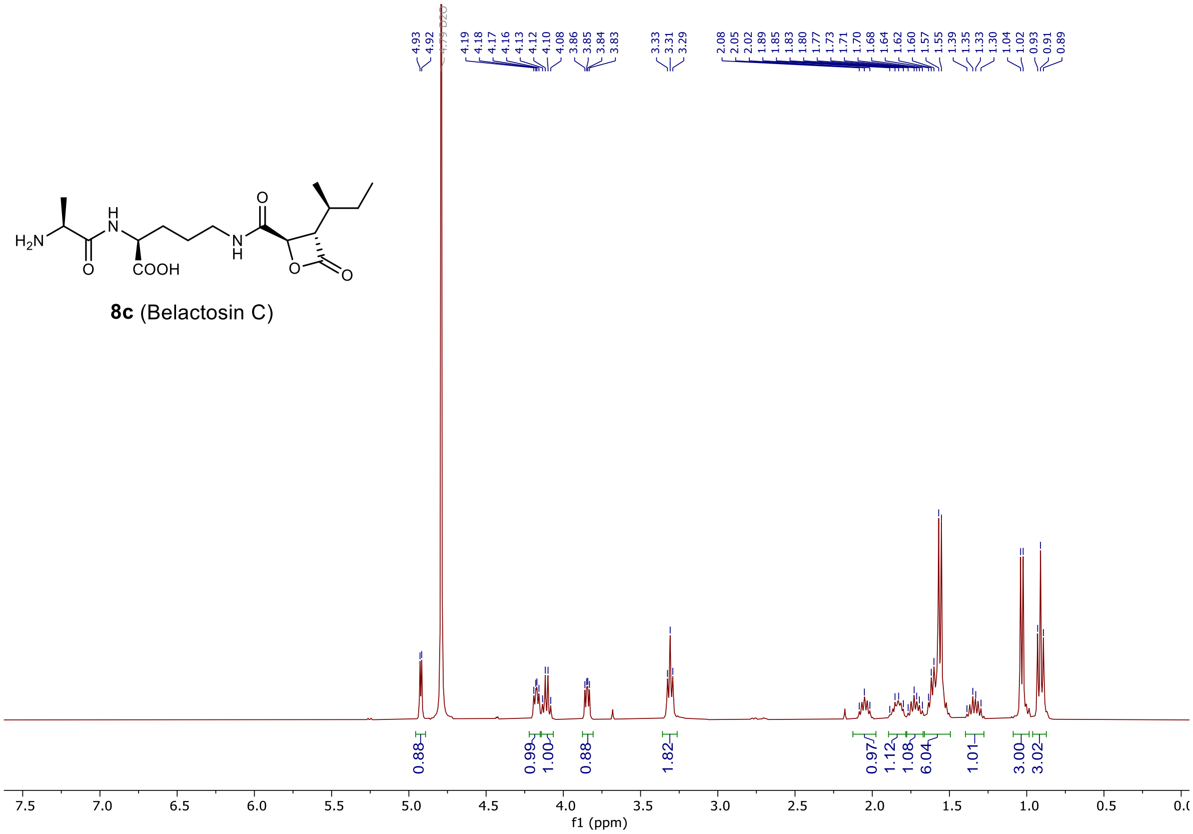

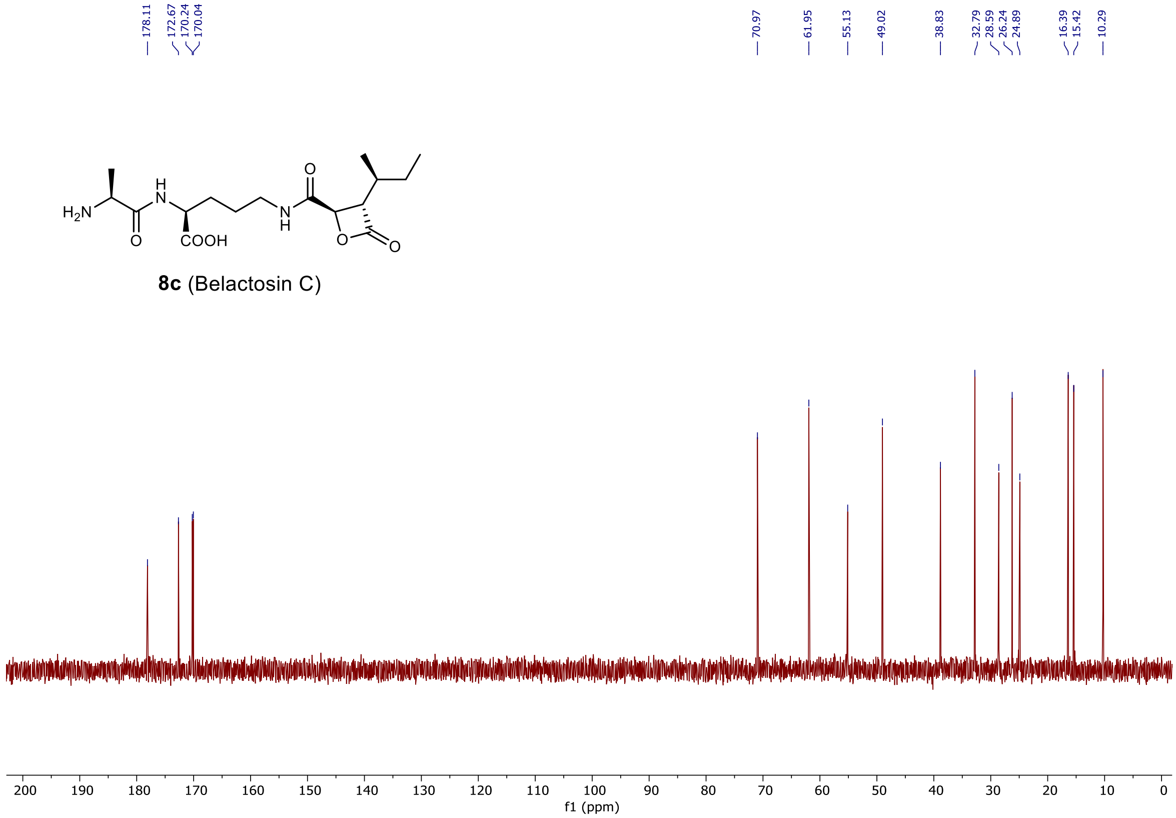

Supplementary Fig. 51. NMR of compound 8c.

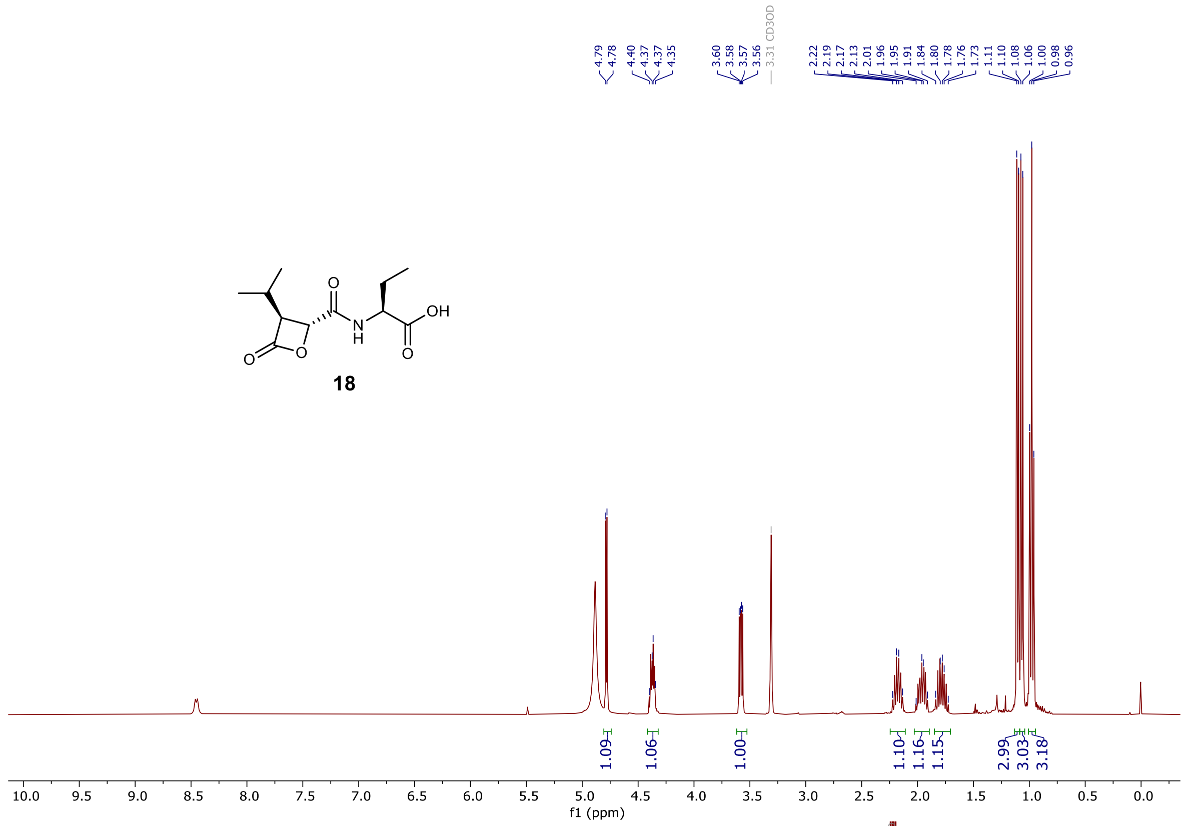

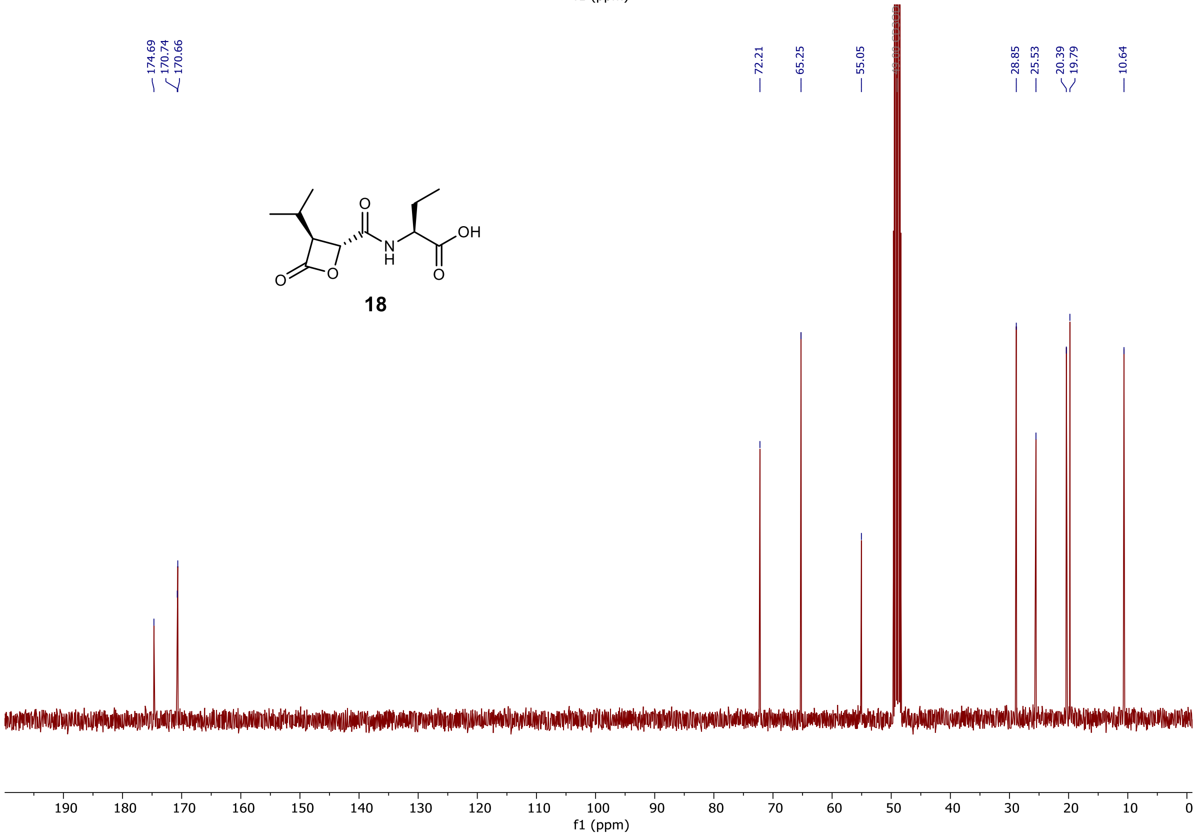

Supplementary Fig. 52. NMR of compound 18.

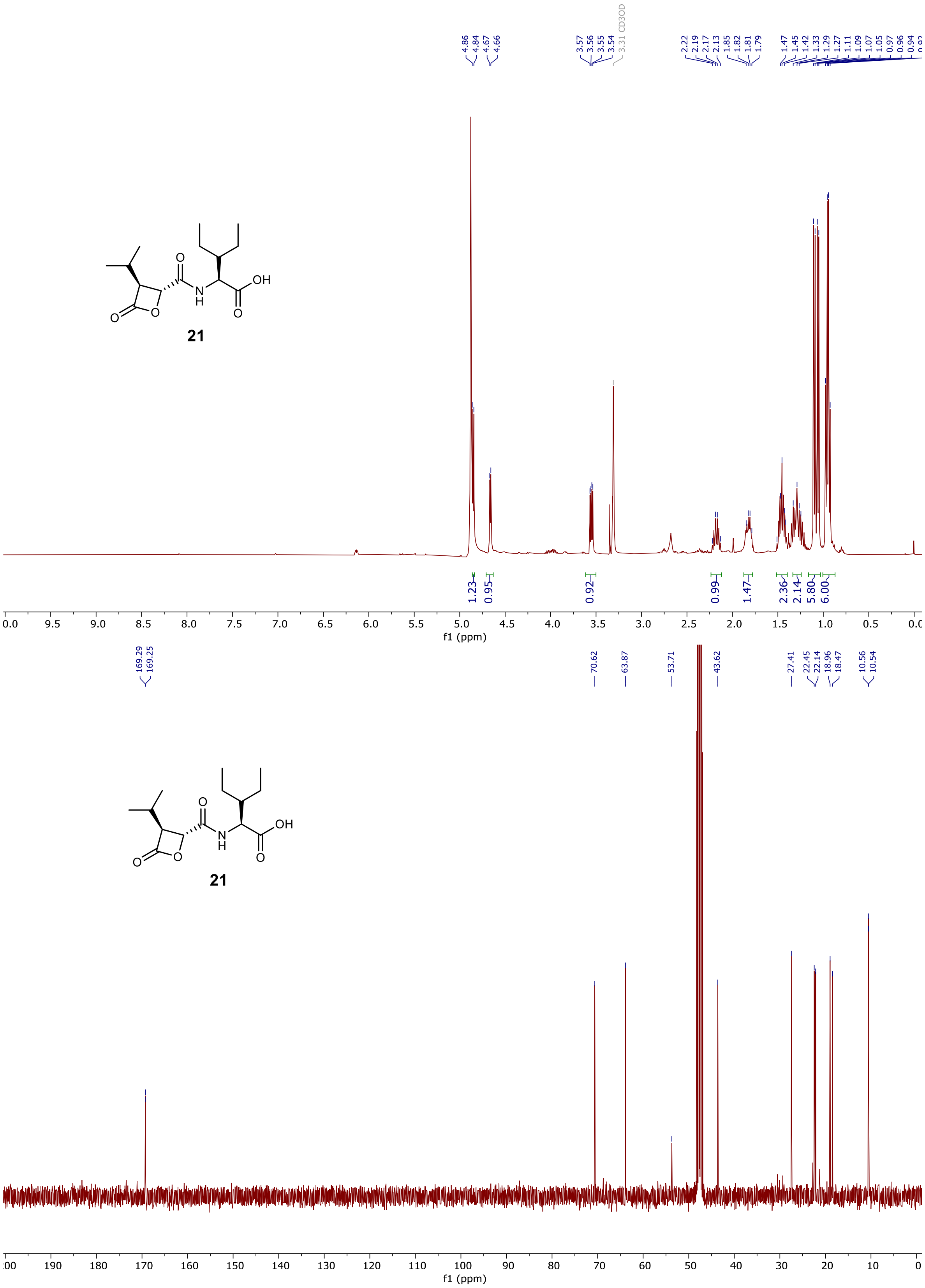

Supplementary Fig. 53. NMR of compound 21.

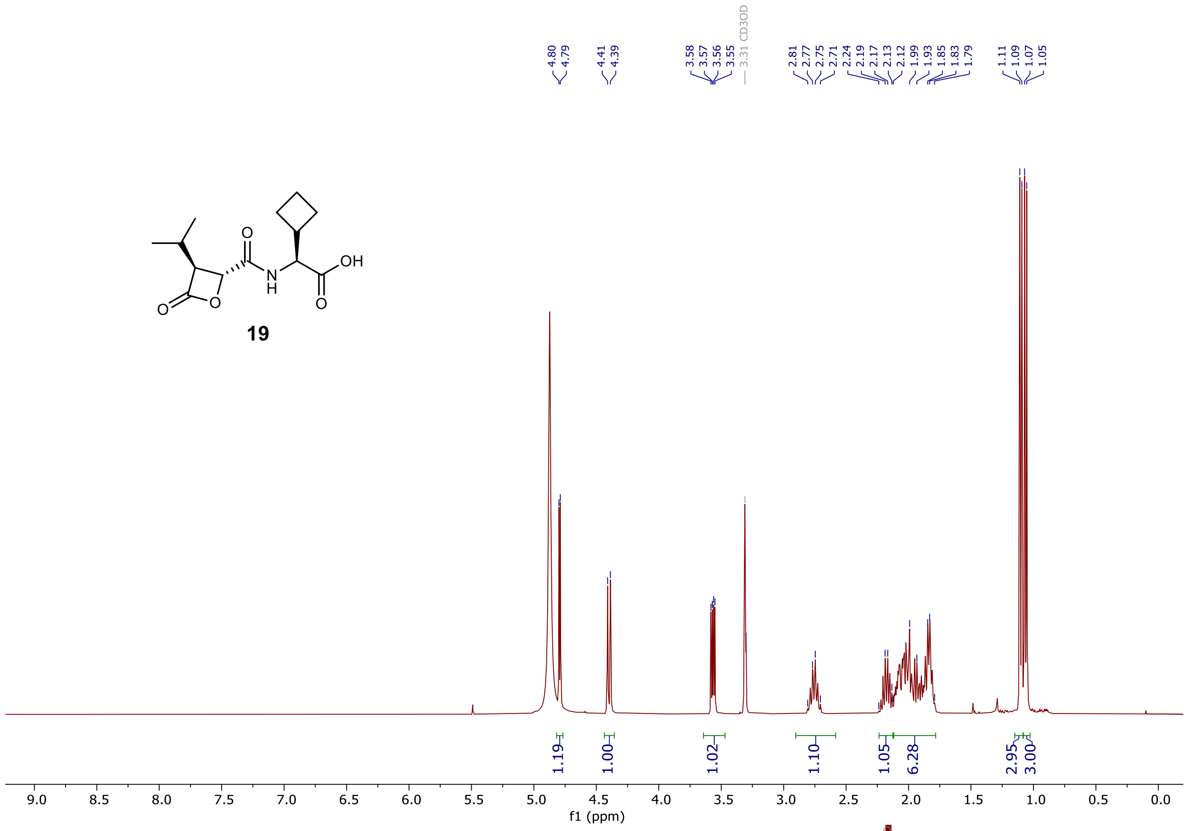

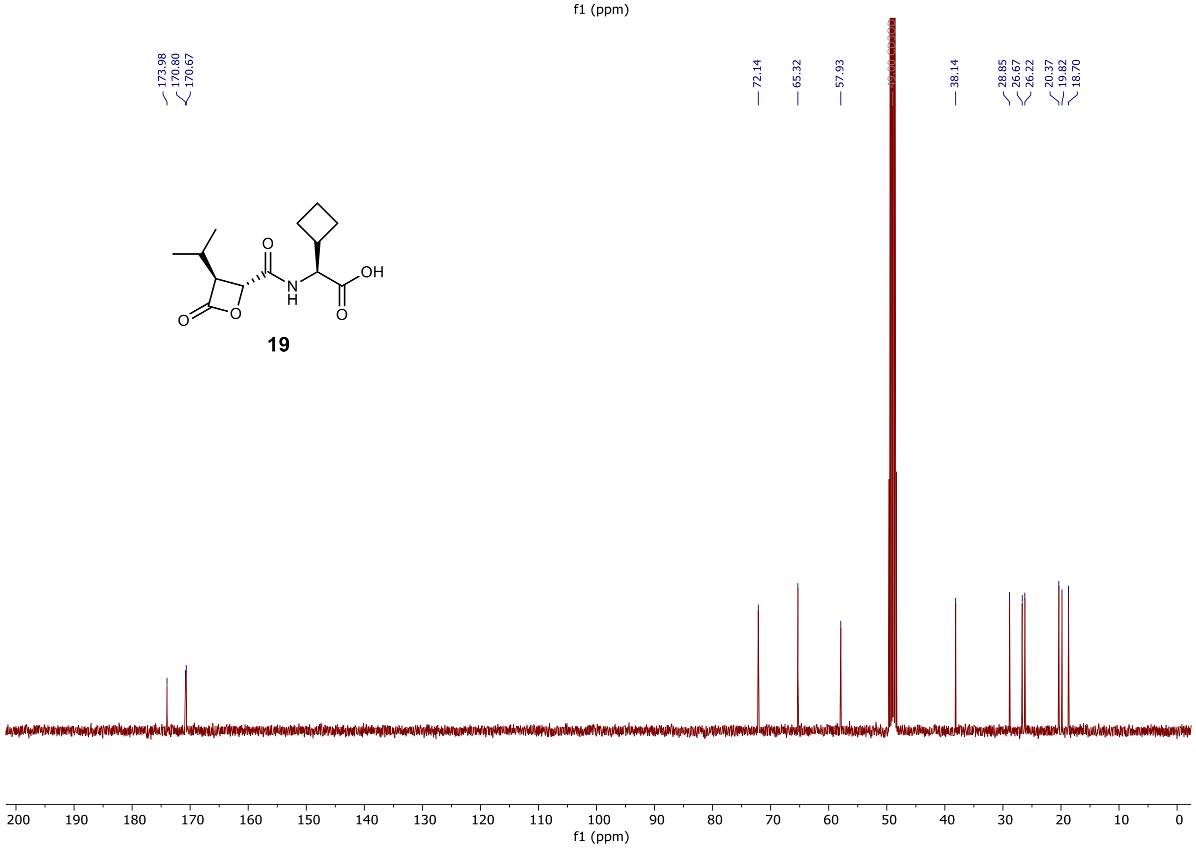

Supplementary Fig. 54. NMR of compound 19.

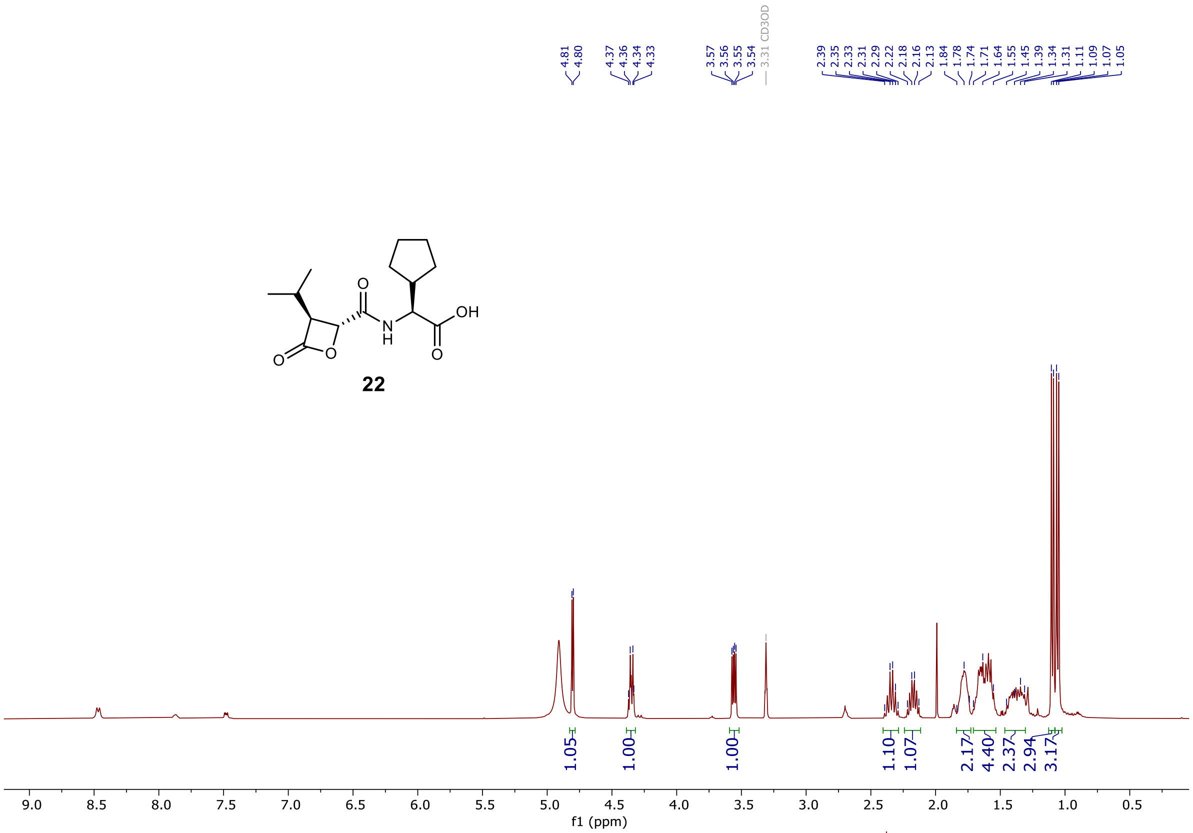

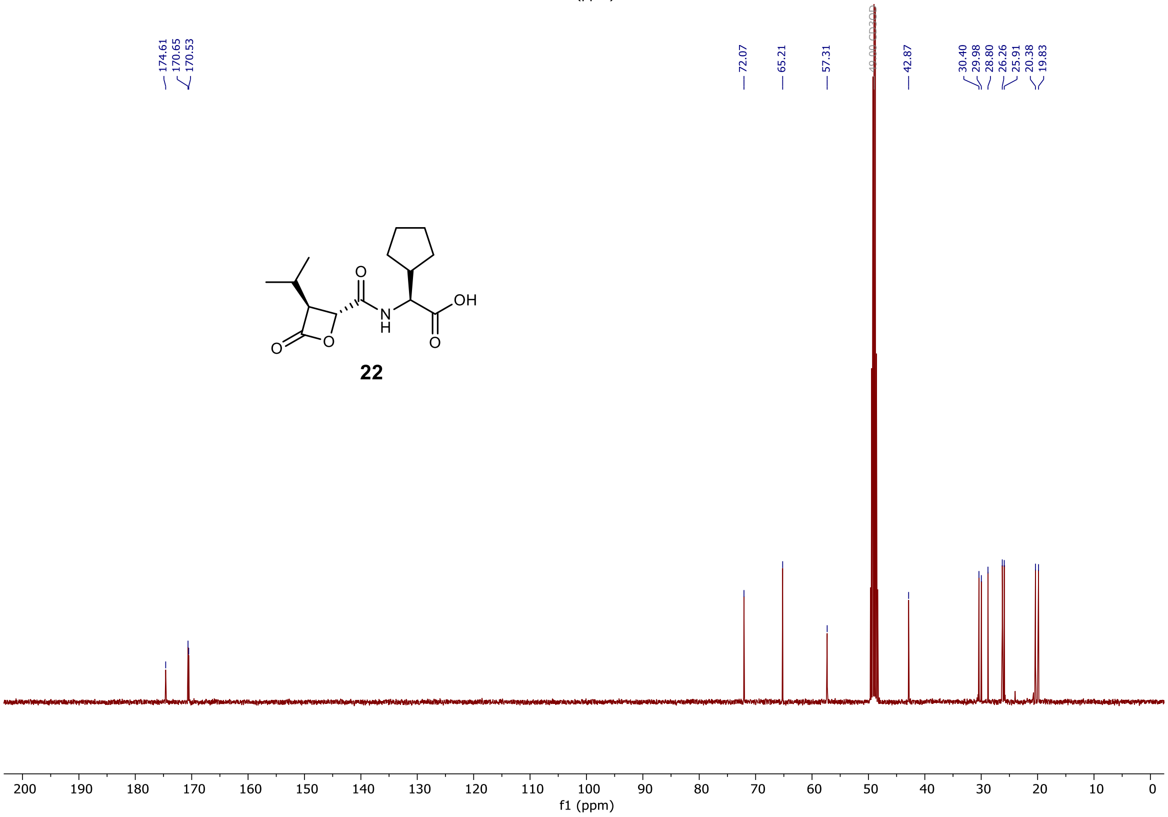

Supplementary Fig. 55. NMR of compound 22.

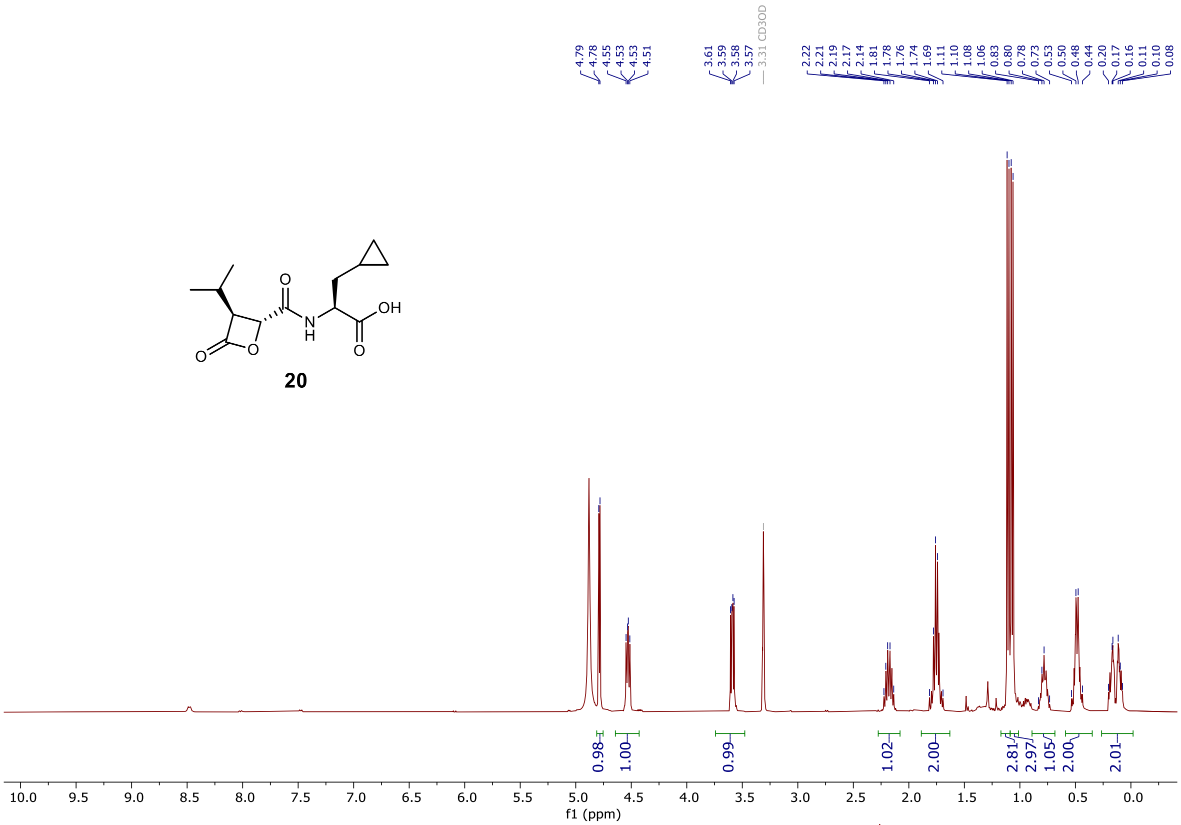

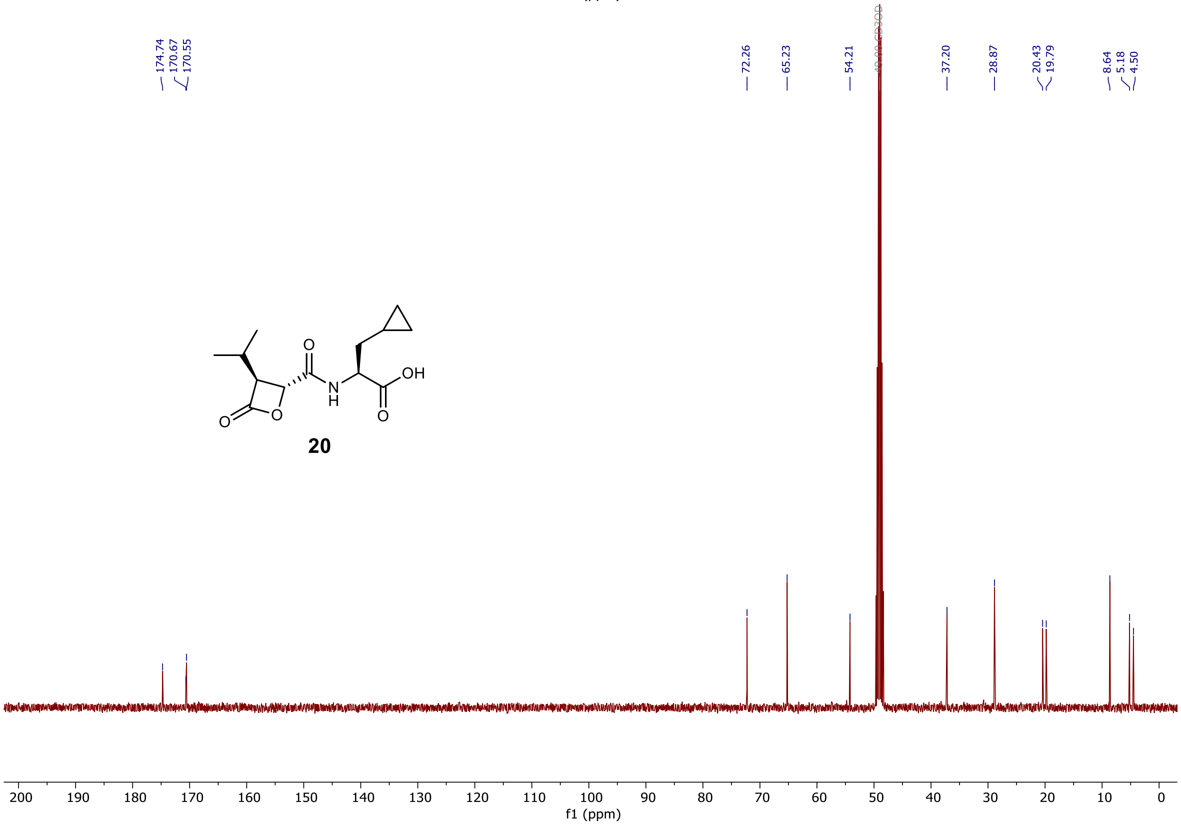

Supplementary Fig. 56. NMR of compound 20.

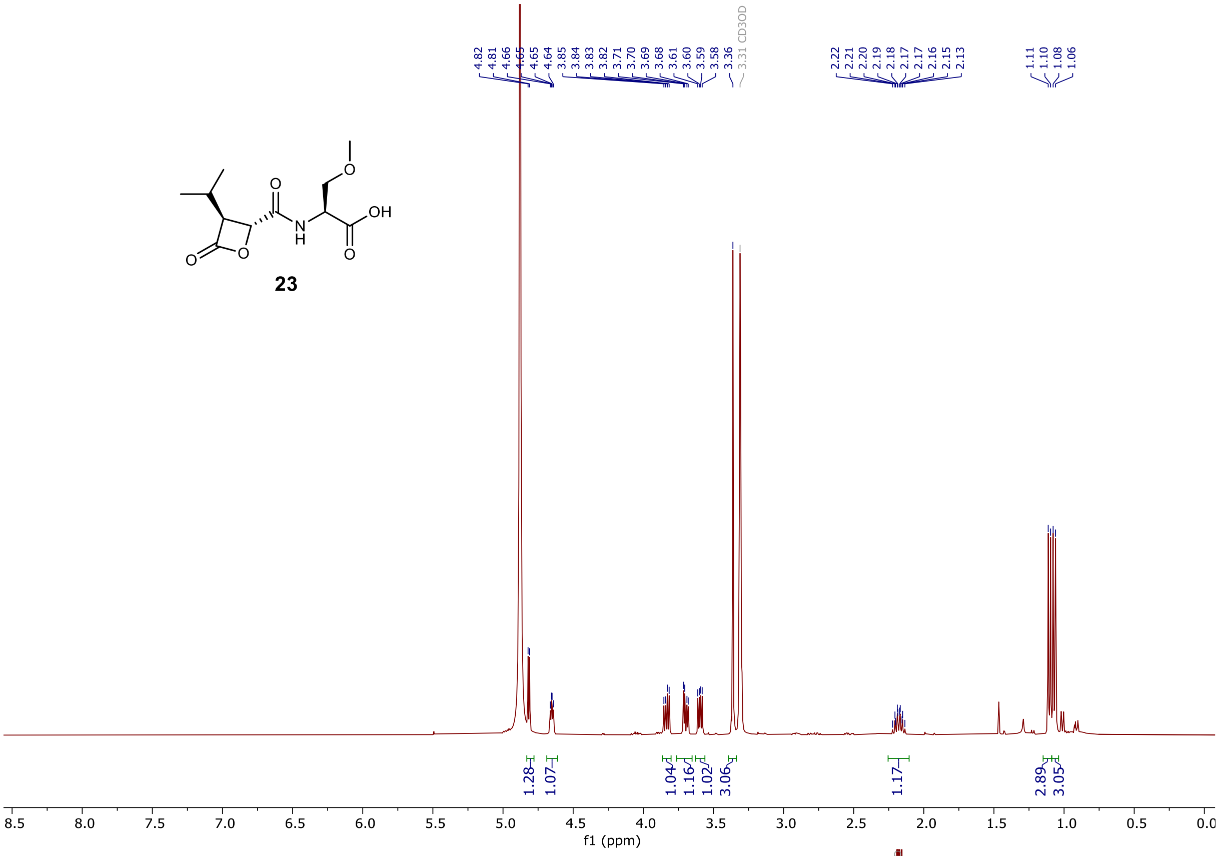

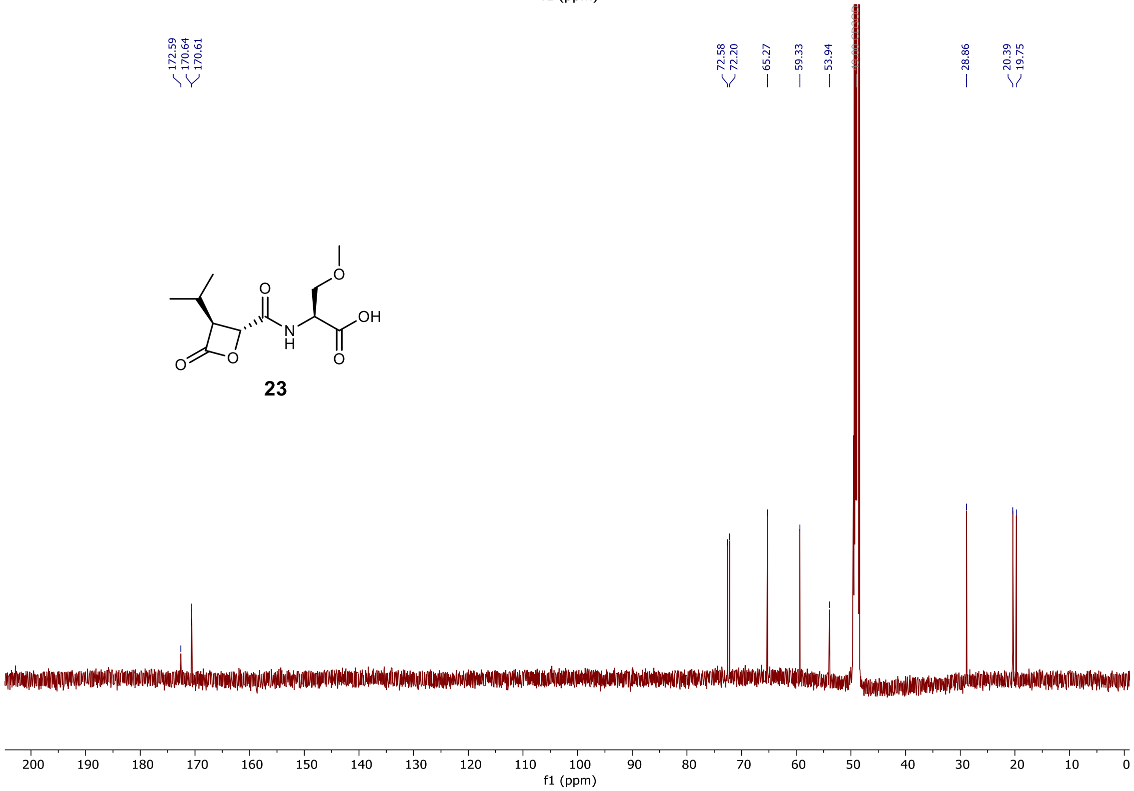

Supplementary Fig. 57. NMR of compound 23.

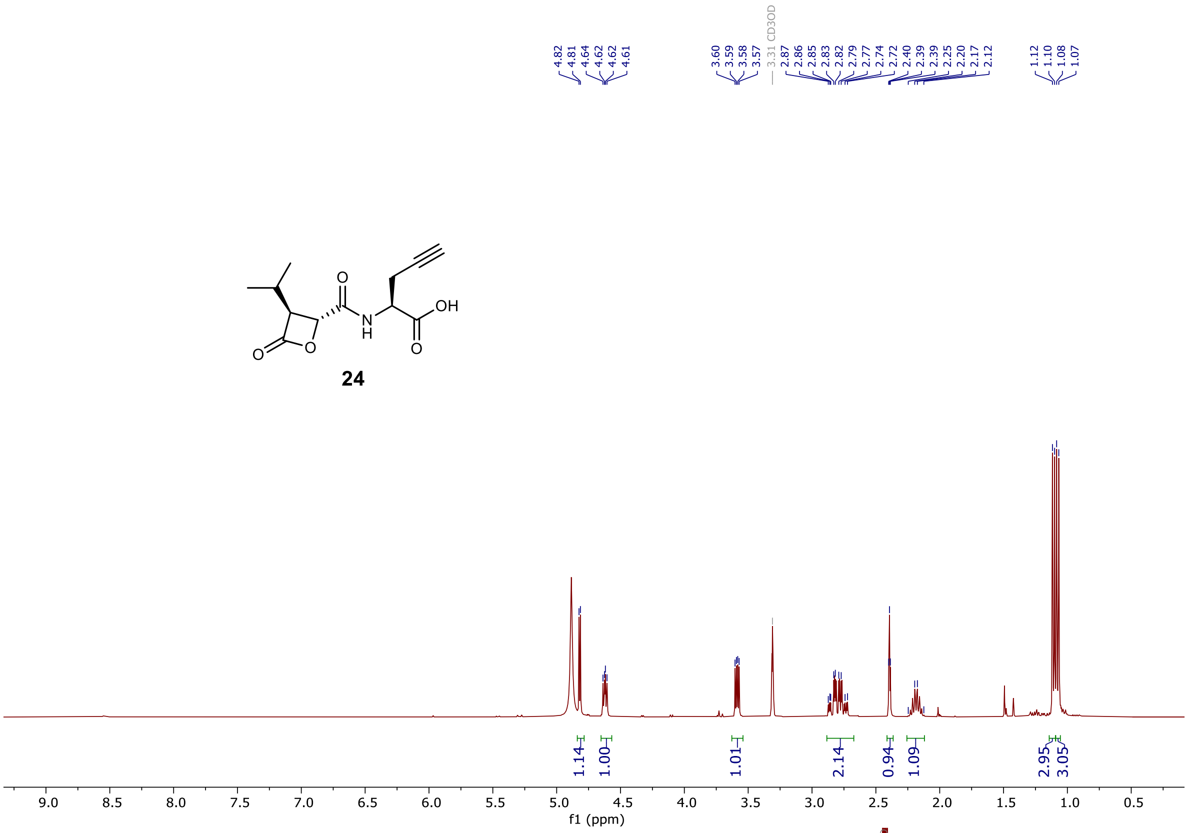

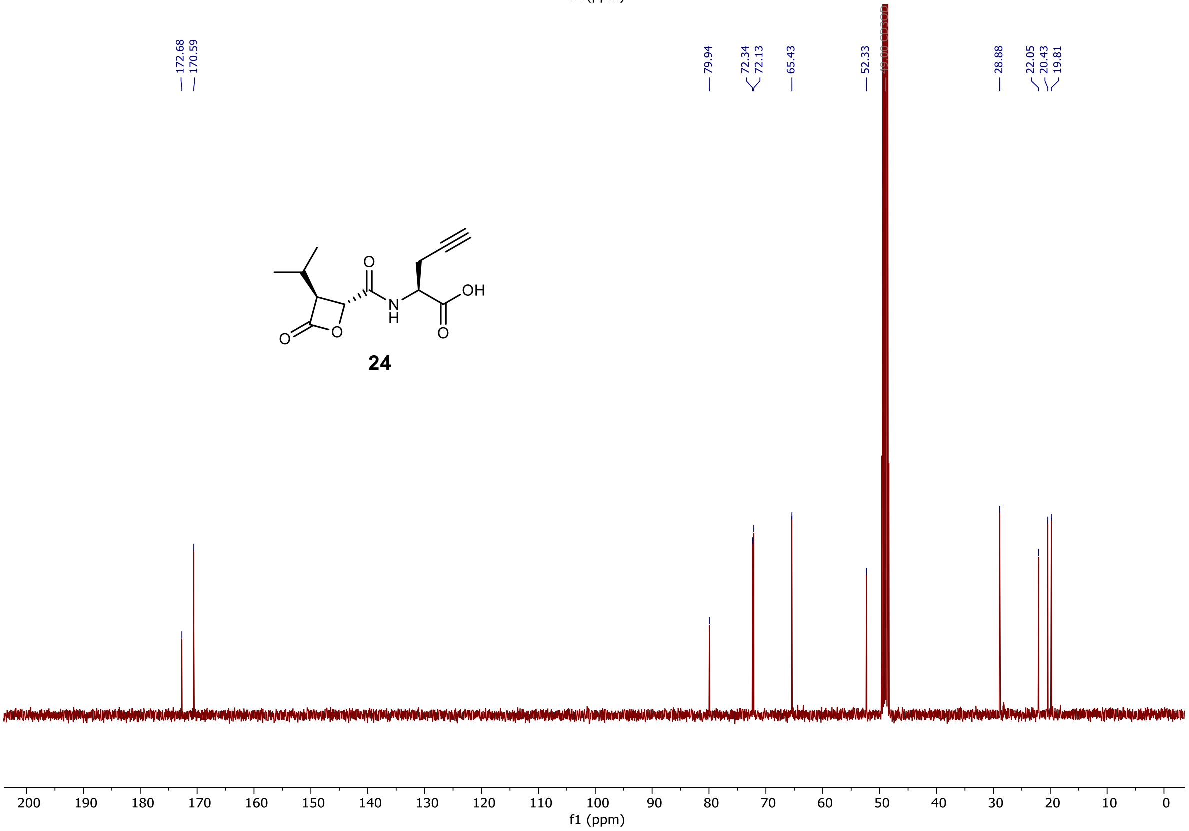

Supplementary Fig. 58. NMR of compound 24.

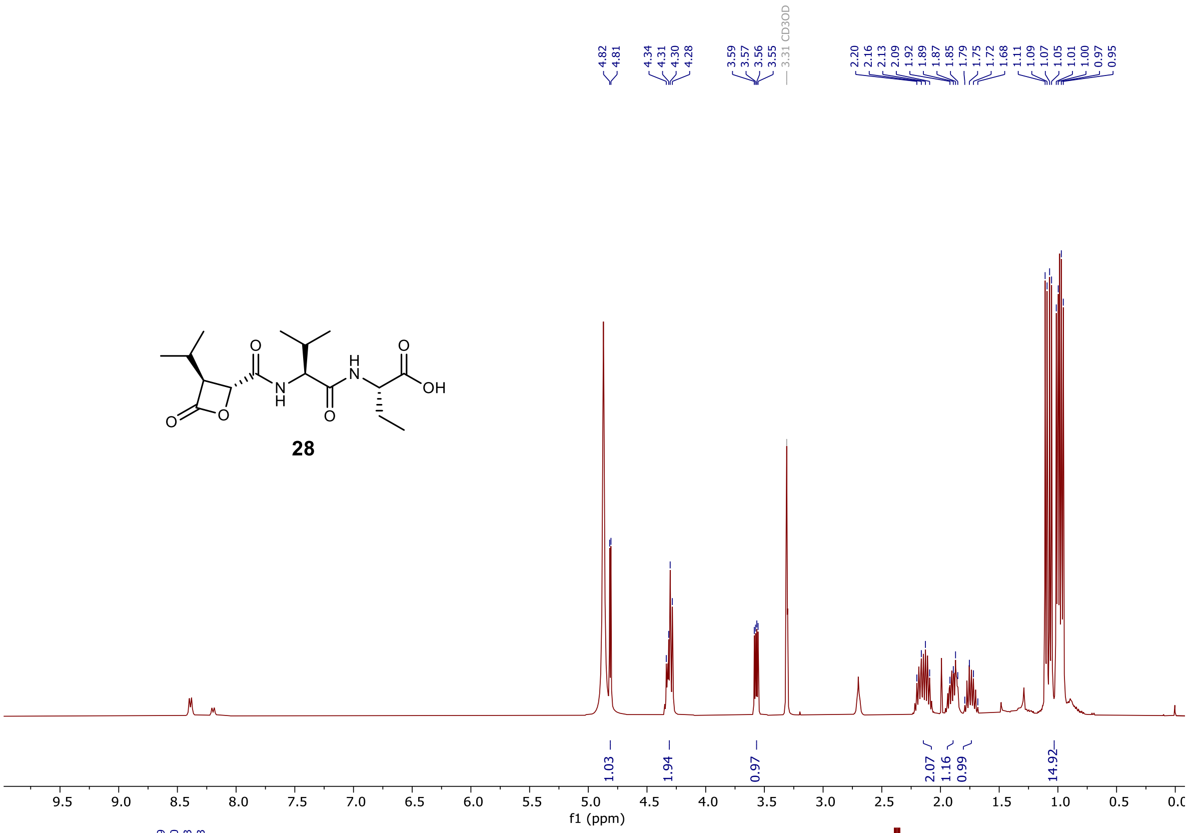

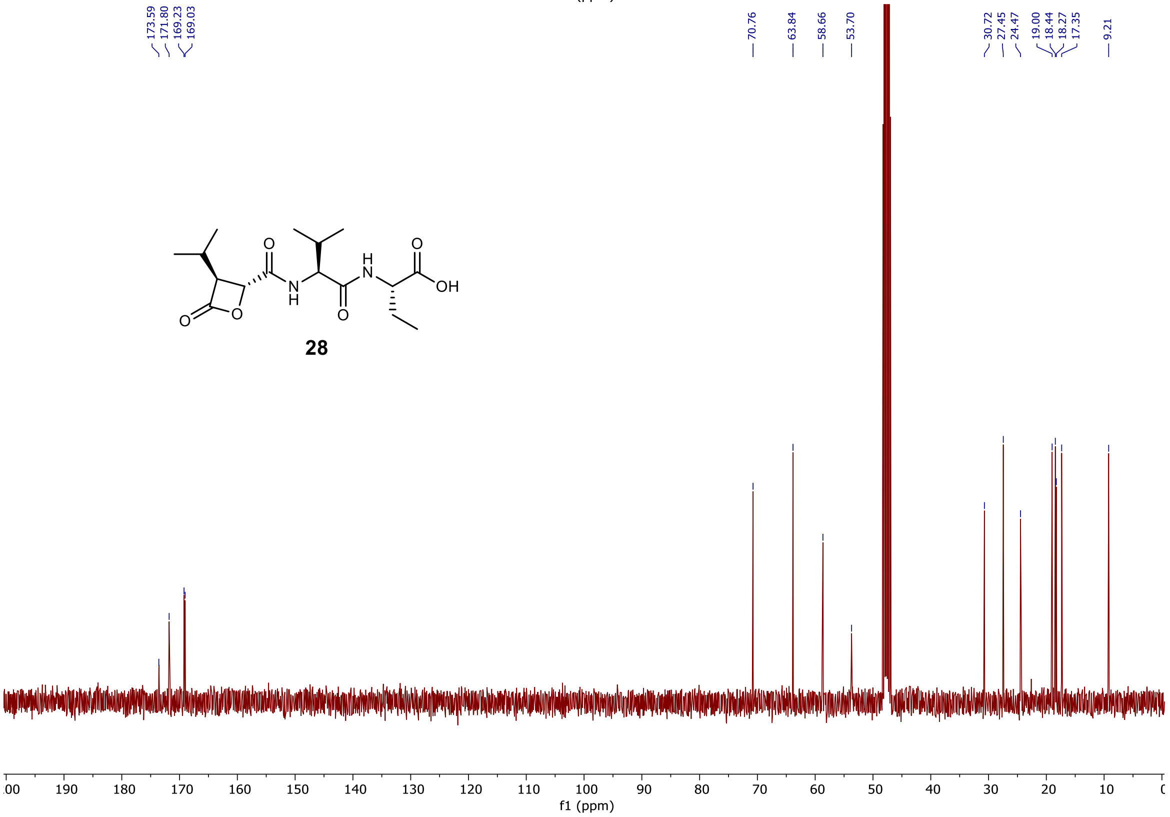

Supplementary Fig. 59. NMR of compound 28.

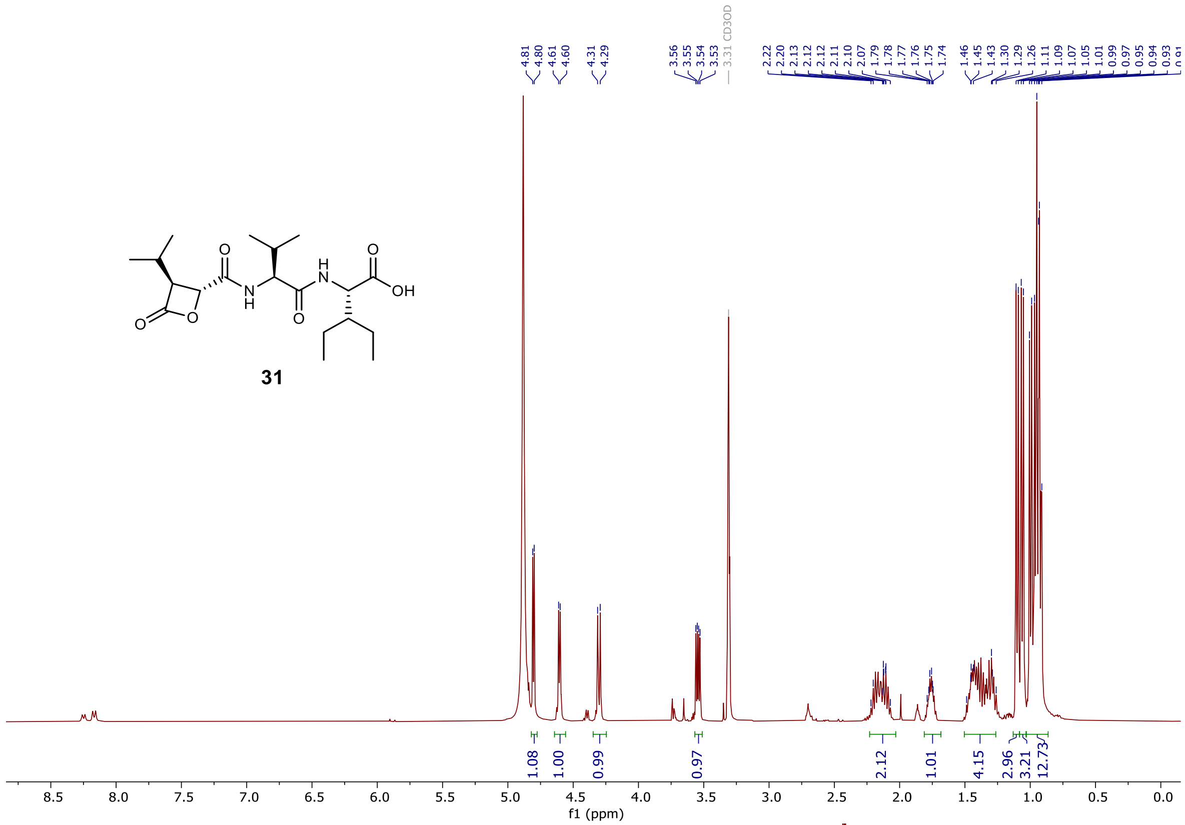

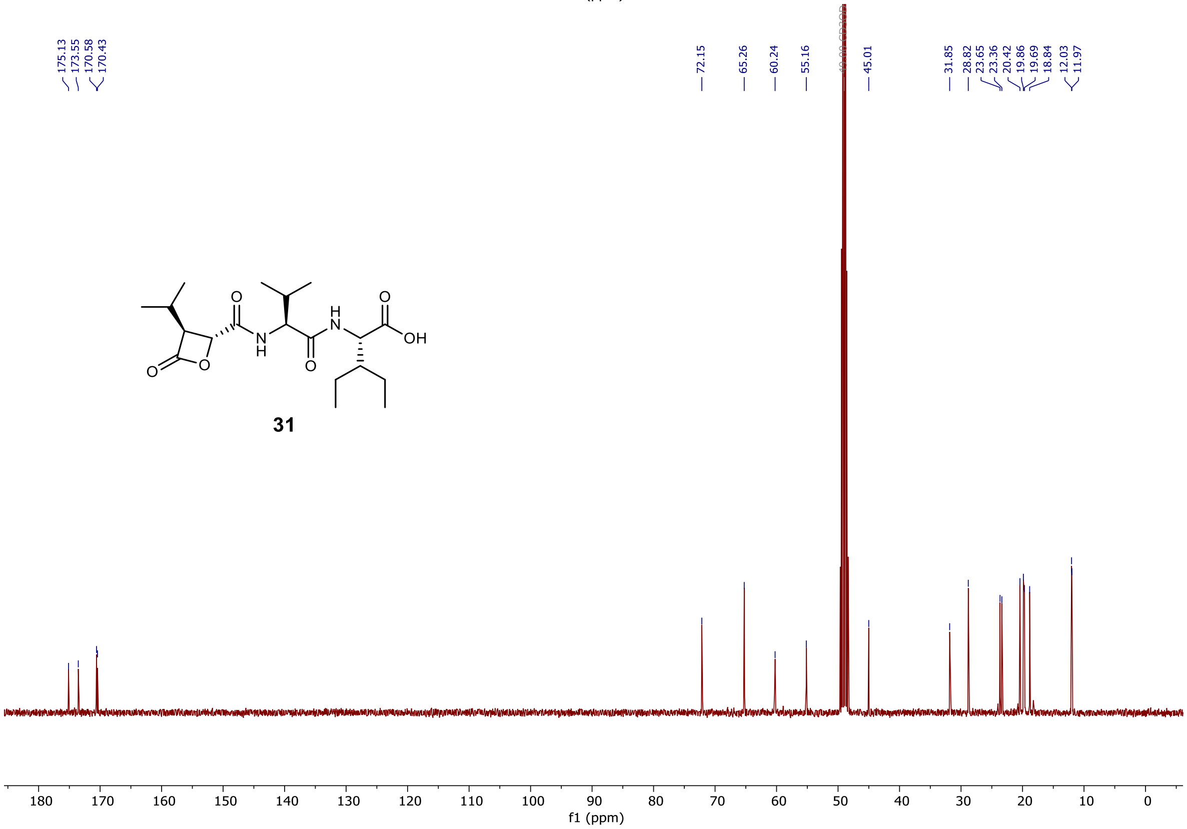

Supplementary Fig. 60. NMR of compound 31.

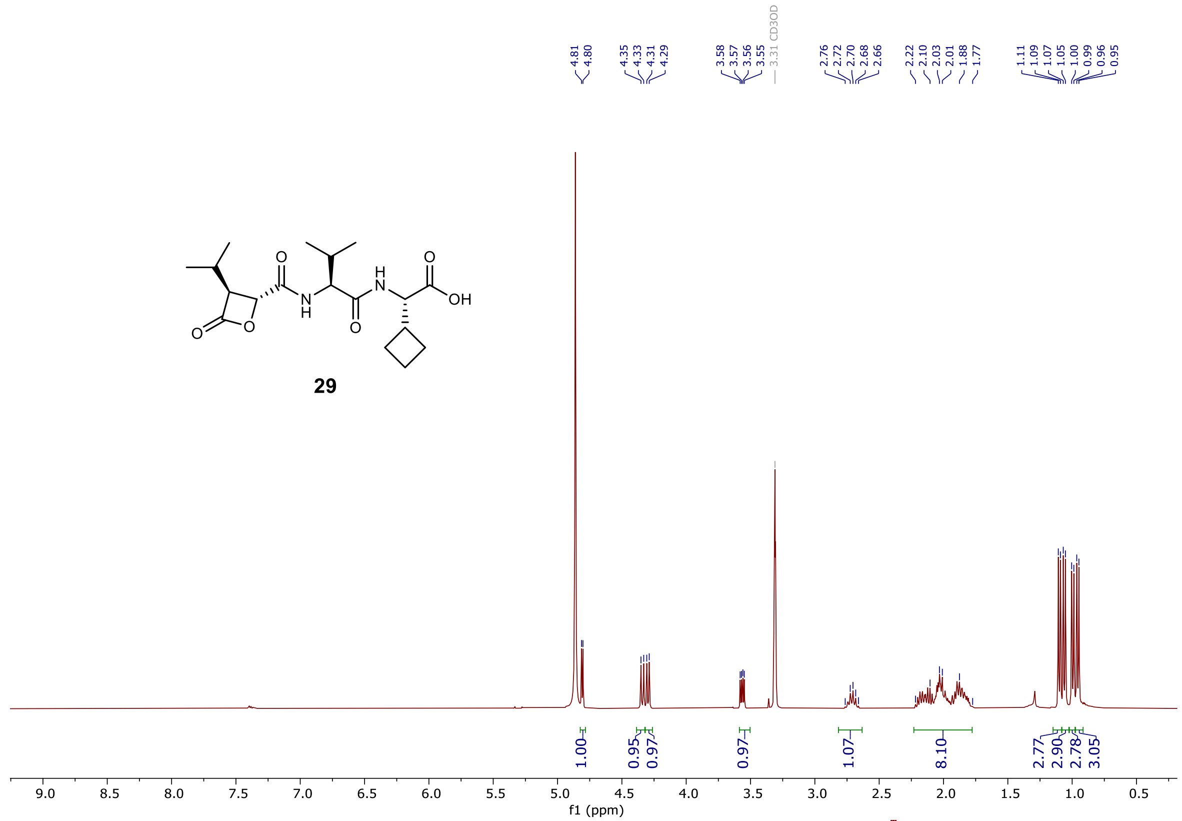

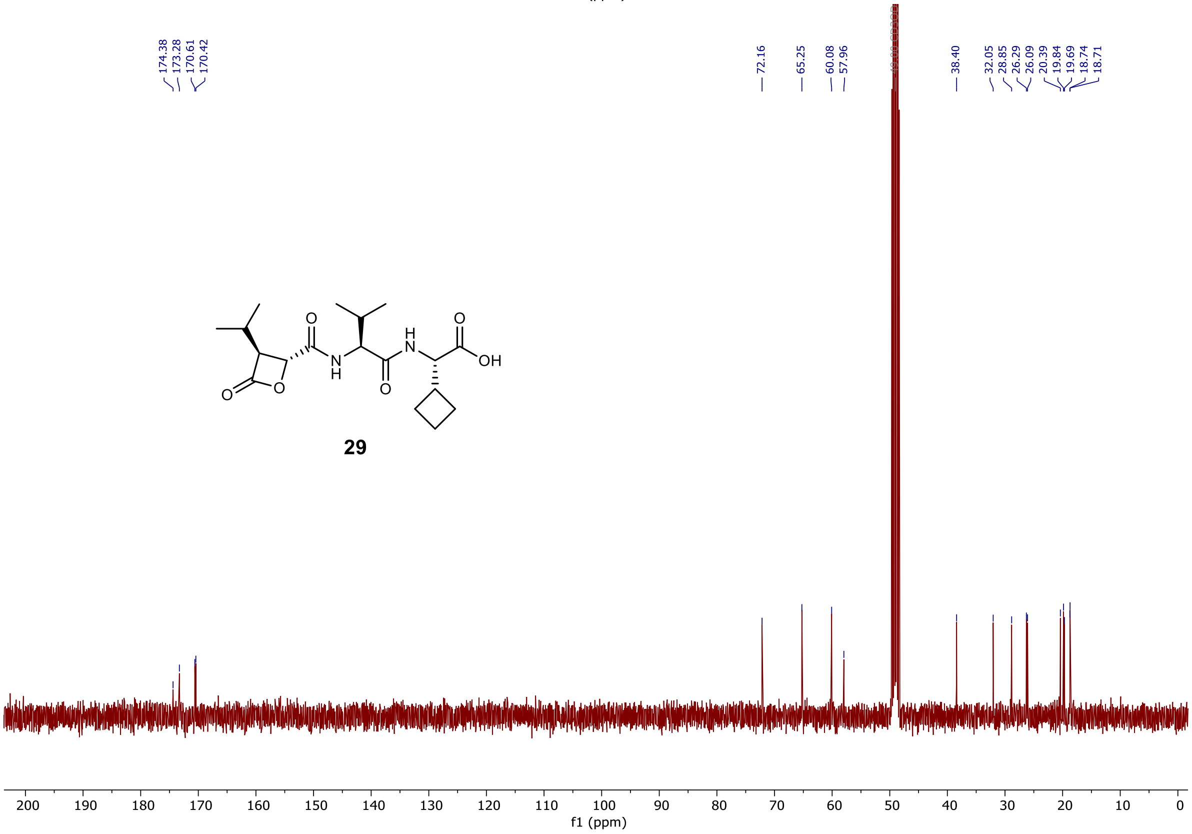

Supplementary Fig. 61. NMR of compound 29.

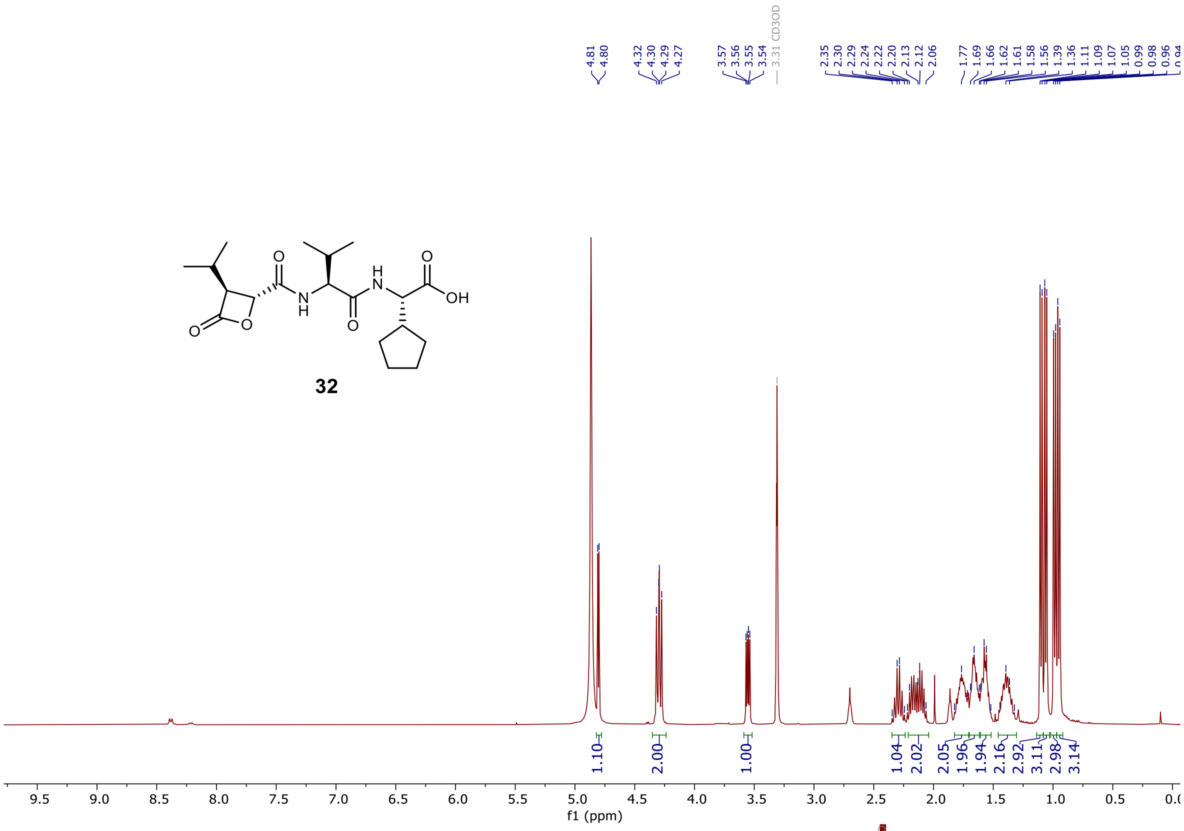

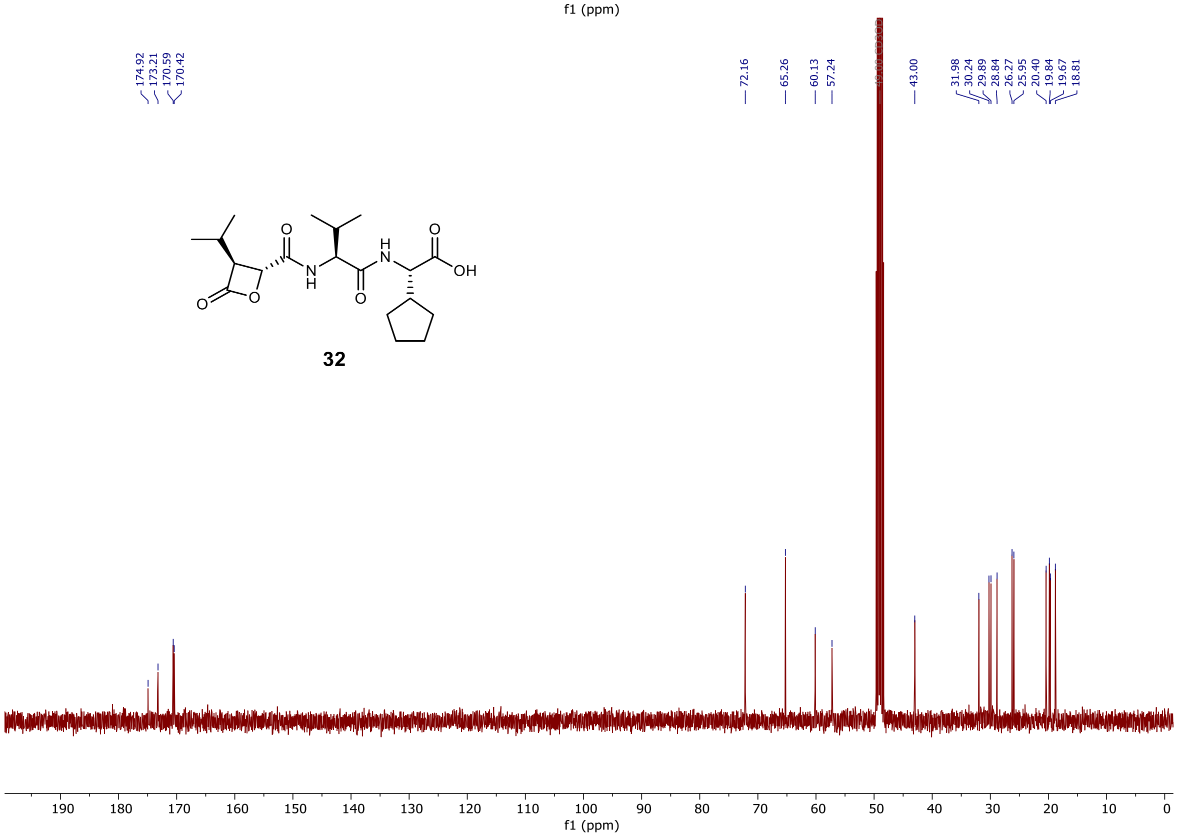

Supplementary Fig. 62. NMR of compound 32.

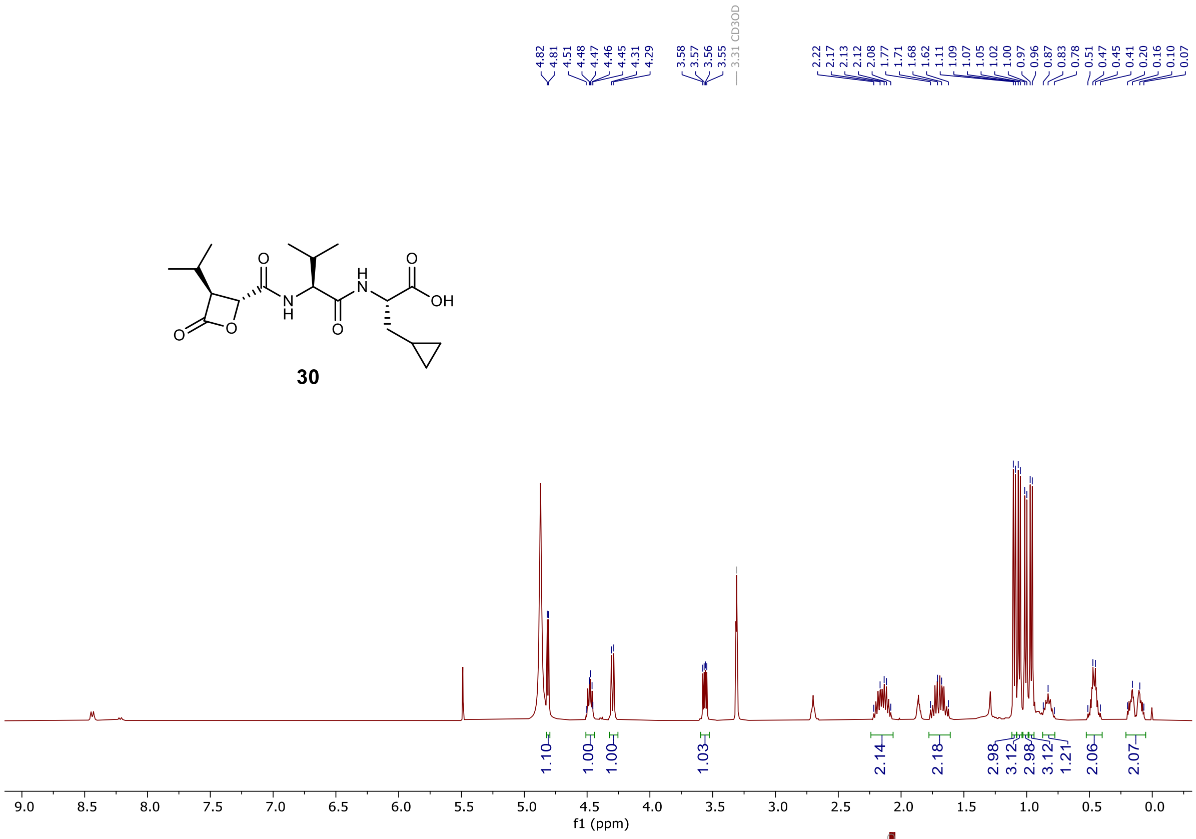

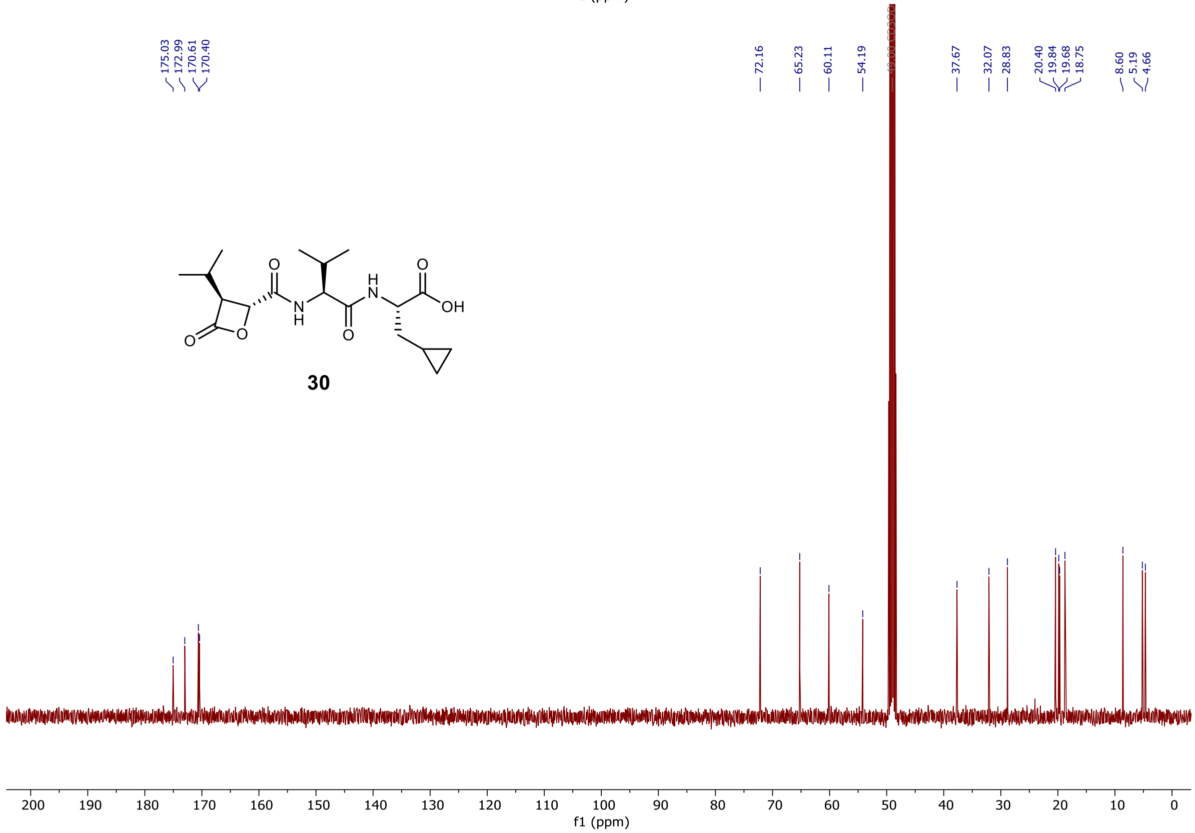

Supplementary Fig. 63. NMR of compound 30.

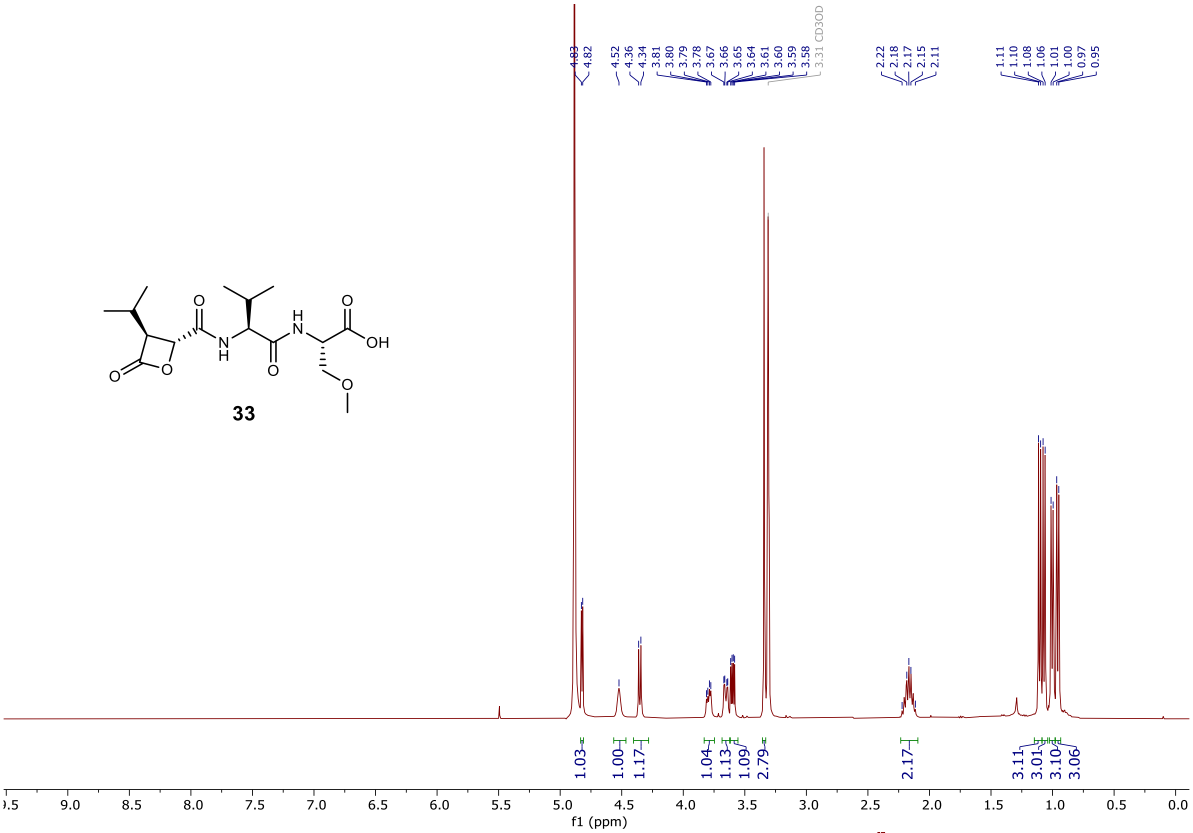

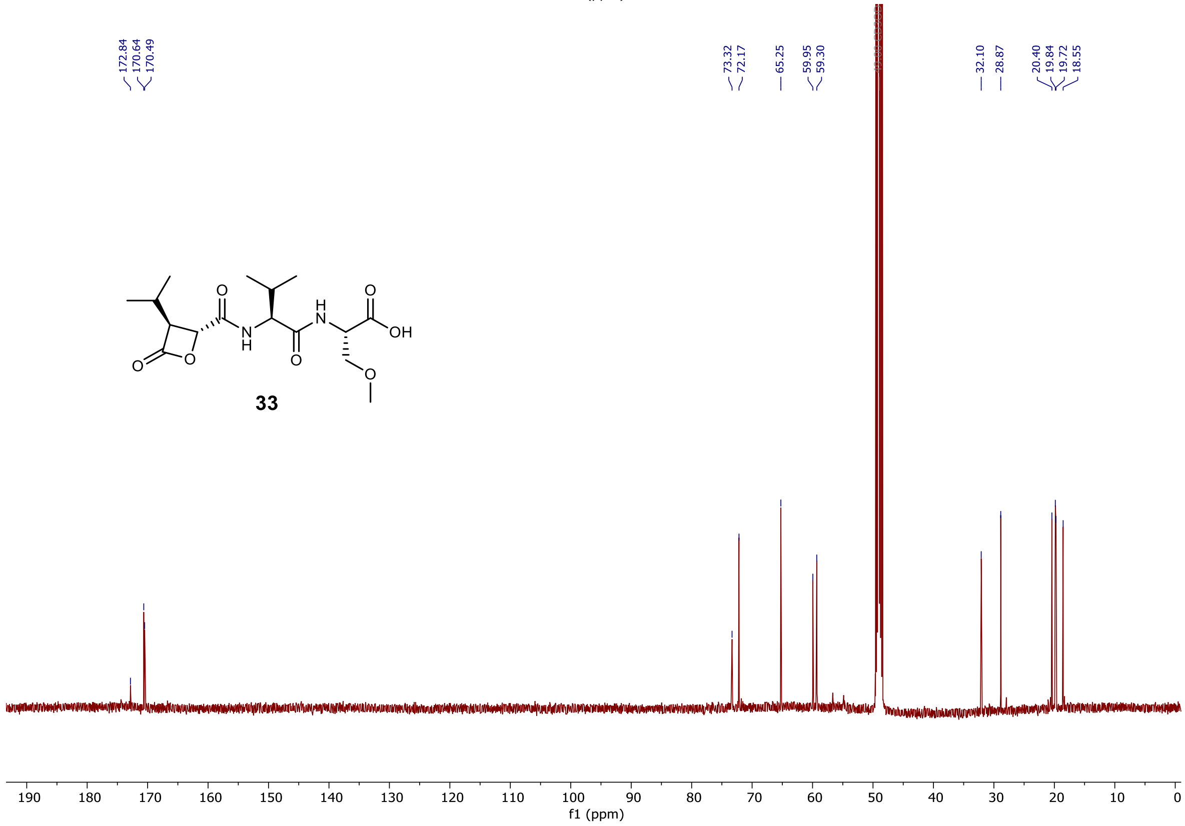

Supplementary Fig. 64. NMR of compound 33.

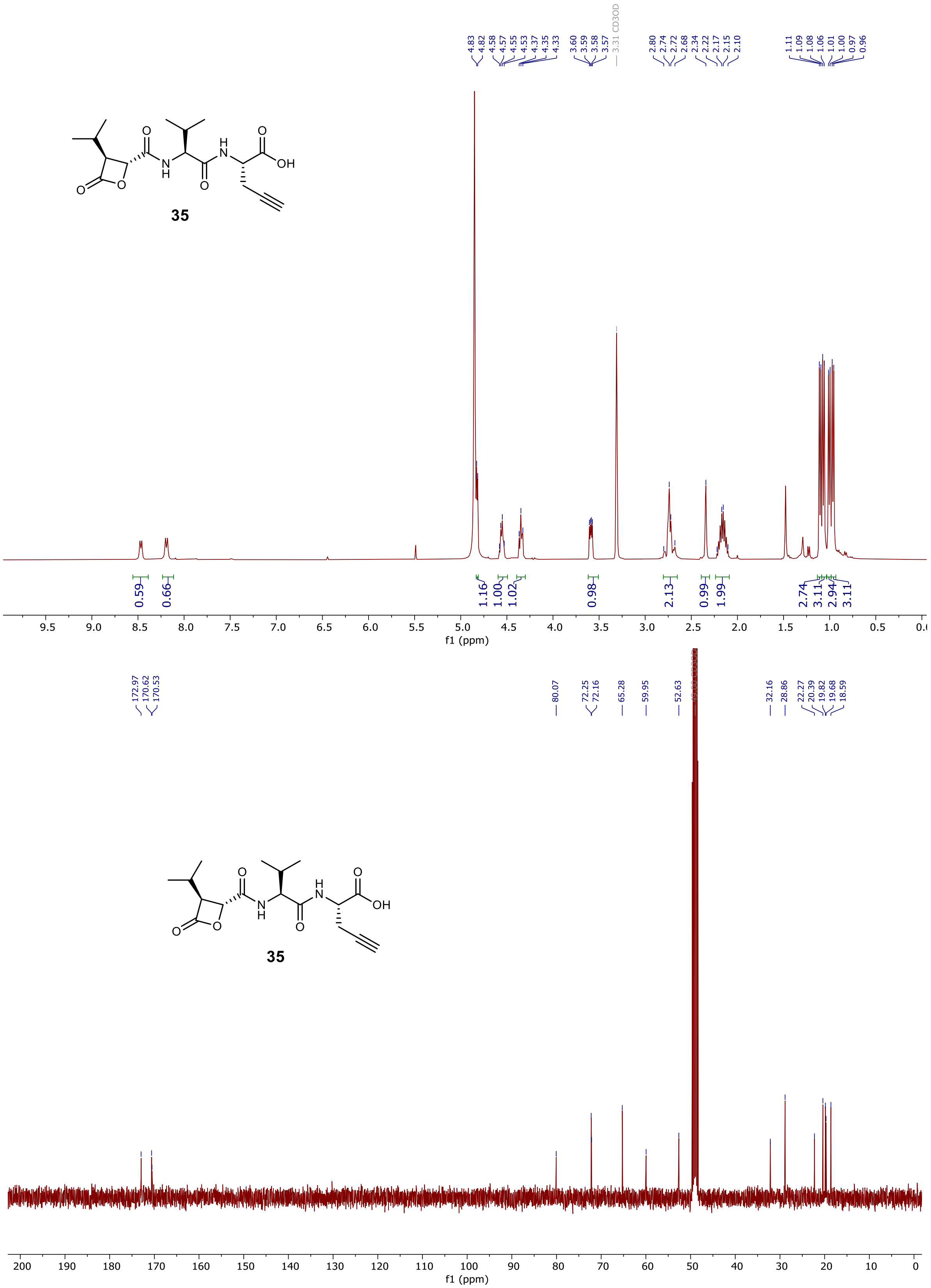

Supplementary Fig. 65. NMR of compound 35.

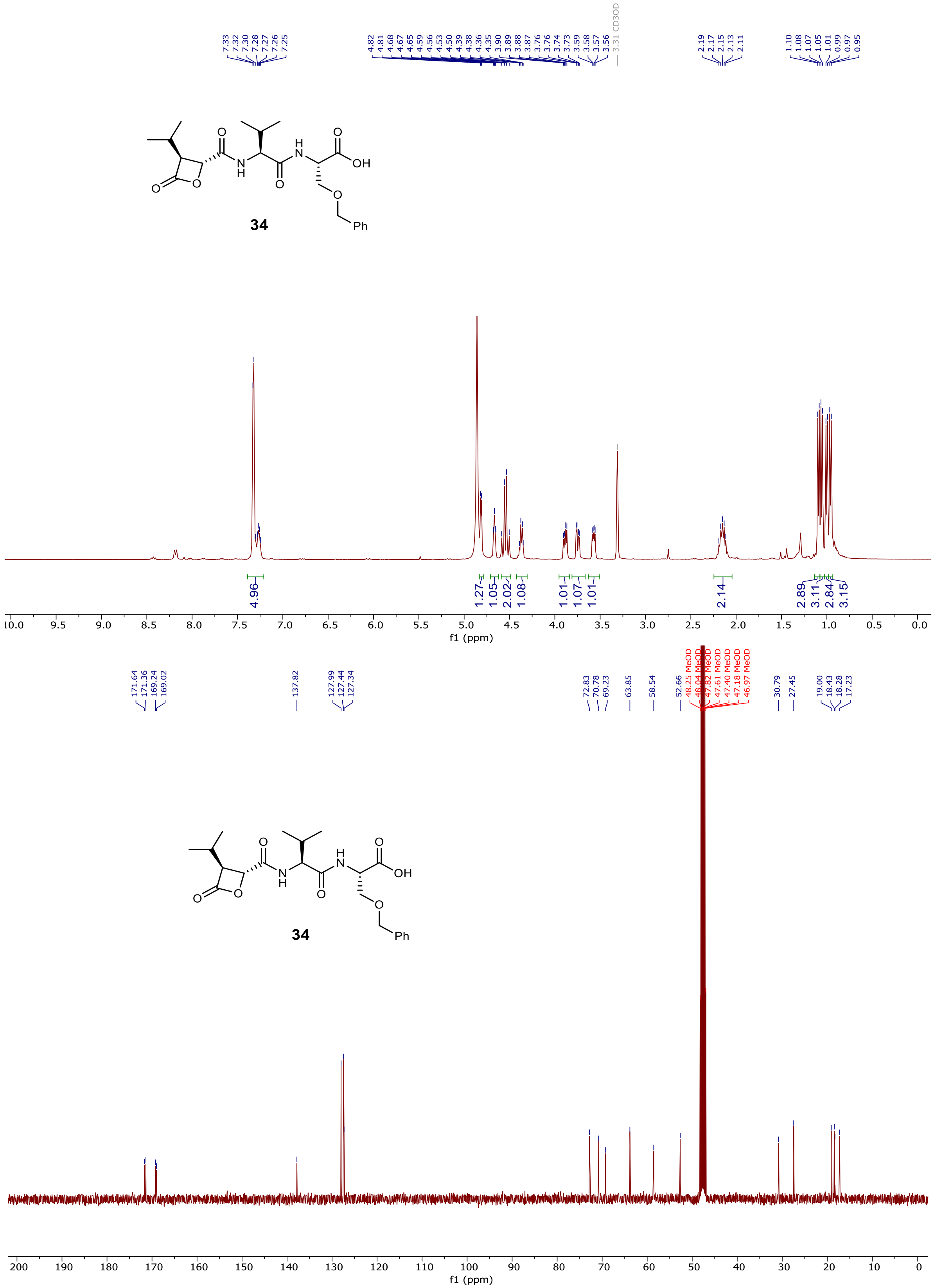

Supplementary Fig. 66. NMR of compound 34.

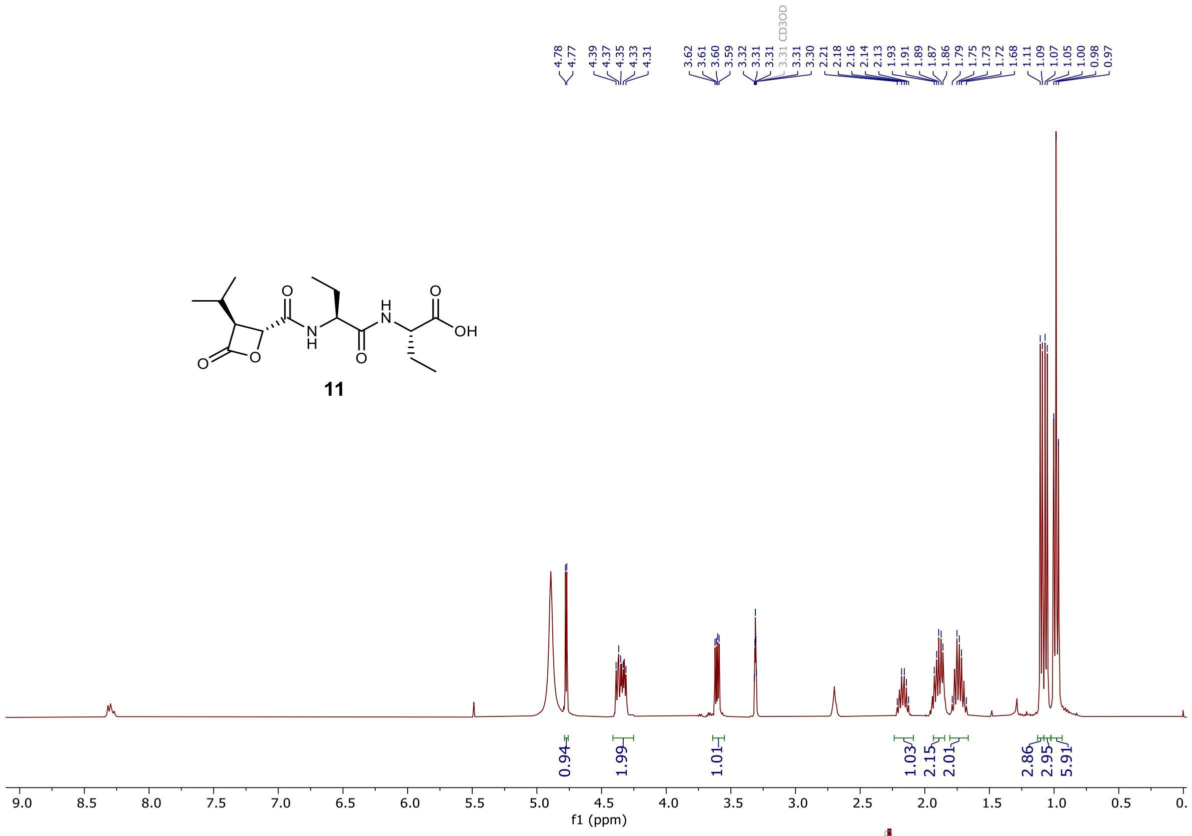

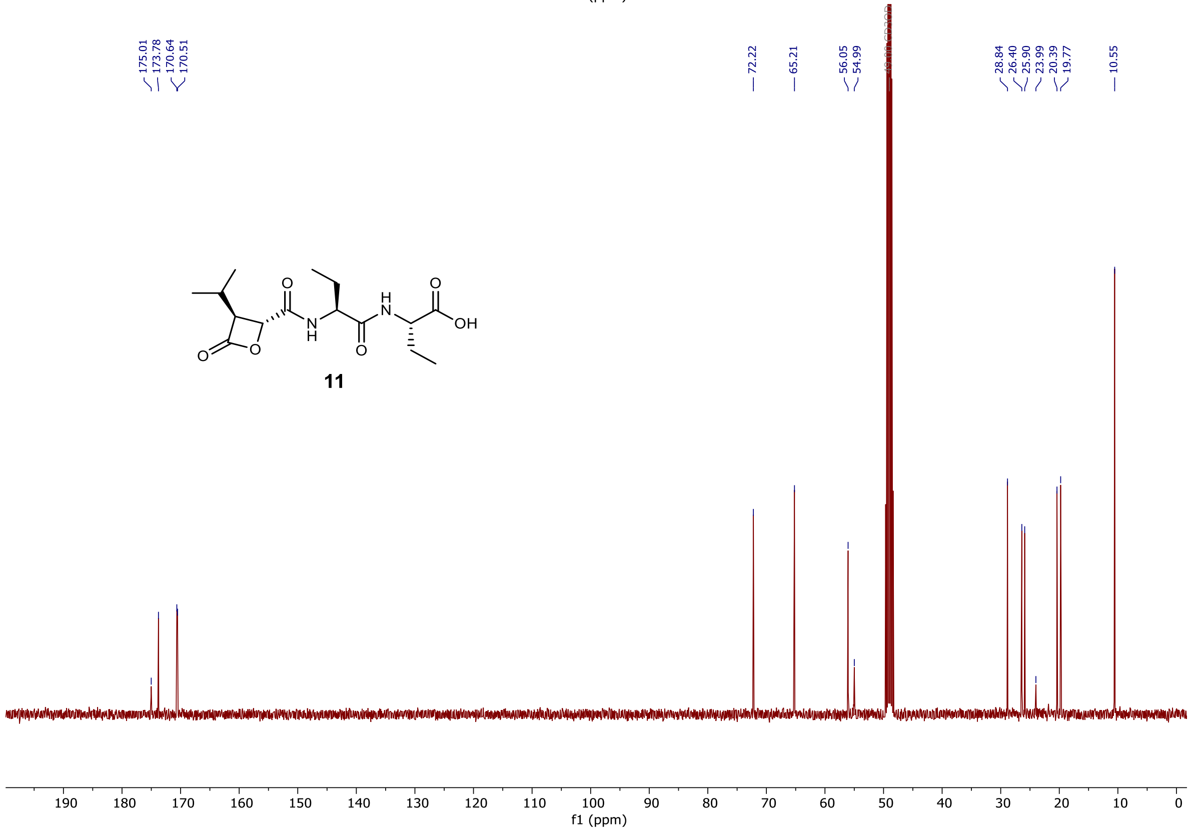

Supplementary Fig. 67. NMR of compound 11.

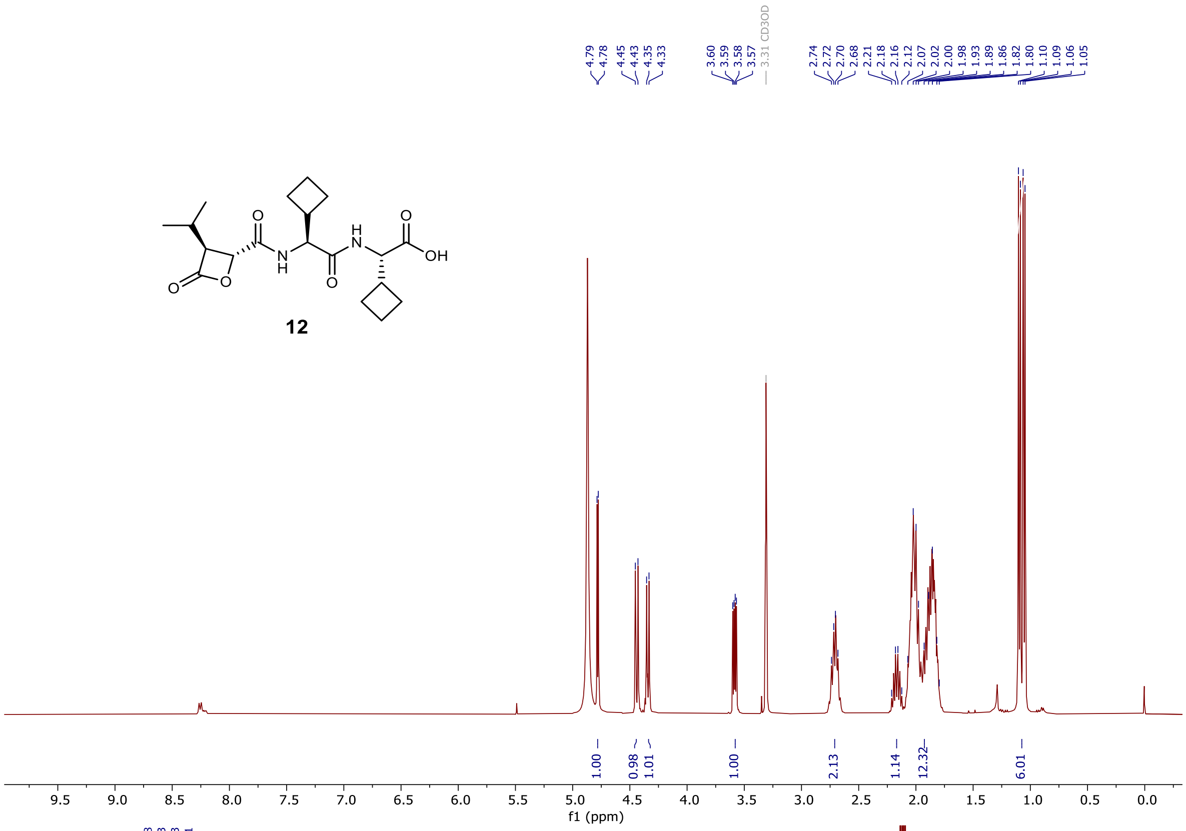

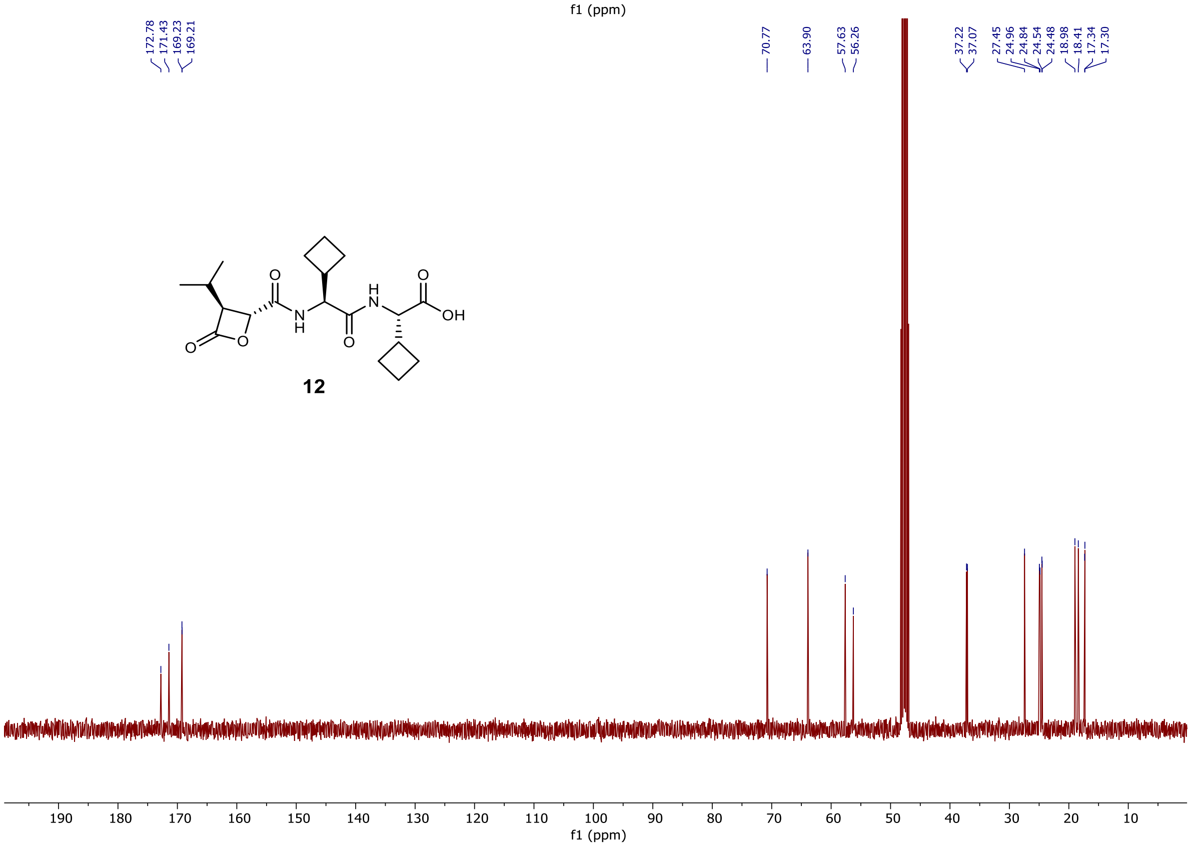

Supplementary Fig. 68. NMR of compound 12.

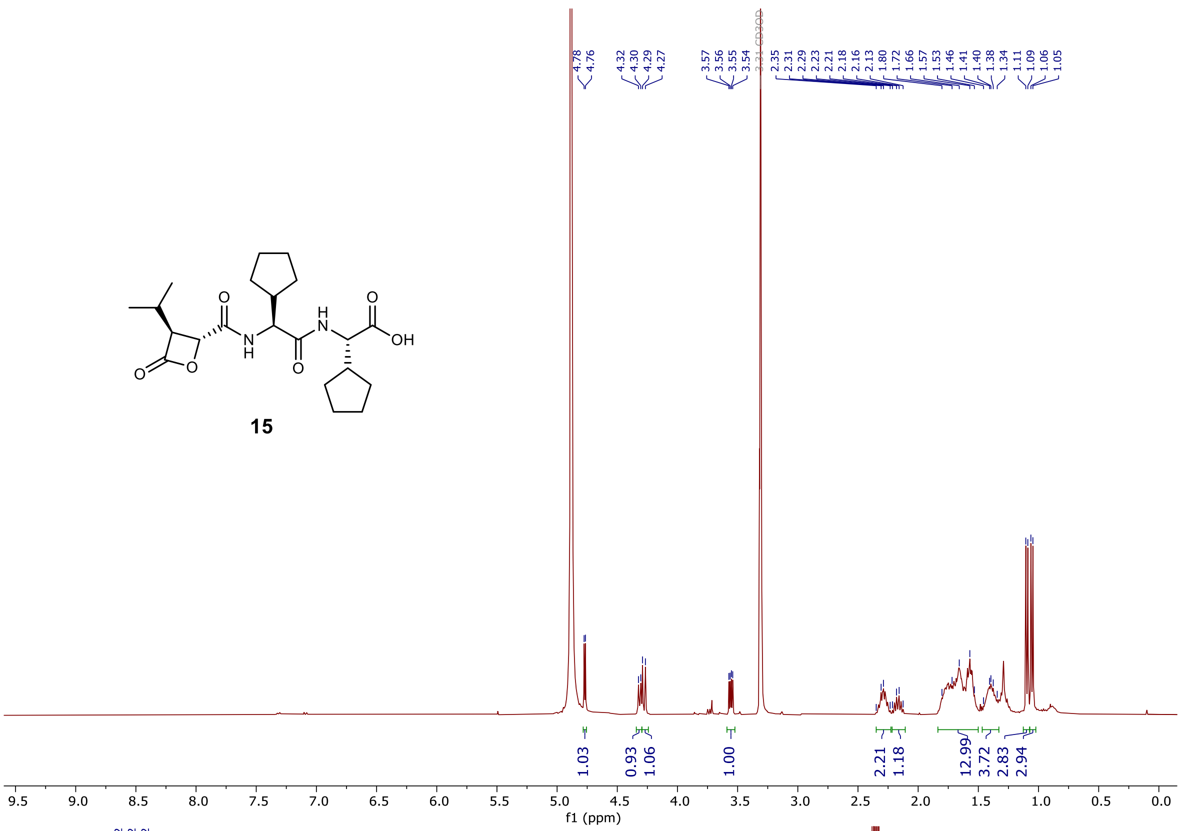

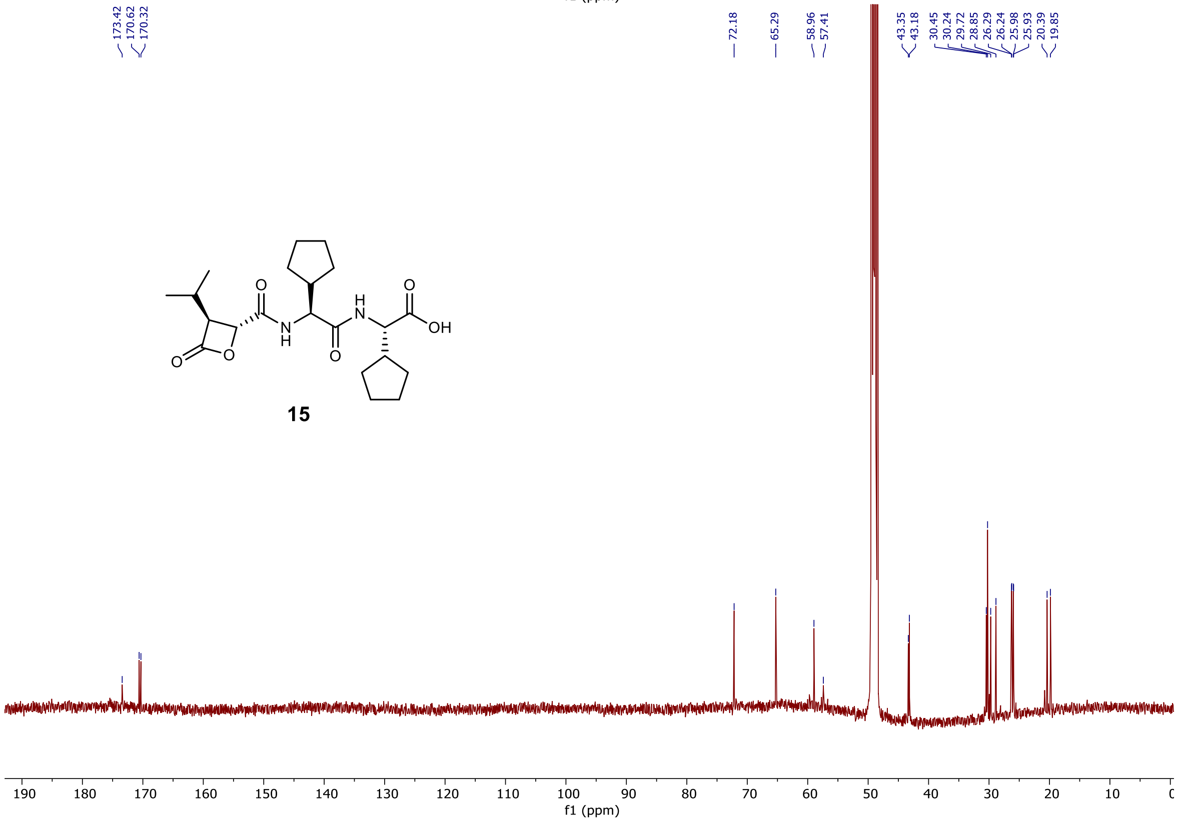

Supplementary Fig. 69. NMR of compound 15.

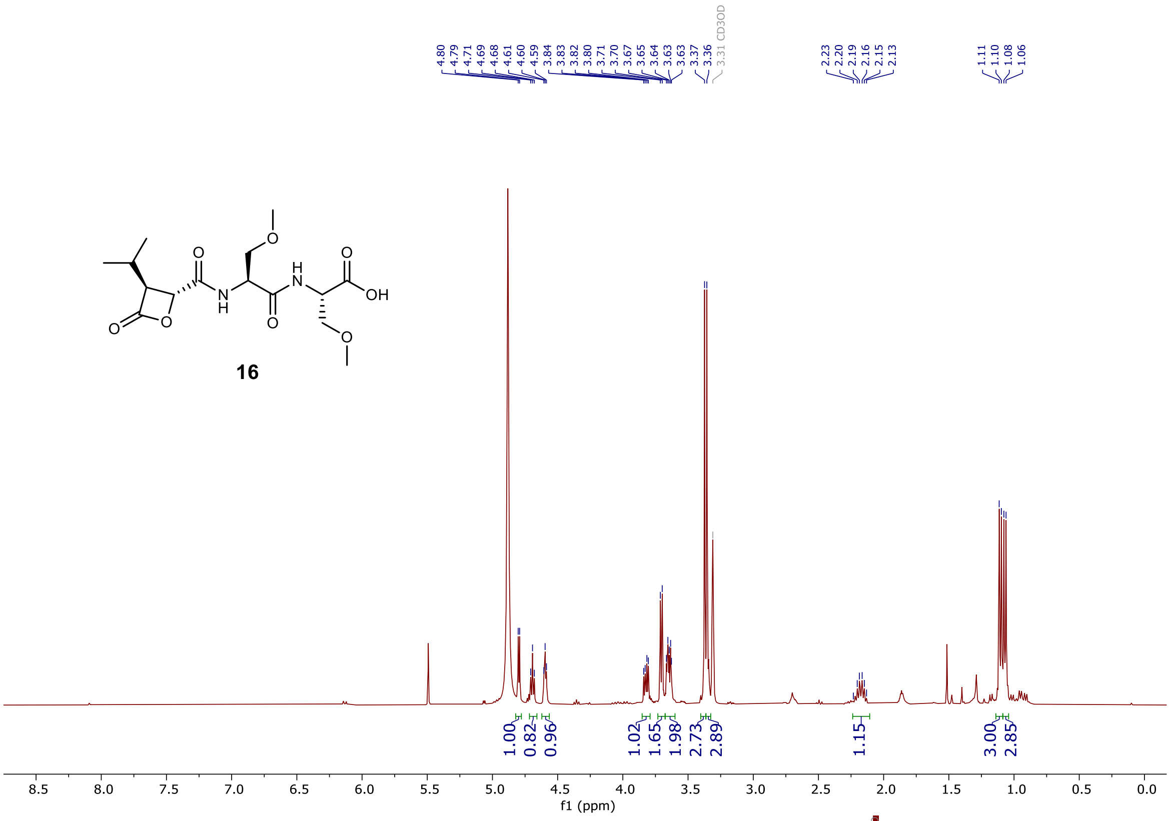

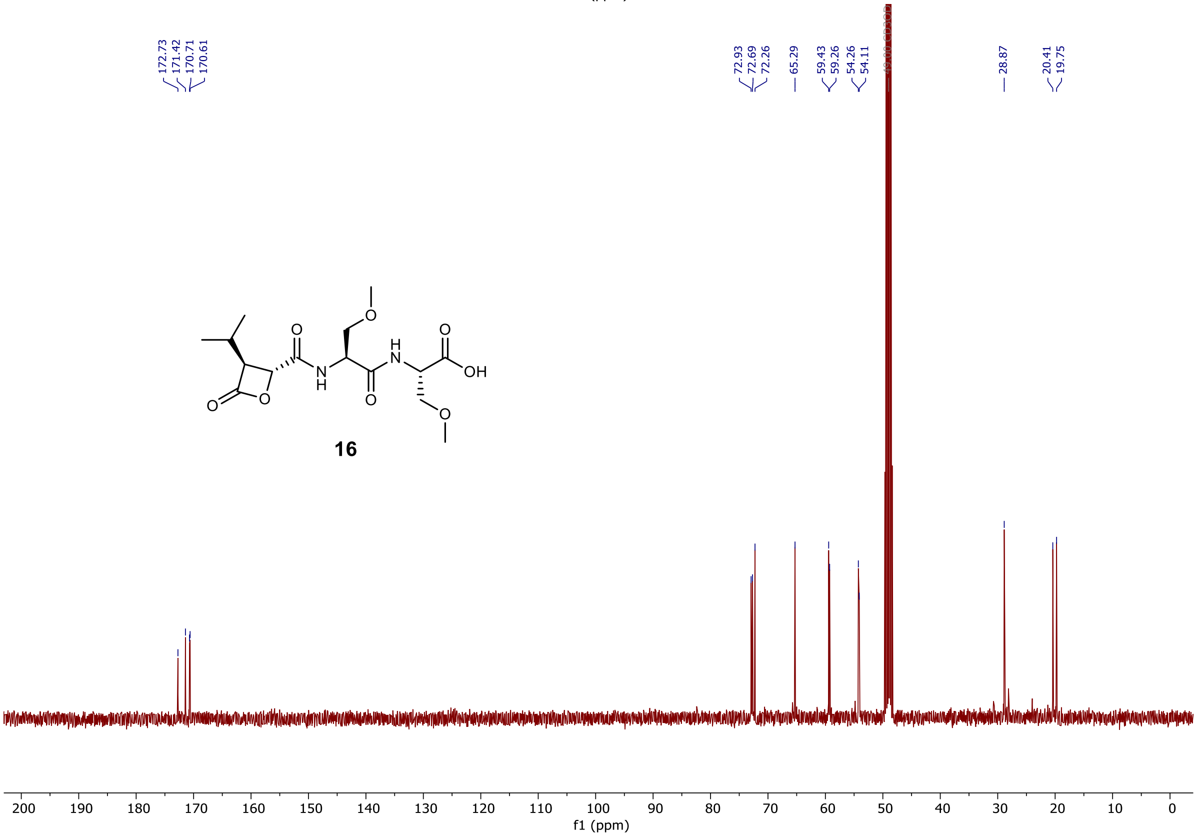

Supplementary Fig. 70. NMR of compound 16.

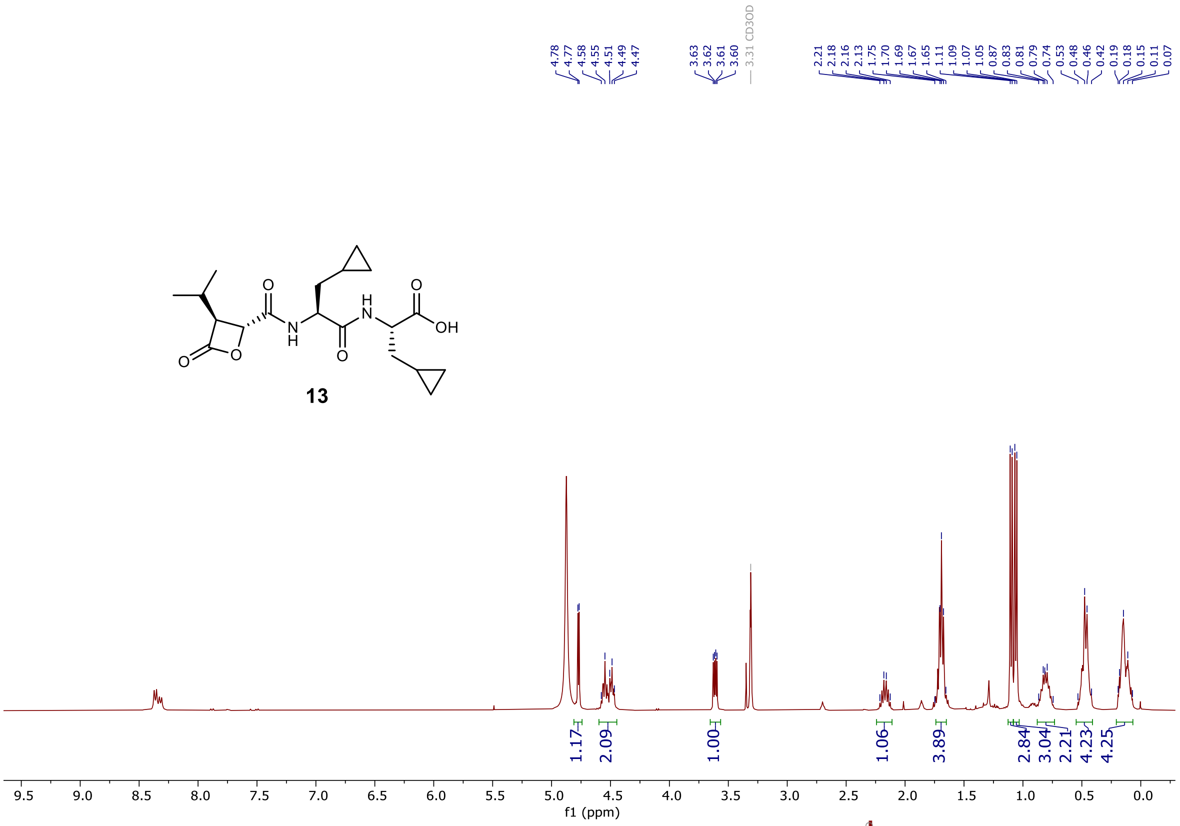

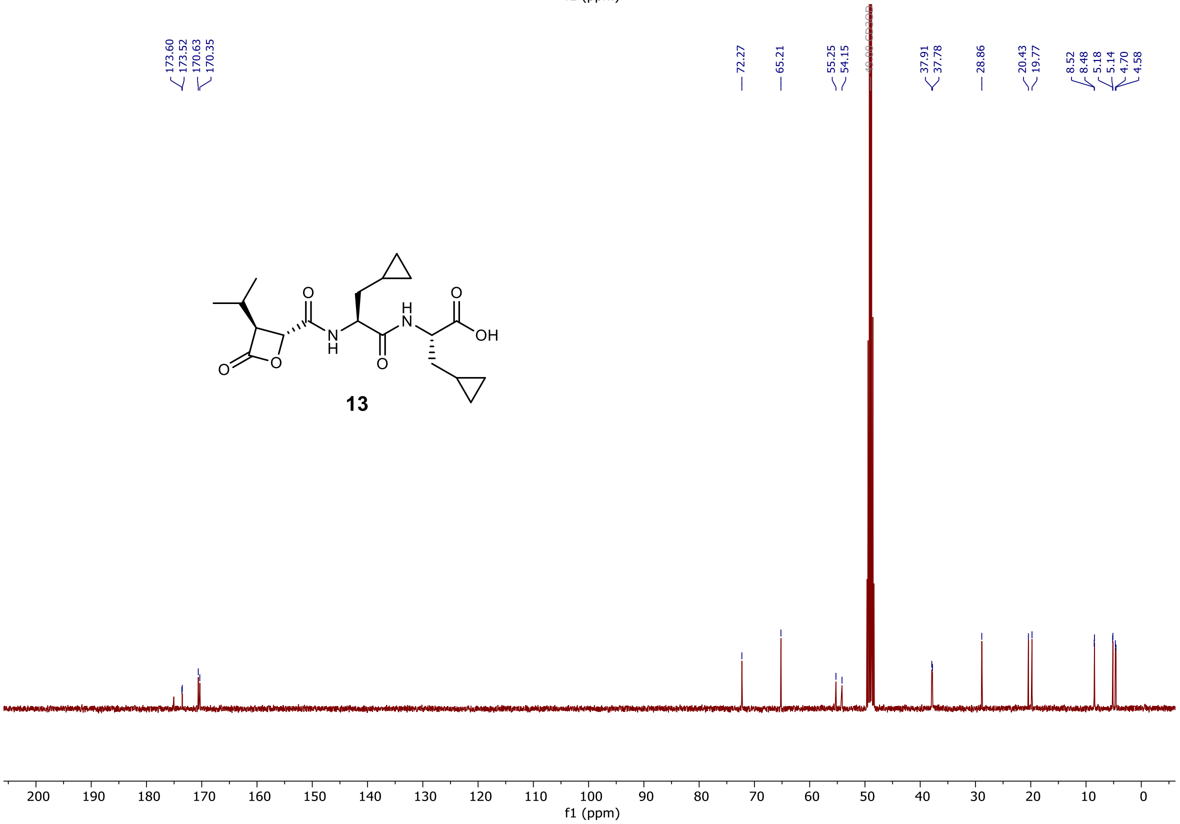

Supplementary Fig. 71. NMR of compound 13.

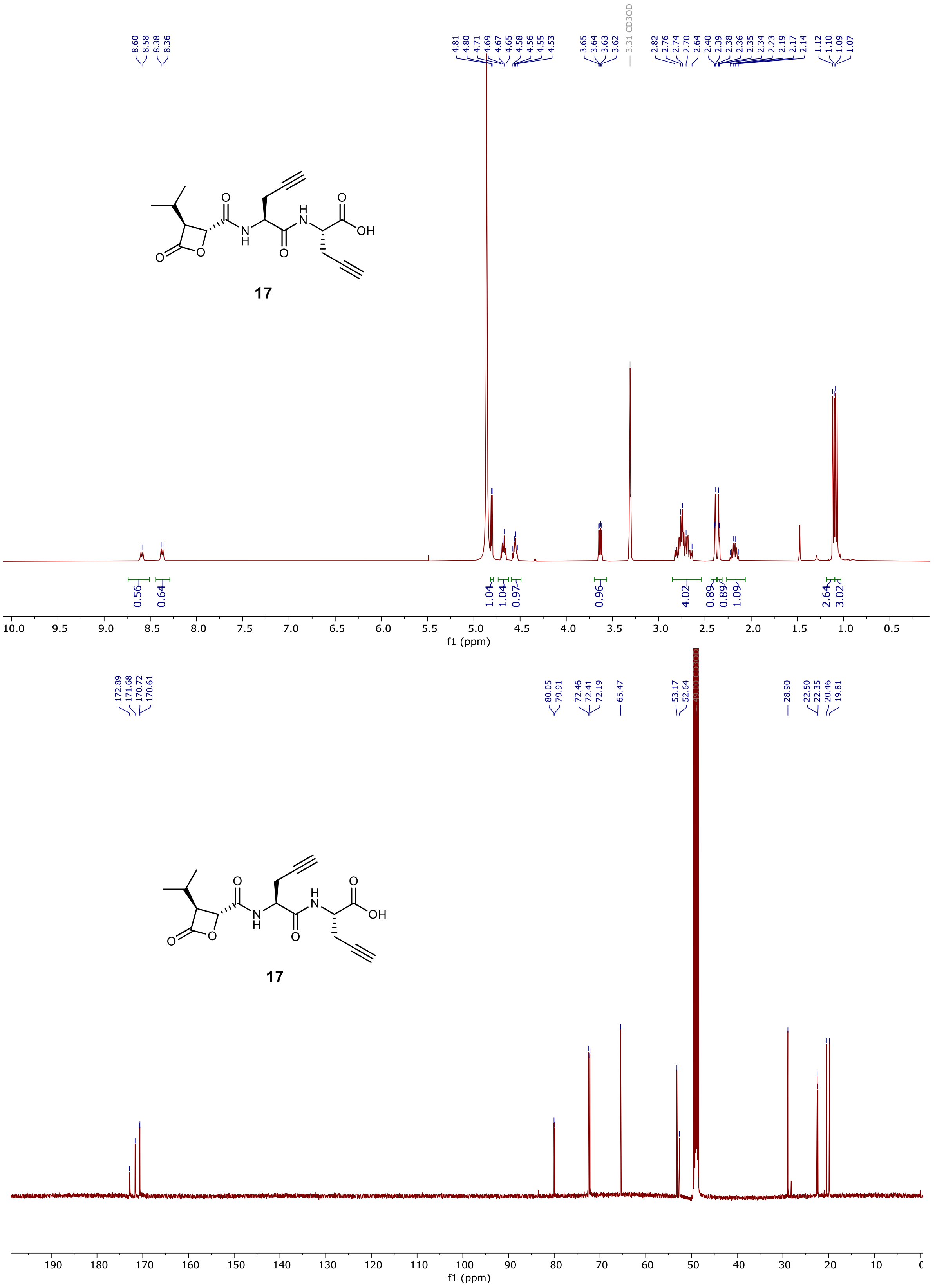

Supplementary Fig. 72. NMR of compound 17.

LC-MS/MS data for assays described in Extended Data Fig. 9-10

Observed mass (m/z) of precursor ion $[M+H]^+$ is indicated in blue.

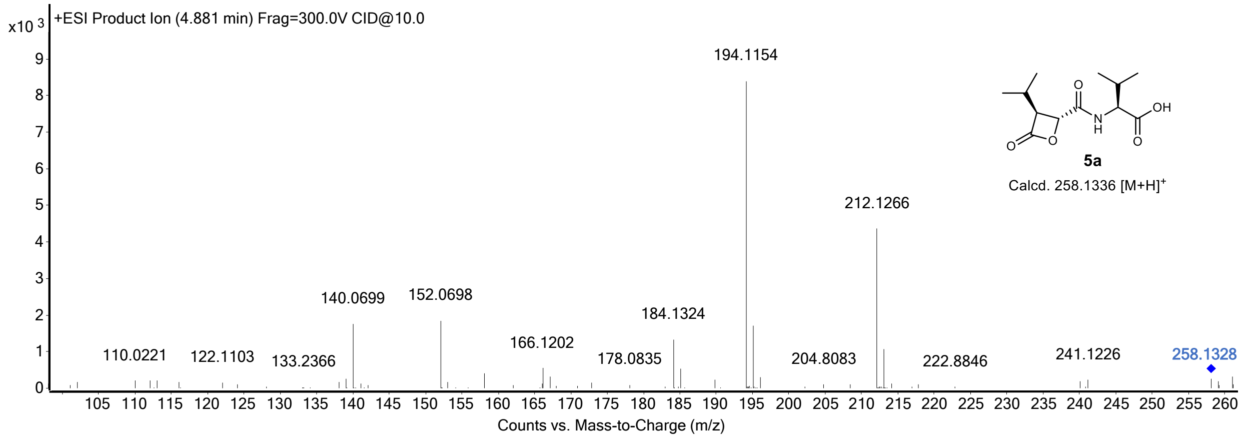

Supplementary Fig. 73. HRMS/MS data of compound 5a.

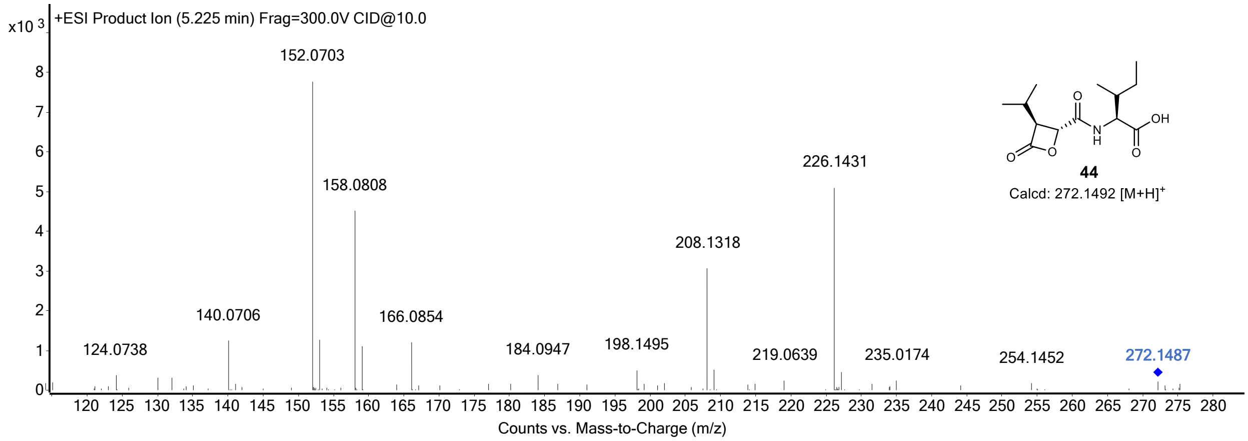

Supplementary Fig. 74. HRMS/MS data of compound 44.

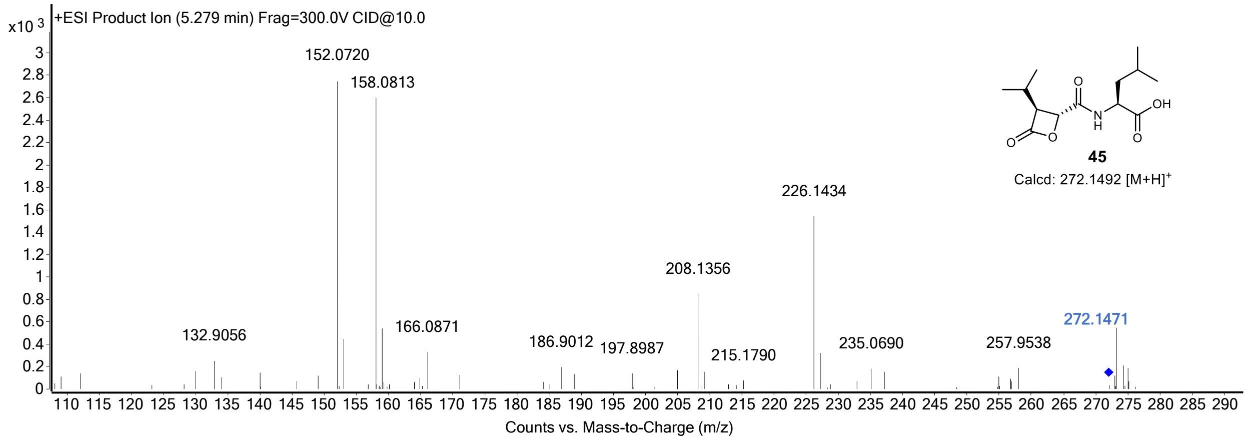

Supplementary Fig. 75. HRMS/MS data of compound 45.

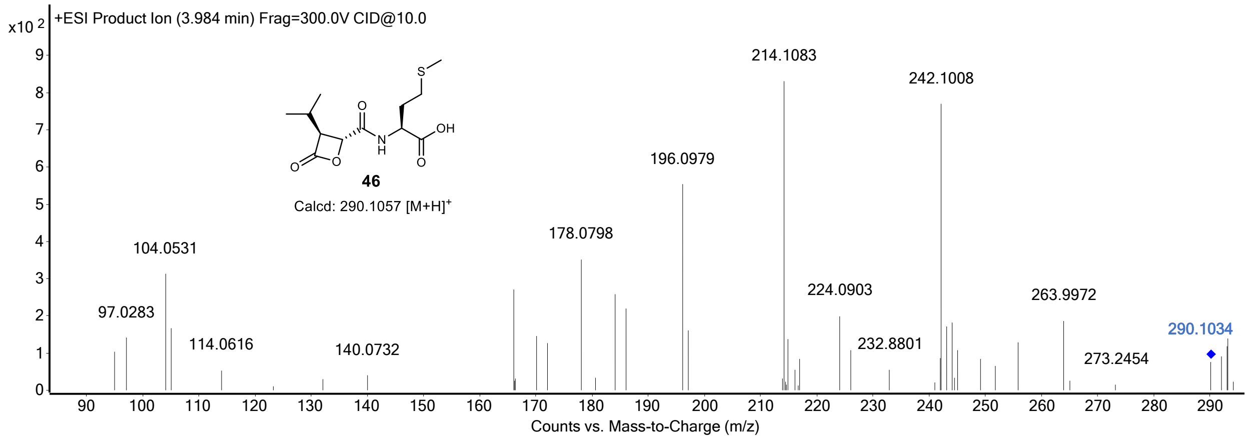

Supplementary Fig. 76. HRMS/MS data of compound 46.

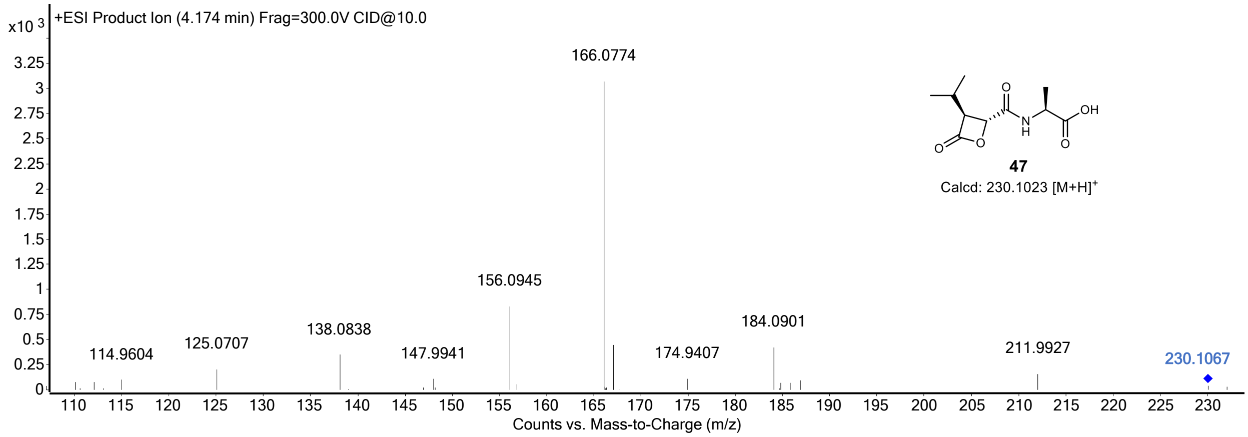

Supplementary Fig. 77. HRMS/MS data of compound 47.

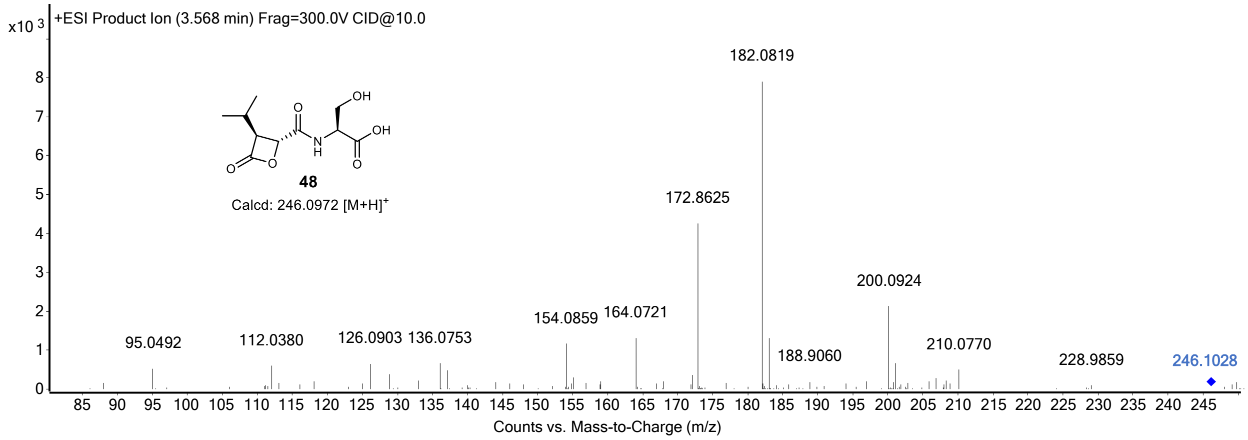

Supplementary Fig. 78. HRMS/MS data of compound 48.

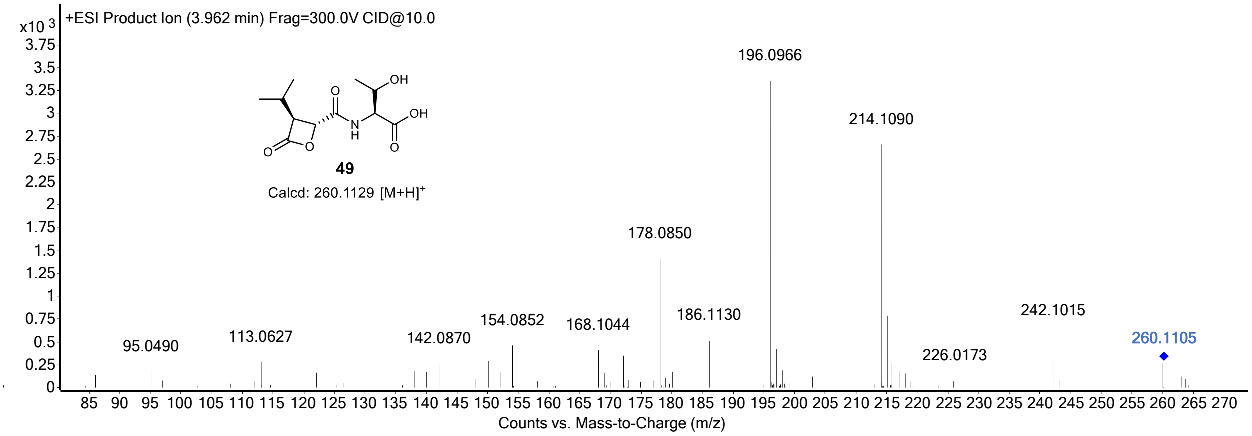

Supplementary Fig. 79. HRMS/MS data of compound 49.

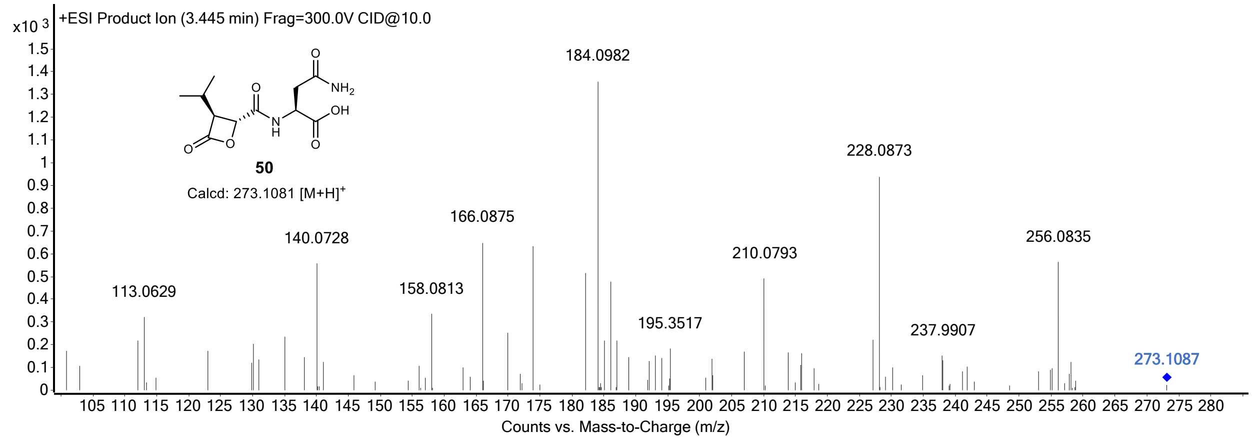

Supplementary Fig. 80. HRMS/MS data of compound 50.

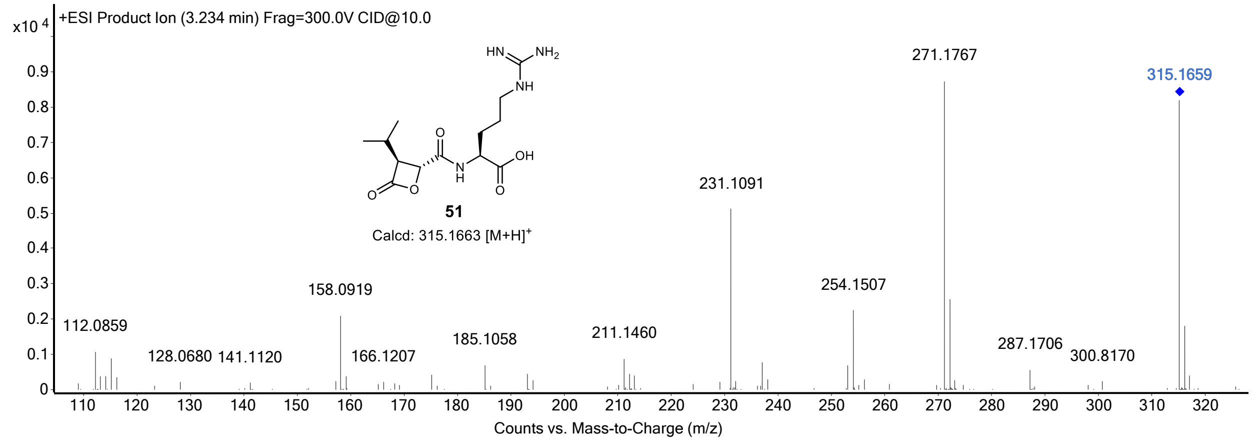

Supplementary Fig. 81. HRMS/MS data of compound 51.

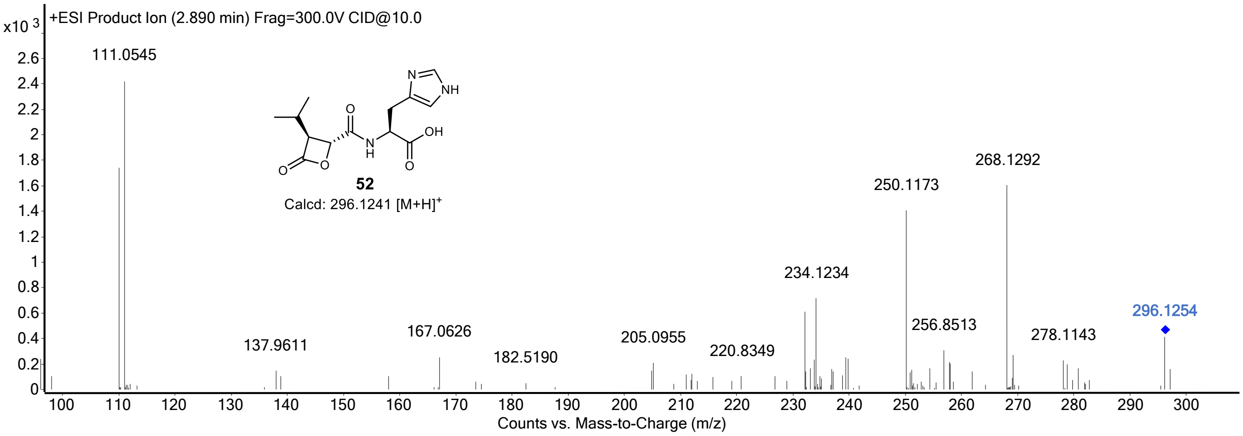

Supplementary Fig. 82. HRMS/MS data of compound 52.

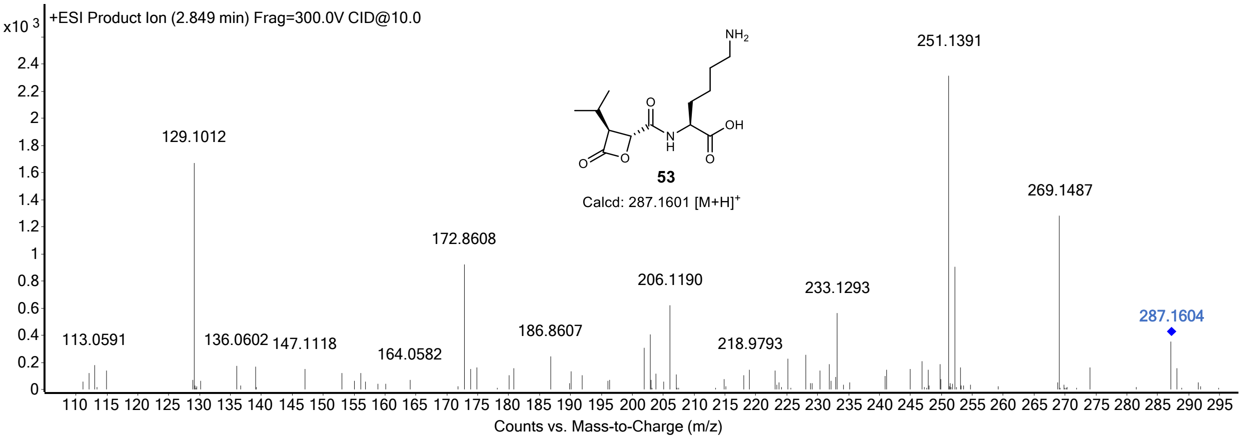

Supplementary Fig. 83. HRMS/MS data of compound 53.

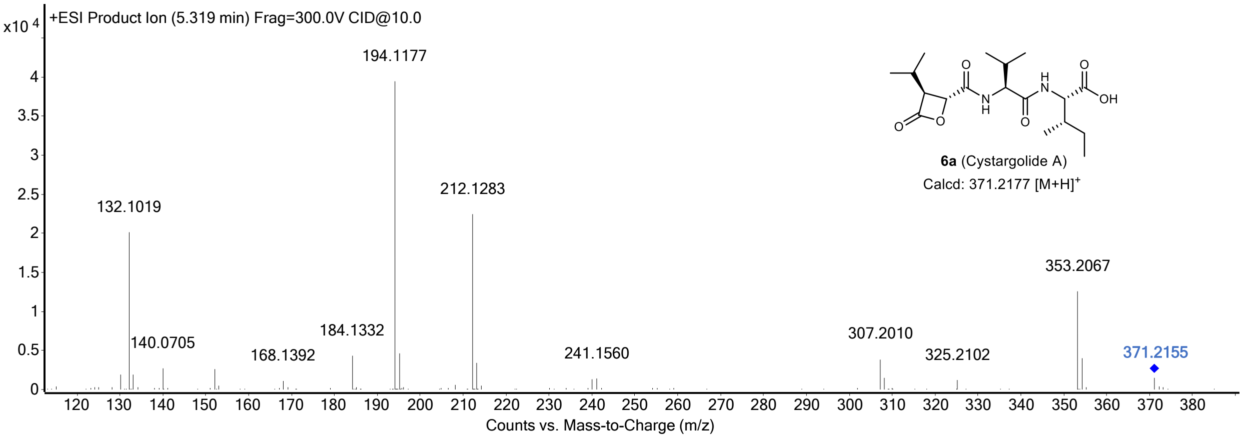

Supplementary Fig. 84. HRMS/MS data of compound 6a.

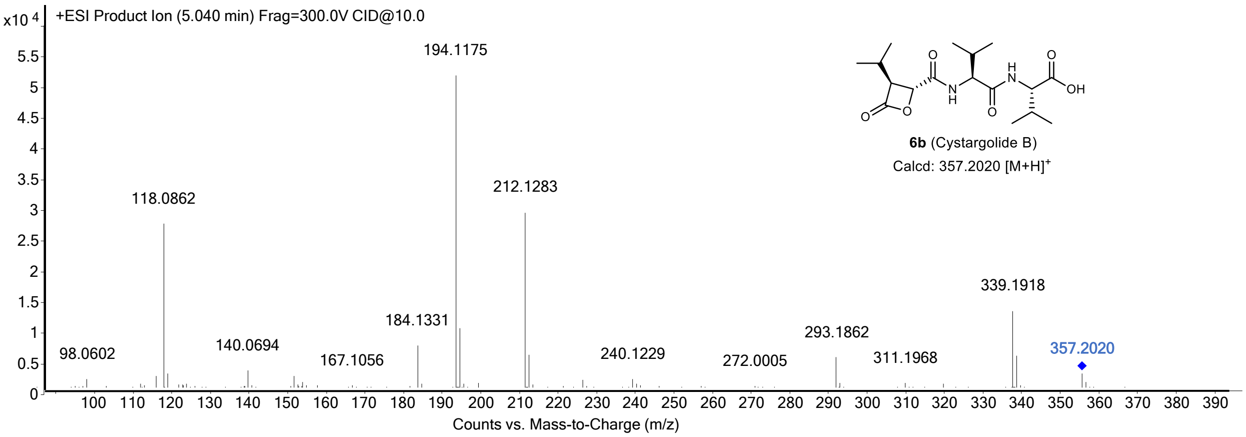

Supplementary Fig. 85. HRMS/MS data of compound 6b.

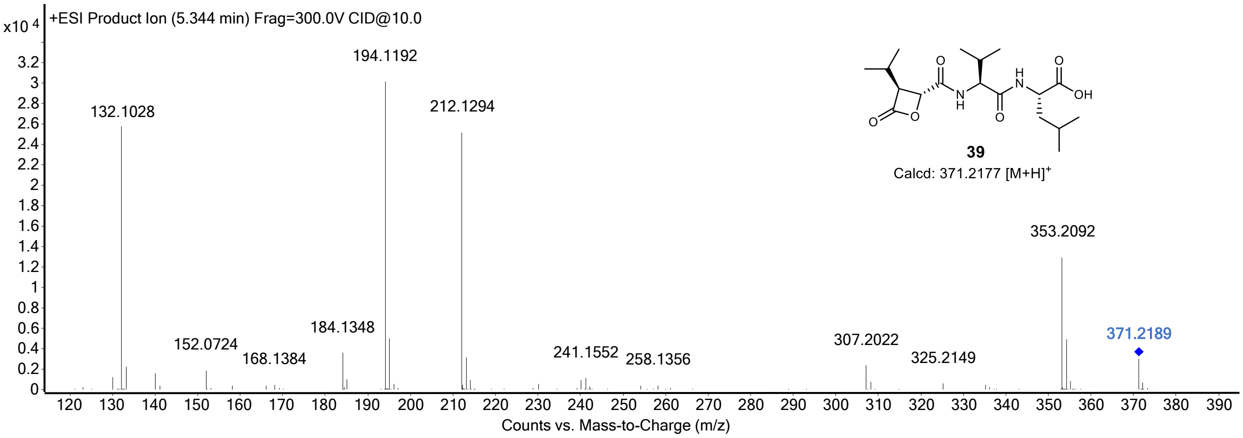

Supplementary Fig. 86. HRMS/MS data of compound 39.

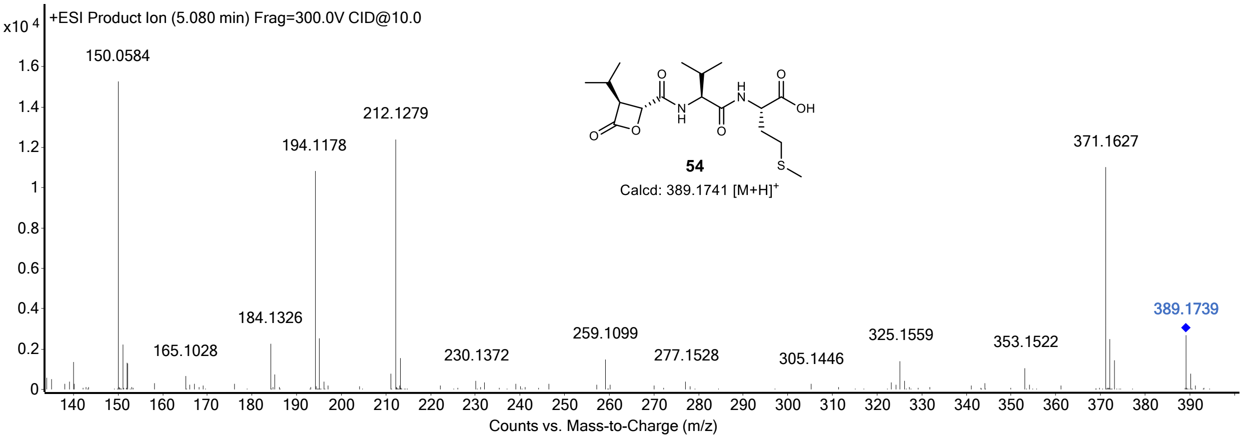

Supplementary Fig. 87. HRMS/MS data of compound 54.

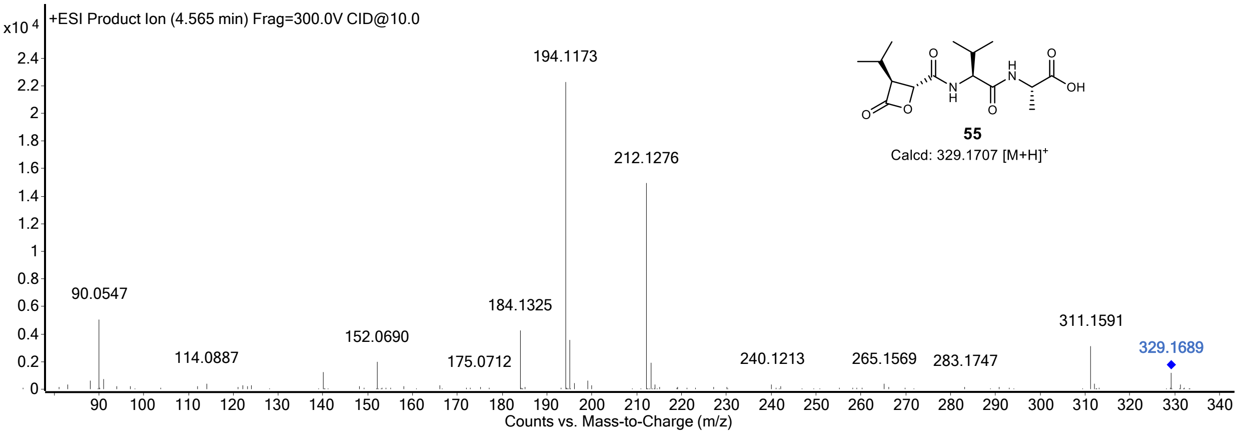

Supplementary Fig. 88. HRMS/MS data of compound 55.

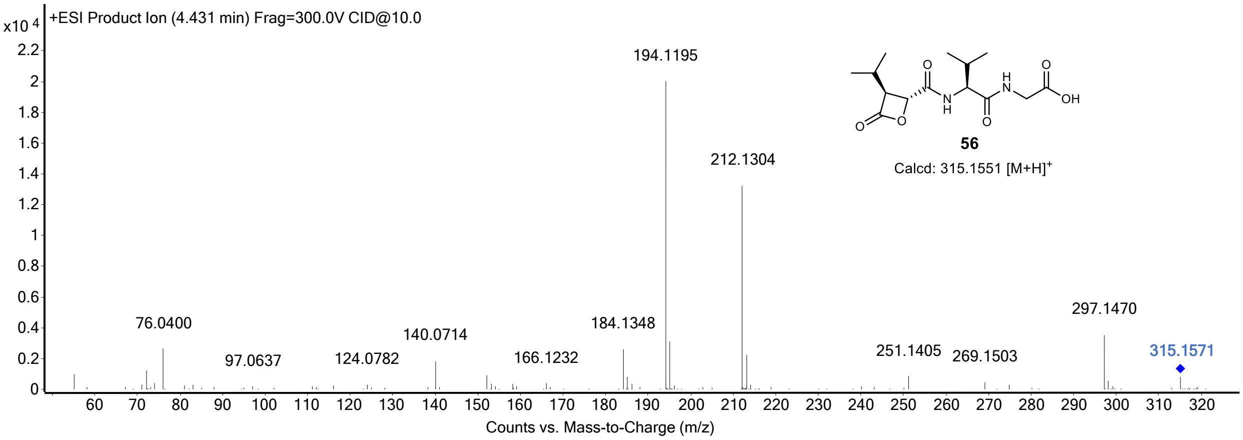

Supplementary Fig. 89. HRMS/MS data of compound 56.

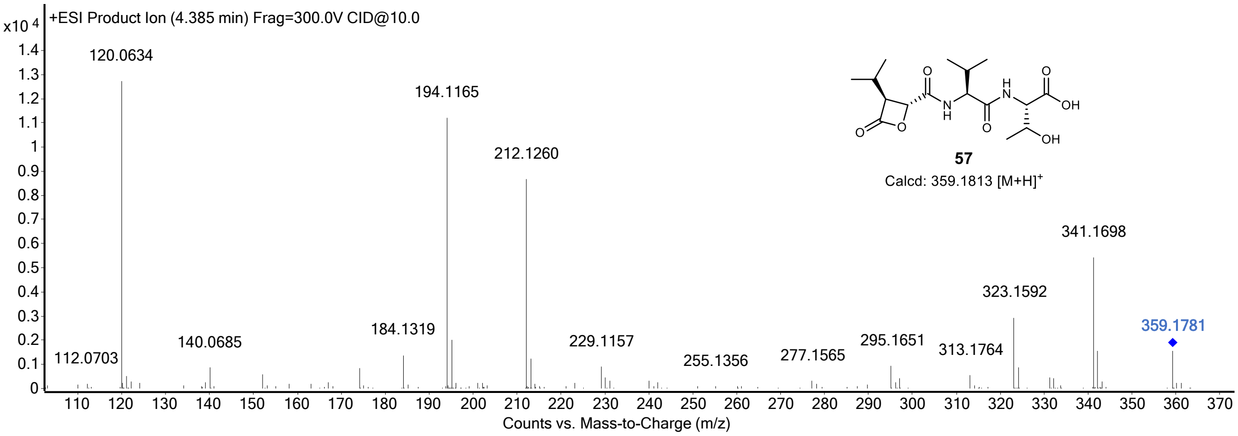

Supplementary Fig. 90. HRMS/MS data of compound 57.

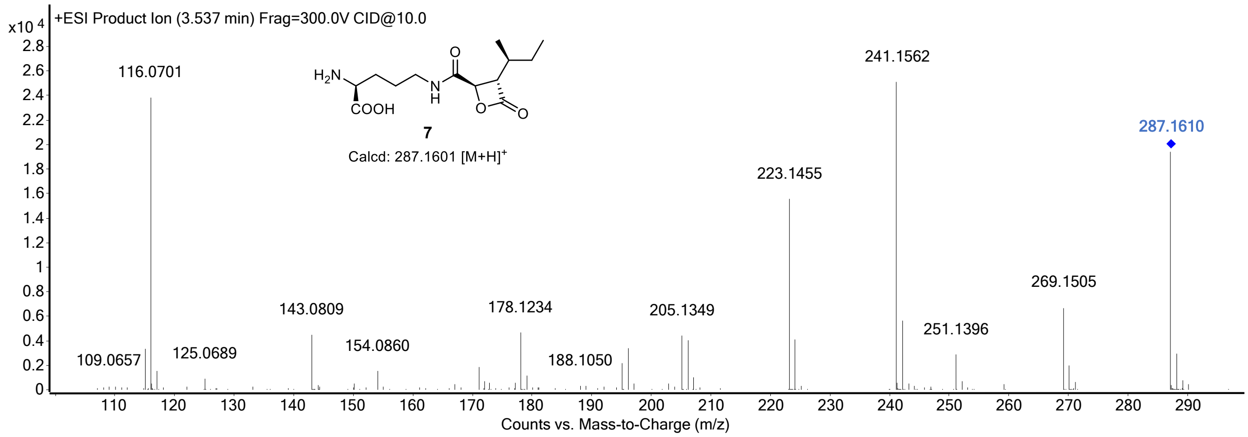

Supplementary Fig. 91. HRMS/MS data of compound 7.

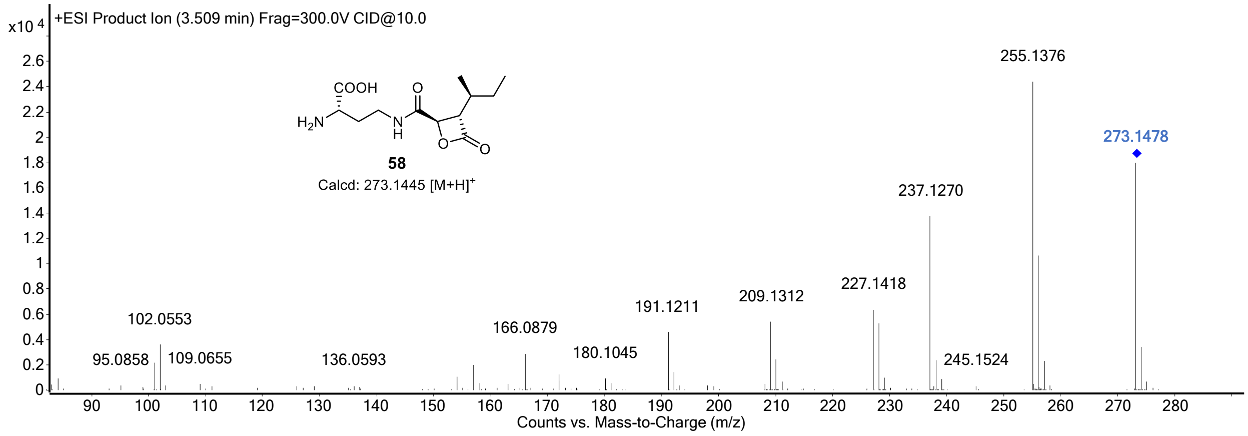

Supplementary Fig. 92. HRMS/MS data of compound 58.

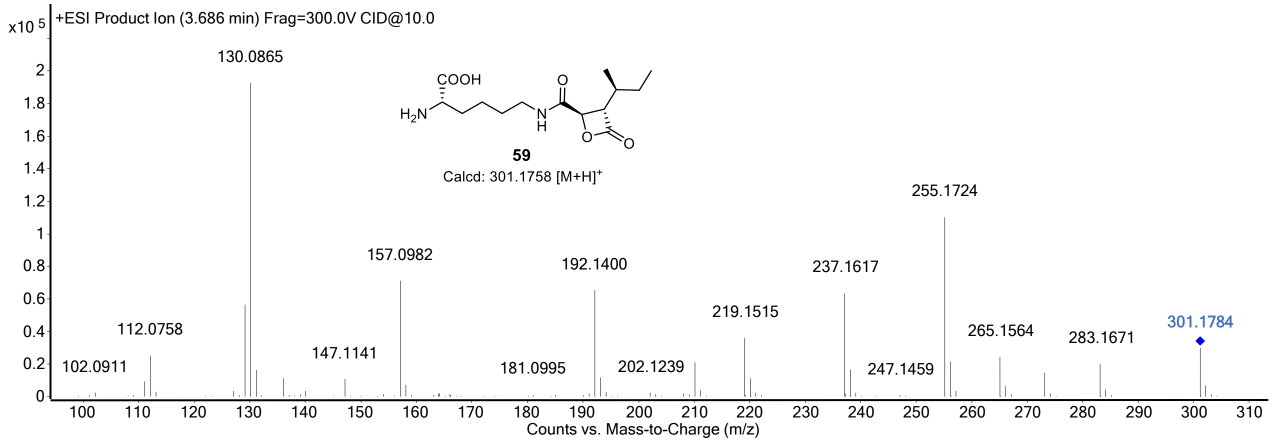

Supplementary Fig. 93. HRMS/MS data of compound 59.

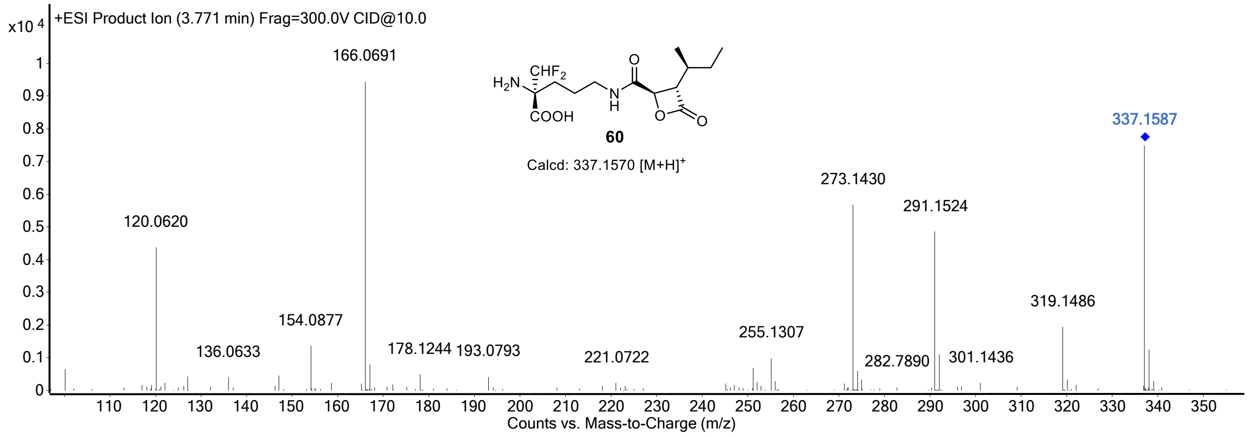

Supplementary Fig. 94. HRMS/MS data of compound 60.

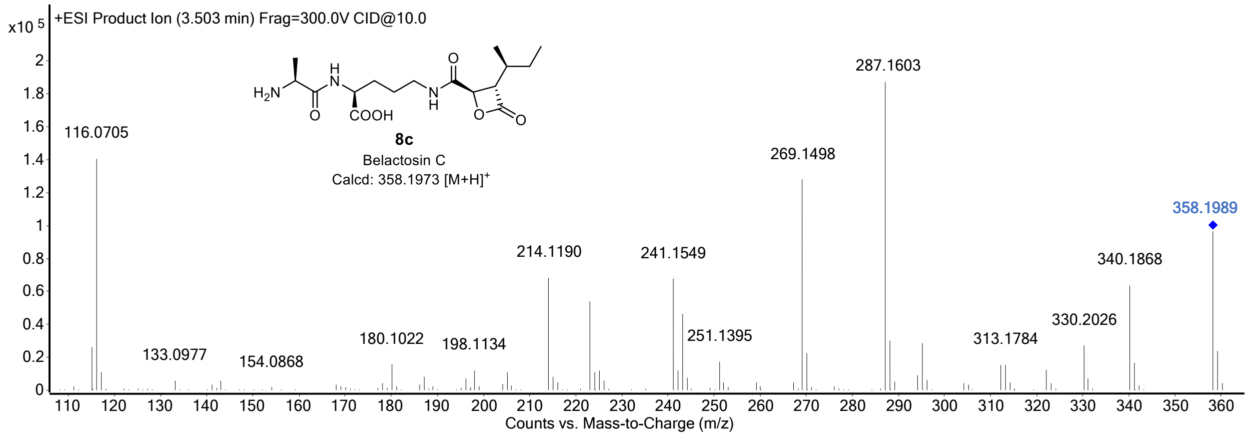

Supplementary Fig. 95. HRMS/MS data of compound 8c.

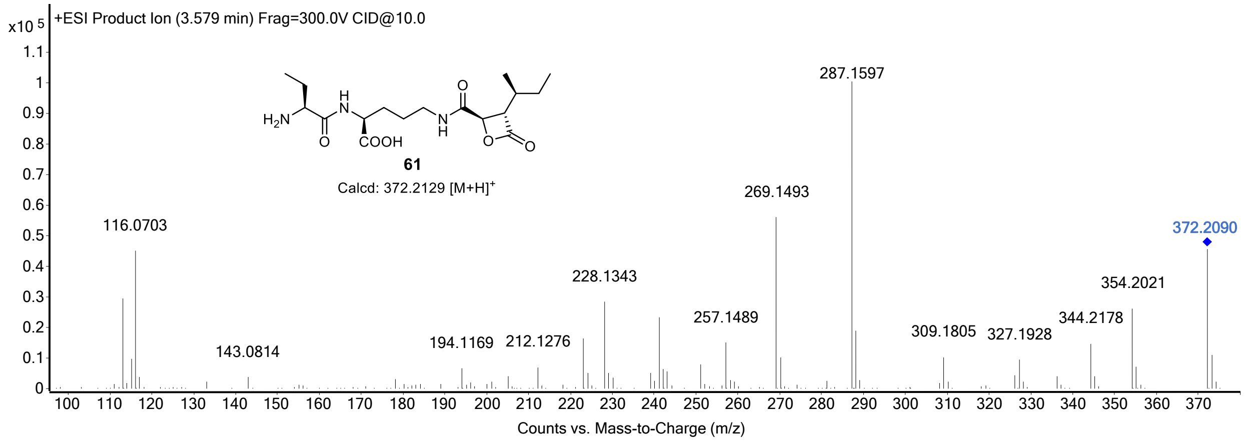

Supplementary Fig. 96. HRMS/MS data of compound 61.

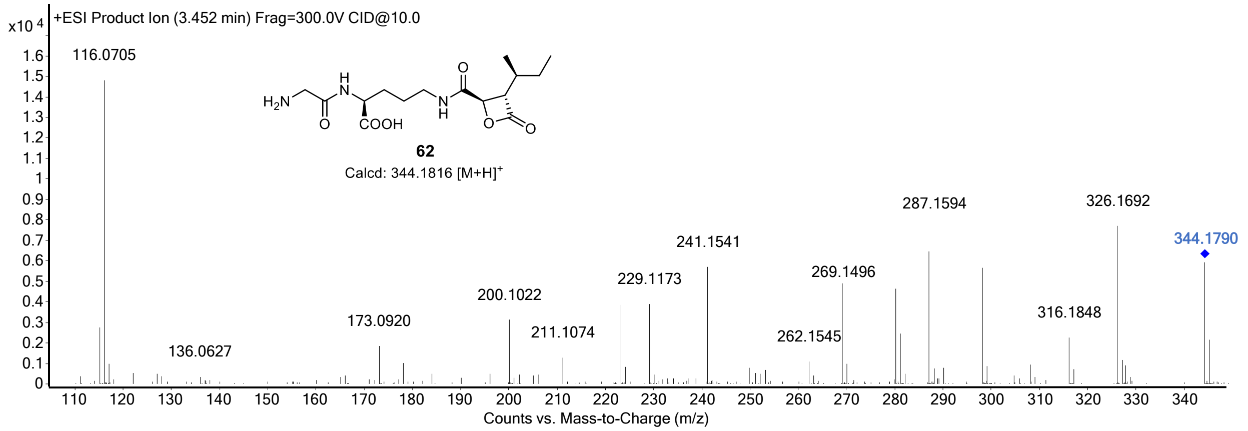

Supplementary Fig. 97. HRMS/MS data of compound 62.

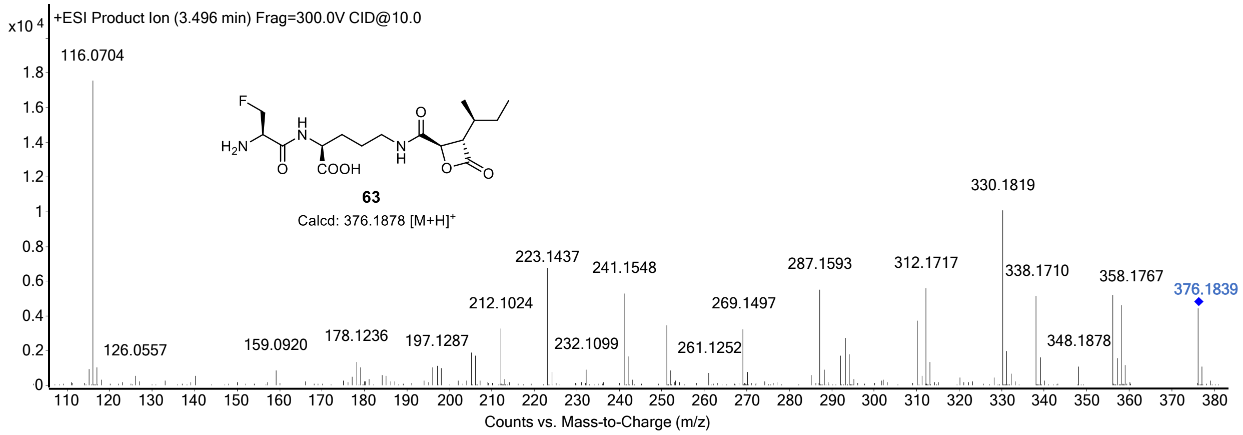

Supplementary Fig. 98. HRMS/MS data of compound 63.

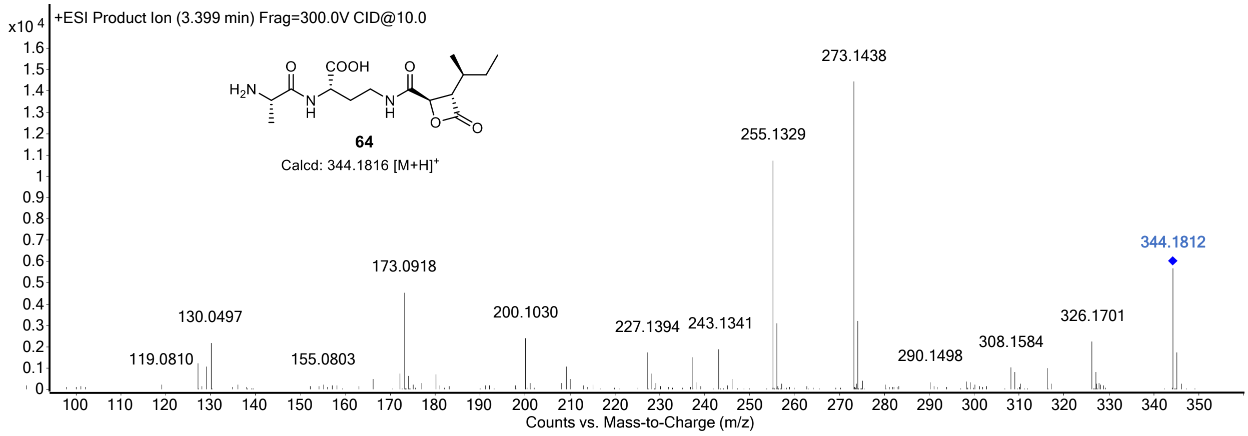

Supplementary Fig. 99. HRMS/MS data of compound 64.

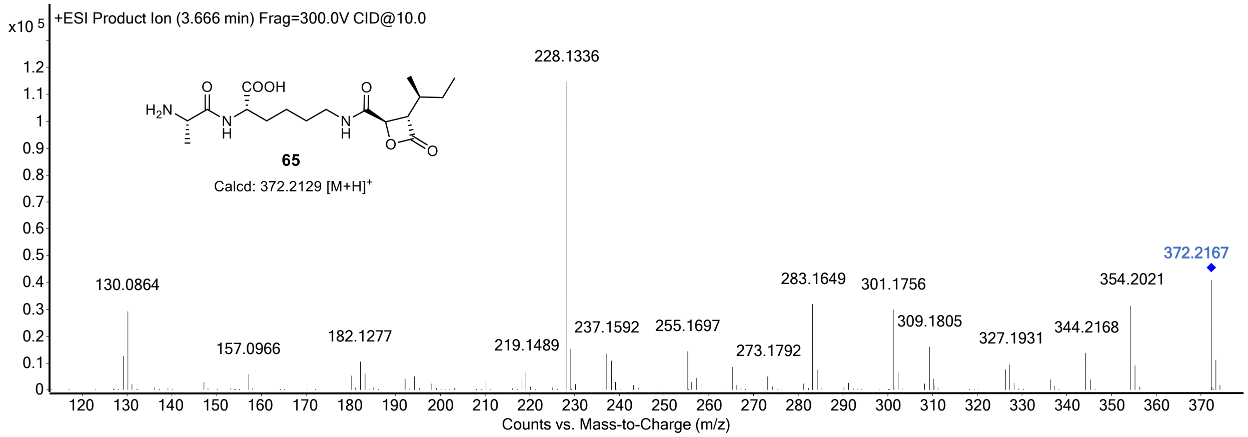

Supplementary Fig. 100. HRMS/MS data of compound 65.

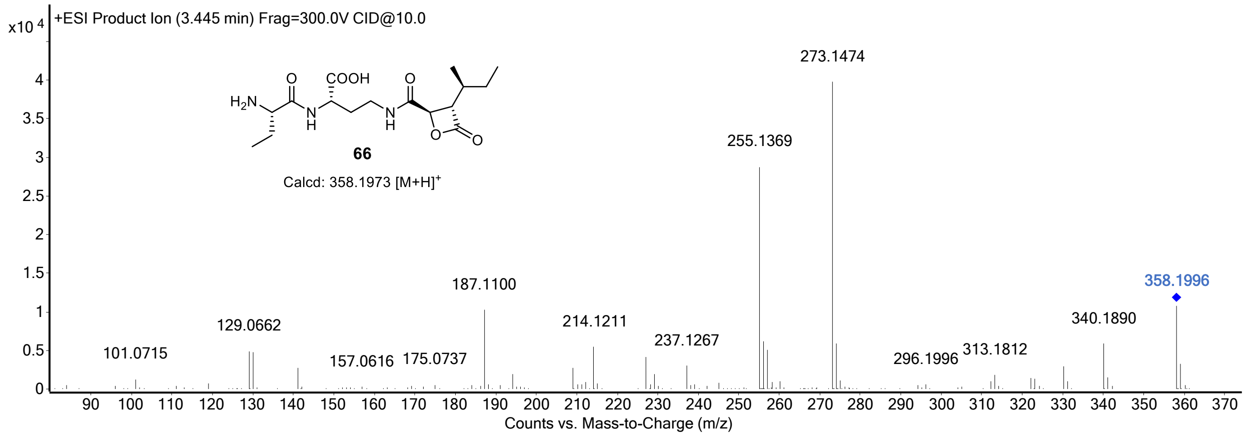

Supplementary Fig. 101. HRMS/MS data of compound 66.

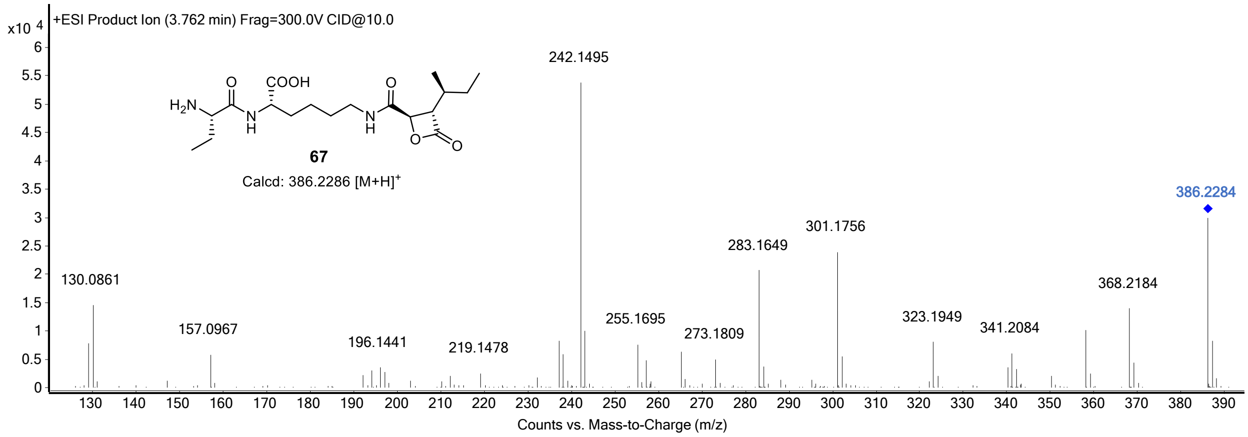

Supplementary Fig. 102. HRMS/MS data of compound 67.

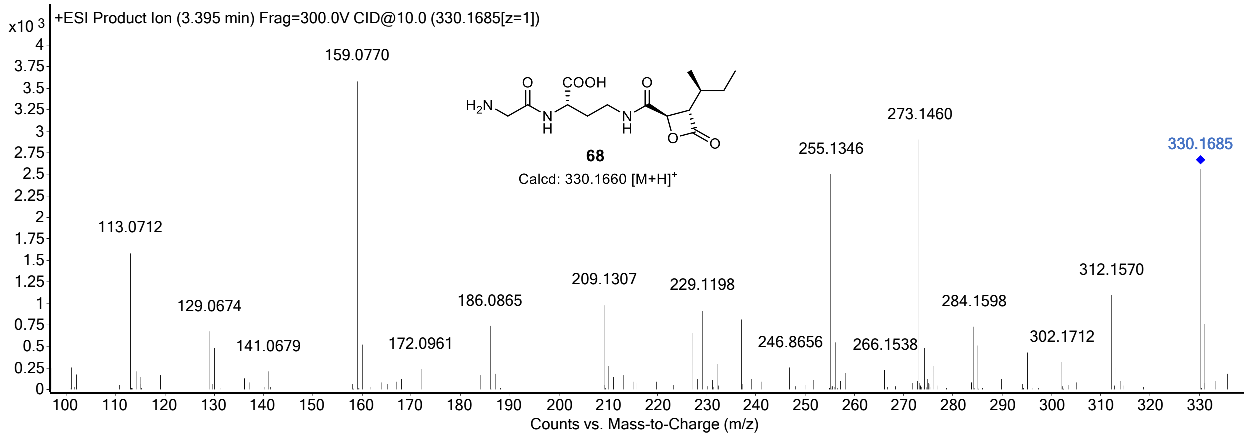

Supplementary Fig. 103. HRMS/MS data of compound 68.

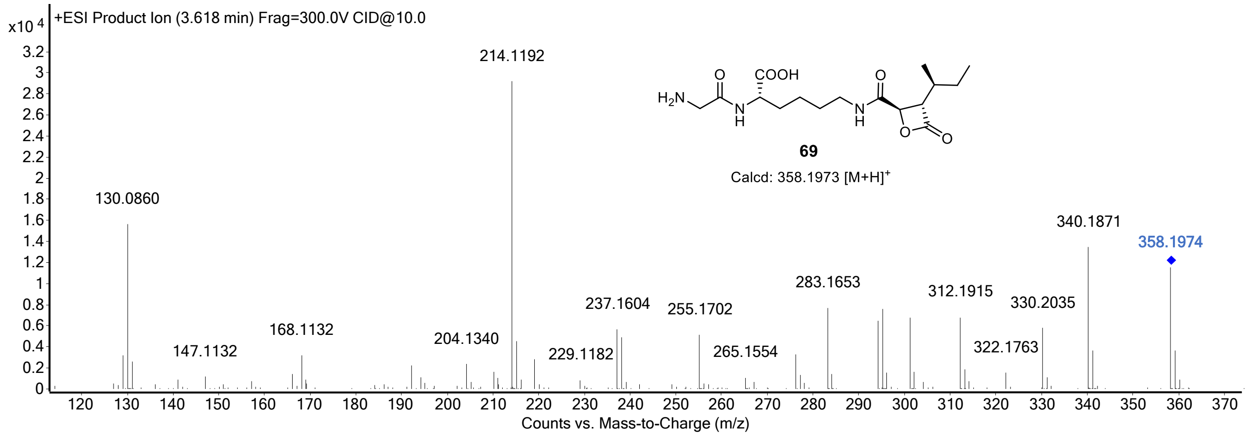

Supplementary Fig. 104. HRMS/MS data of compound 69.

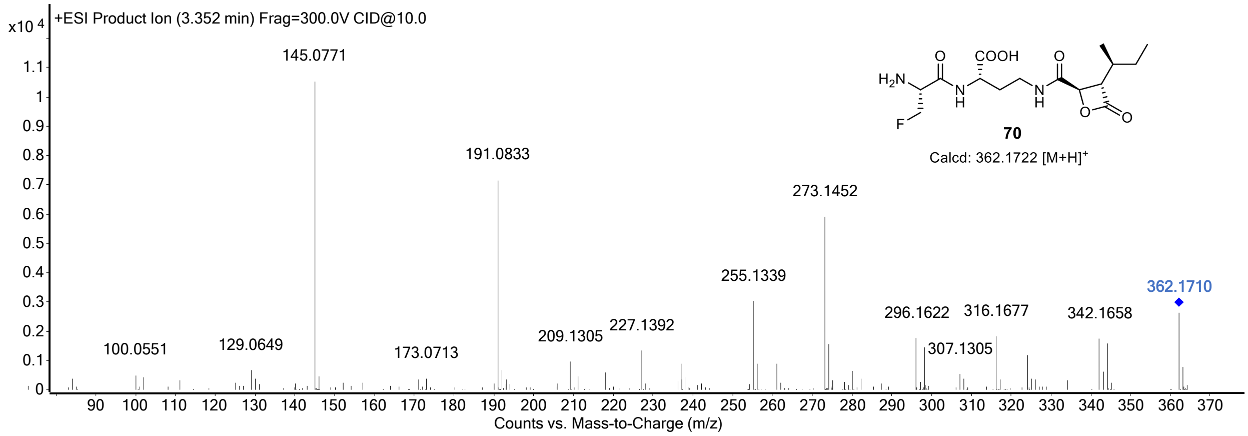

Supplementary Fig. 105. HRMS/MS data of compound 70.

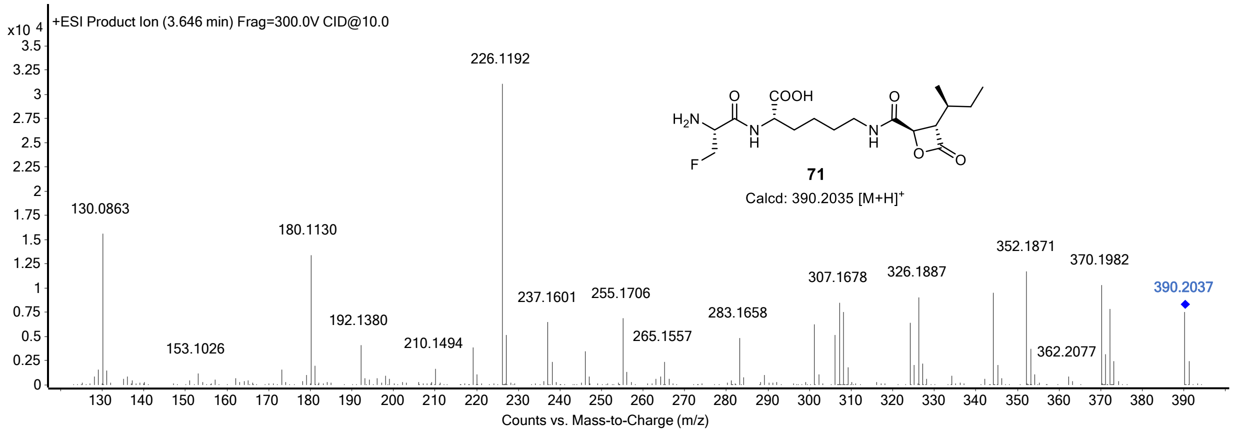

Supplementary Fig. 106. HRMS/MS data of compound 71.

Supplementary references

1. Wen, X., Leisinger, F., Leopold, V. & Seebeck, F. P. Synthetic reagents for enzyme-catalyzed methylation. *Angew. Chem. Int. Ed.* **61**, e202208746 (2022).
2. Tello-Aburto, R., Hallada, L. P., Niroulaa, D., & Rogeljb, S. Total synthesis and absolute stereochemistry of the proteasome inhibitors cystargolides A and B, *Org. Biomol. Chem.* **13**, 10127–10130 (2015).
3. Armstrong, A. & Scutt, J. N. Total synthesis of (+)-belactosin A. *Chem. Commun.* **4**, 510–511 (2004).
4. Roeder, M., Spiegelstein, O., Schurig, V., Bialer, M. & Yagen, B. Absolute configuration of the four stereoisomers of valnoctamide (2- ethyl-3-methyl valeramide), a potentially new stereospecific antiepileptic and CNS drug. *Tetrahedron Asymmetry* **10**, 841–853 (1999).
5. Niroula, D. *et al.*, Design, synthesis, and evaluation of cystargolide-based β-lactones as potent proteasome inhibitors. *Eur. J. Med. Chem.* **157**, 962–977 (2018).
6. Jiang, B., Shi, H., Xu, M., Wang, W. & Zhou, W. Stereoselective synthesis of Certonardolsterol D3. *Tetrahedron* **64**, 9738–9744 (2008).
7. Kawamura, S., Unno, Y., Asai, A., Arisawa, M. & Shuto, S. Design and synthesis of the stabilized analogs of belactosin A with the unnatural cis-cyclopropane structure. *Org. Biomol. Chem.* **11**, 6615–6622 (2013).
8. Kumaraswamy, G. *et al.* Oppolzer sultam directed aldol as a key step for the stereoselective syntheses of antitumor antibiotic belactosin C and its synthetic congeners. *J. Org. Chem.* **71**, 337–340 (2006).
9. Pirrung, M. C., Han, H. & Nunn, D. S. Kinetic mechanism and reaction pathway of thermus thermophilus isopropylmalate dehydrogenase. *J. Org. Chem.* **59**, 2423–2429 (1994).
10. De Meijere, A. *et al*., Synthesis and biological activity of simplified belactogsin C analogues, *Org. & Biomol. Chem.* **10**, 31, 6363–6374 (2012).
11. Kawamura, S. *et al.* Investigation of the noncovalent binding mode of covalent proteasome inhibitors around the transition state by combined use of cyclopropylic strain-based conformational restriction and computational modeling. *J. Med. Chem.* **56**, 5829–5842 (2013).
12. Martin, N. I., Beeson, W. T., Woodward, J. J. & Marletta, M. A. NG-aminoguanidines from primary amines and the preparation of nitric oxide synthase inhibitors. *J. Med. Chem.* **51**, 924–931 (2008).
13. Larionov, O. V. & De Meijere, A. Enantioselective total syntheses of belactosin A, belactosin C, and its homoanalogue. *Org. Lett.* **6**, 2153–2156 (2004).

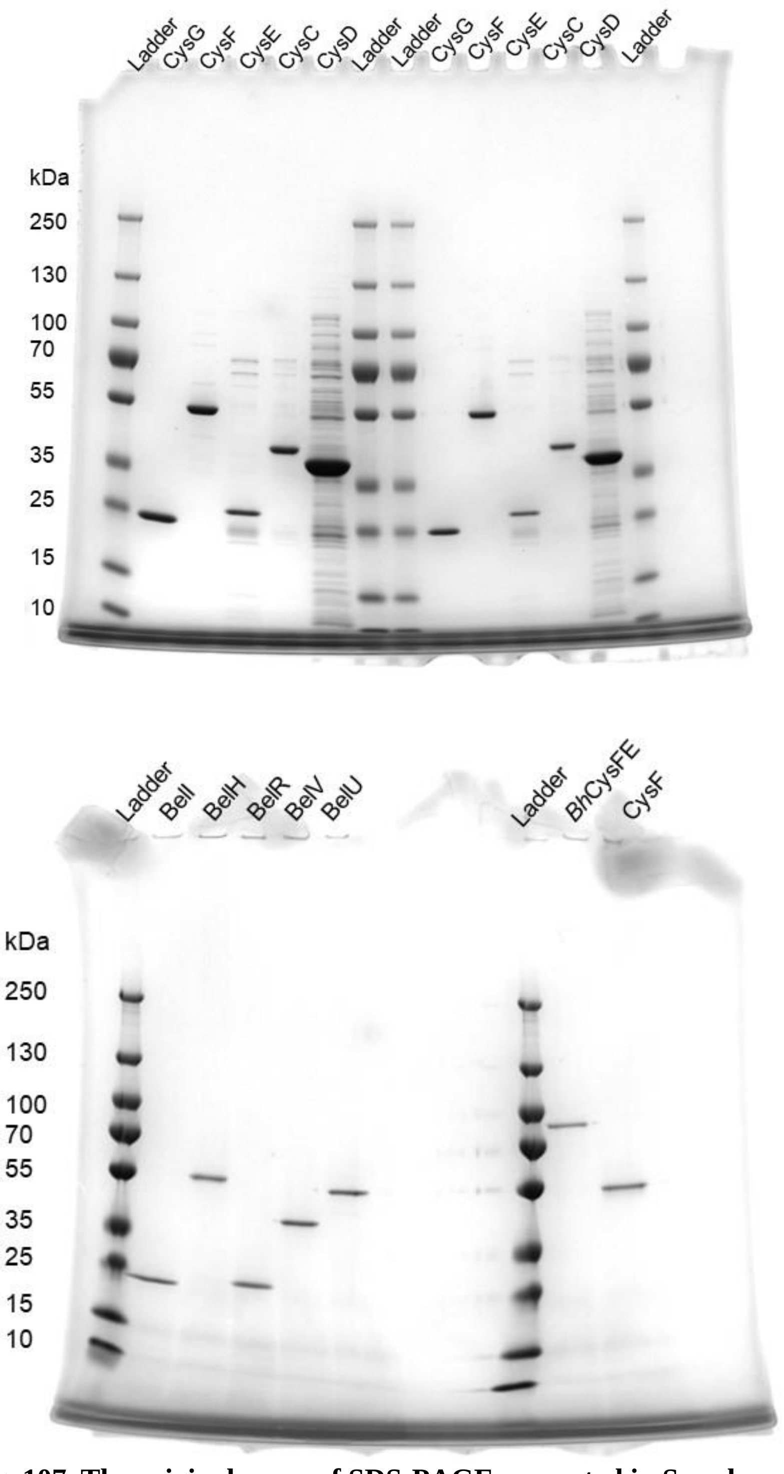

Supplementary Fig. 107. The original scans of SDS-PAGE presented in Supplementary Fig. 2
